# Prognostic Value of Vascular-Expressed PSMA and CD248 in Urothelial Carcinoma of the Bladder

**DOI:** 10.3389/fonc.2021.771036

**Published:** 2021-11-17

**Authors:** Yu Li, Keying Zhang, Fa Yang, Dian Jiao, Mingyang Li, Xiaolong Zhao, Chao Xu, Shaojie Liu, Hongji Li, Shengjia Shi, Bo Yang, Lijun Yang, Donghui Han, Weihong Wen, Weijun Qin

**Affiliations:** ^1^ Department of Urology, Xijing Hospital, Fourth Military Medical University, Xi’an, China; ^2^ Department of Urology, Tangdu Hospital, Fourth Military Medical University, Xi’an, China; ^3^ Department of Pathology, Fourth Military Medical University, Xi’an, China; ^4^ Assisted Reproduction Center, Northwest Women’s and Children’s Hospital, Xi’an, China; ^5^ Institute of Medical Research, Northwestern Polytechnical University, Xi’an, China

**Keywords:** PSMA, CD248, urothelial carcinoma of the bladder, prognosis, angiogenesis

## Abstract

**Background:**

Urothelial carcinoma of the bladder (UCB) is a common cancer of the urinary system. Despite substantial improvements in available treatment options, the survival outcome of patients with advanced UCB is unsatisfactory. Therefore, it is necessary to identify new prognostic biomarkers for monitoring and therapy guidance of UCB. In recent years, prostate-specific membrane antigen (PSMA) and CD248 have been identified promising candidate bio7markers.

**Methods:**

In this study, we first examined PSMA and CD248 expression in tissues from 124 patients with UCB using immunohistochemical and immunofluorescent staining. We then analyzed the association between the expression of the two biomarkers and other clinicopathological features and prognosis. Finally, we performed bioinformatic analysis of *CD248* and *FOLH 1 (PSMA)* using the TCGA-BLCA dataset to explore the underlying mechanism of PSMA and CD248 in the progression of UCB.

**Results:**

Among the 124 cases, PSMA and CD248 were confirmed to be expressed in tumor-associated vessels. Vascular PSMA and CD248 expression levels were associated significantly with several deteriorated clinicopathological features. Furthermore, using univariate and multivariate Cox analyses, high vascular PSMA and CD248 expression levels were observed to be associated significantly with poor prognosis in patients with UCB. As risk factors, both PSMA and CD248 expression showed good performance to predict prognosis. Furthermore, combining these vascular molecules with other clinical risk factors generated a risk score that could promote predictive performance. Bioinformatic analysis showed that both PSMA and CD248 might contribute to angiogenesis and promote further progression of UCB.

**Conclusion:**

Both PSMA and CD248 are specifically expressed in the tumor-associated vasculature of UCB. These two molecules might be used as novel prognostic biomarkers and vascular therapeutic targets for UCB.

## Introduction

Urothelial carcinoma of the bladder (UCB), the commonest type of bladder cancer (BLCA), is the 10^th^ most common cancer, with an estimated 549,000 new diagnoses and 200,000 deaths annually worldwide (1). More than 90% of bladder cancers are derived from the urothelium and are known for their ability to metastasize and recur, especially when they invade muscles (2). About half of the patients with muscle-invasive UCB die of the disease, and traditional chemotherapy cannot increase their survival significantly. Although the survival of patients with non-muscle-invasive UCB is relatively good, tumor recurrence might occur in about two-thirds of them (3). In recent years, remarkable advances have been made in clinical prognostic diagnosis and effective immunotherapies, for instance, immune checkpoint inhibitor therapeutics; however, the response rate of patients with advanced UCB was only 30%, and the extension of survival time remains limited (4).

Theoretically, tissue biomarkers can be used in UCB to predict oncological outcomes, such as recurrence and progression, as well as the response to intravesical drug perfusion. *P53*, the most common tumor suppressor gene that is mutated in most human cancers, is associated with the most aggressive non−muscle−invasive bladder cancer (NMIBC) and muscle-invasive bladder cancer (MIBC) as a prognostic biomarker (5). However, it cannot predict the response to Bacillus Calmette–Guérin (BCG) therapy, and the heterogeneity of the included studies and limitations related to immunohistochemistry precluded any clear conclusions (6, 7). Meanwhile, a phase III trial designed to evaluate the benefit in patients with MIBC based on their p53 status for adjuvant cisplatin-based chemotherapy could not confirm the prognostic value of p53 alteration (8). Apoptosis biomarkers, such as survivin, might be associated with outcomes in NMIBC (9, 10); however, although survivin improved the accuracy of prediction of disease recurrence significantly in a subgroup of patients with pT2-3N0M0 disease (11), the evidence was insufficient for prognostic prediction in MIBC, and large prospective series are still lacking (12). Cell signaling pathway biomarkers such as ErbB (Erb-B2 receptor tyrosine kinase) and fibroblast growth factor receptor (FGFR) family members, angiogenesis biomarkers [vascular endothelial growth factor (VEGF), MVD (mevalonate diphosphate decarboxylase), and hypoxia inducible factor 1 alpha (HIF-1α)], and tumor cell invasion biomarkers (E-cadherin and N-cadherin) have been shown to be related to the outcomes of NMIBC and MIBC (13–15). However, none of the evaluated tissue biomarkers alone could be used to predict oncological outcomes with sufficient accuracy to change decisions in routine clinical practice (12, 16). Thus, it is necessary to identify new independent prognostic biomarkers for monitoring and therapy guidance of UCB.

Prostate specific membrane antigen (PSMA), also known as folate hydrolase 1 (FOLH1) or glutamate carboxypetidase II, is a type II transmembrane glycoprotein, which has folate hydrolase and neurocarboxypeptidase activity. PSMA is expressed specifically on prostate epithelial cells, and its expression is upregulated markedly in prostate cancer (PCa). In recent years, PSMA has also been found to be expressed in the vasculature of non-prostatic solid tumors, such as breast cancer (17, 18), lung cancer (19, 20), gastric cancer (21), colorectal cancer (21, 22), kidney cancer (23), and glioblastoma (24, 25), but not in normal vascular endothelial cells. Thus, PSMA has also been considered as an effective target for the cancers with vascular PSMA expression (26). CD248, also known as tumor endothelial marker 1 (TEM1) or Endosialin, is a transmembrane glycoprotein that belongs to the C-type lectin-like receptor family (27). Importantly, CD248 is overexpressed specifically in tumor-associated fibroblasts and pericytes residing in tumor blood vessels, but is barely expressed in normal tissues (28, 29), making CD248 an oncofetal protein with potential as a biomarker and therapeutic target.

In our previous study, we found that PSMA and CD248 were both expressed specifically in the vasculature in hepatocellular carcinoma (HCC), and vascular-expressed PSMA and CD248 might be used as prognostic markers and vascular therapeutic targets for HCC (30, 31). We also found that overexpression of CD248 in renal cell carcinoma (RCC) was related to poor prognosis (32). Therefore, we wondered whether vascular-expressed PSMA and CD248 could be promising biomarkers for UCB. Although several studies have shown that PSMA is expressed in the vasculature of bladder cancer (33–35), these studies only examined limited samples, or did not analyze the association between vascular PSMA expression and clinicopathological features and prognosis systematically. Besides, the vascular expression pattern of CD248 and its prognostic value in UCB remains unknown.

The present study aimed to determine whether vascular-expressed PSMA and CD248 are biomarkers for UCB. First, we examined PSMA and CD248 expression in 124 UCB tissues using immunohistochemistry (IHC), and analyzed the association between the expression levels of the two biomarkers and other clinicopathological features and prognosis. Then, we constructed PSMA-based and CD248-based prognostic signatures by integrating multiple clinical variables, which might promote the predictive accuracy. Finally, we performed bioinformatic analysis of *CD248* and *PSMA* using the Cancer Genome Atlas (TCGA)-BLCA dataset to explore the underlying mechanism of PSMA and CD248 in UCB progression.

## Materials and Methods

### Patients and Follow-Up

In this retrospective study, 162 UCB specimens were chosen from patients who underwent surgery [transurethral resection of bladder tumor (TRUBT) or radical cystectomy (RC)] at Xijing Hospital from 2006 to 2018. Thirty-eight samples were excluded because (1) medical records or information was lacking; (2) the pathological diagnosis included other primary tumors; (3) there was missing follow-up data; and (4) the patients or their families refused to provide pathological specimens for clinical research. Finally, a total of 124 UCB specimens from 124 patients with UCB were included in this study.

This study was approved by the Ethics Committee of Xijing Hospital, and all of the participating patients gave their informed written consent. Representative formalin-fixed paraffin-embedded tumor blocks were obtained from the Department of Pathology at Xijing Hospital. Patients were followed up from the date of surgery, with an average follow-up period of 42 months (1–144 months). Detailed pathological diagnosis was provided by three experienced pathologists according to the American Joint Committee on Cancer (AJCC) Cancer Staging Manual (Version 9) (36) and the 2016 WHO Classification of Tumors of the Urinary System and Male Genital Organs (37). The clinicopathological features of the patients were obtained from the electronic medical records of Xijing Hospital.

### Immunohistochemistry Staining

Four-micron-thick tissue pieces were cut from representative wax blocks of UCB tissues. Slides were then subjected to IHC to evaluate PSMA and CD248 expression, respectively. Briefly, slides were deparaffinized in xylene and rehydrated through a graded alcohol series, before the antigen was retrieved using a high temperature and pressure of 10 nM citrate buffer (pH 6.0). Endogenous peroxidase activity was inactivated using 3% H_2_O_2_, and non-specific binding was blocked using non-immune serum. Primary antibodies were then applied and incubated overnight in a humidified chamber at 4°C. Next day, the slides were washed three times with phosphate-buffered saline (PBS), followed by incubation with horseradish peroxidase (HRP)-labeled secondary antibody at room temperature for 30 min. The slides were then washed three times with PBS, and visualization was performed using 3, 3’-diaminobenzidine (DAB) chromogen for 2 to 3 min. The slides were counterstained with hematoxylin, rinsed in water, dehydrated in ascending concentrations of ethanol, subjected to xylene clearance, and cover-slipped permanently for light microscopy. The primary antibodies used for IHC were Anti-human CD248 (#ab204914, Abcam, Cambridge, UK), anti-human PSMA (#ab76104, Abcam), and anti-human CD31 antibody (#3528, Cell Signaling Technology, Danvers, MA, USA). The IHC kit was purchased from Fuzhou Maixin Reagent Co., Ltd. (Fuzhou, China). All procedures were carried out according to the manufacturer’s instructions. Sections were analyzed under a light microscope (Leica DM2500; Leica Microsystems, Wetzlar, Germany), and images were acquired using a Leica DFC 490 system (Leica Microsystems).

### Immunofluorescent Staining

Triple immunofluorescent (IF) staining of PSMA, CD248, and CD31 was conducted for paraffin sections of UCB tissues. Briefly, the sections were incubated at room temperature with a mixture of anti-human CD248 (#ab204914, Abcam), anti-human PSMA (#ab76104, Abcam), and anti-human CD31 antibody (#3528, Cell Signaling Technology) for 16–18 h. The sections were then incubated with a mixture of Alexa488-conjugated donkey anti-goat IgG (#ab6721, Abcam), Cy3-conjugated donkey anti-rabbit IgG (#96907, Abcam), and Alexa647-conjugated donkey anti-mouse IgG (#ab150076, Abcam) for 4 h at room temperature. Nuclei were stained with 4'6-diamidino-2-phenylindole (DAPI) (#C1002, Beyotime, Jiangsu, China) or Hoechst 33342 (#C1022, Beyotime). The slides were covered, sealed with Vectashield (Vector, Burlingame, CA, USA), and observed under a confocal laser scanning microscope (FV1000; Olympus, Tokyo, Japan). The digital images were captured and processed using FV10-ASW 1.6 software (Olympus).

### Evaluation of Staining

CD31 staining in serial sections was used to identify the tumor-associated vasculature. To evaluate vascular PSMA and CD248 expression, first we randomly selected three fields under low magnification (100×) and counted the number of CD31^+^ vascular structures. Then, we chose the fields with a microvessel density (MVD) greater than 40 as hot-spot areas, and examined PSMA and CD248 expression in three of these fields under high magnification (200×). Vascular PSMA and CD248 expression was assessed in a semiquantitative manner. Lesions with no detectable PSMA or CD248 expression were scored as “0”; lesions with staining of PSMA or CD248 in 1–50% of the vasculatures were scored as “1”; and lesions with staining of PSMA or CD248 in >50% of the vasculatures were scored as “2”. For statistical analysis, the samples with a staining score of 0 and 1 were grouped as “low expression”, and samples with a staining score of 2 were grouped as “high expression”.

### Nomogram Construction

Cox regression analysis and logistic regression analysis were adopted to construct vascular-CD248/PSMA-based signatures, accompanied by clinicopathological variables [i.e., age, sex, clinical grade, invasive stage, differentiation status, and Ki-67 (marker of proliferation Ki-67) expression]. The regression coefficients were used to weight the variables of the model, and a nomogram was constructed for visualization.

### Bioinformatic Analysis of *CD248* and *PSMA* Using TCGA-BLCA Dataset

#### Data Source and Preprocessing

First, 413 sets of BLCA data and 19 sets of non-tumor data were downloaded from the TCGA portal (https://portal.gdc.cancer.gov/). Then, transcriptomic data [RNA-Seq, as Fragments Per Kilobase of transcript per Million mapped reads (FPKM)] and clinical information were integrated according to their ID numbers, and within-array replicate probes were replaced with their average using the limma package of the R software. All data were processed and analyzed with the R software (https://www.r-project.org/).

#### Prognostic Value Analysis

Microenvironment Cell Population-counter (MCP-counter) in the R package was adopted to estimate “single sample” scores of endothelial cells from the TCGA-BLCA expression matrix. Then, patients in the TCGA-BLCA data were divided into high-expression and low-expression groups according to the median level of CD248, PSMA, and endothelial cell score, respectively. Kaplan–Meier survival analysis was performed to evaluate the prognostic value, and the Pearson correlation coefficient test was employed to assess the correlation between endothelial cells and CD248 and PSMA. P < 0.05 was considered statistically significant.

#### Gene Ontology Enrichment Analysis

Differentially expressed genes (DEGs) between tumor and normal tissue were analyzed using Wilcox tests. The P-value was adjusted using the false discovery rate (FDR), with filter criteria of FDR < 0.05 and |log2 fold-change [FC]| > 1. Then, CD248 and PSMA-correlated DEGs (Cor-DEGs) were selected using the Pearson correlation coefficient test with the filter criteria of |correlation coefficient| > 0.5 and P < 0.001. Subsequently, GO functional enrichment of Cor-DEGs were performed *via* clusterProfiler and enrichplot in the R package, and were visualized using a chord plot through Goplot in the R package. FDR < 0.05 was considered statistically significant.

#### Transcription Factors–Based Regulatory Network

The list of TFs was retrieved from the Cistrome website (https://cistrome.org), and differentially expressed TFs (DETFs) were identified by matching with the DEGs. Meanwhile, univariate Cox regression analysis, with a threshold value of P < 0.01, was used to identify possible prognostic Cor-DEGs (PCor-DEGs). Subsequently, the correlation between DETFs and PCor-DEGs was analyzed, and a TFs-based regulatory network was established using Cytoscape 3.6.0. The threshold of a correlation coefficient > 0.3 and P < 0.05 was employed as the cutoff value.

### Statistical Analysis

All statistical analysis was performed using IBM SPSS statistical software (version 26; IBM Corp., Armonk, NY, USA). Descriptive statistics, such as median, range, and absolute and relative frequencies, were calculated to define the characteristics of the study cohort. The chi-squared test was used to assess the association between PSMA or CD248 expression and various clinicopathological features. Survival time was defined from the day of surgery until death. A survival curve was generated using the Kaplan-Meier method and compared using the log-rank test. Hazard ratios (HR) with corresponding 95% confidence intervals (CI) were estimated using Cox proportional hazards models. A risk plot of the PSMA/CD248-based signature was prepared using the R software. The median risk score was used as the cutoff value. P values < 0.05 were considered to be statistically significant.

## Results

### Vascular Expression of PSMA in UCB and Its Comparison With Clinicopathological Parameters

**Figure 1:**
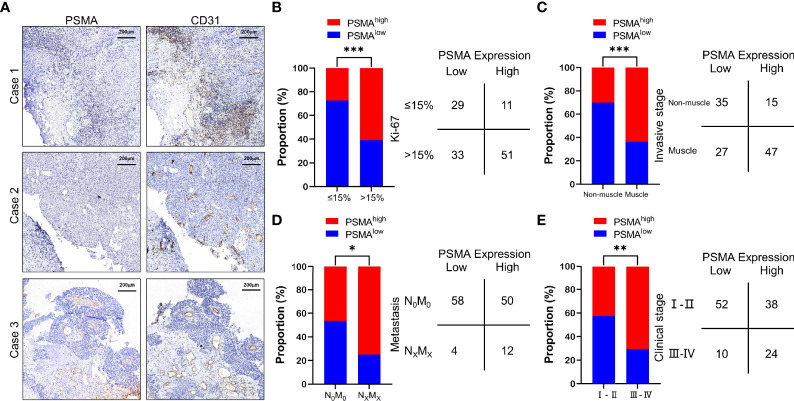
PSMA expression in the tumor-associated vasculature of UCB and the comparison with clinicopathological parameters. **(A)** Representative IHC staining to show different PSMA expression levels in HCC, using CD31 as the positive control. Case 1: No PSMA expression (score = 0), Case 2: Positive PSMA expression in ≤50% of the tumor-associated vasculature (score = 1), and Case 3: Positive PSMA expression in >50% of the tumor-associated vasculature (score = 2). **(B)** Comparison between PSMA expression and the Ki-67 index. **(C)** Comparison between PSMA expression and invasive stage. **(D)** Comparison between PSMA expression and metastasis. **(E)** Comparison between PSMA expression and clinical stage. Scale bar = 200 μm. Representative images are shown. *P < 0.05; **P < 0.01; ***P < 0.001.

To evaluate PSMA expression in UCB, we performed IHC staining in UCB tissues from 124 patients. The clinicopathological parameters of the patients were summarized in **Table 1**. Among these patients, 62 (50.00%) showed PSMA expression in >50% of the tumor-associated vasculature (score 2), 22 (17.74%) showed PSMA expression in ≤50% of tumor-associated vasculature (score 1), while 40 (32.26%) did not show detectable PSMA expression (score 0) (**Table 2**). Representative cases with different PSMA expression levels are shown in **Figure 1A**, and the vascular structure was confirmed by staining for CD31, a well-established endothelial cell marker. These results indicated that PSMA was specifically expressed in the vasculature of patients with UCB. Patients with PSMA expression in >50% of the tumor-associated vasculature were designated as the high PSMA expression group, while those with PSMA expression in ≤50% of the tumor-associated vasculature or no detectable PSMA expression were designated as the low PSMA expression group.

**Table 1:** Clinical and pathological characteristics of the patients with UCB in this study (n = 124).

Characteristics	Value or number of patients
Sex (%)	
Male	104 (83.87)
Female	20 (16.13)
Age, years	
Mean ± SD	64.27 ± 11.64
Median (range)	66.5 (26–87)
Tumor differentiation (%)	
Low grade	84 (67.74)
High grade	40 (32.26)
TNM stage (%)	
T stage	
T1	51 (41.13)
T2	43 (34.68)
T3	16 (12.90)
T4	14 (11.29)
N stage (%)	
N0	108 (87.10)
N1	12(9.68)
N2	4 (3.22)
M stage (%)	
M0	123 (99.19)
M1	1 (0.81)
Clinical stage (%)	
Non-muscle invasive	
I	50 (40.32)
Muscle invasive	
II	40 (32.26)
III	32 (25.81)
IV	2 (1.61)

**Table 2:** Expression of PSMA and CD248 in tumor-associated vasculature of UCB patient*s* (n = 124).

Number of BLCA (%)	Expression score			Correlation analysis	
	0	1	2		
**PSMA**	40 (32.26)	22 (17.74)	62 (50.00)	r = 0.3935	P < 0.0001^*^
**CD248**	8 (6.45)	38 (30.65)	78 (62.90)		

*Statistically significant (P < 0.05).

We then analyzed the association between PSMA expression and clinicopathological features. The results showed that PSMA expression was associated with several clinicopathological features of UCB, such as the Ki-67 index, invasive stage, tumor metastasis, and clinical stage. According to the difference in the Ki-67 index, we divided all cases into two groups based on a Ki-67 index >15% and a Ki-67 indexes ≤15%, because it has been reported that the Ki-67 index of normal bladder tissue is less than 15% (38). Patients with high PSMA expression had significantly higher Ki-67 indexes compared with those of patients with low PSMA expression (χ^2^ = 11.960, P = 0.0005) (**Figure 1B** and **Table S1**). For invasive stage, patients with muscle invasion had significantly higher PSMA expression levels than those with non-muscle invasion (χ^2^ = 13.410, P = 0.0003) (**Figure 1C** and **Table S1**). For tumor metastasis, patients with high PSMA expression were more likely to have more lymph node metastasis and distant metastasis than patients with low PSMA expression (χ^2^ = 4.594, P = 0.0321) (**Figure 1D** and **Table S1**). Patients in clinical stage III–IV were more likely to have high PSMA expression than those in clinical I–II stage (χ^2^ = 7.942, P = 0.0048) (**Figure 1E** and **Table S1**). PSMA expression was not associated significantly with other clinicopathological parameters, such as age, sex, tumor differentiation, and tumor invasion (**Table S1**).

### Vascular Expression of CD248 in UCB and Its Comparison With Clinicopathological Parameters

**Figure 2:**
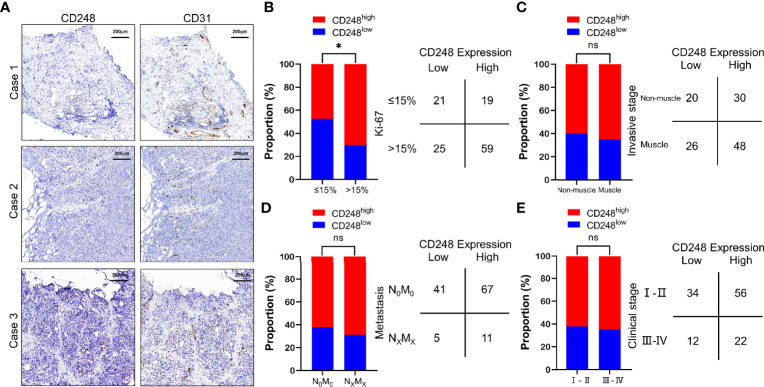
CD248 expression in the tumor-associated vasculature of UCB and the comparison with clinicopathological parameters. **(A)** Representative IHC staining to show different CD248 expression levels in HCC, using CD31 as the positive control. Case 1: No CD248 expression (score = 0), Case 2: Positive CD248 expression in ≤50% of the tumor-associated vasculature (score = 1), and Case 3: Positive CD248 expression in >50% of the tumor-associated vasculature (score = 2). **(B)** Comparison between CD248 expression and the Ki-67 index. **(C)** Comparison between CD248 expression and invasive stage. **(D)** Comparison between CD248 expression and metastasis. **(E)** Comparison between CD248 expression and clinical stage. Scale bar = 200 μm. Representative images are shown. *P < 0.05; ns, no statistically significant.

To evaluate CD248 expression in UCB, we further performed IHC staining of CD248 in UCB tissues from the same 124 patients. Among these patients, 78 (62.90%) showed CD248 expression in >50% of the tumor-associated vasculature (score 2), 38 (30.65%) showed CD248 expression in ≤50% of the tumor-associated vasculature (score 1), respectively, while 8 (6.45%) did not show detectable CD248 expression (score 0) (**Table 2**). Representative cases with different CD248 expression levels are shown in **Figure 2A**, and the vascular structure was confirmed by the staining for CD31. These results indicated that CD248 was also specifically expressed in the vasculature of a subset of patients with UCB. The results showed that difference in CD248 expression remained statistically significant for the different Ki-67 index groups. Patients with high CD248 expression had a significantly higher Ki-67 index compared with that in patients with low PSMA expression (χ^2^ = 6.004, P = 0.0143) (**Figure 2B** and **Table S2**). While CD248 expression showed no significant association with other clinicopathological parameters, such as age, sex, tumor differentiation, tumor invasion, tumor metastasis, clinical stage, and invasive stage (**Figures 2C–E** and **Table S2**).

### Correlation Between the Expression of CD248 and PSMA in UCB Vessels

**Figure 3:**
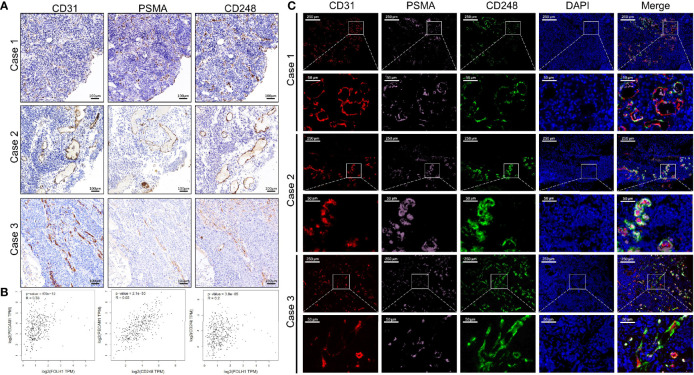
The correlation between the expression levels of CD248 and PSMA in UCB vessels. **(A)** Representative IHC staining in serial paraffin sections of three cases with UCB, using CD31 as the positive control. Scale bar = 100 μm. **(B)** The correlation between the expression levels of *CD31*, *PSMA*, and *CD248* in UCB, respectively, using data from the GEPIA database. **(C)** Representative triple-immunofluorescence histochemistry for CD31 (red), PSMA (pink), and CD248 (green) in serial paraffin sections of three cases with UCB. Scale bar = 250 and 50 μm, respectively. Representative images are shown.

On the basis of the previous results, we performed Spearman correlation analysis on the data, and the results showed that the expression of CD248 in the tumor-associated vasculature correlated significantly and positively with PSMA expression (r = 0.3935, P < 0.0001) (**Table 2**). To intuitively evaluate CD248 and PSMA expression in vessels of UCB, we performed IHC and IF staining in UCB tissues. **Figure 3A** shows that in the serial paraffin sections of UCB tissues, using platelet endothelial cell adhesion molecule-1 (PECAM-1/CD31) as the positive control, the positive expression sites of PSMA and CD248 in blood vessels were basically the same. There was a positive correlation between the expression of CD31, PSMA, and CD248 in UCB, which was verified using data from the GEPIA database (http://gepia.cancer-pku.cn/) (**Figure 3B**). Furthermore, as shown in **Figure 3C**, by co-staining with CD31, both CD248 and PSMA were positive in the blood vessels of UCB, especially in the neovascularization, and their expression locations were basically the same.

### Prognostic Value of Vascular CD248 and PSMA Expression in UCB

**Figure 4:**
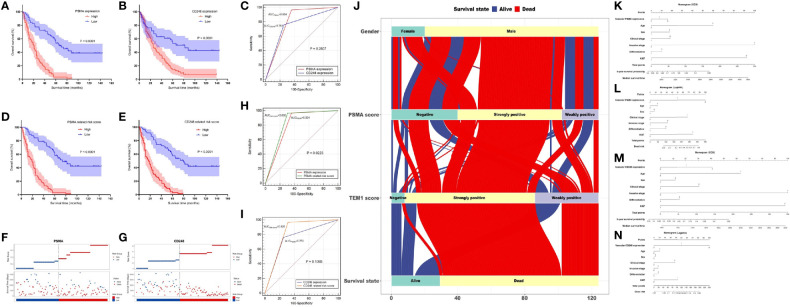
Prognostic value of vascular CD248 and PSMA expression in UCB. **(A, B)** Kaplan–Meier survival curve showing the association between vascular PSMA **(A)**/CD248 **(B)** expression and overall survival of patients with UCB. **(C)** ROC curves for PSMA and CD248 expression. **(D, E)** Kaplan–Meier survival curve showing the association between **(D)** PSMA-/**(E)** CD248-related risk score and overall survival of patients with UCB. **(F, G)** The distribution of the **(F)** PSMA-/**(G)** CD248**-**related risk score and the survival status of each patient. **(H)** ROC curves of PSMA expression and the PSMA-related risk score. **(I)** ROC curves of CD248 expression and the CD248-related risk score. **(J)** The flow direction of the final outcome of patients with different expression levels of PSMA and CD248 in a Sankey plot. Nomogram of the vascular-PSMA-based signature for survival probability prediction **(K)** and risk of death prediction **(L)**. Nomogram of the vascular-CD248-based signature for survival probability prediction **(M)** and risk of death prediction **(N)**.

Analysis of data from the GEPIA database showed that overexpression of *CD31* could partly predict worse prognosis of patients with UCB (**Figure S1**). Therefore, to determine the prognostic value of PSMA and CD248 expression in UCB, we first analyzed the association between vascular PSMA or CD248 expression and overall survival (OS). Survival curves of patients with different CD248 and PSMA expression levels were generated. As shown in **Figure 4A, B**, patients with high PSMA and CD248 expression tended to have a shorter survival time compared with patients with low PSMA and CD248 expression, respectively (P < 0.001). Then, we performed a semiquantitative receiver operating characteristic (ROC) curve analysis and compared the results at the patient-level for UCB to determine the ability to predict the survival outcome of patients according to the expression of these two biomarkers in UCB vessels. As shown in **Figure 4C**, the ROC curves of PSMA expression and CD248 expression had sensitivities of 96.55 and 75.86%, respectively, and specificities of 64.21% and 74.74%, respectively. The area under the ROC curve (AUC) values of PSMA expression and CD248 expression were 0.804 (95% CI, 0.723–0.870) and 0.753 (95% CI, 0.668–0.826) respectively, which indicated that the two biomarkers might have similar prognostic prediction performances (P = 0.2807).

Furthermore, we carried out the Cox regression analysis to evaluate the prognostic factors for OS of UCB. Univariate analysis showed that vascular PSMA expression (P < 0.001), vascular CD248 expression (P < 0.001), age (P = 0.016), clinical stage (P < 0.001), invasive stage (P < 0.001), and Ki-67 index (P < 0.001) could be used as prognostic factors (**Table 3**). Multivariate analysis was then carried out between other prognostic factors of UCB and the vascular expression of PSMA or CD248, respectively. Multivariate analysis 1 revealed that vascular PSMA expression (P < 0.001), clinical stage (P < 0.001), and Ki-67 index (P = 0.002) were associated with OS of UCB (**Table 3**), meanwhile, multivariate analysis 2 revealed that vascular CD248 expression (P < 0.001), clinical stage (P < 0.001), and Ki-67 index (P<0.001) were associated with OS of UCB (**Table 3**).

**Table 3:** Cox regression analysis of prognostic factors for overall survival in UCB (n = 124).

	Univariate analysis			Multivariate analysis 1			Multivariate analysis 2		
	HR	95% CI	P Value	HR	95% CI	P Value	HR	95% CI	P Value
Vascular PSMA expression (High *vs.* Low)	4.744	3.010–7.477	<0.001^*^	3.517	2.138–5.785	<0.001^*^	–		
Vascular TEM1 expression (High *vs.* Low)	2.764	1.717–4.450	<0.001^*^	–			2.581	1.548–4.302	<0.001^*^
Age (≥60 *vs.* <60)	1.718	1.105–2.669	0.016^*^	1.149	0.727–1.817	0.551	1.207	0.759–1.919	0.427
Sex (Male *vs.* Female)	1.073	0.626–1.837	0.798	1.013	0.579–1.775	0.963	0.933	0.528–1.648	0.811
Clinical stage (III–IV *vs.* I–II)	3.943	2.519–6.173	<0.001^*^	2.633	1.548–4.478	<0.001^*^	2.954	1.742–5.008	<0.001^*^
Invasive stage (Muscle *vs.* Non-muscle)	2.246	1.460–3.453	<0.001^*^	1.360	0.815–2.269	0.239	1.593	0.952–2.664	0.076
Differentiation (High grade *vs.* Low grade)	0.680	0.435–1.064	0.092	0.928	0.585–1.471	0.751	0.982	0.616–1.564	0.982
Ki-67 (>15 *vs.* ≤15%)	2.878	1.765–4.691	<0.001^*^	2.258	1.345–3.790	0.002^*^	2.591	1.549–4.335	<0.001^*^

HR, hazard ratio; CI, confidence interval.

*Statistically significant (P <0.05).

### Vascular CD248/PSMA Based Signature With an Enhanced Predictive Performance

We weighted each prognostic factor according to the results of the multivariate analysis in **Table 3**. The specific contents were described as follows: High expression of PSMA was defined as “2”, and low expression was defined as “1”; high expression of CD248 was defined as “2”, and low expression was defined as “1”; clinical stage III–IV was defined as “2”, and the clinical stage I–II was defined as “1”; and a Ki-67 index >15% was defined as “2”, and an index ≤15% was defined as “1”. Subsequently, the risk score for each patient was calculated using the following formula:


PSMA−related risk score=(3.517× the expression level of PSMA)+(2.633×clinical stage)+(2.258×Ki−67 index)

CD248−related risk score=(2.581×the expression level of CD248)+(2.954×clinical stage)+(2.591×Ki−67 index)

According to the median risk scores of PSMA (11.925) and CD248 (13.298), individuals were sorted into a high-risk (n = 67) and a low-risk group (n = 57) according to PSMA and a high-risk (n = 66) and a low-risk group (n = 58) according to CD248. The Kaplan–Meier survival analysis showed that the prognoses according to the PSMA-related risk score and CD248-related risk score were both worse in the high-risk group than in the low-risk group (P1 < 0.0001, P2 < 0.0001, **Figures 4D, E**). Then, we ranked patients by their PSMA and CD248-related risk scores and analyzed their survival status, respectively. The results showed a greater number of deaths in the high-risk group, especially according to the PSMA and CD248 risk score (**Figures 4F, G**). Then, we constructed ROC curves of the PSMA and CD248-related risk score, and made a comparison with the corresponding PSMA and CD248 expression levels, respectively. As shown in **Figure 4H**, the AUC value of the PSMA-related risk score was statistically larger than that of PSMA expression (P = 0.0223) (**Figure 4H**). However, there was no statistical difference between the AUC values of CD248 expression and the CD248-related risk score (P = 0.1093) (**Figure 4I**). A Sankey plot was used, which intuitively illustrated that as an independent risk factor, PSMA- or CD248-related death was largely associated with the strong positive expression group, instead of the weakly positive or negative group (**Figure 4J**).

The nomogram of the vascular-CD248/PSMA-based signature constructed by Cox regression analysis could be used to predict the 5-year survival probability and median survival time of patients with UCB (**Figures 4K, M**). In addition, another nomogram constructed using logistic regression analysis could be employed to predict the risk of death (**Figures 4L, N**).

### Prognostic Value of CD248 and PSMA in TCGA-BLCA

**Figure 5:**
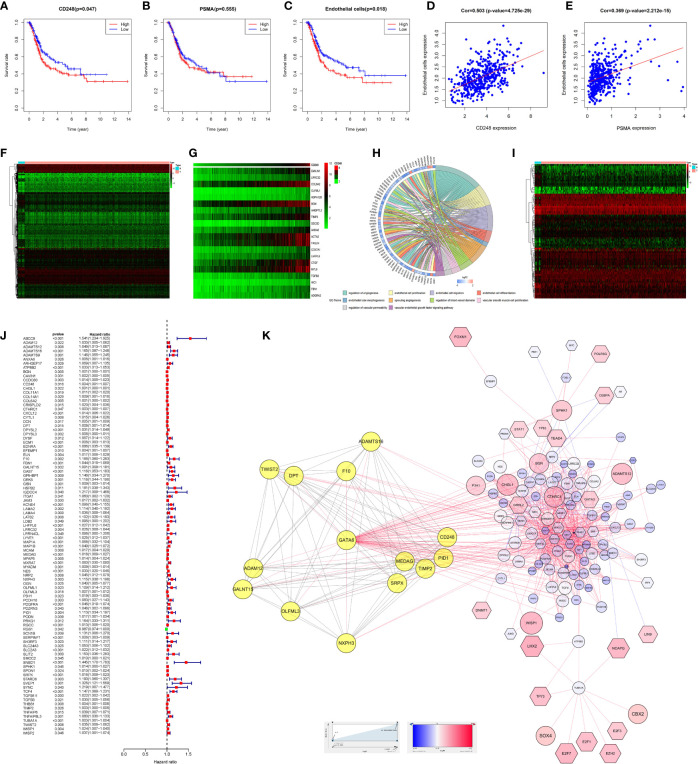
Bioinformatic analysis of *CD248* and *PSMA* using TCGA-BLCA dataset. Kaplan-Meier curve of *CD248* **(A)**, *PSMA* **(B)**, and the endothelial cell score **(C)**. Correlation between the endothelial cell score and *CD248* expression **(D)**. Correlation between the endothelial cell score and *PSMA* expression **(E)**. Heat map of DEGs **(F)**. Co-expressed heatmap of top 20 Cor-DEGs **(G)**. Green to red spectrum indicates low to high gene expression. GO enrichment analysis of Cor-DEGs **(H)**. Heat map of DETFs **(I)**. Forest graph of PCor-DEGs **(J)**. The red and green dots represent PCor-DEGs with a hazard ratio >1 and ≤1, respectively. TFs-based regulatory network for PCor-DEGs **(K)**. P < 0.05 or FDR < 0.05 was considered statistically significant.

Survival analysis indicated that highly CD248 expression accompanied a bad prognosis (P < 0.05, **Figure 5A**), while the expression level of PSMA did not significantly affect the overall survival of patients with BLCA (P > 0.05, **Figure 5B**). Additionally, patients with a high endothelial cell score exhibited a worse survival outcome (P < 0.05, **Figure 5C**). The endothelial cell score was related positively to the expression level of *CD248* and *PSMA* (P < 0.0001, **Figures 5D, E**), indicating that these two genes might contribute to angiogenesis, which was consistent with our aforementioned results.

### GO Enrichment Analysis of Cor-DEGs in TCGA-BLCA

To explore the function of CD248 and PSMA in angiogenesis, GO enrichment analysis of Cor-DEGs was performed. First, we selected 343 Cor-DEGs from among 3,126 DEGs in the TCGA-BLCA dataset, and the top 20 Cor-DEGs that correlated with CD248 and PSMA were adopted to develop a co-expression heatmap (**Figures 5F, G**; Supporting Data 1 and 2). Then, as shown in **Figure 5H**, “Regulation of angiogenesis”, “Endothelial cell proliferation”, “Endothelial cell migration”, “Endothelial cell differentiation”, “Endothelial tube morphogenesis”, “Sprouting angiogenesis”, and “Vascular endothelial growth factor signaling pathway” GO terms were identified as significantly enriched by Cor-DEGs (**Figure 5H**, FDR < 0.05).

### TFs-Based Regulatory Network for PCor-DEGs

To explore the underlying mechanism of the Cor-DEGs in BLCA angiogenesis and progression, 77 DETFs and 96 PCor-DEGs were screened from the DEGs and Cor-DEGs, respectively (**Figures 5I, J**; Supporting Data 3 and 4). Then, a TFs-based regulatory network for PCor-DEGs was visualized (**Figure 5K**; Supporting Data 5). In the regulatory network, several hub genes with maximum intramodular connectivity were identified (i.e., *GATA6*, *SOX17*, *MEF2C*, and *SRF*), which might propose novel insights for tumor angiogenesis.

## Discussion

To the best of our knowledge, this is the first study to illustrate the expression pattern of CD248 in UCB and clarify the relationship between the expression of two tumor-associated vascular biomarkers (PSMA and CD248) and the clinicopathological features, prognosis, and survival of patients with UCB systematically.

The tumor vasculature is the pathological basis for the growth, invasion, and metastasis of solid tumors; therefore, vasculature-targeted diagnosis and therapy has also been proven to be an effective antitumor strategy (39). Angiogenesis inhibitors have been shown to have effective antitumor activity in a broad spectrum of cancer types (40). Although some traditional vascular markers, such as vascular endothelial growth factor (VEGF), vascular endothelial growth factor receptor 2 (VEGFR2), and delta-like 4 (DLL4), have been studied in the diagnosis and treatment of bladder cancer (41, 42), their ability to predict clinical prognosis is limited, resulting in an unvalidated therapeutic effect in UCB (43). Therefore, novel molecular markers specifically expressed in the tumor-associated vasculature, which can not only improve diagnosis and prognosis prediction, but also provide novel treatment strategies for UCB, are urgently required.

We first examined PSMA and CD248 expression, respectively, in 124 patients with UCB using IHC staining and confirmed that both PSMA and CD248 are specifically expressed in the vasculature of UCB. The expression of PSMA was associated significantly with other clinicopathological features, such as metastasis, clinical stage, invasive stage, and the Ki-67 index, while the vascular expression of CD248 was significantly associated only with the Ki-67 index. The nuclear protein Ki-67 is generally expressed only in proliferating cells (44), and the Ki-67 index is a predictive indicator of tumor growth and progression (45). In this study, the association between CD248 expression and the Ki-67 index could partially indicate a correlation with tumor metastasis and tumor invasion, although the association was not statistically significant. Furthermore, vascularly expressed PSMA and CD248 could be used as independent risk factors for UCB. Then, we further analyzed the correlation between the expression of PSMA and CD248 in UCB, and confirmed that there was a positive correlation between these two molecules, which was consistent with the results of analysis in the GEPIA database. Using CD31 as a positive control, serial section staining of UCB tissues intuitively proved the consistent expression sites of PSMA and CD248 in UCB vessels. The above results also confirmed that both PSMA and CD248 are expressed in UCB vessels and might serve as potential tumor-associated vascular biomarkers.

In addition, vascular PSMA and CD248 expression levels were associated with the prognosis of patients with UCB. Patients with high vascular PSMA or CD248 expression tended to have a shorter OS than patients with low vascular expression. The predictive accuracy of the two biomarkers (AUC_CD248_ = 0.753, AUC_PSMA_ = 0.804) were regarded as acceptable. Meanwhile, there was no significant difference between the prognostic predictive performance of vascular PSMA and CD248 expression (P = 0.2807). According to the results of multivariate analysis, taking corresponding hazard ratios as coefficients, we calculated the risk score of each patient using PSMA- and CD248-based signatures. The prognostic predictive performance of the PSMA-related risk score was significantly better than PSMA expression only (AUC_risk score_ = 0.830, AUC_PSMA_ = 0.804, P = 0.0223), while there was no significant difference between the CD248-related risk score and the corresponding expression. Therefore, combining PSMA expression with clinicopathological parameters might promote clinical practicability, while the CD248 expression level could be used independently to predict patient prognosis. To facilitate clinical application, nomograms of PSMA- and CD248-based signatures were prepared.

PSMA expression in UCB vasculature has been confirmed previously. Mary et al. (33) and Chang et al. (34) examined the PSMA expression pattern in various subtypes of bladder cancer and found that PSMA was expressed in the tumor vasculature of all the bladder cancer samples, which was consistent with the results of the present study. However, they did not further analyze the relationship between PSMA expression and patient prognosis. Gala et al. (35) examined PSMA protein expression in three normal tissues and four transitional cell carcinoma (TCC) tissues and found that both normal urothelium and TCC had positive PSMA expression; however, they did not clarify vascular PSMA expression and did not analyze the clinical significance and prognostic value of vascular PSMA expression; meanwhile, the number of samples was quite limited. Schreiber et al. (46) reported that PSMA was expressed in both the parenchyma and vessels of urothelial cell carcinoma (UCC) and that the expression was associated with tumor grade and stage, rather than tumor recurrence and recurrence-free survival. However, they did not investigate the prognostic value of PSMA in terms of OS. To offset the limitations of previous studies, the present study included a relatively higher number of cases and illustrated the vasculature expression of PSMA, and its prognostic value as a risk predictor intuitively.

To date, the expression pattern of CD248 in UCB has not been studied systemically. CD248 has been reported to be expressed by tumor vessel-associated pericytes (47) and stromal fibroblasts (48) in a wide variety of human tumors with different histologies, but not in normal vessels (27). CD248 has also been classified as a marker of tumor vessel-associated pericyte cells (49) and as a selective endothelial precursor cell marker (50). Pericytes provide structural support for endothelial cells and thus stabilize the vasculature. In our previous study, we found that CD248 was expressed specifically in HCC and RCC, and overexpression of CD248 was related to a poor prognosis (31, 32). Davies et al. (51) reported that overexpression of CD248 correlated negatively with the clinical outcome of patients with breast cancer. The above results confirmed the importance of vascular CD248 expression in tumors for predicting prognosis. In this study, using CD31 as the positive control, we evaluated and calculated the expression of CD248 in blood vessels and concluded that high vascular expression of CD248 is a significant predictive risk factor for poor prognosis in patients with UCB.

To verify the results of our research, we performed bioinformatic analyses of *CD248* and *PSMA* using a TCGA-BLCA dataset. The endothelial cell score was related positively to the expression level of *CD248* and *PSMA*, indicating that those two genes might contribute to angiogenesis, which was consistent with our aforementioned results. To explore the function of CD248 and PSMA in angiogenesis, GO enrichment analysis of Cor-DEGs was performed. The results suggested that several angiogenesis-promoting GO terms were significantly enriched, including endothelial tube morphogenesis, endothelial cell proliferation, migration and differentiation, regulation of angiogenesis, vascular permeability, and blood vessel diameter, which might provide insights into the association with angiogenesis mentioned above. To explore the underlying mechanism of Cor-DEGs in UCB angiogenesis and progression, a TFs-based regulatory network for PCor-DEGs was visualized, and several hub genes (i.e., *GATA6*, *SOX17*, *MEF2C*, and *SRF*), with maximum intramodular connectivity, were identified, which might play a vital role in PSMA/CD248-related angiogenesis regulation and in UCB progression.

Regardless of the prognostic value of PSMA and CD248, their large extracellular domains can be recognized by antibodies, peptides, RNA aptamers, and small molecules, making them ideal molecules for targeted therapy. For example, a phase I trial of MORAb-004, a humanized monoclonal antibody engineered to target CD248, was performed in multiple solid tumor types including pancreatic neuroendocrine, hepatocellular, and sarcoma (52). The specific expression of PSMA and CD248 in the vasculature of UCB will contribute to targeted therapy. In our previous studies, we confirmed a PSMA-specific single-chain antibody fragment (scFv) (termed gy1) (53) and a CD248-specific scFv named scFv78 (54) could specifically recognize the extracellular domains of PSMA and CD248, respectively. Furthermore, we reconstructed this scFv of PSMA into a human monoclonal PSMA antibody (PSMAb) and provided evidence that PSMAb could be specifically internalized into PSMA+ prostate cancer cells with high binding affinity *in vitro* and *in vivo*. In addition, we confirmed that PSMAb could inhibit tumor growth through antibody-dependent cell-mediated cytotoxicity (ADCC) and complement-dependent cytotoxicity (CDC) in PSMA+ castration-resistant prostate cancer cell xenografts *in vivo* (55). Meanwhile, we further demonstrated that an endosialin-specific antibody, IgG78, could inhibit the growth of HCC effectively in both subcutaneous and orthotopic models (31). Based on the above research, both PSMA and CD248 might represent ideal therapeutic targets for UCB.

There have been some limitations in this study. First, the sample size was relatively small, especially for advanced or metastatic cases, which might be the reason why CD248 expression didn’t show significant difference in tumor metastasis and tumor invasion. A considerable number of patients with advanced or metastatic disease no longer had indications for surgical treatment, thus their pathological specimens could not be obtained, and they could not be included in this study. Second, we mainly focused on UCB in this study and didn’t include other pathological subtypes, such as glandular and squamous carcinoma, even though UCB accounts for more than 90% bladder cancers. Notably, we should explore the prognostic value of vascular-expressed molecules in future studies for a comprehensive investigation of different pathological subtypes. Finally, the underlying mechanism of PSMA and CD248 in the progression of UCB was explored only *via* a bioinformatics-based study. Therefore, GATA6-mediated tumor angiogenesis should be explored by further laboratory investigations.

In summary, our study confirmed that PSMA and CD248 were expressed in the vasculature of UCB. Both of them were associated with deteriorated clinicopathological features and could be used as novel prognostic markers for UCB. Bioinformatic analyses further revealed the possible functions of PSMA and CD248 in angiogenesis regulation, which might partly explain the progression of UCB and provide potential diagnostic and therapeutic targets.

## Data Availability Statement

The datasets presented in this study can be found in online repositories. The names of the repository/repositories and accession number(s) can be found in the article/**Supplementary Material**.

## Ethics Statement

The study was approved by the Ethics Committee of the Xijing Hospital, Fourth Military Medical University. Informed consent was obtained from all participants included in the study. The patients/participants provided their written informed consent to participate in this study. Written informed consent was obtained from the individual(s) for the publication of any potentially identifiable images or data included in this article.

## Author Contributions

Conceptualization, YL and DH. Methodology, FY and WW. Software, DJ. Validation, CX, SL, SS, and HL. Formal analysis, KZ. Investigation, DJ. Resources, FY. Data curation, KZ. Writing—original draft preparation, YL. Writing—review and editing, BY, LY, and WQ. Visualization, KZ. Supervision, ML. Project administration, WQ. Funding acquisition, LY, WW, and WQ. All authors contributed to the article and approved the submitted version.

## Funding

This work was supported by grants from the National Natural Science Foundation of China (grant numbers 81772734, 81871379) and the Innovation Capability Support Plan of Shaanxi Province (grant numbers 2020PT-021, 2021PT-051).

## Conflict of Interest

The authors declare that the research was conducted in the absence of any commercial or financial relationships that could be construed as a potential conflict interest.

## Publisher’s Note

All claims expressed in this article are solely those of the authors and do not necessarily represent those of their affiliated organizations, or those of the publisher, the editors and the reviewers. Any product that may be evaluated in this article, or claim that may be made by its manufacturer, is not guaranteed or endorsed by the publisher.

## Supplementary Material

Supplementary Figure 1 Kaplan–Meier survival curve showing the association between *CD31* (PECAM1) expression and overall survival of patients with UCB based on the GEPIA database.
