# Supplementary figures and images for: Prognostic Value of Vascular-Expressed PSMA and CD248 in Urothelial Carcinoma of the Bladder
Source: Front Oncol. 2021 Nov 17;11:771036. doi: 10.3389/fonc.2021.771036 (PMC8635966; doi:10.3389/fonc.2021.771036)

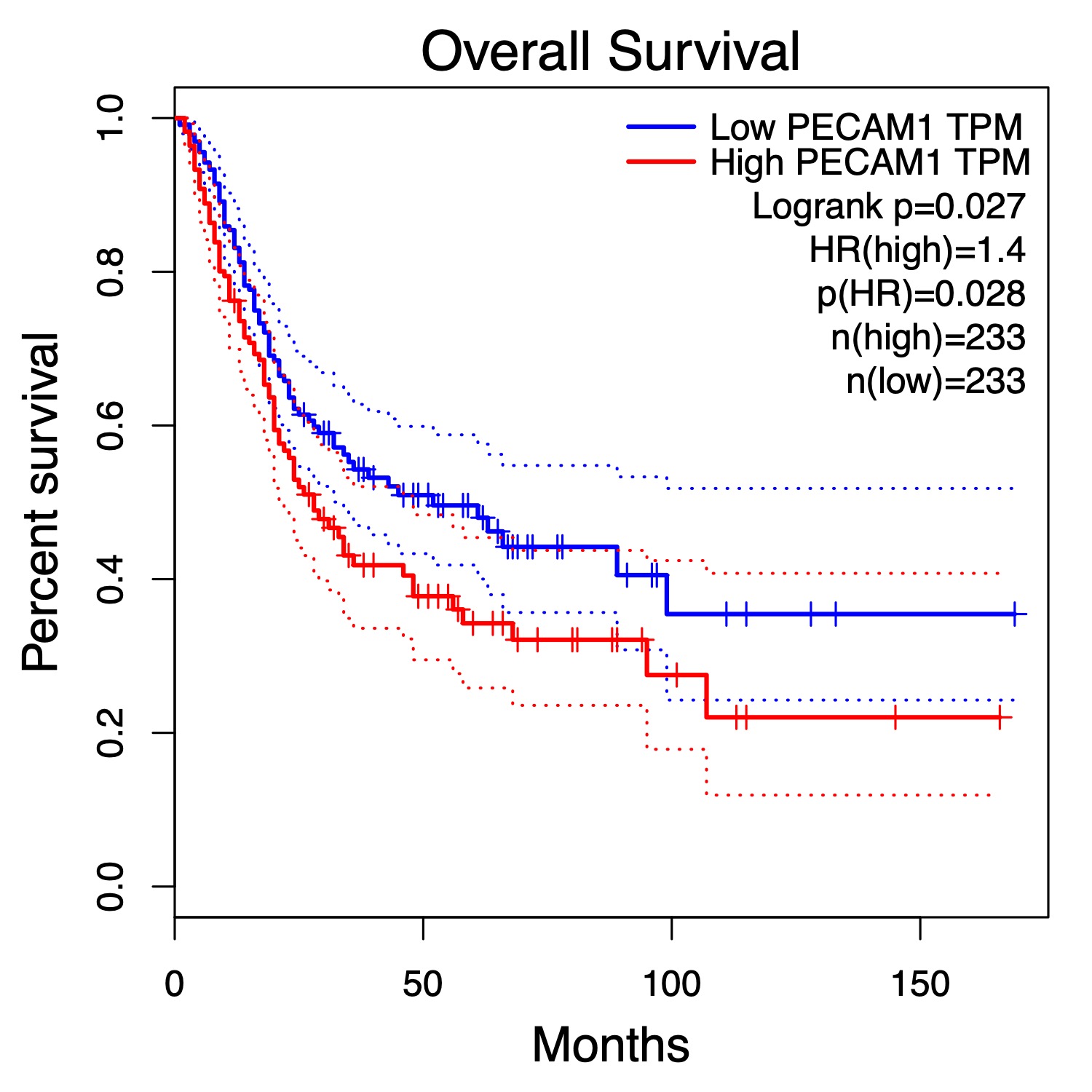

Supplement: Supplementary Figure 1 — Kaplan–Meier survival curve showing the association between CD31 (PECAM1) expression and overall survival of patients with UCB based on the GEPIA database. [file Image_1.jpeg]
