# Supplementary material for: Prognostic Value of Vascular-Expressed PSMA and CD248 in Urothelial Carcinoma of the Bladder
Source: Front Oncol. 2021 Nov 17;11:771036. doi: 10.3389/fonc.2021.771036 (PMC8635966; doi:10.3389/fonc.2021.771036)
Supplement: Supplementary file 2 [file DataSheet_1.zip › Supplementary Table 1.DOCX]

**Table S1. The association between the expression of PSMA and clinical parameters in patients with UCB (n = 124).**

| **Characteristics** | **N** | **PSMA-expression** | | **χ2** | **P value** |
| --- | --- | --- | --- | --- | --- |
|  |  | **low** | **high** |  |  |
| **Age** |  |  |  |  |  |
| < 60 | 37 | 22 | 15 | 1.888 | 0.1695 |
| ≥ 60 | 87 | 40 | 47 |  |  |
| **Sex** |  |  |  |  |  |
| Male | 104 | 54 | 50 | 0.954 | 0.3287 |
| Female | 20 | 8 | 12 |  |  |
| **Tumor differentiation** |  |  |  |  |  |
| Low grade | 84 | 38 | 46 | 2.362 | 0.1243 |
| High grade | 40 | 24 | 16 |  |  |
| **Tumor invasion** |  |  |  |  |  |
| T1–2 | 94 | 53 | 41 | 1.752 | 0.1856 |
| T3–4 | 30 | 9 | 21 |  |  |
| **Metastasis** |  |  |  |  |  |
| N0, M0 | 108 | 58 | 50 | 4.594 | 0.0321* |
| Nx, Mx | 16 | 4 | 12 |  |  |
| **Clinical stage** |  |  |  |  |  |
| Ⅰ–Ⅱ | 90 | 52 | 38 | 7.942 | 0.0048* |
| Ⅲ–Ⅳ | 34 | 10 | 24 |  |  |
| **Invasive stage** |  |  |  |  |  |
| Non-muscle | 50 | 35 | 15 | 13.410 | 0.0003* |
| Muscle | 74 | 27 | 47 |  |  |
| **Ki-67** |  |  |  |  |  |
| ≤ 15% | 40 | 29 | 11 | 11.960 | 0.0005* |
| > 15% | 84 | 33 | 51 |  |  |

*Statistically significant (P < 0.05)

**Table S2. The association between the expression of CD248 and clinical parameters in patients with UCB (n = 124).**

| **Characteristics** | **N** | **CD248-expression** | | **χ2** | **P value** |
| --- | --- | --- | --- | --- | --- |
|  |  | **low** | **high** |  |  |
| **Age** |  |  |  |  |  |
| < 60 | 37 | 15 | 22 | 0.268 | 0.6047 |
| ≥ 60 | 87 | 31 | 56 |  |  |
| **Sex** |  |  |  |  |  |
| Male | 104 | 40 | 64 | 0.515 | 0.4731 |
| Female | 20 | 6 | 14 |  |  |
| **Tumor differentiation** |  |  |  |  |  |
| Low grade | 84 | 27 | 57 | 2.739 | 0.0980 |
| High grade | 40 | 19 | 21 |  |  |
| **Tumor invasion** |  |  |  |  |  |
| T1–2 | 94 | 36 | 58 | 0.2402 | 0.6241 |
| T3–4 | 30 | 10 | 20 |  |  |
| **Metastasis** |  |  |  |  |  |
| N0M0 | 108 | 41 | 67 | 0.2691 | 0.6039 |
| NxMx | 16 | 5 | 11 |  |  |
| **Clinical stage** |  |  |  |  |  |
| Ⅰ–Ⅱ | 90 | 34 | 56 | 0.065 | 0.7984 |
| Ⅲ–Ⅳ | 34 | 12 | 22 |  |  |
| **Invasive stage** |  |  |  |  |  |
| Non-muscle | 50 | 20 | 30 | 0.3026 | 0.5822 |
| Muscle | 74 | 26 | 48 |  |  |
| **Ki-67** |  |  |  |  |  |
| ≤ 15% | 40 | 21 | 19 | 6.004 | 0.0143* |
| > 15% | 84 | 25 | 59 |  |  |

*Statistically significant (P < 0.05)
