# Supplementary material for: Prognostic Value of Vascular-Expressed PSMA and CD248 in Urothelial Carcinoma of the Bladder
Source: Front Oncol. 2021 Nov 17;11:771036. doi: 10.3389/fonc.2021.771036 (PMC8635966; doi:10.3389/fonc.2021.771036)
Supplement: Supplementary file 2 [file DataSheet_1.zip › Supporting Data 1.DOCX]

**Supporting data 1. DEGs list of TCGA-BLCA dataset**

| **Gene** | **ConMean** | **TreatMean** | **logFC** | ***P*** | **FDR** |
| --- | --- | --- | --- | --- | --- |
| PLP1 | 2.6938 | 0.1539 | -4.1292 | 0.0000 | 0.0000 |
| TK1 | 12.3386 | 55.0355 | 2.1572 | 0.0000 | 0.0000 |
| CCT5 | 22.5545 | 51.0907 | 1.1796 | 0.0000 | 0.0000 |
| CXCL12 | 22.2139 | 6.4569 | -1.7825 | 0.0000 | 0.0000 |
| RUVBL1 | 5.1789 | 11.6867 | 1.1741 | 0.0000 | 0.0000 |
| GPR19 | 0.1123 | 0.5712 | 2.3470 | 0.0000 | 0.0000 |
| DACT3 | 9.8950 | 1.3235 | -2.9023 | 0.0000 | 0.0000 |
| CENPW | 10.4780 | 26.2993 | 1.3277 | 0.0000 | 0.0000 |
| LBHD1 | 0.9437 | 1.9406 | 1.0401 | 0.0000 | 0.0001 |
| TCF3 | 9.0227 | 19.9240 | 1.1429 | 0.0000 | 0.0000 |
| BCL2 | 3.3494 | 1.2882 | -1.3785 | 0.0000 | 0.0000 |
| OCIAD2 | 13.5698 | 33.0539 | 1.2844 | 0.0000 | 0.0000 |
| FAM131C | 0.2671 | 0.7862 | 1.5577 | 0.0006 | 0.0014 |
| ZNF385C | 0.1807 | 0.5755 | 1.6711 | 0.0000 | 0.0001 |
| ENTPD6 | 8.3101 | 18.0984 | 1.1229 | 0.0000 | 0.0000 |
| COL11A1 | 0.0845 | 4.8236 | 5.8343 | 0.0000 | 0.0000 |
| AKAP12 | 25.1071 | 4.9825 | -2.3332 | 0.0000 | 0.0000 |
| MAGEA11 | 0.0436 | 1.1267 | 4.6911 | 0.0023 | 0.0049 |
| PKMYT1 | 1.4174 | 5.4179 | 1.9345 | 0.0000 | 0.0000 |
| KDELR3 | 5.1297 | 14.5991 | 1.5089 | 0.0000 | 0.0002 |
| ACP5 | 10.9319 | 24.3101 | 1.1530 | 0.0112 | 0.0193 |
| RAD54B | 0.3049 | 0.9618 | 1.6575 | 0.0000 | 0.0000 |
| CDKN3 | 2.3379 | 8.2076 | 1.8118 | 0.0000 | 0.0000 |
| AC119396.1 | 0.7409 | 0.2253 | -1.7172 | 0.0000 | 0.0000 |
| XPNPEP2 | 4.4633 | 0.3911 | -3.5126 | 0.0000 | 0.0000 |
| SFRP5 | 1.0612 | 0.1732 | -2.6151 | 0.0000 | 0.0000 |
| KIF23 | 1.3407 | 5.7495 | 2.1004 | 0.0000 | 0.0000 |
| FRY | 4.7682 | 2.2005 | -1.1156 | 0.0000 | 0.0000 |
| LYPD8 | 0.0451 | 0.3130 | 2.7950 | 0.0038 | 0.0075 |
| SAPCD1 | 0.1001 | 0.4816 | 2.2663 | 0.0000 | 0.0000 |
| MT1G | 4.3401 | 9.3963 | 1.1143 | 0.0122 | 0.0208 |
| ILK | 9.1172 | 2.6615 | -1.7763 | 0.0000 | 0.0000 |
| PITX1 | 39.1804 | 15.8993 | -1.3012 | 0.0005 | 0.0013 |
| ATXN7L2 | 0.8212 | 1.9450 | 1.2440 | 0.0000 | 0.0000 |
| FCN1 | 1.9577 | 0.6971 | -1.4897 | 0.0000 | 0.0002 |
| GPBAR1 | 2.1667 | 0.5198 | -2.0595 | 0.0000 | 0.0000 |
| GHR | 1.8465 | 0.4167 | -2.1479 | 0.0000 | 0.0000 |
| LAMTOR2 | 21.8901 | 45.6405 | 1.0600 | 0.0000 | 0.0000 |
| SKA3 | 1.0559 | 5.2453 | 2.3126 | 0.0000 | 0.0000 |
| AURKB | 3.3063 | 16.4743 | 2.3169 | 0.0000 | 0.0000 |
| DLEU7 | 0.0646 | 0.2202 | 1.7685 | 0.0000 | 0.0001 |
| NES | 18.6010 | 8.9057 | -1.0626 | 0.0000 | 0.0000 |
| STXBP6 | 2.0655 | 0.6015 | -1.7798 | 0.0000 | 0.0000 |
| PDE4B | 4.2675 | 1.1938 | -1.8378 | 0.0000 | 0.0000 |
| TMEM52 | 0.2930 | 1.5489 | 2.4022 | 0.0000 | 0.0001 |
| OXLD1 | 8.4495 | 20.5010 | 1.2788 | 0.0000 | 0.0000 |
| CDCP1 | 6.0969 | 15.0017 | 1.2990 | 0.0001 | 0.0003 |
| BOLA2B | 0.3350 | 0.9247 | 1.4646 | 0.0000 | 0.0000 |
| CSPG4 | 16.9093 | 5.9102 | -1.5165 | 0.0002 | 0.0006 |
| AR | 3.0652 | 1.4591 | -1.0709 | 0.0000 | 0.0000 |
| GFRA2 | 0.8275 | 0.2008 | -2.0433 | 0.0000 | 0.0000 |
| IGF1 | 0.5983 | 0.2612 | -1.1960 | 0.0000 | 0.0000 |
| LIMS2 | 23.4183 | 2.6994 | -3.1169 | 0.0000 | 0.0000 |
| TM7SF2 | 6.8274 | 14.1637 | 1.0528 | 0.0052 | 0.0099 |
| CXCR1 | 0.7934 | 0.2746 | -1.5307 | 0.0003 | 0.0008 |
| CMA1 | 6.7037 | 0.8556 | -2.9699 | 0.0000 | 0.0000 |
| KCNE4 | 14.3569 | 1.6295 | -3.1393 | 0.0000 | 0.0000 |
| ZBTB8B | 0.1057 | 0.2464 | 1.2214 | 0.0313 | 0.0474 |
| PRR15L | 4.0137 | 11.5645 | 1.5267 | 0.0097 | 0.0170 |
| HKDC1 | 0.3620 | 0.8223 | 1.1839 | 0.0227 | 0.0358 |
| VAV2 | 5.5147 | 12.2503 | 1.1515 | 0.0000 | 0.0001 |
| PASK | 0.8716 | 1.8466 | 1.0832 | 0.0000 | 0.0000 |
| CGREF1 | 0.3807 | 2.4172 | 2.6666 | 0.0009 | 0.0021 |
| DPP3 | 9.0322 | 21.0832 | 1.2230 | 0.0000 | 0.0000 |
| PRAG1 | 2.4608 | 5.2181 | 1.0844 | 0.0000 | 0.0001 |
| TMEM273 | 2.8015 | 1.2553 | -1.1581 | 0.0000 | 0.0000 |
| MRVI1 | 20.5796 | 3.0158 | -2.7706 | 0.0000 | 0.0000 |
| INSYN1 | 1.9227 | 0.4521 | -2.0884 | 0.0000 | 0.0000 |
| GTPBP3 | 2.5692 | 5.9855 | 1.2202 | 0.0000 | 0.0000 |
| FHL5 | 1.7906 | 0.4015 | -2.1569 | 0.0000 | 0.0000 |
| MALL | 5.9613 | 2.3148 | -1.3647 | 0.0251 | 0.0390 |
| SERPINB7 | 0.2275 | 2.3265 | 3.3539 | 0.0044 | 0.0086 |
| TMCO6 | 2.2146 | 4.5001 | 1.0229 | 0.0000 | 0.0000 |
| HIST1H1B | 0.0480 | 3.7626 | 6.2911 | 0.0000 | 0.0000 |
| KCNJ8 | 7.2736 | 2.5578 | -1.5078 | 0.0000 | 0.0001 |
| CACNA2D1 | 4.3487 | 1.0699 | -2.0231 | 0.0000 | 0.0000 |
| MYCN | 0.9926 | 3.9781 | 2.0029 | 0.0242 | 0.0379 |
| KANK3 | 2.4002 | 1.1518 | -1.0593 | 0.0000 | 0.0000 |
| PDE2A | 5.7639 | 1.0736 | -2.4246 | 0.0000 | 0.0000 |
| SLC52A2 | 13.5375 | 37.8172 | 1.4821 | 0.0000 | 0.0000 |
| HOMER3 | 7.0081 | 17.5517 | 1.3245 | 0.0000 | 0.0000 |
| PALM | 7.3568 | 3.2183 | -1.1928 | 0.0000 | 0.0000 |
| AMH | 0.1931 | 1.6585 | 3.1023 | 0.0000 | 0.0000 |
| MYH11 | 976.3795 | 30.7093 | -4.9907 | 0.0000 | 0.0000 |
| CTHRC1 | 9.7606 | 31.6084 | 1.6953 | 0.0207 | 0.0330 |
| NACAD | 1.9947 | 0.7770 | -1.3602 | 0.0000 | 0.0000 |
| ZNF675 | 0.7185 | 1.8071 | 1.3306 | 0.0000 | 0.0000 |
| ANKRD35 | 4.9725 | 2.2877 | -1.1201 | 0.0000 | 0.0000 |
| IL1RAP | 0.9817 | 2.3223 | 1.2422 | 0.0174 | 0.0283 |
| CCHCR1 | 4.1131 | 8.4803 | 1.0439 | 0.0000 | 0.0000 |
| DDN | 0.0363 | 0.2502 | 2.7857 | 0.0000 | 0.0000 |
| ARHGEF17 | 7.8386 | 3.7906 | -1.0482 | 0.0002 | 0.0007 |
| LKAAEAR1 | 0.0475 | 0.2334 | 2.2962 | 0.0007 | 0.0016 |
| GRIN3B | 0.0310 | 0.2731 | 3.1405 | 0.0000 | 0.0000 |
| SLC25A25 | 22.1516 | 4.1266 | -2.4244 | 0.0000 | 0.0000 |
| MEP1A | 0.0187 | 0.3474 | 4.2119 | 0.0299 | 0.0456 |
| ACTL8 | 0.0740 | 0.6747 | 3.1888 | 0.0010 | 0.0023 |
| WDR54 | 3.8372 | 8.4736 | 1.1429 | 0.0000 | 0.0000 |
| PLEK2 | 9.4663 | 22.1086 | 1.2237 | 0.0030 | 0.0061 |
| SH2D5 | 0.0638 | 0.5904 | 3.2094 | 0.0017 | 0.0037 |
| BEST4 | 0.2716 | 0.6097 | 1.1664 | 0.0022 | 0.0047 |
| ANKRD53 | 0.5573 | 0.2205 | -1.3380 | 0.0000 | 0.0000 |
| DISP1 | 3.1282 | 1.1815 | -1.4047 | 0.0000 | 0.0001 |
| AMPH | 0.6679 | 0.3091 | -1.1115 | 0.0000 | 0.0000 |
| CBX7 | 15.1581 | 3.2511 | -2.2211 | 0.0000 | 0.0000 |
| MGP | 206.3658 | 69.2456 | -1.5754 | 0.0000 | 0.0000 |
| MAP3K20 | 24.7598 | 3.7359 | -2.7285 | 0.0000 | 0.0000 |
| AQP1 | 110.1434 | 23.8330 | -2.2084 | 0.0000 | 0.0000 |
| PROCA1 | 0.3690 | 1.0121 | 1.4557 | 0.0000 | 0.0001 |
| MAST1 | 0.1026 | 0.5380 | 2.3906 | 0.0000 | 0.0000 |
| RAB42 | 0.6265 | 1.8287 | 1.5453 | 0.0001 | 0.0003 |
| ESR1 | 2.4269 | 0.4085 | -2.5708 | 0.0000 | 0.0000 |
| MAGEA3 | 1.7220 | 11.4232 | 2.7298 | 0.0102 | 0.0177 |
| CCDC77 | 1.5843 | 3.3631 | 1.0860 | 0.0000 | 0.0000 |
| POLR2H | 10.1553 | 23.5689 | 1.2146 | 0.0000 | 0.0000 |
| YJEFN3 | 0.8258 | 2.2950 | 1.4747 | 0.0014 | 0.0032 |
| HIST1H3I | 0.0225 | 0.8145 | 5.1792 | 0.0000 | 0.0001 |
| GAREM2 | 0.8251 | 1.8991 | 1.2026 | 0.0004 | 0.0011 |
| ATP2B4 | 43.4326 | 13.1684 | -1.7217 | 0.0000 | 0.0000 |
| THBS1 | 197.0031 | 38.1688 | -2.3678 | 0.0000 | 0.0001 |
| EMILIN1 | 75.2884 | 22.9546 | -1.7136 | 0.0000 | 0.0000 |
| KCNIP4 | 0.7829 | 0.2973 | -1.3970 | 0.0000 | 0.0000 |
| ATP11A | 1.3369 | 2.7622 | 1.0470 | 0.0006 | 0.0016 |
| ABL1 | 33.6390 | 13.2451 | -1.3447 | 0.0000 | 0.0000 |
| BUB1 | 1.7113 | 6.6737 | 1.9634 | 0.0000 | 0.0000 |
| ZNF273 | 0.5773 | 1.1952 | 1.0498 | 0.0000 | 0.0001 |
| KIF18B | 0.7415 | 6.1802 | 3.0592 | 0.0000 | 0.0000 |
| PAICS | 9.3274 | 21.0035 | 1.1711 | 0.0000 | 0.0000 |
| NAV3 | 0.6828 | 0.2282 | -1.5810 | 0.0000 | 0.0000 |
| AL136531.2 | 0.9608 | 0.2607 | -1.8818 | 0.0058 | 0.0108 |
| RGN | 2.2993 | 0.4793 | -2.2623 | 0.0000 | 0.0000 |
| TIMP2 | 86.8500 | 42.5270 | -1.0301 | 0.0000 | 0.0001 |
| TFDP1 | 15.9227 | 33.4462 | 1.0708 | 0.0000 | 0.0000 |
| TBC1D31 | 0.5664 | 1.3304 | 1.2320 | 0.0000 | 0.0000 |
| KPTN | 3.4829 | 7.9359 | 1.1881 | 0.0000 | 0.0000 |
| MCTS2P | 1.0379 | 2.1282 | 1.0360 | 0.0022 | 0.0046 |
| MRPS26 | 30.3292 | 68.0703 | 1.1663 | 0.0000 | 0.0000 |
| MAOB | 29.1017 | 3.8199 | -2.9295 | 0.0000 | 0.0000 |
| FCHO1 | 1.1450 | 3.3983 | 1.5695 | 0.0000 | 0.0000 |
| CFAP57 | 0.0684 | 0.2380 | 1.7986 | 0.0072 | 0.0131 |
| MYOM3 | 0.0409 | 0.2103 | 2.3638 | 0.0000 | 0.0001 |
| CCDC34 | 1.9959 | 4.9527 | 1.3111 | 0.0000 | 0.0000 |
| LHFPL6 | 26.8588 | 10.1413 | -1.4052 | 0.0000 | 0.0000 |
| IL6ST | 24.4684 | 9.2054 | -1.4104 | 0.0000 | 0.0000 |
| PKIG | 56.8237 | 16.0506 | -1.8239 | 0.0000 | 0.0000 |
| CPNE1 | 29.4853 | 60.3592 | 1.0336 | 0.0000 | 0.0000 |
| TP53INP2 | 37.7455 | 9.6346 | -1.9700 | 0.0000 | 0.0000 |
| SARS2 | 1.5856 | 3.4246 | 1.1109 | 0.0000 | 0.0000 |
| FAM19A3 | 0.0288 | 0.2886 | 3.3262 | 0.0000 | 0.0000 |
| TYMS | 6.0669 | 20.2403 | 1.7382 | 0.0000 | 0.0000 |
| SH3RF3 | 2.9453 | 1.3477 | -1.1279 | 0.0000 | 0.0000 |
| FASTKD3 | 1.6360 | 3.6727 | 1.1667 | 0.0000 | 0.0000 |
| CGB5 | 0.0128 | 3.7021 | 8.1804 | 0.0001 | 0.0004 |
| DMD | 6.9254 | 0.6369 | -3.4429 | 0.0000 | 0.0000 |
| TP63 | 10.5906 | 21.2218 | 1.0028 | 0.0111 | 0.0190 |
| CKM | 1.9593 | 0.5717 | -1.7770 | 0.0227 | 0.0358 |
| HIST2H2BE | 4.3889 | 18.2664 | 2.0573 | 0.0000 | 0.0000 |
| CPT1B | 0.3835 | 1.0822 | 1.4966 | 0.0000 | 0.0000 |
| SRRM5 | 0.2435 | 0.6152 | 1.3368 | 0.0000 | 0.0001 |
| RGS9 | 1.1689 | 0.3777 | -1.6297 | 0.0000 | 0.0000 |
| TMEM191C | 0.0402 | 0.2429 | 2.5966 | 0.0000 | 0.0000 |
| KLHL15 | 2.9600 | 1.4215 | -1.0582 | 0.0000 | 0.0001 |
| KRT14 | 12.2185 | 455.8566 | 5.2214 | 0.0000 | 0.0001 |
| CCDC150 | 0.0743 | 0.3932 | 2.4033 | 0.0000 | 0.0000 |
| SLC25A22 | 4.1239 | 8.9626 | 1.1199 | 0.0000 | 0.0000 |
| CSRNP1 | 73.2407 | 12.5312 | -2.5471 | 0.0000 | 0.0000 |
| UBE2T | 5.8033 | 23.0512 | 1.9899 | 0.0000 | 0.0000 |
| CCDC17 | 0.3281 | 0.6733 | 1.0372 | 0.0013 | 0.0029 |
| CCNO | 2.6506 | 6.1171 | 1.2065 | 0.0004 | 0.0010 |
| TMEM220 | 2.1120 | 0.5634 | -1.9063 | 0.0000 | 0.0000 |
| ULBP1 | 0.0424 | 0.4160 | 3.2954 | 0.0000 | 0.0000 |
| NEURL1 | 3.2757 | 1.0321 | -1.6663 | 0.0003 | 0.0007 |
| PTGS1 | 108.3385 | 6.7910 | -3.9958 | 0.0000 | 0.0000 |
| GZMM | 4.1539 | 1.7046 | -1.2850 | 0.0097 | 0.0169 |
| SLAMF8 | 1.6589 | 4.9173 | 1.5677 | 0.0140 | 0.0233 |
| HOOK1 | 1.6099 | 3.5609 | 1.1453 | 0.0001 | 0.0002 |
| KIAA0895 | 0.5018 | 1.0183 | 1.0209 | 0.0107 | 0.0184 |
| UBXN11 | 2.9442 | 6.5082 | 1.1444 | 0.0000 | 0.0000 |
| DENND2A | 5.8012 | 1.6723 | -1.7945 | 0.0000 | 0.0000 |
| NR2F1 | 11.6356 | 3.7170 | -1.6463 | 0.0000 | 0.0000 |
| SOCS3 | 218.8541 | 48.4856 | -2.1743 | 0.0000 | 0.0000 |
| DNAJB4 | 16.3774 | 4.4457 | -1.8812 | 0.0000 | 0.0000 |
| DUSP3 | 30.2446 | 13.9567 | -1.1157 | 0.0002 | 0.0006 |
| SLC5A5 | 0.0351 | 0.5370 | 3.9340 | 0.0009 | 0.0021 |
| CMPK2 | 1.4092 | 4.7686 | 1.7587 | 0.0000 | 0.0002 |
| GNG7 | 5.1238 | 0.8572 | -2.5795 | 0.0000 | 0.0000 |
| SHCBP1 | 1.3141 | 4.2489 | 1.6930 | 0.0000 | 0.0000 |
| ZNF682 | 0.7034 | 1.8743 | 1.4140 | 0.0002 | 0.0006 |
| ST6GALNAC5 | 2.1538 | 1.0003 | -1.1064 | 0.0009 | 0.0022 |
| PPP1R3B | 16.5989 | 6.2660 | -1.4055 | 0.0000 | 0.0000 |
| MRPL12 | 19.3980 | 40.8557 | 1.0746 | 0.0000 | 0.0000 |
| PALLD | 100.8551 | 14.6228 | -2.7860 | 0.0000 | 0.0000 |
| PDGFRA | 10.1090 | 2.7486 | -1.8789 | 0.0000 | 0.0000 |
| UBE2S | 7.1297 | 20.1623 | 1.4998 | 0.0000 | 0.0000 |
| P3H4 | 3.9088 | 11.9157 | 1.6081 | 0.0000 | 0.0000 |
| SERPINA1 | 2.3569 | 17.0137 | 2.8517 | 0.0011 | 0.0026 |
| MTERF3 | 7.0056 | 14.0328 | 1.0022 | 0.0000 | 0.0000 |
| MAGEA10 | 0.7518 | 3.8090 | 2.3410 | 0.0287 | 0.0441 |
| SIX3 | 0.2914 | 1.1320 | 1.9578 | 0.0274 | 0.0423 |
| CCL2 | 92.5650 | 12.4648 | -2.8926 | 0.0000 | 0.0000 |
| GTPBP2 | 9.4151 | 19.2259 | 1.0300 | 0.0000 | 0.0000 |
| COLEC12 | 7.1333 | 1.9919 | -1.8404 | 0.0000 | 0.0000 |
| CYP2A6 | 2.8805 | 0.2004 | -3.8451 | 0.0000 | 0.0000 |
| BPGM | 8.6863 | 25.9321 | 1.5779 | 0.0001 | 0.0003 |
| FOXD2 | 0.1874 | 0.5598 | 1.5789 | 0.0000 | 0.0001 |
| ESM1 | 0.2595 | 5.9719 | 4.5245 | 0.0000 | 0.0000 |
| ADAM12 | 1.2223 | 3.4588 | 1.5007 | 0.0147 | 0.0244 |
| TUBB | 155.0932 | 317.9101 | 1.0355 | 0.0000 | 0.0000 |
| CSF2 | 0.2164 | 1.5646 | 2.8543 | 0.0014 | 0.0031 |
| TPX2 | 5.6288 | 34.0751 | 2.5978 | 0.0000 | 0.0000 |
| CCDC114 | 0.0727 | 0.3304 | 2.1837 | 0.0000 | 0.0001 |
| HIST1H2BG | 0.4716 | 6.6746 | 3.8230 | 0.0000 | 0.0000 |
| CENPS | 1.3039 | 2.7465 | 1.0748 | 0.0000 | 0.0000 |
| GPER1 | 1.9997 | 0.5754 | -1.7972 | 0.0000 | 0.0000 |
| PODNL1 | 0.6791 | 2.1242 | 1.6452 | 0.0071 | 0.0130 |
| JUND | 232.2511 | 94.7068 | -1.2941 | 0.0000 | 0.0000 |
| VCX | 0.0722 | 0.8856 | 3.6167 | 0.0048 | 0.0092 |
| RBBP8NL | 1.6600 | 6.3226 | 1.9294 | 0.0000 | 0.0001 |
| AIM2 | 2.8910 | 11.6212 | 2.0071 | 0.0188 | 0.0303 |
| COLEC11 | 2.1739 | 0.9129 | -1.2518 | 0.0000 | 0.0000 |
| CRYBB3 | 0.1716 | 0.4863 | 1.5031 | 0.0047 | 0.0090 |
| SKP2 | 3.4962 | 13.6628 | 1.9664 | 0.0000 | 0.0000 |
| NKAIN4 | 0.0339 | 0.2716 | 3.0023 | 0.0003 | 0.0008 |
| IGF2BP1 | 0.0377 | 1.0531 | 4.8029 | 0.0000 | 0.0000 |
| FAM53A | 0.2244 | 0.4843 | 1.1099 | 0.0032 | 0.0065 |
| ZYG11A | 0.1142 | 0.3636 | 1.6714 | 0.0326 | 0.0493 |
| OAS1 | 12.7812 | 28.4927 | 1.1566 | 0.0010 | 0.0023 |
| FAM129A | 64.6786 | 6.1285 | -3.3997 | 0.0000 | 0.0000 |
| CALML6 | 0.1240 | 0.2971 | 1.2603 | 0.0089 | 0.0158 |
| HIST1H2BO | 0.0669 | 2.8424 | 5.4091 | 0.0000 | 0.0000 |
| ATP1B2 | 2.5334 | 0.4980 | -2.3469 | 0.0000 | 0.0000 |
| RNF128 | 13.5470 | 29.2275 | 1.1094 | 0.0161 | 0.0264 |
| FANK1 | 0.6661 | 1.3634 | 1.0334 | 0.0001 | 0.0003 |
| JUN | 257.5879 | 68.2420 | -1.9163 | 0.0000 | 0.0000 |
| MRPL36 | 10.2586 | 20.5761 | 1.0041 | 0.0000 | 0.0000 |
| GARS | 17.3696 | 35.6488 | 1.0373 | 0.0000 | 0.0000 |
| ZNF525 | 0.7402 | 2.1421 | 1.5330 | 0.0000 | 0.0000 |
| COASY | 12.8249 | 26.6201 | 1.0536 | 0.0000 | 0.0000 |
| ARL6IP1 | 40.6533 | 85.9364 | 1.0799 | 0.0000 | 0.0000 |
| SS18L1 | 2.7119 | 5.4860 | 1.0164 | 0.0000 | 0.0000 |
| NEXN | 22.9352 | 2.8687 | -2.9991 | 0.0000 | 0.0000 |
| DHTKD1 | 4.4570 | 9.7098 | 1.1234 | 0.0000 | 0.0000 |
| MFAP2 | 4.7097 | 18.9225 | 2.0064 | 0.0000 | 0.0001 |
| GRB7 | 8.4016 | 28.8907 | 1.7819 | 0.0000 | 0.0000 |
| PLAC8L1 | 0.2174 | 0.5016 | 1.2063 | 0.0028 | 0.0056 |
| RCAN2 | 24.4643 | 3.1431 | -2.9604 | 0.0000 | 0.0000 |
| AGTRAP | 12.7494 | 27.2157 | 1.0940 | 0.0000 | 0.0000 |
| CLEC10A | 4.9591 | 1.5263 | -1.7000 | 0.0000 | 0.0000 |
| CGB8 | 0.0065 | 1.1339 | 7.4375 | 0.0003 | 0.0007 |
| HAGHL | 0.4622 | 1.0560 | 1.1919 | 0.0000 | 0.0000 |
| ALOXE3 | 0.1494 | 0.5049 | 1.7563 | 0.0022 | 0.0047 |
| PANX2 | 1.6288 | 4.6146 | 1.5024 | 0.0041 | 0.0080 |
| SMTNL1 | 0.1103 | 0.5035 | 2.1903 | 0.0001 | 0.0003 |
| IGFBP5 | 130.1210 | 45.6972 | -1.5097 | 0.0000 | 0.0000 |
| MYBL2 | 6.8975 | 40.2458 | 2.5447 | 0.0000 | 0.0000 |
| PTH1R | 2.1484 | 0.4529 | -2.2459 | 0.0000 | 0.0000 |
| PPFIA4 | 0.0790 | 0.4580 | 2.5345 | 0.0000 | 0.0000 |
| CNTN4 | 0.8265 | 0.2083 | -1.9884 | 0.0000 | 0.0000 |
| MRM1 | 3.1778 | 7.8914 | 1.3122 | 0.0000 | 0.0000 |
| CDIP1 | 9.3568 | 4.3993 | -1.0887 | 0.0000 | 0.0000 |
| NEGR1 | 5.5978 | 0.3951 | -3.8245 | 0.0000 | 0.0000 |
| HTR2C | 0.0984 | 0.4583 | 2.2192 | 0.0010 | 0.0023 |
| CFH | 20.0224 | 9.3470 | -1.0990 | 0.0000 | 0.0000 |
| SNED1 | 1.7678 | 0.4985 | -1.8262 | 0.0000 | 0.0000 |
| SORBS1 | 64.4594 | 4.1257 | -3.9657 | 0.0000 | 0.0000 |
| OAZ3 | 0.2331 | 0.4886 | 1.0674 | 0.0000 | 0.0000 |
| KIAA0513 | 5.4990 | 2.0507 | -1.4231 | 0.0000 | 0.0000 |
| ENOX1 | 2.0178 | 0.9893 | -1.0283 | 0.0005 | 0.0012 |
| GAS2L3 | 0.6330 | 1.3990 | 1.1441 | 0.0000 | 0.0001 |
| PBX1 | 10.7898 | 4.7747 | -1.1762 | 0.0000 | 0.0000 |
| ABCA8 | 3.2823 | 0.3525 | -3.2191 | 0.0000 | 0.0000 |
| RTKN | 2.9610 | 9.0510 | 1.6120 | 0.0000 | 0.0000 |
| LIN28A | 0.0007 | 0.2509 | 8.5453 | 0.0008 | 0.0018 |
| SYT16 | 0.0740 | 0.2661 | 1.8470 | 0.0099 | 0.0173 |
| FOXD1 | 0.1932 | 1.5711 | 3.0233 | 0.0275 | 0.0424 |
| CHML | 1.3584 | 2.7926 | 1.0397 | 0.0005 | 0.0012 |
| SMKR1 | 0.3050 | 1.5187 | 2.3160 | 0.0000 | 0.0001 |
| NRM | 8.2217 | 16.9452 | 1.0434 | 0.0000 | 0.0000 |
| MAP3K21 | 0.4337 | 1.0623 | 1.2924 | 0.0002 | 0.0006 |
| CCDC137 | 8.7906 | 19.8832 | 1.1775 | 0.0000 | 0.0000 |
| CORO2B | 1.5152 | 0.6597 | -1.1995 | 0.0000 | 0.0000 |
| WFDC12 | 0.1630 | 0.7690 | 2.2384 | 0.0076 | 0.0137 |
| SGK1 | 29.4807 | 10.2425 | -1.5252 | 0.0000 | 0.0001 |
| PACSIN1 | 0.3264 | 0.9079 | 1.4760 | 0.0022 | 0.0045 |
| IL4I1 | 1.9354 | 4.5125 | 1.2213 | 0.0058 | 0.0108 |
| VAX2 | 0.2757 | 1.3109 | 2.2494 | 0.0018 | 0.0038 |
| GFRA3 | 3.3227 | 1.0768 | -1.6257 | 0.0000 | 0.0000 |
| CD302 | 1.9811 | 0.7642 | -1.3743 | 0.0000 | 0.0000 |
| LAMC2 | 7.3082 | 53.1758 | 2.8632 | 0.0001 | 0.0002 |
| ATF3 | 160.0250 | 21.1543 | -2.9193 | 0.0000 | 0.0000 |
| RNFT2 | 0.1930 | 0.9479 | 2.2958 | 0.0000 | 0.0000 |
| PDIA4 | 38.3543 | 108.8048 | 1.5043 | 0.0000 | 0.0000 |
| KLHL30 | 0.8273 | 0.2379 | -1.7981 | 0.0000 | 0.0000 |
| SBK1 | 0.7634 | 5.3817 | 2.8175 | 0.0000 | 0.0001 |
| AGAP2 | 0.8333 | 1.7546 | 1.0742 | 0.0031 | 0.0062 |
| DAAM2 | 3.9533 | 1.0166 | -1.9593 | 0.0000 | 0.0000 |
| C6orf136 | 5.7139 | 11.5904 | 1.0204 | 0.0000 | 0.0000 |
| CARD9 | 0.4248 | 1.5735 | 1.8889 | 0.0000 | 0.0001 |
| PIF1 | 0.5363 | 2.3095 | 2.1065 | 0.0000 | 0.0000 |
| HGH1 | 5.8838 | 16.8721 | 1.5198 | 0.0000 | 0.0000 |
| C2orf66 | 0.1142 | 0.3080 | 1.4312 | 0.0233 | 0.0366 |
| PRC1 | 3.2170 | 11.2231 | 1.8027 | 0.0000 | 0.0000 |
| RAB23 | 13.6635 | 2.2436 | -2.6064 | 0.0000 | 0.0001 |
| JPH2 | 18.4228 | 2.0221 | -3.1875 | 0.0000 | 0.0000 |
| CYB5R2 | 1.5838 | 3.3614 | 1.0857 | 0.0002 | 0.0005 |
| TAGLN2 | 213.3691 | 470.9892 | 1.1423 | 0.0000 | 0.0000 |
| CACNA1A | 0.0882 | 0.2894 | 1.7145 | 0.0305 | 0.0464 |
| SLC8A2 | 1.0264 | 0.2968 | -1.7901 | 0.0021 | 0.0044 |
| PDLIM3 | 31.8033 | 4.4032 | -2.8525 | 0.0000 | 0.0000 |
| VEGFD | 4.9818 | 0.4742 | -3.3932 | 0.0000 | 0.0000 |
| HMGA2 | 0.0200 | 1.3684 | 6.0982 | 0.0003 | 0.0007 |
| RANBP3L | 4.6466 | 0.2517 | -4.2064 | 0.0000 | 0.0000 |
| SPRN | 0.8118 | 1.9471 | 1.2621 | 0.0000 | 0.0000 |
| CYR61 | 459.5135 | 68.6112 | -2.7436 | 0.0000 | 0.0000 |
| MRPL53 | 1.6256 | 3.2811 | 1.0133 | 0.0000 | 0.0000 |
| PLPP7 | 2.2186 | 0.7657 | -1.5349 | 0.0000 | 0.0000 |
| STC1 | 26.3976 | 8.6048 | -1.6172 | 0.0008 | 0.0020 |
| KLHL41 | 4.4307 | 0.2024 | -4.4524 | 0.0000 | 0.0000 |
| SAMD10 | 3.5953 | 7.8888 | 1.1337 | 0.0002 | 0.0006 |
| HACD1 | 4.0338 | 1.4458 | -1.4803 | 0.0000 | 0.0000 |
| RAB37 | 1.7355 | 0.4876 | -1.8316 | 0.0000 | 0.0000 |
| CCDC69 | 41.1026 | 7.2432 | -2.5045 | 0.0000 | 0.0000 |
| GNGT1 | 0.1777 | 0.5566 | 1.6475 | 0.0002 | 0.0006 |
| LSM4 | 20.6703 | 42.1477 | 1.0279 | 0.0000 | 0.0000 |
| HRASLS5 | 1.1975 | 0.5046 | -1.2468 | 0.0000 | 0.0000 |
| KRT81 | 0.1382 | 22.0165 | 7.3161 | 0.0005 | 0.0012 |
| PPAT | 1.6069 | 3.4518 | 1.1031 | 0.0000 | 0.0000 |
| FAM72A | 0.1036 | 0.4947 | 2.2556 | 0.0000 | 0.0000 |
| TRAIP | 1.0841 | 3.5805 | 1.7237 | 0.0000 | 0.0000 |
| GRAP2 | 0.6925 | 0.3296 | -1.0712 | 0.0000 | 0.0001 |
| GRASP | 10.4856 | 2.9988 | -1.8060 | 0.0000 | 0.0000 |
| BOLA3 | 2.7052 | 5.9184 | 1.1295 | 0.0000 | 0.0000 |
| PLK4 | 0.8906 | 3.4880 | 1.9695 | 0.0000 | 0.0000 |
| PDE3A | 1.2177 | 0.5859 | -1.0555 | 0.0000 | 0.0000 |
| CLEC5A | 0.1612 | 0.7906 | 2.2944 | 0.0000 | 0.0001 |
| PYCR1 | 3.0554 | 21.4902 | 2.8143 | 0.0000 | 0.0000 |
| SLC45A3 | 2.0715 | 5.1582 | 1.3162 | 0.0005 | 0.0013 |
| SHISA3 | 1.6627 | 0.2400 | -2.7922 | 0.0000 | 0.0000 |
| PRSS53 | 0.1402 | 0.3422 | 1.2875 | 0.0000 | 0.0000 |
| ADA | 3.1344 | 6.7249 | 1.1013 | 0.0099 | 0.0173 |
| ACTA1 | 2.1030 | 0.5792 | -1.8602 | 0.0012 | 0.0028 |
| OXSM | 2.4742 | 4.9548 | 1.0019 | 0.0000 | 0.0000 |
| OR51E1 | 0.1432 | 0.3203 | 1.1610 | 0.0021 | 0.0044 |
| CD69 | 5.8612 | 2.5999 | -1.1727 | 0.0000 | 0.0000 |
| GGH | 5.9150 | 19.2378 | 1.7015 | 0.0000 | 0.0000 |
| PTPRD | 1.2262 | 0.3850 | -1.6715 | 0.0000 | 0.0000 |
| KCND2 | 0.2665 | 0.6515 | 1.2897 | 0.0252 | 0.0392 |
| S1PR5 | 1.3674 | 6.1800 | 2.1761 | 0.0000 | 0.0000 |
| CTSV | 0.6754 | 4.6068 | 2.7700 | 0.0000 | 0.0000 |
| NUP62CL | 0.5252 | 1.4620 | 1.4771 | 0.0001 | 0.0004 |
| PDE3B | 2.4229 | 1.1966 | -1.0178 | 0.0001 | 0.0003 |
| MPDZ | 4.1131 | 1.5485 | -1.4094 | 0.0008 | 0.0019 |
| CNIH2 | 0.3125 | 1.6663 | 2.4147 | 0.0000 | 0.0000 |
| BIN1 | 16.7546 | 4.4480 | -1.9133 | 0.0000 | 0.0000 |
| KCNK3 | 3.1431 | 0.5958 | -2.3993 | 0.0000 | 0.0000 |
| NPM3 | 19.4801 | 39.4765 | 1.0190 | 0.0000 | 0.0000 |
| HESX1 | 0.3237 | 0.7024 | 1.1176 | 0.0000 | 0.0001 |
| TNFAIP2 | 48.3742 | 121.5572 | 1.3293 | 0.0020 | 0.0043 |
| ZNF106 | 9.0377 | 3.9436 | -1.1965 | 0.0002 | 0.0007 |
| SAA4 | 0.0263 | 0.2499 | 3.2486 | 0.0190 | 0.0306 |
| ZNF485 | 0.7214 | 1.4978 | 1.0541 | 0.0000 | 0.0000 |
| LGR6 | 2.3702 | 1.0141 | -1.2249 | 0.0000 | 0.0000 |
| CYP2C9 | 0.0371 | 0.7710 | 4.3782 | 0.0121 | 0.0206 |
| TANC2 | 1.5714 | 3.7786 | 1.2658 | 0.0000 | 0.0000 |
| USP21 | 5.6244 | 12.7770 | 1.1838 | 0.0000 | 0.0000 |
| KRT12 | 0.0771 | 0.4367 | 2.5013 | 0.0126 | 0.0213 |
| CHAC2 | 1.4177 | 2.8893 | 1.0272 | 0.0000 | 0.0001 |
| ZNF841 | 1.7154 | 3.9022 | 1.1858 | 0.0001 | 0.0002 |
| AKT3 | 7.5842 | 2.5637 | -1.5648 | 0.0000 | 0.0000 |
| ZSCAN2 | 0.8108 | 1.7420 | 1.1033 | 0.0000 | 0.0000 |
| USP12 | 12.8474 | 5.5227 | -1.2180 | 0.0000 | 0.0000 |
| SMPDL3B | 1.1484 | 4.1344 | 1.8481 | 0.0000 | 0.0001 |
| GTSE1 | 0.8270 | 4.9940 | 2.5943 | 0.0000 | 0.0000 |
| PSMG3 | 10.3304 | 29.2633 | 1.5022 | 0.0000 | 0.0000 |
| NECTIN4 | 29.5899 | 70.3751 | 1.2500 | 0.0008 | 0.0020 |
| ZNF93 | 0.7978 | 2.1639 | 1.4395 | 0.0000 | 0.0000 |
| SLC10A4 | 0.1154 | 0.6609 | 2.5176 | 0.0104 | 0.0180 |
| ZSCAN16 | 2.4160 | 6.1600 | 1.3503 | 0.0000 | 0.0000 |
| TREM2 | 1.8881 | 8.0099 | 2.0848 | 0.0001 | 0.0003 |
| MLLT11 | 2.6466 | 6.9822 | 1.3995 | 0.0015 | 0.0033 |
| CCDC134 | 1.3957 | 3.2615 | 1.2246 | 0.0000 | 0.0000 |
| IQGAP3 | 1.1978 | 7.1325 | 2.5740 | 0.0000 | 0.0000 |
| RAD54L | 0.7451 | 4.1116 | 2.4642 | 0.0000 | 0.0000 |
| KRT79 | 0.0459 | 3.4870 | 6.2481 | 0.0000 | 0.0001 |
| C7 | 44.5083 | 3.4235 | -3.7005 | 0.0000 | 0.0000 |
| RNF186 | 0.0696 | 1.1318 | 4.0227 | 0.0211 | 0.0336 |
| HIPK3 | 19.5050 | 8.0411 | -1.2784 | 0.0000 | 0.0000 |
| RTL10 | 2.2624 | 5.3036 | 1.2291 | 0.0000 | 0.0000 |
| TSHZ3 | 6.2660 | 1.7924 | -1.8056 | 0.0000 | 0.0000 |
| NUSAP1 | 4.6414 | 20.1934 | 2.1212 | 0.0000 | 0.0000 |
| GADD45B | 119.8627 | 23.8533 | -2.3291 | 0.0000 | 0.0000 |
| ADGRA2 | 18.8081 | 4.2999 | -2.1290 | 0.0000 | 0.0000 |
| FAM129C | 0.7835 | 0.3306 | -1.2447 | 0.0058 | 0.0108 |
| ECSCR | 6.8016 | 2.5324 | -1.4254 | 0.0000 | 0.0000 |
| DLX3 | 0.6091 | 2.7225 | 2.1603 | 0.0195 | 0.0313 |
| LHX5 | 0.0194 | 0.4886 | 4.6570 | 0.0000 | 0.0000 |
| PSAT1 | 6.4517 | 18.1366 | 1.4911 | 0.0000 | 0.0000 |
| AMN | 0.5662 | 1.7150 | 1.5988 | 0.0009 | 0.0021 |
| NAP1L5 | 5.9790 | 2.2076 | -1.4374 | 0.0000 | 0.0000 |
| THRA | 11.1196 | 4.1514 | -1.4214 | 0.0000 | 0.0000 |
| HIST1H2AE | 0.7484 | 10.0231 | 3.7433 | 0.0000 | 0.0000 |
| SPON1 | 28.6916 | 6.2084 | -2.2083 | 0.0000 | 0.0000 |
| PPP2R3B | 1.4441 | 3.1504 | 1.1254 | 0.0000 | 0.0000 |
| CNTN1 | 13.1486 | 1.9333 | -2.7657 | 0.0000 | 0.0000 |
| SLC16A10 | 0.1373 | 0.4640 | 1.7568 | 0.0013 | 0.0029 |
| CFAP157 | 0.1948 | 0.4481 | 1.2015 | 0.0000 | 0.0000 |
| ANKRD29 | 1.6693 | 0.4779 | -1.8044 | 0.0000 | 0.0000 |
| SLC25A23 | 27.0247 | 7.3096 | -1.8864 | 0.0000 | 0.0000 |
| NBEA | 2.8948 | 0.5349 | -2.4361 | 0.0000 | 0.0000 |
| SETD7 | 8.8582 | 4.3390 | -1.0296 | 0.0001 | 0.0002 |
| TEK | 2.7376 | 1.2106 | -1.1772 | 0.0000 | 0.0000 |
| DIAPH3 | 0.7370 | 1.8183 | 1.3029 | 0.0000 | 0.0000 |
| PI15 | 2.0472 | 0.7586 | -1.4323 | 0.0002 | 0.0005 |
| TIMELESS | 3.6932 | 11.6052 | 1.6518 | 0.0000 | 0.0000 |
| TPSB2 | 23.6386 | 5.8173 | -2.0227 | 0.0000 | 0.0000 |
| SLC17A9 | 0.6956 | 1.8388 | 1.4024 | 0.0027 | 0.0056 |
| CFL2 | 20.5453 | 2.7302 | -2.9117 | 0.0000 | 0.0000 |
| SDF2L1 | 11.9522 | 30.4216 | 1.3478 | 0.0000 | 0.0000 |
| GNRH2 | 0.1405 | 0.3334 | 1.2467 | 0.0161 | 0.0264 |
| ADAMTS7 | 1.0990 | 3.0558 | 1.4754 | 0.0000 | 0.0001 |
| HIST1H2BH | 0.5958 | 8.8222 | 3.8882 | 0.0000 | 0.0000 |
| CCL21 | 31.2784 | 14.6117 | -1.0980 | 0.0002 | 0.0007 |
| CD3EAP | 1.0718 | 2.7245 | 1.3459 | 0.0000 | 0.0000 |
| RHNO1 | 7.6278 | 16.0045 | 1.0691 | 0.0000 | 0.0000 |
| NR4A3 | 22.9383 | 1.8308 | -3.6472 | 0.0000 | 0.0000 |
| P2RY14 | 3.6181 | 0.5127 | -2.8191 | 0.0000 | 0.0000 |
| ZIC5 | 0.0092 | 0.3254 | 5.1389 | 0.0002 | 0.0005 |
| GRIK5 | 1.4682 | 0.4516 | -1.7011 | 0.0000 | 0.0000 |
| GPT | 0.4610 | 1.0987 | 1.2528 | 0.0053 | 0.0099 |
| CHST6 | 0.1801 | 0.5784 | 1.6832 | 0.0142 | 0.0237 |
| SPIN4 | 1.5588 | 3.3807 | 1.1169 | 0.0033 | 0.0066 |
| CYP27B1 | 0.2804 | 1.6065 | 2.5184 | 0.0000 | 0.0000 |
| ATP8B2 | 6.5150 | 2.7853 | -1.2259 | 0.0000 | 0.0000 |
| CENPI | 0.4280 | 1.8919 | 2.1443 | 0.0000 | 0.0000 |
| SEMA3A | 2.5527 | 0.8814 | -1.5341 | 0.0000 | 0.0001 |
| MLXIP | 14.5662 | 6.7237 | -1.1153 | 0.0001 | 0.0003 |
| GPR89A | 0.4069 | 0.8970 | 1.1403 | 0.0000 | 0.0000 |
| MFSD13A | 1.1271 | 3.1212 | 1.4695 | 0.0000 | 0.0000 |
| CRISPLD2 | 30.7942 | 8.0197 | -1.9410 | 0.0000 | 0.0000 |
| ZNF296 | 2.5205 | 5.2717 | 1.0646 | 0.0004 | 0.0011 |
| ZNF697 | 0.9032 | 2.1481 | 1.2499 | 0.0000 | 0.0000 |
| MAGEC2 | 0.0607 | 2.5687 | 5.4027 | 0.0221 | 0.0350 |
| SLC16A3 | 7.9405 | 19.6276 | 1.3056 | 0.0001 | 0.0004 |
| GUCA2A | 0.1280 | 8.6922 | 6.0850 | 0.0007 | 0.0016 |
| HMBS | 3.5786 | 7.4540 | 1.0586 | 0.0000 | 0.0000 |
| SLC8A1 | 2.5519 | 0.5643 | -2.1770 | 0.0000 | 0.0000 |
| CCDC3 | 12.2333 | 5.2345 | -1.2247 | 0.0000 | 0.0000 |
| PRICKLE2 | 5.9806 | 1.4433 | -2.0509 | 0.0000 | 0.0000 |
| TIGD3 | 0.2853 | 0.7616 | 1.4168 | 0.0004 | 0.0009 |
| TMEM100 | 3.2297 | 0.4484 | -2.8484 | 0.0000 | 0.0000 |
| SLC5A6 | 4.0347 | 15.0032 | 1.8947 | 0.0000 | 0.0000 |
| AMIGO2 | 12.1577 | 31.3872 | 1.3683 | 0.0011 | 0.0026 |
| KCNK13 | 0.1699 | 1.1369 | 2.7425 | 0.0006 | 0.0015 |
| STARD10 | 10.2511 | 20.9874 | 1.0338 | 0.0003 | 0.0007 |
| E2F1 | 3.8291 | 15.3941 | 2.0073 | 0.0000 | 0.0000 |
| ABCC9 | 2.0300 | 0.4423 | -2.1984 | 0.0000 | 0.0000 |
| KCNAB1 | 0.7007 | 0.2666 | -1.3943 | 0.0000 | 0.0000 |
| HMGA1 | 67.7510 | 166.6521 | 1.2985 | 0.0000 | 0.0000 |
| ACTL6A | 8.4131 | 20.7169 | 1.3001 | 0.0000 | 0.0000 |
| MSH2 | 4.7519 | 10.5068 | 1.1447 | 0.0000 | 0.0000 |
| KCNK2 | 0.8339 | 0.1733 | -2.2664 | 0.0000 | 0.0000 |
| CELF2 | 6.7850 | 1.3353 | -2.3451 | 0.0000 | 0.0000 |
| PTTG1 | 7.6798 | 22.7815 | 1.5687 | 0.0000 | 0.0000 |
| KCNIP3 | 0.9429 | 0.3976 | -1.2457 | 0.0000 | 0.0000 |
| PSMD3 | 19.2363 | 41.0630 | 1.0940 | 0.0000 | 0.0000 |
| HIST1H2BL | 0.1054 | 1.2322 | 3.5476 | 0.0000 | 0.0001 |
| HLA-G | 1.6267 | 4.1855 | 1.3635 | 0.0126 | 0.0213 |
| NCALD | 4.7739 | 1.2034 | -1.9880 | 0.0000 | 0.0000 |
| AP1M2 | 21.6342 | 47.8640 | 1.1456 | 0.0000 | 0.0000 |
| GATA5 | 5.6844 | 0.5000 | -3.5071 | 0.0000 | 0.0000 |
| LARGE1 | 4.7963 | 2.3071 | -1.0559 | 0.0000 | 0.0001 |
| CNGB1 | 0.0295 | 0.4295 | 3.8635 | 0.0000 | 0.0000 |
| TWIST2 | 5.1498 | 1.5414 | -1.7403 | 0.0000 | 0.0000 |
| RGS2 | 132.5773 | 21.8729 | -2.5996 | 0.0000 | 0.0000 |
| MAD2L1 | 1.4240 | 5.5034 | 1.9504 | 0.0000 | 0.0000 |
| SKA1 | 1.0261 | 5.1675 | 2.3323 | 0.0000 | 0.0000 |
| FAM72C | 0.0664 | 0.4507 | 2.7637 | 0.0000 | 0.0000 |
| SH3D19 | 14.0120 | 5.7156 | -1.2937 | 0.0000 | 0.0000 |
| TDRD5 | 0.2641 | 1.2358 | 2.2261 | 0.0010 | 0.0023 |
| SAMD5 | 1.5739 | 0.7708 | -1.0300 | 0.0010 | 0.0024 |
| HABP4 | 6.2656 | 2.6685 | -1.2314 | 0.0000 | 0.0000 |
| FAM83A | 8.6053 | 21.0554 | 1.2909 | 0.0023 | 0.0048 |
| TAGLN | 743.6427 | 94.6789 | -2.9735 | 0.0000 | 0.0000 |
| ZNF556 | 0.0463 | 0.3586 | 2.9535 | 0.0000 | 0.0001 |
| CHAF1B | 1.1317 | 4.1799 | 1.8849 | 0.0000 | 0.0000 |
| TCN1 | 3.7188 | 16.9570 | 2.1890 | 0.0085 | 0.0152 |
| ANKRD44 | 1.6044 | 0.7912 | -1.0200 | 0.0000 | 0.0000 |
| SYNE4 | 1.6371 | 6.5929 | 2.0098 | 0.0000 | 0.0000 |
| TSPAN2 | 14.7531 | 3.3180 | -2.1526 | 0.0000 | 0.0000 |
| KRT75 | 0.1522 | 1.9730 | 3.6962 | 0.0240 | 0.0376 |
| TMOD1 | 8.7617 | 0.9354 | -3.2275 | 0.0000 | 0.0000 |
| TFAP4 | 1.1044 | 2.6927 | 1.2858 | 0.0000 | 0.0000 |
| NFIB | 9.0506 | 3.7895 | -1.2560 | 0.0000 | 0.0000 |
| POLE | 1.7839 | 4.0330 | 1.1768 | 0.0000 | 0.0000 |
| MEOX1 | 4.2039 | 0.9121 | -2.2044 | 0.0000 | 0.0000 |
| MCM3 | 23.2922 | 50.4448 | 1.1149 | 0.0000 | 0.0000 |
| WDR72 | 1.7269 | 5.4612 | 1.6610 | 0.0003 | 0.0008 |
| RNF207 | 1.5866 | 3.5964 | 1.1806 | 0.0001 | 0.0003 |
| PYGO1 | 1.3678 | 0.6537 | -1.0653 | 0.0001 | 0.0003 |
| LYPD1 | 0.2517 | 1.0829 | 2.1051 | 0.0002 | 0.0005 |
| C12orf75 | 2.3577 | 8.3148 | 1.8183 | 0.0018 | 0.0039 |
| KBTBD11 | 1.4371 | 0.5854 | -1.2958 | 0.0000 | 0.0000 |
| SLC2A3 | 21.9974 | 8.2693 | -1.4115 | 0.0000 | 0.0000 |
| AUNIP | 0.6995 | 3.1789 | 2.1841 | 0.0000 | 0.0000 |
| CDKN2A | 0.8594 | 15.7339 | 4.1943 | 0.0022 | 0.0047 |
| ZNF205 | 3.9563 | 8.1002 | 1.0338 | 0.0000 | 0.0000 |
| NHSL2 | 0.9861 | 0.2362 | -2.0615 | 0.0000 | 0.0000 |
| FZD5 | 1.6890 | 4.0048 | 1.2456 | 0.0005 | 0.0012 |
| UNC5C | 1.1426 | 0.3352 | -1.7691 | 0.0000 | 0.0000 |
| TSPAN11 | 2.9874 | 0.9773 | -1.6121 | 0.0000 | 0.0000 |
| SNORC | 0.2213 | 0.5629 | 1.3470 | 0.0001 | 0.0004 |
| PNPLA1 | 0.0670 | 0.4076 | 2.6049 | 0.0001 | 0.0002 |
| TRABD2B | 1.3062 | 0.5604 | -1.2209 | 0.0000 | 0.0000 |
| RPUSD3 | 3.8020 | 7.6262 | 1.0042 | 0.0000 | 0.0000 |
| NAGS | 0.3586 | 1.6951 | 2.2411 | 0.0000 | 0.0000 |
| ACVR2B | 0.6144 | 1.3618 | 1.1483 | 0.0003 | 0.0007 |
| BOC | 5.0671 | 1.1335 | -2.1604 | 0.0000 | 0.0000 |
| NKX3-2 | 0.0202 | 0.2093 | 3.3752 | 0.0000 | 0.0000 |
| RAP1A | 30.1664 | 12.9067 | -1.2248 | 0.0000 | 0.0001 |
| SH3TC2 | 0.3591 | 0.7515 | 1.0654 | 0.0014 | 0.0031 |
| PCOLCE2 | 16.1048 | 1.4264 | -3.4970 | 0.0000 | 0.0000 |
| KRTCAP2 | 4.1274 | 8.2972 | 1.0074 | 0.0000 | 0.0000 |
| SPDYE6 | 0.1252 | 0.3364 | 1.4261 | 0.0000 | 0.0000 |
| PDCD2L | 3.1412 | 6.8863 | 1.1324 | 0.0000 | 0.0000 |
| EIF4EBP1 | 23.2291 | 74.6144 | 1.6835 | 0.0000 | 0.0000 |
| MELTF | 0.7018 | 4.5527 | 2.6977 | 0.0011 | 0.0026 |
| NR3C2 | 4.6452 | 0.6431 | -2.8526 | 0.0000 | 0.0000 |
| RHBDL1 | 0.5145 | 1.2737 | 1.3079 | 0.0027 | 0.0055 |
| GALNT16 | 0.7981 | 0.3158 | -1.3376 | 0.0000 | 0.0000 |
| EVA1A | 0.1494 | 2.1224 | 3.8281 | 0.0000 | 0.0000 |
| PCGF2 | 8.0942 | 19.2764 | 1.2519 | 0.0000 | 0.0000 |
| INMT | 5.0642 | 1.2605 | -2.0063 | 0.0000 | 0.0000 |
| PABPC1L | 1.9181 | 10.4689 | 2.4483 | 0.0000 | 0.0000 |
| ARHGEF26 | 4.6864 | 1.5610 | -1.5860 | 0.0000 | 0.0000 |
| GALNT6 | 1.0030 | 4.2947 | 2.0982 | 0.0000 | 0.0000 |
| MANEAL | 2.2585 | 6.7175 | 1.5726 | 0.0001 | 0.0002 |
| IGFBP3 | 92.7835 | 265.7172 | 1.5180 | 0.0036 | 0.0072 |
| SDC3 | 23.9278 | 11.9147 | -1.0059 | 0.0002 | 0.0005 |
| INCENP | 3.1627 | 7.7832 | 1.2992 | 0.0000 | 0.0000 |
| PCSK6 | 0.3693 | 0.8428 | 1.1904 | 0.0322 | 0.0486 |
| IFIH1 | 3.1134 | 6.8143 | 1.1301 | 0.0226 | 0.0357 |
| ZDHHC12 | 11.1067 | 25.7158 | 1.2112 | 0.0000 | 0.0000 |
| TGFA | 4.5287 | 9.1477 | 1.0143 | 0.0065 | 0.0120 |
| TMTC1 | 3.1032 | 1.0232 | -1.6006 | 0.0000 | 0.0000 |
| CST1 | 0.1700 | 40.6852 | 7.9032 | 0.0000 | 0.0000 |
| GINS1 | 1.5738 | 7.1374 | 2.1811 | 0.0000 | 0.0000 |
| ANO8 | 1.8307 | 3.9085 | 1.0942 | 0.0001 | 0.0003 |
| DMRT2 | 0.0538 | 0.6020 | 3.4846 | 0.0058 | 0.0109 |
| EML1 | 6.1011 | 1.4540 | -2.0691 | 0.0000 | 0.0001 |
| EFNA2 | 0.1167 | 0.6507 | 2.4791 | 0.0232 | 0.0365 |
| RTN4R | 0.4319 | 2.2972 | 2.4112 | 0.0000 | 0.0000 |
| CTSC | 8.9373 | 23.4650 | 1.3926 | 0.0006 | 0.0015 |
| FAM241B | 3.6100 | 9.8033 | 1.4413 | 0.0000 | 0.0000 |
| PLN | 55.9697 | 4.3513 | -3.6851 | 0.0000 | 0.0000 |
| RASD1 | 20.0641 | 6.2206 | -1.6895 | 0.0000 | 0.0000 |
| NEK2 | 1.3103 | 7.2941 | 2.4768 | 0.0000 | 0.0000 |
| TAS2R5 | 0.1903 | 0.4140 | 1.1213 | 0.0252 | 0.0392 |
| ADH1B | 27.1307 | 1.4078 | -4.2684 | 0.0000 | 0.0000 |
| CDX2 | 0.0704 | 0.7411 | 3.3965 | 0.0178 | 0.0288 |
| PLEKHO1 | 20.6429 | 9.3717 | -1.1393 | 0.0000 | 0.0001 |
| TESMIN | 0.2537 | 1.1123 | 2.1322 | 0.0000 | 0.0000 |
| PTPRN | 0.0336 | 0.2789 | 3.0542 | 0.0018 | 0.0039 |
| ZFPM2 | 2.6797 | 0.5007 | -2.4201 | 0.0000 | 0.0000 |
| CDCA8 | 2.4818 | 14.1428 | 2.5106 | 0.0000 | 0.0000 |
| UNC119 | 3.9355 | 8.3865 | 1.0915 | 0.0000 | 0.0000 |
| SRPX | 39.3936 | 8.0897 | -2.2838 | 0.0000 | 0.0000 |
| CLDN9 | 0.1411 | 2.6225 | 4.2164 | 0.0001 | 0.0002 |
| PRX | 2.0906 | 0.9822 | -1.0898 | 0.0000 | 0.0000 |
| C1R | 105.3019 | 52.3921 | -1.0071 | 0.0000 | 0.0001 |
| DUSP12 | 6.0302 | 12.5134 | 1.0532 | 0.0000 | 0.0000 |
| GEM | 47.7413 | 6.3662 | -2.9067 | 0.0000 | 0.0000 |
| EZH2 | 1.9273 | 7.5238 | 1.9649 | 0.0000 | 0.0000 |
| FAT3 | 0.5974 | 0.1954 | -1.6127 | 0.0001 | 0.0003 |
| KIF26A | 1.1790 | 0.5721 | -1.0432 | 0.0000 | 0.0000 |
| CIDEC | 1.7905 | 0.5068 | -1.8209 | 0.0000 | 0.0000 |
| C3orf18 | 3.2973 | 1.3002 | -1.3425 | 0.0001 | 0.0004 |
| SSPN | 9.2495 | 2.8451 | -1.7009 | 0.0000 | 0.0000 |
| PRSS41 | 0.0252 | 0.4484 | 4.1548 | 0.0090 | 0.0160 |
| MYOCD | 11.4634 | 0.5209 | -4.4598 | 0.0000 | 0.0000 |
| TNFAIP3 | 33.0966 | 13.0565 | -1.3419 | 0.0001 | 0.0003 |
| ANKRD61 | 0.1197 | 0.3577 | 1.5792 | 0.0000 | 0.0000 |
| TINAGL1 | 24.0209 | 87.5480 | 1.8658 | 0.0000 | 0.0000 |
| WDR66 | 0.0825 | 0.7465 | 3.1782 | 0.0000 | 0.0000 |
| C9orf66 | 0.2259 | 0.4726 | 1.0647 | 0.0183 | 0.0295 |
| HIST1H4J | 0.1119 | 0.5013 | 2.1642 | 0.0002 | 0.0005 |
| SMYD2 | 4.6568 | 10.7885 | 1.2121 | 0.0000 | 0.0000 |
| SAMD4A | 4.8720 | 1.2096 | -2.0099 | 0.0000 | 0.0000 |
| PUSL1 | 4.9323 | 10.0530 | 1.0273 | 0.0000 | 0.0000 |
| CDCA3 | 1.0001 | 5.0574 | 2.3383 | 0.0000 | 0.0000 |
| RADIL | 0.5180 | 0.2257 | -1.1987 | 0.0000 | 0.0000 |
| NPR2 | 4.6610 | 1.6844 | -1.4684 | 0.0000 | 0.0000 |
| SLC2A4 | 13.3860 | 0.6082 | -4.4601 | 0.0000 | 0.0000 |
| GRHL2 | 7.2730 | 15.0435 | 1.0485 | 0.0001 | 0.0003 |
| PRIMA1 | 3.0741 | 0.4086 | -2.9116 | 0.0000 | 0.0000 |
| NR4A1 | 151.4262 | 12.1114 | -3.6442 | 0.0000 | 0.0000 |
| PTK7 | 7.7657 | 16.6404 | 1.0995 | 0.0001 | 0.0003 |
| LRGUK | 0.0592 | 0.2439 | 2.0425 | 0.0000 | 0.0001 |
| FSD1 | 0.2450 | 1.8127 | 2.8872 | 0.0001 | 0.0003 |
| STON1 | 6.3336 | 1.3196 | -2.2629 | 0.0000 | 0.0000 |
| C1orf35 | 3.2708 | 7.3987 | 1.1776 | 0.0000 | 0.0000 |
| CACYBP | 10.0152 | 21.0712 | 1.0731 | 0.0000 | 0.0000 |
| EBF1 | 4.2858 | 0.8909 | -2.2663 | 0.0000 | 0.0000 |
| RSAD2 | 1.3203 | 6.5169 | 2.3033 | 0.0024 | 0.0050 |
| TPSG1 | 1.2088 | 0.1587 | -2.9294 | 0.0000 | 0.0000 |
| EN2 | 0.0186 | 0.3828 | 4.3653 | 0.0001 | 0.0003 |
| GPR135 | 0.7464 | 0.2740 | -1.4457 | 0.0001 | 0.0003 |
| MAMDC2 | 11.6171 | 1.2019 | -3.2728 | 0.0000 | 0.0000 |
| TSLP | 1.7592 | 0.4363 | -2.0116 | 0.0000 | 0.0000 |
| KRTDAP | 16.2956 | 48.4458 | 1.5719 | 0.0320 | 0.0484 |
| TLE6 | 0.4106 | 1.9175 | 2.2233 | 0.0005 | 0.0013 |
| TCF19 | 4.4386 | 14.4641 | 1.7043 | 0.0000 | 0.0000 |
| KMT5C | 1.6226 | 3.6426 | 1.1667 | 0.0000 | 0.0000 |
| PLA2G5 | 5.0173 | 0.7067 | -2.8278 | 0.0000 | 0.0001 |
| IGSF9 | 1.9434 | 9.4043 | 2.2748 | 0.0000 | 0.0000 |
| TEX45 | 0.2044 | 0.9532 | 2.2216 | 0.0072 | 0.0130 |
| VSTM4 | 5.7763 | 1.1733 | -2.2995 | 0.0000 | 0.0000 |
| ARID5A | 26.9496 | 7.8167 | -1.7856 | 0.0000 | 0.0000 |
| HSPB7 | 29.8995 | 2.7514 | -3.4419 | 0.0000 | 0.0000 |
| HEPH | 7.0685 | 2.0962 | -1.7536 | 0.0000 | 0.0000 |
| IQANK1 | 7.6586 | 19.2820 | 1.3321 | 0.0000 | 0.0001 |
| CHRNA5 | 0.6493 | 1.6320 | 1.3298 | 0.0000 | 0.0000 |
| GFRA1 | 2.6353 | 0.3199 | -3.0420 | 0.0000 | 0.0000 |
| PODXL2 | 5.6409 | 25.6854 | 2.1869 | 0.0000 | 0.0000 |
| SUCNR1 | 0.1075 | 0.4779 | 2.1524 | 0.0128 | 0.0217 |
| TLX1 | 0.0778 | 0.4385 | 2.4948 | 0.0000 | 0.0001 |
| CEP72 | 0.9968 | 2.2584 | 1.1799 | 0.0000 | 0.0000 |
| CCDC14 | 1.7099 | 4.7078 | 1.4611 | 0.0000 | 0.0000 |
| CASP2 | 3.0089 | 7.6683 | 1.3497 | 0.0000 | 0.0000 |
| RFX2 | 3.6811 | 1.5713 | -1.2282 | 0.0000 | 0.0000 |
| RPN1 | 53.3751 | 109.0309 | 1.0305 | 0.0000 | 0.0000 |
| STRA6 | 0.7881 | 2.3003 | 1.5454 | 0.0034 | 0.0068 |
| CBX3 | 23.2061 | 49.7704 | 1.1008 | 0.0000 | 0.0000 |
| RASSF3 | 38.6381 | 9.6245 | -2.0052 | 0.0001 | 0.0003 |
| SERPINB11 | 1.8595 | 0.3349 | -2.4729 | 0.0069 | 0.0127 |
| NAT14 | 4.6829 | 11.3423 | 1.2763 | 0.0000 | 0.0000 |
| CASP14 | 1.3697 | 38.5383 | 4.8144 | 0.0000 | 0.0000 |
| MYH3 | 5.3080 | 0.8084 | -2.7150 | 0.0005 | 0.0013 |
| CRTAC1 | 50.1990 | 16.1395 | -1.6371 | 0.0004 | 0.0009 |
| PTPRR | 0.6640 | 3.8658 | 2.5416 | 0.0144 | 0.0240 |
| TRIM46 | 0.3113 | 0.7188 | 1.2072 | 0.0021 | 0.0045 |
| PRKAA2 | 1.8338 | 0.3654 | -2.3273 | 0.0000 | 0.0000 |
| STAT1 | 26.6203 | 53.4255 | 1.0050 | 0.0112 | 0.0192 |
| ANKRD13B | 2.0894 | 4.4290 | 1.0839 | 0.0014 | 0.0032 |
| ZBTB47 | 12.0562 | 5.2016 | -1.2127 | 0.0000 | 0.0000 |
| NGB | 0.0209 | 0.4593 | 4.4595 | 0.0043 | 0.0083 |
| STK31 | 0.0952 | 0.4728 | 2.3122 | 0.0000 | 0.0000 |
| RYR2 | 1.1809 | 0.1600 | -2.8837 | 0.0000 | 0.0000 |
| BCHE | 6.9981 | 0.8431 | -3.0531 | 0.0000 | 0.0000 |
| PRSS16 | 1.1688 | 3.1598 | 1.4348 | 0.0000 | 0.0000 |
| DLX5 | 1.6040 | 4.9533 | 1.6267 | 0.0102 | 0.0177 |
| SLC52A3 | 3.7887 | 7.6836 | 1.0201 | 0.0031 | 0.0063 |
| PKD1 | 6.8792 | 2.8543 | -1.2691 | 0.0000 | 0.0000 |
| SYNC | 2.5249 | 0.5594 | -2.1742 | 0.0000 | 0.0001 |
| POPDC2 | 26.6200 | 2.0715 | -3.6838 | 0.0000 | 0.0000 |
| SGCD | 3.8585 | 0.5100 | -2.9195 | 0.0000 | 0.0000 |
| FANCD2 | 1.1283 | 4.0310 | 1.8370 | 0.0000 | 0.0000 |
| SFRP1 | 20.7511 | 3.2464 | -2.6763 | 0.0000 | 0.0000 |
| SMIM24 | 0.1416 | 1.0401 | 2.8770 | 0.0002 | 0.0005 |
| FCGR3A | 4.5946 | 19.6094 | 2.0935 | 0.0081 | 0.0145 |
| TMEM246 | 4.6587 | 2.0410 | -1.1907 | 0.0000 | 0.0000 |
| IL6R | 4.0857 | 1.9400 | -1.0745 | 0.0000 | 0.0001 |
| ULBP2 | 0.9135 | 5.6975 | 2.6408 | 0.0000 | 0.0000 |
| PYCR3 | 3.4425 | 8.3796 | 1.2834 | 0.0000 | 0.0000 |
| CNTD1 | 0.2380 | 0.5322 | 1.1611 | 0.0000 | 0.0000 |
| FIGNL1 | 1.3311 | 3.4617 | 1.3789 | 0.0000 | 0.0000 |
| ST8SIA1 | 0.7869 | 0.2372 | -1.7304 | 0.0000 | 0.0000 |
| MEIS2 | 5.0196 | 1.7162 | -1.5483 | 0.0000 | 0.0000 |
| ZNF724 | 0.1212 | 0.5574 | 2.2014 | 0.0000 | 0.0000 |
| RHOD | 15.9152 | 36.6732 | 1.2043 | 0.0031 | 0.0062 |
| GLI2 | 1.0289 | 0.4611 | -1.1580 | 0.0000 | 0.0000 |
| FZD7 | 21.8469 | 6.3884 | -1.7739 | 0.0000 | 0.0000 |
| LIPK | 0.0740 | 0.5653 | 2.9325 | 0.0004 | 0.0011 |
| MTFR2 | 0.6749 | 3.0398 | 2.1712 | 0.0000 | 0.0000 |
| TSPAN18 | 11.7507 | 2.3453 | -2.3249 | 0.0000 | 0.0000 |
| SIRT7 | 3.8607 | 8.1451 | 1.0771 | 0.0000 | 0.0000 |
| MPZ | 4.3229 | 1.0152 | -2.0902 | 0.0000 | 0.0000 |
| PHACTR1 | 0.9559 | 0.4551 | -1.0707 | 0.0000 | 0.0000 |
| WDR90 | 1.4834 | 3.9772 | 1.4228 | 0.0000 | 0.0000 |
| CSRP1 | 217.6064 | 26.2443 | -3.0516 | 0.0000 | 0.0000 |
| CHAD | 0.3831 | 0.9892 | 1.3685 | 0.0196 | 0.0315 |
| LGALS2 | 4.1191 | 1.9936 | -1.0470 | 0.0034 | 0.0067 |
| EPB41L2 | 7.0224 | 3.1624 | -1.1509 | 0.0000 | 0.0000 |
| GLP2R | 4.2645 | 0.1486 | -4.8429 | 0.0000 | 0.0000 |
| CACNB2 | 3.0994 | 0.4610 | -2.7491 | 0.0000 | 0.0000 |
| CILP2 | 0.8976 | 2.4358 | 1.4402 | 0.0204 | 0.0326 |
| CAVIN2 | 43.0681 | 3.7120 | -3.5364 | 0.0000 | 0.0000 |
| HMOX1 | 13.4398 | 31.3818 | 1.2234 | 0.0222 | 0.0351 |
| TEAD1 | 18.0913 | 5.7635 | -1.6503 | 0.0000 | 0.0001 |
| ZHX3 | 4.4277 | 1.9608 | -1.1751 | 0.0000 | 0.0000 |
| OPLAH | 2.4655 | 6.0165 | 1.2871 | 0.0001 | 0.0002 |
| RAB30 | 1.9419 | 0.8303 | -1.2258 | 0.0000 | 0.0001 |
| B4GALT3 | 9.8693 | 24.0811 | 1.2869 | 0.0000 | 0.0000 |
| ECEL1 | 0.1032 | 0.8075 | 2.9677 | 0.0135 | 0.0227 |
| TUBA3E | 0.0440 | 0.6253 | 3.8283 | 0.0080 | 0.0144 |
| IL17RB | 0.7903 | 2.1098 | 1.4167 | 0.0017 | 0.0036 |
| FLNA | 792.9072 | 102.7258 | -2.9484 | 0.0000 | 0.0000 |
| ZNF730 | 0.0404 | 0.2628 | 2.6999 | 0.0024 | 0.0051 |
| RBMS3 | 2.8231 | 0.7291 | -1.9530 | 0.0000 | 0.0000 |
| MFAP5 | 17.1103 | 3.5165 | -2.2827 | 0.0000 | 0.0000 |
| PSG5 | 0.0020 | 0.5039 | 7.9968 | 0.0001 | 0.0002 |
| FOXL2 | 0.0752 | 0.8252 | 3.4556 | 0.0000 | 0.0001 |
| RDH16 | 0.2012 | 0.9369 | 2.2191 | 0.0001 | 0.0003 |
| ASF1B | 3.7594 | 17.7316 | 2.2377 | 0.0000 | 0.0000 |
| RAD18 | 1.2513 | 2.9406 | 1.2327 | 0.0000 | 0.0000 |
| CENPO | 1.0477 | 3.1222 | 1.5753 | 0.0000 | 0.0000 |
| COMTD1 | 6.7211 | 15.6187 | 1.2165 | 0.0000 | 0.0000 |
| PRSS35 | 0.7710 | 0.2249 | -1.7775 | 0.0000 | 0.0000 |
| ARHGAP10 | 6.6961 | 2.8452 | -1.2348 | 0.0000 | 0.0000 |
| PCK2 | 6.7841 | 16.2365 | 1.2590 | 0.0000 | 0.0000 |
| ARHGEF19 | 6.2785 | 13.3335 | 1.0866 | 0.0003 | 0.0009 |
| LLGL2 | 7.6281 | 16.3399 | 1.0990 | 0.0001 | 0.0003 |
| POU6F1 | 2.0133 | 0.9075 | -1.1497 | 0.0000 | 0.0000 |
| MIOX | 0.0252 | 0.3297 | 3.7093 | 0.0000 | 0.0000 |
| ZDHHC23 | 0.5710 | 1.6797 | 1.5566 | 0.0000 | 0.0000 |
| TSTD1 | 23.9355 | 52.5826 | 1.1354 | 0.0001 | 0.0003 |
| TMEM270 | 0.3063 | 0.6256 | 1.0305 | 0.0041 | 0.0081 |
| EPHB1 | 1.5354 | 0.2853 | -2.4280 | 0.0001 | 0.0004 |
| CDC45 | 1.6496 | 8.2013 | 2.3137 | 0.0000 | 0.0000 |
| SUV39H1 | 2.4885 | 5.2414 | 1.0747 | 0.0000 | 0.0000 |
| BNC2 | 2.0948 | 0.4658 | -2.1691 | 0.0000 | 0.0000 |
| MATN3 | 0.3121 | 1.5505 | 2.3125 | 0.0000 | 0.0000 |
| PPARGC1B | 1.8532 | 0.8490 | -1.1261 | 0.0000 | 0.0000 |
| MYOM2 | 1.0396 | 0.1829 | -2.5067 | 0.0000 | 0.0000 |
| KCNQ4 | 2.3572 | 0.4923 | -2.2594 | 0.0000 | 0.0000 |
| TUBA1A | 91.4231 | 40.1857 | -1.1859 | 0.0000 | 0.0000 |
| PTPRQ | 0.8225 | 0.3882 | -1.0832 | 0.0001 | 0.0002 |
| PER2 | 9.8434 | 2.4851 | -1.9859 | 0.0000 | 0.0000 |
| IRF5 | 3.2458 | 9.9357 | 1.6140 | 0.0000 | 0.0000 |
| TSPAN7 | 12.7006 | 5.1979 | -1.2889 | 0.0000 | 0.0000 |
| GPM6B | 2.8411 | 1.0042 | -1.5004 | 0.0000 | 0.0000 |
| MYLPF | 0.2865 | 1.1559 | 2.0123 | 0.0020 | 0.0042 |
| SLC25A1 | 29.4086 | 61.9628 | 1.0752 | 0.0000 | 0.0000 |
| LBX2 | 0.1541 | 0.8414 | 2.4486 | 0.0000 | 0.0000 |
| IFIT2 | 2.0712 | 6.3533 | 1.6170 | 0.0151 | 0.0250 |
| PDLIM7 | 52.0123 | 19.0958 | -1.4456 | 0.0004 | 0.0010 |
| PCP2 | 0.4788 | 1.9710 | 2.0414 | 0.0001 | 0.0003 |
| SPSB2 | 2.7952 | 5.9453 | 1.0888 | 0.0000 | 0.0000 |
| CGAS | 1.5777 | 3.7255 | 1.2396 | 0.0006 | 0.0016 |
| MS4A14 | 0.1726 | 0.4458 | 1.3694 | 0.0136 | 0.0228 |
| C5orf34 | 0.7797 | 1.7753 | 1.1871 | 0.0000 | 0.0000 |
| PFDN2 | 56.6275 | 137.5105 | 1.2800 | 0.0000 | 0.0000 |
| MPV17L | 1.6672 | 0.7809 | -1.0943 | 0.0000 | 0.0000 |
| SPNS1 | 0.4123 | 0.8764 | 1.0880 | 0.0000 | 0.0000 |
| FAM13B | 8.1367 | 3.7387 | -1.1219 | 0.0001 | 0.0004 |
| CCDC68 | 0.4569 | 1.0602 | 1.2142 | 0.0135 | 0.0227 |
| ST20 | 0.8391 | 1.8134 | 1.1119 | 0.0000 | 0.0000 |
| CABP7 | 0.1498 | 0.5089 | 1.7640 | 0.0007 | 0.0016 |
| CEP85 | 1.7595 | 4.4572 | 1.3410 | 0.0000 | 0.0000 |
| CAMK2N2 | 0.2002 | 1.3400 | 2.7424 | 0.0000 | 0.0000 |
| NDC1 | 4.4050 | 10.1472 | 1.2038 | 0.0000 | 0.0000 |
| NLN | 1.2429 | 2.7137 | 1.1266 | 0.0000 | 0.0000 |
| WDR97 | 0.0922 | 0.2716 | 1.5587 | 0.0000 | 0.0000 |
| BCL2L2-PABPN1 | 0.1667 | 0.4292 | 1.3642 | 0.0000 | 0.0000 |
| CHEK2 | 2.3768 | 4.8599 | 1.0319 | 0.0000 | 0.0000 |
| PLPP3 | 40.8549 | 9.2898 | -2.1368 | 0.0000 | 0.0000 |
| ZDBF2 | 1.0114 | 0.4977 | -1.0229 | 0.0000 | 0.0000 |
| LAMA4 | 14.7250 | 5.8627 | -1.3286 | 0.0011 | 0.0026 |
| ZNF813 | 0.8457 | 2.0205 | 1.2564 | 0.0000 | 0.0000 |
| 44440.0000 | 4.9084 | 2.3807 | -1.0439 | 0.0189 | 0.0305 |
| NDUFA4L2 | 13.2129 | 34.4619 | 1.3830 | 0.0115 | 0.0198 |
| CDC25A | 1.0778 | 3.8167 | 1.8243 | 0.0000 | 0.0000 |
| APOC1 | 6.3936 | 37.3014 | 2.5445 | 0.0000 | 0.0000 |
| PIP4P2 | 5.2362 | 2.4287 | -1.1083 | 0.0006 | 0.0014 |
| GLDC | 1.4445 | 4.0586 | 1.4904 | 0.0059 | 0.0110 |
| BHMT2 | 5.1575 | 0.3845 | -3.7457 | 0.0000 | 0.0000 |
| C1orf21 | 10.8178 | 4.9884 | -1.1168 | 0.0000 | 0.0000 |
| GPRASP1 | 6.0378 | 0.8866 | -2.7676 | 0.0000 | 0.0000 |
| F12 | 1.3647 | 2.9010 | 1.0880 | 0.0006 | 0.0015 |
| DCLK1 | 1.5144 | 0.3531 | -2.1008 | 0.0000 | 0.0000 |
| GABRA3 | 0.6456 | 1.9381 | 1.5859 | 0.0040 | 0.0078 |
| ADAMTS16 | 1.5787 | 0.6221 | -1.3434 | 0.0287 | 0.0440 |
| ITGA7 | 20.2385 | 3.4503 | -2.5523 | 0.0000 | 0.0000 |
| SMCO2 | 0.0865 | 0.3977 | 2.2005 | 0.0000 | 0.0000 |
| KIAA1614 | 0.9382 | 0.3601 | -1.3815 | 0.0000 | 0.0001 |
| IGSF8 | 15.3596 | 35.3065 | 1.2008 | 0.0000 | 0.0000 |
| TONSL | 1.6575 | 5.9385 | 1.8411 | 0.0000 | 0.0000 |
| SLIT3 | 18.5858 | 2.4095 | -2.9474 | 0.0000 | 0.0000 |
| NLRP7 | 0.0607 | 4.8914 | 6.3326 | 0.0002 | 0.0005 |
| FKBP5 | 11.5980 | 5.3839 | -1.1072 | 0.0005 | 0.0012 |
| ABHD12 | 21.1485 | 45.2633 | 1.0978 | 0.0000 | 0.0000 |
| RAB9B | 5.4682 | 0.5960 | -3.1978 | 0.0000 | 0.0000 |
| CCT2 | 27.9289 | 63.0973 | 1.1758 | 0.0000 | 0.0000 |
| MRGBP | 5.4637 | 12.1688 | 1.1552 | 0.0000 | 0.0000 |
| RHBG | 0.4454 | 4.1657 | 3.2255 | 0.0008 | 0.0019 |
| C19orf48 | 10.0008 | 29.1504 | 1.5434 | 0.0000 | 0.0000 |
| CCSAP | 1.1558 | 3.0598 | 1.4045 | 0.0000 | 0.0000 |
| ZNF572 | 0.4173 | 0.9175 | 1.1367 | 0.0001 | 0.0004 |
| AL159163.1 | 0.2761 | 0.8284 | 1.5850 | 0.0000 | 0.0002 |
| GJB6 | 19.3494 | 50.2142 | 1.3758 | 0.0007 | 0.0018 |
| CLK2 | 7.8780 | 17.0466 | 1.1136 | 0.0000 | 0.0000 |
| TGFB1I1 | 25.6236 | 6.3967 | -2.0021 | 0.0000 | 0.0000 |
| ANLN | 5.0148 | 11.6257 | 1.2131 | 0.0000 | 0.0000 |
| SLC15A1 | 0.5910 | 3.7448 | 2.6636 | 0.0004 | 0.0011 |
| ESPN | 1.4990 | 4.5268 | 1.5945 | 0.0003 | 0.0009 |
| ADAM10 | 7.9148 | 16.4124 | 1.0522 | 0.0000 | 0.0001 |
| PPM1L | 6.8596 | 1.9920 | -1.7839 | 0.0000 | 0.0000 |
| DDIT4 | 50.9248 | 110.8479 | 1.1221 | 0.0245 | 0.0382 |
| RPA3 | 4.3961 | 9.6844 | 1.1394 | 0.0000 | 0.0000 |
| ZNF419 | 0.5617 | 1.1297 | 1.0082 | 0.0000 | 0.0000 |
| DEPDC1 | 0.7031 | 3.1085 | 2.1445 | 0.0000 | 0.0000 |
| MEX3D | 6.5158 | 13.3929 | 1.0395 | 0.0000 | 0.0000 |
| RNF122 | 18.8079 | 5.3581 | -1.8115 | 0.0000 | 0.0000 |
| ADAT3 | 0.7754 | 1.7137 | 1.1441 | 0.0000 | 0.0001 |
| MRPL14 | 33.9929 | 71.5364 | 1.0734 | 0.0000 | 0.0000 |
| SLC6A6 | 4.0176 | 9.0948 | 1.1787 | 0.0000 | 0.0001 |
| SDC2 | 29.7522 | 10.1954 | -1.5451 | 0.0000 | 0.0000 |
| RFC3 | 2.6766 | 6.9640 | 1.3795 | 0.0000 | 0.0000 |
| KAT2A | 11.6456 | 28.2280 | 1.2773 | 0.0000 | 0.0000 |
| PPP1R16A | 2.6185 | 6.5727 | 1.3278 | 0.0000 | 0.0000 |
| ABCB1 | 1.5690 | 0.4474 | -1.8101 | 0.0000 | 0.0000 |
| CAPG | 60.6820 | 128.8153 | 1.0860 | 0.0002 | 0.0006 |
| CLEC3B | 53.1329 | 2.6517 | -4.3246 | 0.0000 | 0.0000 |
| KCTD12 | 22.8151 | 10.0221 | -1.1868 | 0.0000 | 0.0000 |
| ABLIM1 | 27.4704 | 10.1134 | -1.4416 | 0.0000 | 0.0000 |
| WWTR1 | 22.0261 | 6.3980 | -1.7835 | 0.0000 | 0.0000 |
| S100A7 | 46.7744 | 277.8789 | 2.5707 | 0.0071 | 0.0129 |
| DCUN1D5 | 10.3780 | 23.1606 | 1.1581 | 0.0000 | 0.0000 |
| TUBB3 | 0.2840 | 1.6441 | 2.5331 | 0.0000 | 0.0000 |
| MT1H | 0.2049 | 2.6551 | 3.6956 | 0.0001 | 0.0002 |
| MAP3K8 | 13.7280 | 6.0730 | -1.1767 | 0.0000 | 0.0001 |
| SLC5A3 | 2.0017 | 4.2838 | 1.0977 | 0.0001 | 0.0003 |
| FOSL1 | 80.0768 | 22.2569 | -1.8471 | 0.0024 | 0.0051 |
| STIL | 0.4834 | 2.5185 | 2.3814 | 0.0000 | 0.0000 |
| DEPDC1B | 0.6599 | 3.3976 | 2.3643 | 0.0000 | 0.0000 |
| MORN5 | 2.7657 | 0.1214 | -4.5094 | 0.0000 | 0.0000 |
| NAT9 | 5.0161 | 10.9250 | 1.1230 | 0.0000 | 0.0000 |
| KLC3 | 0.5994 | 3.0902 | 2.3660 | 0.0000 | 0.0000 |
| IER3 | 297.8179 | 60.7991 | -2.2923 | 0.0000 | 0.0001 |
| MCIDAS | 0.2012 | 0.8660 | 2.1059 | 0.0000 | 0.0000 |
| NEURL1B | 11.6727 | 4.9200 | -1.2464 | 0.0001 | 0.0002 |
| PLEKHG4 | 0.6464 | 2.3794 | 1.8800 | 0.0114 | 0.0195 |
| UNG | 12.9230 | 27.5801 | 1.0937 | 0.0000 | 0.0000 |
| HENMT1 | 3.9792 | 8.7144 | 1.1309 | 0.0000 | 0.0000 |
| HPDL | 0.5242 | 2.3283 | 2.1512 | 0.0085 | 0.0152 |
| PAM16 | 2.2793 | 4.5737 | 1.0048 | 0.0000 | 0.0000 |
| ZGLP1 | 0.3122 | 0.7846 | 1.3293 | 0.0000 | 0.0001 |
| NFIC | 19.7054 | 8.9237 | -1.1429 | 0.0000 | 0.0000 |
| ROS1 | 0.0045 | 0.3975 | 6.4562 | 0.0007 | 0.0018 |
| PDIA2 | 0.0625 | 0.5204 | 3.0576 | 0.0001 | 0.0004 |
| ARNTL2 | 2.9313 | 7.3153 | 1.3193 | 0.0004 | 0.0011 |
| CENPE | 0.6850 | 2.8795 | 2.0717 | 0.0000 | 0.0000 |
| CLBA1 | 1.4235 | 2.8516 | 1.0023 | 0.0000 | 0.0000 |
| LIME1 | 0.6063 | 1.8737 | 1.6279 | 0.0000 | 0.0000 |
| PODN | 27.2646 | 5.9080 | -2.2063 | 0.0000 | 0.0000 |
| C18orf54 | 0.5771 | 1.4511 | 1.3302 | 0.0000 | 0.0000 |
| TGFBR3L | 0.1241 | 0.3956 | 1.6719 | 0.0331 | 0.0499 |
| APLN | 0.5676 | 3.0802 | 2.4401 | 0.0000 | 0.0000 |
| ATP6V0D2 | 0.0323 | 0.3047 | 3.2396 | 0.0000 | 0.0001 |
| NPAS4 | 0.6998 | 0.2739 | -1.3534 | 0.0000 | 0.0000 |
| GATA6 | 9.2907 | 1.5442 | -2.5889 | 0.0000 | 0.0000 |
| CXCL9 | 4.4038 | 24.2374 | 2.4604 | 0.0256 | 0.0397 |
| MANF | 22.7370 | 48.6702 | 1.0980 | 0.0000 | 0.0000 |
| PDLIM4 | 13.9041 | 6.9252 | -1.0056 | 0.0000 | 0.0000 |
| NPR1 | 5.3286 | 1.6402 | -1.6999 | 0.0000 | 0.0000 |
| SYCE3 | 0.3408 | 0.7080 | 1.0549 | 0.0003 | 0.0007 |
| C2orf40 | 15.6743 | 0.7868 | -4.3162 | 0.0000 | 0.0000 |
| KNSTRN | 2.1443 | 6.5119 | 1.6026 | 0.0000 | 0.0000 |
| HES6 | 1.4937 | 8.0729 | 2.4342 | 0.0001 | 0.0002 |
| C14orf132 | 2.4591 | 1.1429 | -1.1054 | 0.0000 | 0.0000 |
| FREM1 | 1.0481 | 0.3796 | -1.4654 | 0.0000 | 0.0000 |
| CORT | 0.1524 | 0.3679 | 1.2713 | 0.0000 | 0.0001 |
| LHX4 | 0.1274 | 0.3396 | 1.4143 | 0.0005 | 0.0013 |
| NRCAM | 0.1812 | 1.5635 | 3.1093 | 0.0021 | 0.0045 |
| NT5DC3 | 7.2428 | 1.3879 | -2.3836 | 0.0196 | 0.0314 |
| WNT2B | 1.0959 | 0.3624 | -1.5963 | 0.0000 | 0.0000 |
| FAM124A | 1.1074 | 0.3780 | -1.5508 | 0.0000 | 0.0000 |
| CCDC80 | 36.8499 | 6.6151 | -2.4778 | 0.0000 | 0.0000 |
| SPERT | 0.4041 | 0.8904 | 1.1397 | 0.0019 | 0.0041 |
| FJX1 | 2.5562 | 5.8289 | 1.1892 | 0.0013 | 0.0029 |
| FBXL8 | 1.0041 | 2.3818 | 1.2461 | 0.0000 | 0.0000 |
| DSN1 | 4.5670 | 11.6438 | 1.3503 | 0.0000 | 0.0000 |
| DCST1 | 0.0573 | 0.2479 | 2.1122 | 0.0000 | 0.0000 |
| SYT7 | 1.2118 | 4.5368 | 1.9046 | 0.0003 | 0.0009 |
| SERINC2 | 60.8495 | 158.1937 | 1.3784 | 0.0000 | 0.0001 |
| HOXC4 | 0.2891 | 1.4199 | 2.2960 | 0.0009 | 0.0021 |
| KCNJ15 | 3.7727 | 7.8466 | 1.0564 | 0.0198 | 0.0317 |
| EFNA4 | 6.1018 | 16.7821 | 1.4596 | 0.0000 | 0.0000 |
| CST4 | 0.0081 | 2.5789 | 8.3179 | 0.0000 | 0.0000 |
| HBB | 39.5619 | 15.6590 | -1.3371 | 0.0000 | 0.0001 |
| IL36RN | 0.4289 | 1.6372 | 1.9326 | 0.0000 | 0.0001 |
| SOHLH1 | 0.0074 | 0.3960 | 5.7332 | 0.0055 | 0.0103 |
| DLK2 | 1.1187 | 2.4386 | 1.1242 | 0.0302 | 0.0460 |
| CEBPA | 8.2394 | 19.1930 | 1.2200 | 0.0055 | 0.0103 |
| HAND1 | 4.8723 | 0.2909 | -4.0662 | 0.0003 | 0.0009 |
| RASGRF1 | 0.1138 | 0.4027 | 1.8227 | 0.0084 | 0.0150 |
| C11orf80 | 2.3672 | 5.5771 | 1.2363 | 0.0000 | 0.0000 |
| SLC16A8 | 0.3182 | 0.6645 | 1.0623 | 0.0276 | 0.0425 |
| MNX1 | 0.0471 | 0.5130 | 3.4444 | 0.0000 | 0.0000 |
| ZNF215 | 0.3306 | 0.7291 | 1.1409 | 0.0064 | 0.0117 |
| ACTA2 | 864.0475 | 104.9944 | -3.0408 | 0.0000 | 0.0000 |
| TNXB | 11.8447 | 0.7128 | -4.0546 | 0.0000 | 0.0000 |
| CNPY2 | 5.8433 | 12.6304 | 1.1120 | 0.0000 | 0.0000 |
| HCN3 | 0.8247 | 2.6861 | 1.7036 | 0.0000 | 0.0000 |
| CDC25B | 9.3622 | 23.1367 | 1.3053 | 0.0002 | 0.0006 |
| KCNS1 | 0.1115 | 0.6588 | 2.5629 | 0.0005 | 0.0012 |
| MYB | 0.3828 | 1.6231 | 2.0839 | 0.0001 | 0.0004 |
| ALDH2 | 17.2419 | 6.0274 | -1.5163 | 0.0000 | 0.0000 |
| CDR2L | 7.4724 | 16.2754 | 1.1230 | 0.0000 | 0.0001 |
| FLNC | 150.6621 | 6.0034 | -4.6494 | 0.0000 | 0.0000 |
| NR2C2AP | 8.9073 | 19.6470 | 1.1412 | 0.0000 | 0.0000 |
| EYA4 | 0.6979 | 0.2163 | -1.6900 | 0.0000 | 0.0000 |
| PTGIS | 54.2121 | 6.3505 | -3.0937 | 0.0000 | 0.0000 |
| PKNOX2 | 0.9942 | 0.3853 | -1.3676 | 0.0000 | 0.0000 |
| C7orf61 | 0.1501 | 0.5190 | 1.7898 | 0.0000 | 0.0000 |
| ACACB | 4.8420 | 0.8684 | -2.4791 | 0.0000 | 0.0000 |
| TRAF2 | 4.7977 | 10.3441 | 1.1084 | 0.0000 | 0.0000 |
| PANO1 | 0.1646 | 0.4999 | 1.6025 | 0.0000 | 0.0000 |
| REEP6 | 10.1887 | 22.5509 | 1.1462 | 0.0294 | 0.0449 |
| FANCG | 3.3825 | 8.8537 | 1.3882 | 0.0000 | 0.0000 |
| SYNE3 | 0.7748 | 0.3541 | -1.1296 | 0.0000 | 0.0000 |
| HOXC8 | 0.0686 | 0.6642 | 3.2750 | 0.0005 | 0.0013 |
| HMCN2 | 1.9526 | 0.1954 | -3.3211 | 0.0000 | 0.0000 |
| HDC | 1.5943 | 0.3755 | -2.0862 | 0.0000 | 0.0000 |
| PDZD7 | 0.0687 | 0.3082 | 2.1660 | 0.0000 | 0.0000 |
| E2F3 | 3.7303 | 10.3299 | 1.4695 | 0.0000 | 0.0000 |
| MDFI | 9.0522 | 25.8784 | 1.5154 | 0.0002 | 0.0006 |
| SPEG | 6.0740 | 0.8670 | -2.8085 | 0.0000 | 0.0000 |
| SPEF1 | 0.0581 | 0.2695 | 2.2140 | 0.0000 | 0.0001 |
| PLXNA3 | 2.7189 | 6.1685 | 1.1819 | 0.0000 | 0.0000 |
| XRCC3 | 1.2506 | 3.0138 | 1.2689 | 0.0000 | 0.0000 |
| PRSS1 | 0.0343 | 6.5464 | 7.5748 | 0.0028 | 0.0057 |
| TMEM125 | 2.9982 | 6.6166 | 1.1420 | 0.0012 | 0.0027 |
| TPSAB1 | 27.4220 | 5.6378 | -2.2821 | 0.0000 | 0.0000 |
| ARHGAP24 | 2.5567 | 0.8047 | -1.6678 | 0.0000 | 0.0000 |
| LRRC4B | 2.2840 | 0.7247 | -1.6560 | 0.0000 | 0.0000 |
| MUC16 | 0.0157 | 0.7142 | 5.5076 | 0.0010 | 0.0023 |
| FANCB | 0.1890 | 0.8259 | 2.1275 | 0.0000 | 0.0000 |
| GATA3 | 42.1348 | 86.2928 | 1.0342 | 0.0051 | 0.0097 |
| NR4A2 | 32.5401 | 4.7387 | -2.7797 | 0.0000 | 0.0000 |
| CLPSL2 | 0.0077 | 0.2454 | 4.9932 | 0.0000 | 0.0000 |
| ESCO2 | 0.2872 | 0.9633 | 1.7459 | 0.0000 | 0.0000 |
| FZD2 | 1.3509 | 5.8923 | 2.1249 | 0.0000 | 0.0000 |
| FAM227A | 0.0817 | 0.3551 | 2.1201 | 0.0000 | 0.0000 |
| ZNF341 | 1.1958 | 2.3964 | 1.0029 | 0.0000 | 0.0000 |
| KLF6 | 92.4865 | 35.2341 | -1.3923 | 0.0000 | 0.0000 |
| ARHGEF9 | 5.9234 | 2.6409 | -1.1654 | 0.0000 | 0.0001 |
| DKK1 | 54.6465 | 17.1068 | -1.6756 | 0.0081 | 0.0146 |
| RCAN1 | 35.2885 | 7.1288 | -2.3075 | 0.0000 | 0.0000 |
| GGCT | 20.9119 | 50.3390 | 1.2674 | 0.0000 | 0.0000 |
| FBXO43 | 0.1237 | 0.3284 | 1.4087 | 0.0000 | 0.0000 |
| HSD3B1 | 0.0226 | 1.9442 | 6.4238 | 0.0001 | 0.0002 |
| HOXB7 | 8.3365 | 20.0301 | 1.2647 | 0.0000 | 0.0000 |
| TOPBP1 | 3.8013 | 8.5362 | 1.1671 | 0.0000 | 0.0000 |
| RGS19 | 5.2533 | 11.7038 | 1.1557 | 0.0000 | 0.0000 |
| RPS6KA5 | 1.1459 | 0.4915 | -1.2211 | 0.0000 | 0.0000 |
| BUB1B | 1.2229 | 5.8808 | 2.2657 | 0.0000 | 0.0000 |
| VLDLR | 3.8753 | 1.9312 | -1.0048 | 0.0093 | 0.0164 |
| ELN | 26.4519 | 6.8002 | -1.9597 | 0.0000 | 0.0000 |
| CDK5R1 | 0.9480 | 2.1589 | 1.1873 | 0.0009 | 0.0020 |
| SLC51B | 1.4059 | 0.3304 | -2.0892 | 0.0000 | 0.0000 |
| PAGE2B | 0.0399 | 2.3130 | 5.8565 | 0.0109 | 0.0188 |
| DIXDC1 | 9.5731 | 1.3281 | -2.8497 | 0.0000 | 0.0000 |
| ITPR1 | 9.7653 | 1.8531 | -2.3977 | 0.0000 | 0.0000 |
| PFN4 | 0.3687 | 0.8648 | 1.2301 | 0.0000 | 0.0000 |
| NFKBIZ | 15.2721 | 6.1626 | -1.3093 | 0.0002 | 0.0006 |
| SGO2 | 0.7225 | 2.1235 | 1.5554 | 0.0000 | 0.0000 |
| NAP1L3 | 2.2109 | 0.6656 | -1.7319 | 0.0000 | 0.0000 |
| E2F8 | 1.3755 | 4.0421 | 1.5552 | 0.0000 | 0.0000 |
| CXCR4 | 47.4861 | 19.6747 | -1.2712 | 0.0021 | 0.0045 |
| C1orf112 | 0.8064 | 2.6785 | 1.7319 | 0.0000 | 0.0000 |
| RAMP1 | 50.9102 | 12.3098 | -2.0481 | 0.0000 | 0.0000 |
| RUNX2 | 1.2023 | 2.5262 | 1.0712 | 0.0061 | 0.0113 |
| PECAM1 | 27.0390 | 13.0303 | -1.0532 | 0.0000 | 0.0001 |
| SCN11A | 4.2814 | 0.2174 | -4.2995 | 0.0000 | 0.0000 |
| PHKG1 | 1.3127 | 0.5638 | -1.2194 | 0.0095 | 0.0167 |
| ZNF696 | 0.9608 | 2.0328 | 1.0811 | 0.0000 | 0.0000 |
| IRF9 | 1.0732 | 2.3841 | 1.1516 | 0.0000 | 0.0001 |
| CCNB1 | 6.8104 | 30.3342 | 2.1551 | 0.0000 | 0.0000 |
| HIST1H3A | 0.1191 | 1.1222 | 3.2364 | 0.0003 | 0.0008 |
| MAP1B | 23.3192 | 3.1265 | -2.8989 | 0.0000 | 0.0000 |
| NCKAP1L | 5.3899 | 2.1815 | -1.3050 | 0.0000 | 0.0001 |
| CAMK2N1 | 26.8715 | 63.7536 | 1.2464 | 0.0057 | 0.0106 |
| CKAP2L | 0.6959 | 3.7623 | 2.4347 | 0.0000 | 0.0000 |
| TTYH3 | 7.8061 | 24.8337 | 1.6696 | 0.0000 | 0.0000 |
| NRXN2 | 0.9126 | 0.2743 | -1.7344 | 0.0000 | 0.0000 |
| NMB | 5.7719 | 17.3696 | 1.5894 | 0.0000 | 0.0000 |
| ZNF497 | 0.2266 | 0.5682 | 1.3261 | 0.0000 | 0.0000 |
| APOL4 | 6.5800 | 14.6181 | 1.1516 | 0.0263 | 0.0407 |
| CD1C | 2.9232 | 1.4363 | -1.0252 | 0.0000 | 0.0001 |
| KLHDC7A | 2.9203 | 7.0850 | 1.2787 | 0.0310 | 0.0470 |
| IL17RE | 2.0636 | 4.6114 | 1.1600 | 0.0019 | 0.0040 |
| TMEM104 | 2.9538 | 5.9789 | 1.0173 | 0.0000 | 0.0000 |
| ACKR4 | 1.5094 | 0.5672 | -1.4122 | 0.0000 | 0.0000 |
| CRYM | 4.5968 | 1.0631 | -2.1124 | 0.0000 | 0.0000 |
| ALDH1L1 | 8.2054 | 3.9605 | -1.0509 | 0.0025 | 0.0051 |
| ADAMTS1 | 80.3329 | 9.0476 | -3.1504 | 0.0000 | 0.0000 |
| RCC1 | 8.4197 | 25.4986 | 1.5986 | 0.0000 | 0.0000 |
| IGSF21 | 1.5083 | 0.7224 | -1.0621 | 0.0001 | 0.0004 |
| PRDM1 | 10.5738 | 3.9981 | -1.4031 | 0.0227 | 0.0358 |
| MACC1 | 1.4785 | 3.2152 | 1.1207 | 0.0320 | 0.0484 |
| TNFSF8 | 0.8883 | 0.4418 | -1.0077 | 0.0001 | 0.0002 |
| LRRC46 | 0.1651 | 0.6537 | 1.9850 | 0.0000 | 0.0000 |
| ASB2 | 10.5361 | 1.1584 | -3.1851 | 0.0000 | 0.0000 |
| CTSG | 12.4415 | 1.8783 | -2.7276 | 0.0000 | 0.0000 |
| SEMA3G | 4.6763 | 1.5651 | -1.5791 | 0.0000 | 0.0000 |
| DEFB126 | 0.0657 | 0.8190 | 3.6400 | 0.0001 | 0.0004 |
| DENND5A | 10.3837 | 4.9844 | -1.0588 | 0.0001 | 0.0002 |
| SYNM | 228.1519 | 6.9676 | -5.0332 | 0.0000 | 0.0000 |
| ADM2 | 0.2890 | 1.4969 | 2.3729 | 0.0000 | 0.0000 |
| LIFR | 4.7634 | 1.3410 | -1.8287 | 0.0000 | 0.0000 |
| TRIM24 | 3.9275 | 9.3899 | 1.2575 | 0.0002 | 0.0005 |
| CHMP4C | 6.9872 | 16.1475 | 1.2085 | 0.0000 | 0.0000 |
| DTX3L | 8.3775 | 17.5626 | 1.0679 | 0.0000 | 0.0000 |
| POLA2 | 2.4105 | 5.5802 | 1.2110 | 0.0000 | 0.0000 |
| NUP107 | 3.2357 | 8.5733 | 1.4058 | 0.0000 | 0.0000 |
| NCAPG2 | 1.8234 | 5.5314 | 1.6010 | 0.0000 | 0.0000 |
| DDX60L | 1.1951 | 2.9213 | 1.2896 | 0.0001 | 0.0004 |
| TRIM63 | 2.7791 | 0.3616 | -2.9422 | 0.0005 | 0.0012 |
| FADS1 | 2.3952 | 5.5202 | 1.2046 | 0.0074 | 0.0134 |
| KRT33B | 0.0798 | 0.8706 | 3.4476 | 0.0055 | 0.0103 |
| HID1 | 6.5647 | 15.3644 | 1.2268 | 0.0058 | 0.0108 |
| IFI6 | 56.5329 | 370.4078 | 2.7120 | 0.0001 | 0.0002 |
| IBSP | 0.0467 | 1.8749 | 5.3260 | 0.0000 | 0.0000 |
| LARGE2 | 3.8150 | 10.9205 | 1.5173 | 0.0082 | 0.0147 |
| SLC6A12 | 0.1318 | 0.3576 | 1.4403 | 0.0237 | 0.0371 |
| QRICH2 | 0.3438 | 0.8326 | 1.2763 | 0.0005 | 0.0013 |
| WDR4 | 3.5966 | 8.5438 | 1.2483 | 0.0000 | 0.0000 |
| OR2B6 | 0.0275 | 0.5675 | 4.3654 | 0.0000 | 0.0000 |
| TENT5B | 26.1403 | 3.7789 | -2.7902 | 0.0000 | 0.0000 |
| PKDCC | 13.4060 | 2.5911 | -2.3713 | 0.0000 | 0.0000 |
| CKB | 149.9894 | 35.8242 | -2.0659 | 0.0000 | 0.0000 |
| PIP5KL1 | 0.3121 | 0.8189 | 1.3916 | 0.0005 | 0.0012 |
| ZNF600 | 1.8607 | 4.5073 | 1.2764 | 0.0000 | 0.0000 |
| NUDT5 | 7.0693 | 15.8209 | 1.1622 | 0.0000 | 0.0000 |
| CSAG1 | 1.0813 | 8.4134 | 2.9599 | 0.0008 | 0.0018 |
| TIGD2 | 2.0960 | 4.6386 | 1.1461 | 0.0000 | 0.0000 |
| SYNPO | 19.8413 | 6.7404 | -1.5576 | 0.0000 | 0.0000 |
| TMEM88B | 0.0503 | 0.4249 | 3.0793 | 0.0037 | 0.0074 |
| HSPB2 | 2.3281 | 0.5286 | -2.1388 | 0.0000 | 0.0000 |
| TMEM35A | 7.7678 | 1.0394 | -2.9018 | 0.0000 | 0.0000 |
| ECT2 | 2.5146 | 9.4621 | 1.9118 | 0.0000 | 0.0000 |
| IGIP | 5.4413 | 2.6525 | -1.0366 | 0.0000 | 0.0001 |
| MCM10 | 0.6819 | 3.2343 | 2.2459 | 0.0000 | 0.0000 |
| C20orf194 | 6.5369 | 3.1950 | -1.0328 | 0.0000 | 0.0000 |
| BVES | 5.3346 | 0.9635 | -2.4690 | 0.0000 | 0.0000 |
| NACC2 | 15.5697 | 3.4428 | -2.1771 | 0.0000 | 0.0000 |
| GAD1 | 0.2127 | 0.6624 | 1.6389 | 0.0169 | 0.0276 |
| ELOVL3 | 0.1517 | 0.6907 | 2.1870 | 0.0014 | 0.0031 |
| HIST2H2AC | 1.4356 | 6.2716 | 2.1272 | 0.0000 | 0.0000 |
| HES4 | 8.9956 | 21.1070 | 1.2304 | 0.0008 | 0.0018 |
| EFEMP1 | 37.2686 | 15.7699 | -1.2408 | 0.0000 | 0.0000 |
| PDK4 | 62.7058 | 6.3336 | -3.3075 | 0.0000 | 0.0000 |
| P4HB | 110.3674 | 233.7734 | 1.0828 | 0.0000 | 0.0000 |
| APOL2 | 15.1698 | 30.5522 | 1.0101 | 0.0005 | 0.0012 |
| SYCE2 | 0.3271 | 0.7530 | 1.2031 | 0.0019 | 0.0040 |
| PSG4 | 0.0145 | 0.5873 | 5.3404 | 0.0056 | 0.0105 |
| FAIM2 | 2.5628 | 0.2750 | -3.2203 | 0.0000 | 0.0000 |
| STAC | 1.6246 | 0.4658 | -1.8024 | 0.0000 | 0.0000 |
| TLL2 | 0.0446 | 0.2902 | 2.7023 | 0.0003 | 0.0009 |
| SLC29A2 | 1.7183 | 6.5521 | 1.9310 | 0.0000 | 0.0000 |
| CD79B | 11.0467 | 4.2240 | -1.3869 | 0.0001 | 0.0003 |
| CHDH | 0.2325 | 0.8749 | 1.9120 | 0.0002 | 0.0006 |
| ZCCHC24 | 23.1641 | 4.3679 | -2.4069 | 0.0000 | 0.0000 |
| ZNF692 | 4.4401 | 11.1806 | 1.3323 | 0.0000 | 0.0000 |
| CHPF | 23.0508 | 50.7789 | 1.1394 | 0.0000 | 0.0000 |
| ADHFE1 | 2.2196 | 1.0087 | -1.1379 | 0.0000 | 0.0000 |
| CACNB3 | 2.7905 | 7.3031 | 1.3880 | 0.0000 | 0.0000 |
| MCM2 | 4.9885 | 20.3211 | 2.0263 | 0.0000 | 0.0000 |
| PEG3 | 1.5751 | 0.6161 | -1.3543 | 0.0000 | 0.0000 |
| HIST1H3J | 0.0266 | 0.3654 | 3.7789 | 0.0000 | 0.0000 |
| DTL | 1.0922 | 4.3305 | 1.9873 | 0.0000 | 0.0000 |
| HIST2H2AB | 0.0437 | 1.6108 | 5.2028 | 0.0000 | 0.0000 |
| MGME1 | 5.9842 | 14.6190 | 1.2886 | 0.0000 | 0.0000 |
| MTCP1 | 0.3788 | 0.9241 | 1.2864 | 0.0000 | 0.0000 |
| ZNF107 | 0.9766 | 2.6706 | 1.4513 | 0.0000 | 0.0000 |
| KCNJ10 | 0.0740 | 0.2260 | 1.6103 | 0.0082 | 0.0147 |
| FAM189A2 | 4.0283 | 0.7354 | -2.4536 | 0.0000 | 0.0000 |
| MYC | 95.1203 | 35.7543 | -1.4116 | 0.0000 | 0.0001 |
| JPH4 | 0.7659 | 0.3543 | -1.1119 | 0.0000 | 0.0000 |
| HIST1H3C | 0.1037 | 1.8598 | 4.1641 | 0.0000 | 0.0000 |
| CDON | 1.7752 | 0.4662 | -1.9290 | 0.0000 | 0.0000 |
| PTCH2 | 0.8823 | 0.3131 | -1.4945 | 0.0000 | 0.0000 |
| DNAJC22 | 0.1530 | 0.6454 | 2.0766 | 0.0001 | 0.0002 |
| SCN1B | 2.3436 | 0.9963 | -1.2341 | 0.0000 | 0.0000 |
| PNPLA7 | 1.1874 | 0.4537 | -1.3879 | 0.0000 | 0.0000 |
| HOXC9 | 0.1697 | 2.1563 | 3.6679 | 0.0001 | 0.0002 |
| PHLDA1 | 45.6121 | 17.2379 | -1.4038 | 0.0159 | 0.0261 |
| RAB3IP | 0.9557 | 3.3521 | 1.8104 | 0.0000 | 0.0000 |
| ZNF432 | 1.4948 | 5.2308 | 1.8071 | 0.0000 | 0.0001 |
| NUDT8 | 4.3409 | 8.7852 | 1.0171 | 0.0000 | 0.0000 |
| ROR1 | 2.2081 | 0.4402 | -2.3265 | 0.0000 | 0.0000 |
| HPSE2 | 9.2465 | 0.8603 | -3.4260 | 0.0000 | 0.0000 |
| ANGPTL7 | 1.9716 | 0.1584 | -3.6379 | 0.0000 | 0.0000 |
| MMS22L | 0.3104 | 0.7439 | 1.2611 | 0.0000 | 0.0000 |
| PLCB4 | 6.1538 | 0.9541 | -2.6892 | 0.0000 | 0.0000 |
| LIN28B | 0.0311 | 0.2426 | 2.9651 | 0.0083 | 0.0148 |
| HSF2BP | 0.1828 | 0.6337 | 1.7940 | 0.0000 | 0.0000 |
| S100A11 | 942.8115 | 2012.6998 | 1.0941 | 0.0000 | 0.0001 |
| SMC6 | 2.1395 | 4.2808 | 1.0006 | 0.0000 | 0.0000 |
| GRB14 | 0.1730 | 0.6289 | 1.8616 | 0.0088 | 0.0155 |
| KRT8 | 163.6022 | 382.8129 | 1.2264 | 0.0003 | 0.0007 |
| MYO10 | 1.8086 | 5.4179 | 1.5828 | 0.0000 | 0.0001 |
| RAB11FIP4 | 1.7836 | 4.3579 | 1.2888 | 0.0003 | 0.0007 |
| PCDH18 | 4.4399 | 1.3055 | -1.7660 | 0.0000 | 0.0000 |
| ANK2 | 2.2262 | 0.3385 | -2.7175 | 0.0000 | 0.0000 |
| HHIP | 1.5100 | 0.2296 | -2.7173 | 0.0000 | 0.0000 |
| TMEM238 | 2.9274 | 8.3623 | 1.5143 | 0.0000 | 0.0000 |
| RBP7 | 10.6043 | 4.8554 | -1.1270 | 0.0000 | 0.0000 |
| PLAG1 | 0.3895 | 0.8998 | 1.2081 | 0.0056 | 0.0105 |
| ADCYAP1 | 1.6483 | 0.2626 | -2.6499 | 0.0000 | 0.0000 |
| SYNGR2 | 34.9759 | 71.3155 | 1.0279 | 0.0000 | 0.0000 |
| C8orf88 | 7.8607 | 1.5586 | -2.3344 | 0.0000 | 0.0000 |
| KCNN3 | 0.8743 | 0.2912 | -1.5860 | 0.0000 | 0.0000 |
| PRSS33 | 0.0183 | 0.4614 | 4.6578 | 0.0169 | 0.0276 |
| OXTR | 0.1919 | 0.9415 | 2.2949 | 0.0000 | 0.0001 |
| PBK | 1.9976 | 7.2885 | 1.8673 | 0.0000 | 0.0000 |
| MAP6 | 1.2126 | 0.5346 | -1.1817 | 0.0000 | 0.0000 |
| CSAG2 | 0.1754 | 0.9908 | 2.4979 | 0.0010 | 0.0024 |
| HIST1H2BM | 0.0062 | 0.5545 | 6.4713 | 0.0000 | 0.0000 |
| E2F7 | 0.3556 | 1.8553 | 2.3832 | 0.0000 | 0.0000 |
| FERMT1 | 5.9928 | 14.6278 | 1.2874 | 0.0000 | 0.0001 |
| TMEM56 | 3.1781 | 1.5637 | -1.0233 | 0.0001 | 0.0003 |
| TRIM45 | 1.5016 | 3.1567 | 1.0719 | 0.0002 | 0.0006 |
| ACKR1 | 62.3767 | 7.8815 | -2.9845 | 0.0000 | 0.0000 |
| XCL1 | 0.6199 | 2.0566 | 1.7302 | 0.0014 | 0.0031 |
| PPARGC1A | 0.6600 | 0.2079 | -1.6662 | 0.0000 | 0.0000 |
| GSN | 168.7679 | 36.7816 | -2.1980 | 0.0000 | 0.0000 |
| SPDYC | 0.0155 | 1.0135 | 6.0350 | 0.0000 | 0.0001 |
| DUSP5 | 136.9614 | 31.3084 | -2.1291 | 0.0000 | 0.0002 |
| SGMS2 | 5.7947 | 2.5978 | -1.1574 | 0.0001 | 0.0004 |
| RFX8 | 0.0814 | 0.2851 | 1.8091 | 0.0062 | 0.0114 |
| CD248 | 45.3202 | 17.6300 | -1.3621 | 0.0000 | 0.0000 |
| CXorf36 | 3.7710 | 1.7137 | -1.1379 | 0.0000 | 0.0000 |
| PYGB | 110.0407 | 40.1199 | -1.4556 | 0.0038 | 0.0076 |
| CST6 | 2.5258 | 43.7010 | 4.1129 | 0.0000 | 0.0000 |
| SMOC2 | 47.3837 | 7.2474 | -2.7089 | 0.0000 | 0.0000 |
| EDNRB | 8.4013 | 2.4963 | -1.7508 | 0.0000 | 0.0000 |
| NOVA1 | 0.9919 | 0.1860 | -2.4150 | 0.0000 | 0.0000 |
| PCDHGC3 | 4.3207 | 2.1410 | -1.0129 | 0.0001 | 0.0002 |
| LRRC61 | 3.2707 | 8.4101 | 1.3625 | 0.0000 | 0.0000 |
| MN1 | 2.0996 | 0.8509 | -1.3030 | 0.0000 | 0.0000 |
| RCOR2 | 1.0062 | 3.4896 | 1.7941 | 0.0049 | 0.0094 |
| OLFM1 | 3.4869 | 0.8360 | -2.0603 | 0.0000 | 0.0000 |
| WDR76 | 1.5948 | 4.0853 | 1.3571 | 0.0000 | 0.0000 |
| FBXL22 | 6.4418 | 0.7035 | -3.1948 | 0.0000 | 0.0000 |
| SPINT2 | 67.6253 | 148.0272 | 1.1302 | 0.0000 | 0.0000 |
| HIST1H2BE | 0.0827 | 1.8258 | 4.4652 | 0.0000 | 0.0000 |
| MEDAG | 16.2647 | 3.7661 | -2.1106 | 0.0000 | 0.0000 |
| TUBG1 | 12.9884 | 29.7118 | 1.1938 | 0.0000 | 0.0000 |
| CALHM3 | 0.0725 | 1.3466 | 4.2149 | 0.0062 | 0.0115 |
| ADRA2B | 1.0118 | 0.4285 | -1.2394 | 0.0001 | 0.0003 |
| LRFN5 | 2.1777 | 0.2694 | -3.0152 | 0.0000 | 0.0000 |
| PDZD4 | 4.1555 | 0.8280 | -2.3274 | 0.0000 | 0.0000 |
| CLIP3 | 28.2417 | 4.7576 | -2.5695 | 0.0000 | 0.0000 |
| MIEN1 | 13.9819 | 38.7423 | 1.4703 | 0.0000 | 0.0000 |
| AIFM3 | 0.5906 | 2.5719 | 2.1226 | 0.0005 | 0.0013 |
| RTN4RL2 | 0.2418 | 0.7081 | 1.5502 | 0.0044 | 0.0084 |
| TCEAL3 | 22.4484 | 11.0727 | -1.0196 | 0.0000 | 0.0000 |
| LRWD1 | 2.3630 | 5.0283 | 1.0895 | 0.0000 | 0.0000 |
| CXCL5 | 0.3321 | 4.8464 | 3.8673 | 0.0019 | 0.0040 |
| GPR84 | 0.3149 | 0.9300 | 1.5624 | 0.0007 | 0.0017 |
| ZNF761 | 2.0356 | 6.1622 | 1.5980 | 0.0000 | 0.0000 |
| TEDC2 | 0.8448 | 3.8469 | 2.1871 | 0.0000 | 0.0000 |
| IGDCC4 | 1.4358 | 0.5442 | -1.3996 | 0.0000 | 0.0000 |
| ENTPD8 | 0.0578 | 1.1678 | 4.3355 | 0.0081 | 0.0145 |
| ALPG | 0.0084 | 0.6760 | 6.3273 | 0.0001 | 0.0004 |
| PIPOX | 0.8970 | 0.3111 | -1.5279 | 0.0000 | 0.0001 |
| PCDHB9 | 0.3535 | 0.7989 | 1.1764 | 0.0094 | 0.0165 |
| SLC16A13 | 1.6350 | 4.3009 | 1.3953 | 0.0000 | 0.0000 |
| LONRF3 | 1.2874 | 0.6023 | -1.0959 | 0.0004 | 0.0011 |
| MAT2A | 75.9767 | 29.5717 | -1.3613 | 0.0001 | 0.0002 |
| FAXDC2 | 5.4444 | 0.9361 | -2.5401 | 0.0000 | 0.0000 |
| SEC61A2 | 1.2107 | 2.5509 | 1.0751 | 0.0000 | 0.0000 |
| ACYP1 | 2.2045 | 5.8484 | 1.4076 | 0.0000 | 0.0000 |
| CDK2 | 7.4066 | 17.0603 | 1.2038 | 0.0000 | 0.0000 |
| SLC4A11 | 2.9146 | 6.4922 | 1.1554 | 0.0018 | 0.0039 |
| MEX3A | 1.2710 | 7.8522 | 2.6271 | 0.0000 | 0.0000 |
| C15orf48 | 5.9181 | 30.8053 | 2.3800 | 0.0001 | 0.0003 |
| NCOA7 | 14.9002 | 7.0245 | -1.0849 | 0.0000 | 0.0001 |
| ITPR3 | 3.5914 | 10.5813 | 1.5589 | 0.0000 | 0.0000 |
| ARHGAP8 | 0.1785 | 0.4970 | 1.4775 | 0.0000 | 0.0000 |
| ODAPH | 0.0165 | 0.8073 | 5.6089 | 0.0000 | 0.0000 |
| BRCA2 | 0.3191 | 1.2473 | 1.9666 | 0.0000 | 0.0000 |
| CDCA2 | 0.5939 | 2.9302 | 2.3026 | 0.0000 | 0.0000 |
| HIST1H2BI | 0.0279 | 1.9799 | 6.1466 | 0.0000 | 0.0000 |
| ABCB9 | 0.3007 | 0.7390 | 1.2975 | 0.0000 | 0.0000 |
| AGMAT | 0.9221 | 2.4586 | 1.4148 | 0.0000 | 0.0000 |
| APOLD1 | 17.5406 | 3.7918 | -2.2097 | 0.0000 | 0.0000 |
| CPQ | 18.5894 | 7.2267 | -1.3631 | 0.0000 | 0.0000 |
| AKR1B1 | 21.3937 | 63.3260 | 1.5656 | 0.0006 | 0.0016 |
| MGARP | 0.7309 | 0.1817 | -2.0084 | 0.0000 | 0.0001 |
| HBEGF | 74.4291 | 11.5628 | -2.6864 | 0.0000 | 0.0001 |
| ARHGAP6 | 3.2943 | 0.9368 | -1.8142 | 0.0000 | 0.0000 |
| SMG9 | 3.3441 | 6.7620 | 1.0158 | 0.0000 | 0.0000 |
| PDE1A | 3.2791 | 0.5254 | -2.6419 | 0.0000 | 0.0000 |
| HIST1H2AI | 0.1296 | 3.1943 | 4.6232 | 0.0000 | 0.0000 |
| PDZRN4 | 7.2105 | 0.3234 | -4.4786 | 0.0000 | 0.0000 |
| AKR7A3 | 0.2966 | 0.7166 | 1.2728 | 0.0001 | 0.0004 |
| WNT3 | 0.2149 | 1.2580 | 2.5496 | 0.0000 | 0.0000 |
| CDH3 | 4.5973 | 31.9048 | 2.7949 | 0.0000 | 0.0001 |
| TMEM145 | 0.0736 | 0.6156 | 3.0650 | 0.0000 | 0.0000 |
| RAB17 | 1.1407 | 2.9501 | 1.3708 | 0.0002 | 0.0006 |
| DTYMK | 7.0398 | 16.7662 | 1.2519 | 0.0000 | 0.0000 |
| HIST1H2AL | 0.0454 | 1.0363 | 4.5122 | 0.0000 | 0.0000 |
| SALL4 | 0.1522 | 1.4427 | 3.2451 | 0.0000 | 0.0000 |
| ABCC4 | 7.8728 | 3.4674 | -1.1830 | 0.0001 | 0.0002 |
| NSD2 | 2.4847 | 5.4540 | 1.1342 | 0.0000 | 0.0000 |
| MSLN | 0.7447 | 8.9585 | 3.5886 | 0.0100 | 0.0173 |
| FCGR1A | 0.3644 | 1.3486 | 1.8880 | 0.0033 | 0.0067 |
| CHRNA1 | 0.0219 | 0.3872 | 4.1437 | 0.0000 | 0.0000 |
| SLC25A15 | 1.4062 | 2.8221 | 1.0050 | 0.0000 | 0.0000 |
| PIDD1 | 2.6262 | 5.3336 | 1.0221 | 0.0000 | 0.0000 |
| LONRF2 | 1.8640 | 0.2971 | -2.6492 | 0.0000 | 0.0000 |
| IGSF10 | 1.9107 | 0.1841 | -3.3752 | 0.0000 | 0.0000 |
| POU5F1 | 1.0754 | 4.6919 | 2.1253 | 0.0011 | 0.0025 |
| KIF22 | 9.3701 | 23.0311 | 1.2974 | 0.0000 | 0.0000 |
| RNASE4 | 1.2325 | 0.3662 | -1.7508 | 0.0000 | 0.0000 |
| SLC24A3 | 10.0749 | 2.3590 | -2.0945 | 0.0000 | 0.0000 |
| UBALD2 | 31.3963 | 71.9990 | 1.1974 | 0.0000 | 0.0000 |
| GGT2 | 0.0153 | 0.2712 | 4.1482 | 0.0008 | 0.0019 |
| RSPO2 | 1.0390 | 0.2137 | -2.2813 | 0.0000 | 0.0000 |
| HIST1H2BD | 4.7513 | 26.4295 | 2.4758 | 0.0000 | 0.0000 |
| ATP12A | 0.0845 | 0.7497 | 3.1491 | 0.0147 | 0.0244 |
| NFATC2 | 4.2783 | 1.6603 | -1.3656 | 0.0000 | 0.0000 |
| SVOPL | 0.1306 | 0.3042 | 1.2204 | 0.0096 | 0.0168 |
| ATAD3A | 7.3862 | 15.8462 | 1.1012 | 0.0000 | 0.0000 |
| C1orf159 | 1.7311 | 4.3586 | 1.3321 | 0.0000 | 0.0000 |
| METTL27 | 1.1086 | 2.8323 | 1.3533 | 0.0052 | 0.0099 |
| PROK1 | 0.5905 | 0.2607 | -1.1794 | 0.0001 | 0.0004 |
| RBKS | 2.9577 | 1.2582 | -1.2332 | 0.0000 | 0.0000 |
| RNF180 | 1.8887 | 0.3773 | -2.3238 | 0.0000 | 0.0000 |
| AURKA | 2.1353 | 12.3834 | 2.5359 | 0.0000 | 0.0000 |
| FAXC | 1.3094 | 0.3907 | -1.7449 | 0.0120 | 0.0204 |
| PRAC2 | 2.2257 | 0.9522 | -1.2249 | 0.0000 | 0.0001 |
| C3orf67 | 0.1188 | 0.5184 | 2.1258 | 0.0000 | 0.0001 |
| INA | 3.4452 | 14.3272 | 2.0561 | 0.0074 | 0.0135 |
| SVIL | 63.5447 | 10.7677 | -2.5611 | 0.0001 | 0.0003 |
| CLPB | 2.6451 | 5.6724 | 1.1006 | 0.0000 | 0.0000 |
| SEC14L2 | 0.9828 | 3.0353 | 1.6269 | 0.0000 | 0.0001 |
| SGK494 | 0.3996 | 1.0683 | 1.4186 | 0.0001 | 0.0002 |
| BST2 | 88.1137 | 193.8836 | 1.1378 | 0.0176 | 0.0286 |
| RANBP17 | 0.1382 | 0.3731 | 1.4325 | 0.0016 | 0.0035 |
| SBSPON | 17.7369 | 2.2891 | -2.9539 | 0.0000 | 0.0000 |
| MCM5 | 6.5395 | 14.5278 | 1.1516 | 0.0000 | 0.0000 |
| ZNF737 | 1.6181 | 5.3555 | 1.7268 | 0.0294 | 0.0449 |
| KLF2 | 50.1027 | 7.8978 | -2.6654 | 0.0000 | 0.0000 |
| CCDC189 | 0.1839 | 0.6267 | 1.7689 | 0.0000 | 0.0000 |
| LGI4 | 3.9533 | 0.9313 | -2.0857 | 0.0000 | 0.0000 |
| CH25H | 15.9923 | 2.0900 | -2.9358 | 0.0000 | 0.0000 |
| IGF2BP3 | 0.0353 | 0.8917 | 4.6592 | 0.0002 | 0.0005 |
| TNNT1 | 0.2767 | 5.8118 | 4.3926 | 0.0000 | 0.0000 |
| MORN3 | 0.2536 | 0.8412 | 1.7299 | 0.0003 | 0.0007 |
| SELENOP | 13.7630 | 6.8034 | -1.0165 | 0.0000 | 0.0000 |
| STAC3 | 1.2818 | 2.7381 | 1.0950 | 0.0000 | 0.0000 |
| TGFB3 | 10.5770 | 4.6773 | -1.1772 | 0.0000 | 0.0000 |
| SEM1 | 6.5860 | 13.7104 | 1.0578 | 0.0000 | 0.0000 |
| NTF3 | 1.4520 | 0.3023 | -2.2641 | 0.0000 | 0.0000 |
| MGLL | 14.0120 | 4.1869 | -1.7427 | 0.0000 | 0.0000 |
| ATAD3B | 2.7937 | 5.7802 | 1.0490 | 0.0000 | 0.0000 |
| YEATS4 | 6.0512 | 16.5057 | 1.4477 | 0.0000 | 0.0000 |
| AFF3 | 2.4820 | 0.2203 | -3.4939 | 0.0000 | 0.0000 |
| KISS1 | 0.8949 | 9.0979 | 3.3457 | 0.0048 | 0.0092 |
| CLDN2 | 0.0203 | 0.2390 | 3.5595 | 0.0000 | 0.0001 |
| PABPC1 | 326.8946 | 710.9111 | 1.1208 | 0.0007 | 0.0017 |
| PYDC1 | 0.0450 | 0.2719 | 2.5949 | 0.0210 | 0.0334 |
| RGL3 | 2.5346 | 6.7266 | 1.4081 | 0.0116 | 0.0199 |
| PREX2 | 1.3406 | 0.4004 | -1.7432 | 0.0000 | 0.0000 |
| PCNA | 42.7351 | 119.8052 | 1.4872 | 0.0000 | 0.0000 |
| PLPP4 | 0.1289 | 2.5141 | 4.2852 | 0.0000 | 0.0001 |
| CILP | 24.2077 | 3.2693 | -2.8884 | 0.0000 | 0.0000 |
| ADH1C | 20.1049 | 6.7863 | -1.5669 | 0.0001 | 0.0002 |
| CYS1 | 2.7215 | 0.8553 | -1.6699 | 0.0000 | 0.0000 |
| SLC23A3 | 0.1835 | 0.6565 | 1.8388 | 0.0000 | 0.0000 |
| LBP | 0.0820 | 1.8712 | 4.5124 | 0.0122 | 0.0207 |
| EPHX4 | 0.4941 | 1.1490 | 1.2176 | 0.0054 | 0.0101 |
| DNMT3B | 0.8915 | 4.4842 | 2.3305 | 0.0000 | 0.0000 |
| SASS6 | 1.0298 | 2.7795 | 1.4325 | 0.0000 | 0.0000 |
| LRRN4CL | 6.1143 | 1.0163 | -2.5889 | 0.0000 | 0.0000 |
| CSAG3 | 0.1721 | 1.8209 | 3.4035 | 0.0020 | 0.0043 |
| RTL5 | 3.7216 | 1.0418 | -1.8369 | 0.0000 | 0.0000 |
| TRPC1 | 2.1730 | 0.9577 | -1.1820 | 0.0000 | 0.0000 |
| CCR3 | 0.0435 | 0.4122 | 3.2443 | 0.0000 | 0.0000 |
| PPP1R12A | 16.5622 | 5.3717 | -1.6244 | 0.0001 | 0.0004 |
| UPK3B | 37.4429 | 77.3936 | 1.0475 | 0.0288 | 0.0442 |
| FAM110B | 2.3311 | 0.7744 | -1.5899 | 0.0000 | 0.0000 |
| ACRBP | 0.4336 | 1.0781 | 1.3141 | 0.0006 | 0.0015 |
| ZNF468 | 2.0541 | 4.8367 | 1.2355 | 0.0000 | 0.0000 |
| SLC23A1 | 0.1044 | 0.3921 | 1.9086 | 0.0039 | 0.0076 |
| GINS4 | 0.4911 | 1.6170 | 1.7192 | 0.0000 | 0.0000 |
| CD300LB | 0.5539 | 0.2514 | -1.1396 | 0.0015 | 0.0034 |
| CDH24 | 1.5903 | 4.9053 | 1.6250 | 0.0000 | 0.0000 |
| IFITM5 | 0.0101 | 0.2324 | 4.5238 | 0.0326 | 0.0492 |
| TMEM206 | 1.8369 | 5.6729 | 1.6268 | 0.0000 | 0.0000 |
| GARNL3 | 1.5875 | 0.5617 | -1.4988 | 0.0000 | 0.0000 |
| COL11A2 | 0.1209 | 0.3529 | 1.5456 | 0.0028 | 0.0057 |
| SPATA33 | 0.7076 | 1.4154 | 1.0003 | 0.0000 | 0.0000 |
| UCN | 0.6000 | 1.2443 | 1.0523 | 0.0103 | 0.0179 |
| EIF4E3 | 4.9741 | 2.2388 | -1.1517 | 0.0000 | 0.0000 |
| SCN7A | 1.7034 | 0.1524 | -3.4821 | 0.0000 | 0.0000 |
| SRRM3 | 1.1746 | 2.4590 | 1.0659 | 0.0165 | 0.0269 |
| SCX | 1.1312 | 3.6446 | 1.6879 | 0.0000 | 0.0000 |
| CKLF-CMTM1 | 0.1176 | 0.2617 | 1.1543 | 0.0000 | 0.0001 |
| MKRN2OS | 1.0033 | 3.5279 | 1.8140 | 0.0000 | 0.0000 |
| LMOD1 | 196.0279 | 11.5485 | -4.0853 | 0.0000 | 0.0000 |
| OVOL3 | 0.1276 | 0.2601 | 1.0271 | 0.0000 | 0.0001 |
| HMMR | 1.1672 | 5.6458 | 2.2742 | 0.0000 | 0.0000 |
| CNRIP1 | 5.8094 | 1.8663 | -1.6382 | 0.0000 | 0.0000 |
| ZNF284 | 0.3972 | 0.8100 | 1.0278 | 0.0000 | 0.0000 |
| USP18 | 1.3901 | 4.4644 | 1.6833 | 0.0000 | 0.0000 |
| CCDC151 | 0.1518 | 0.4283 | 1.4960 | 0.0110 | 0.0190 |
| CDT1 | 2.4753 | 11.7068 | 2.2417 | 0.0000 | 0.0000 |
| TMEM191B | 0.0409 | 0.3139 | 2.9389 | 0.0000 | 0.0000 |
| TNFSF12 | 20.9430 | 9.1494 | -1.1947 | 0.0000 | 0.0000 |
| MEF2D | 18.6638 | 8.6009 | -1.1177 | 0.0000 | 0.0000 |
| GTF2H4 | 0.7038 | 1.4137 | 1.0063 | 0.0000 | 0.0000 |
| SLC39A11 | 5.1657 | 11.1285 | 1.1072 | 0.0000 | 0.0000 |
| SCRG1 | 1.8768 | 0.3659 | -2.3586 | 0.0000 | 0.0000 |
| CDK5 | 3.2624 | 7.4931 | 1.1996 | 0.0000 | 0.0000 |
| HIST2H2BF | 0.0953 | 1.4487 | 3.9262 | 0.0000 | 0.0000 |
| UQCC3 | 6.2634 | 13.5960 | 1.1182 | 0.0000 | 0.0000 |
| PLCD4 | 3.5338 | 0.5792 | -2.6092 | 0.0000 | 0.0000 |
| TMEM171 | 0.6272 | 1.4693 | 1.2282 | 0.0119 | 0.0203 |
| ROMO1 | 62.6711 | 130.8765 | 1.0623 | 0.0000 | 0.0000 |
| SLC39A4 | 2.4356 | 10.2888 | 2.0787 | 0.0000 | 0.0000 |
| TOP2A | 6.1722 | 30.2177 | 2.2915 | 0.0000 | 0.0000 |
| ATAT1 | 2.5023 | 5.6877 | 1.1846 | 0.0000 | 0.0000 |
| LMNB2 | 10.8851 | 26.9768 | 1.3094 | 0.0000 | 0.0000 |
| TFR2 | 0.1903 | 1.1289 | 2.5686 | 0.0000 | 0.0000 |
| NRG2 | 1.3867 | 0.4158 | -1.7375 | 0.0000 | 0.0000 |
| NUP85 | 5.9087 | 12.8040 | 1.1157 | 0.0000 | 0.0000 |
| KDM6B | 19.8578 | 9.0722 | -1.1302 | 0.0002 | 0.0007 |
| ZC3H12B | 0.4880 | 0.2399 | -1.0245 | 0.0000 | 0.0000 |
| ACTN1 | 64.9789 | 28.9581 | -1.1660 | 0.0165 | 0.0269 |
| INHA | 0.2022 | 1.0332 | 2.3533 | 0.0001 | 0.0002 |
| KATNAL1 | 3.0187 | 1.1680 | -1.3699 | 0.0017 | 0.0036 |
| SLC28A3 | 0.2566 | 1.6217 | 2.6597 | 0.0013 | 0.0029 |
| RIC3 | 1.0577 | 0.3044 | -1.7970 | 0.0000 | 0.0000 |
| ITGA1 | 8.4630 | 2.4311 | -1.7995 | 0.0011 | 0.0026 |
| ACTC1 | 220.2905 | 7.1666 | -4.9420 | 0.0000 | 0.0000 |
| KRT34 | 0.0598 | 0.9782 | 4.0325 | 0.0025 | 0.0052 |
| MYOT | 0.9435 | 0.1817 | -2.3760 | 0.0000 | 0.0000 |
| ACRV1 | 0.0840 | 0.2303 | 1.4550 | 0.0000 | 0.0000 |
| PTX3 | 13.0602 | 3.1913 | -2.0330 | 0.0000 | 0.0001 |
| CENPL | 0.7178 | 1.9692 | 1.4560 | 0.0000 | 0.0000 |
| ZMAT1 | 1.4326 | 0.7018 | -1.0294 | 0.0000 | 0.0000 |
| S100A7A | 0.1679 | 3.1239 | 4.2178 | 0.0010 | 0.0024 |
| RLN2 | 0.1022 | 0.5187 | 2.3440 | 0.0004 | 0.0010 |
| IL22RA1 | 0.9433 | 2.5687 | 1.4453 | 0.0000 | 0.0000 |
| TMEM106C | 10.0805 | 22.3196 | 1.1467 | 0.0000 | 0.0000 |
| PKIB | 0.6949 | 2.5695 | 1.8867 | 0.0022 | 0.0046 |
| THSD4 | 5.4063 | 1.3315 | -2.0215 | 0.0000 | 0.0000 |
| EFHC2 | 0.5828 | 0.2591 | -1.1694 | 0.0000 | 0.0000 |
| GBX1 | 0.0031 | 0.2151 | 6.1362 | 0.0002 | 0.0006 |
| ARC | 2.4727 | 0.6116 | -2.0153 | 0.0000 | 0.0000 |
| RASGEF1A | 0.5464 | 1.6246 | 1.5721 | 0.0117 | 0.0201 |
| MSI1 | 0.2641 | 1.9070 | 2.8523 | 0.0033 | 0.0066 |
| MITF | 3.3856 | 1.0033 | -1.7546 | 0.0000 | 0.0000 |
| TRMT2A | 5.9354 | 12.5972 | 1.0857 | 0.0000 | 0.0000 |
| NANOS3 | 0.3494 | 0.7418 | 1.0862 | 0.0034 | 0.0067 |
| KIAA1755 | 1.1640 | 0.5670 | -1.0376 | 0.0002 | 0.0007 |
| RHBDD3 | 4.8704 | 10.8654 | 1.1576 | 0.0000 | 0.0000 |
| CNN1 | 670.6525 | 34.5311 | -4.2796 | 0.0000 | 0.0000 |
| HIST1H2AC | 8.8408 | 27.6783 | 1.6465 | 0.0000 | 0.0000 |
| ADAMTS8 | 3.8138 | 0.5504 | -2.7926 | 0.0000 | 0.0000 |
| FBXO5 | 1.2229 | 2.9741 | 1.2822 | 0.0000 | 0.0000 |
| PDZK1 | 0.0532 | 0.5406 | 3.3464 | 0.0000 | 0.0000 |
| CPXM2 | 21.2445 | 3.4458 | -2.6242 | 0.0000 | 0.0000 |
| CLU | 201.5167 | 67.6991 | -1.5737 | 0.0000 | 0.0000 |
| KRT40 | 0.0282 | 0.4852 | 4.1044 | 0.0193 | 0.0310 |
| PCSK7 | 3.7167 | 1.2054 | -1.6245 | 0.0000 | 0.0000 |
| NEURL3 | 0.1864 | 0.9964 | 2.4187 | 0.0071 | 0.0130 |
| KCNMB3 | 0.1153 | 0.2974 | 1.3668 | 0.0000 | 0.0000 |
| CKMT2 | 1.7424 | 0.3507 | -2.3126 | 0.0000 | 0.0000 |
| ARID3A | 0.9785 | 4.2947 | 2.1339 | 0.0005 | 0.0012 |
| CAP2 | 9.6986 | 2.5739 | -1.9138 | 0.0000 | 0.0000 |
| TSHZ2 | 1.6220 | 0.7429 | -1.1265 | 0.0000 | 0.0000 |
| FDX2 | 1.1542 | 2.4973 | 1.1134 | 0.0000 | 0.0000 |
| IFFO1 | 3.1586 | 1.5327 | -1.0432 | 0.0000 | 0.0001 |
| SLC25A19 | 1.5702 | 3.2550 | 1.0517 | 0.0000 | 0.0000 |
| HIST1H4H | 1.5759 | 7.9326 | 2.3316 | 0.0057 | 0.0107 |
| SPRY1 | 28.8122 | 8.1985 | -1.8132 | 0.0000 | 0.0000 |
| CCL14 | 2.2028 | 0.2610 | -3.0774 | 0.0000 | 0.0000 |
| JAM3 | 19.5551 | 3.5410 | -2.4653 | 0.0000 | 0.0000 |
| ARHGAP40 | 1.7473 | 5.7149 | 1.7096 | 0.0003 | 0.0009 |
| BMP8B | 0.8055 | 2.8319 | 1.8137 | 0.0000 | 0.0000 |
| MROH6 | 2.5821 | 7.3983 | 1.5186 | 0.0000 | 0.0001 |
| PLA2G7 | 1.7614 | 4.4083 | 1.3235 | 0.0011 | 0.0025 |
| CCNE1 | 2.2712 | 11.6451 | 2.3582 | 0.0000 | 0.0000 |
| BLM | 0.5688 | 2.2130 | 1.9601 | 0.0000 | 0.0000 |
| CPEB4 | 6.3543 | 2.4583 | -1.3701 | 0.0000 | 0.0000 |
| RBM24 | 3.3939 | 0.5148 | -2.7210 | 0.0000 | 0.0000 |
| EGR3 | 21.9725 | 1.9379 | -3.5031 | 0.0000 | 0.0000 |
| CRYAB | 72.9754 | 7.1650 | -3.3484 | 0.0000 | 0.0000 |
| WDR62 | 0.5898 | 1.8929 | 1.6824 | 0.0000 | 0.0000 |
| CENPM | 3.0857 | 9.5855 | 1.6352 | 0.0000 | 0.0000 |
| EDNRA | 9.7135 | 2.7926 | -1.7984 | 0.0000 | 0.0000 |
| PIMREG | 1.0108 | 5.2858 | 2.3866 | 0.0000 | 0.0000 |
| LDB3 | 1.9229 | 0.3464 | -2.4729 | 0.0000 | 0.0000 |
| WDHD1 | 0.9486 | 3.1928 | 1.7510 | 0.0000 | 0.0000 |
| FAM110D | 3.8062 | 1.0656 | -1.8367 | 0.0000 | 0.0000 |
| MOCOS | 2.0503 | 4.3352 | 1.0802 | 0.0000 | 0.0001 |
| CFD | 225.1983 | 18.8575 | -3.5780 | 0.0000 | 0.0000 |
| TSACC | 0.1517 | 0.6791 | 2.1623 | 0.0000 | 0.0000 |
| ERVMER34-1 | 0.4626 | 2.2856 | 2.3048 | 0.0000 | 0.0000 |
| IFI27 | 19.1033 | 88.5550 | 2.2128 | 0.0036 | 0.0072 |
| JPT1 | 25.3791 | 71.7704 | 1.4997 | 0.0000 | 0.0000 |
| ALDH1B1 | 81.9402 | 16.8738 | -2.2798 | 0.0001 | 0.0003 |
| BRCA1 | 0.9776 | 3.0501 | 1.6415 | 0.0000 | 0.0000 |
| CX3CR1 | 0.5728 | 0.2720 | -1.0745 | 0.0013 | 0.0029 |
| ABHD3 | 2.5191 | 5.3230 | 1.0794 | 0.0000 | 0.0000 |
| AARD | 4.3175 | 0.3164 | -3.7702 | 0.0000 | 0.0000 |
| GEMIN6 | 2.0052 | 4.1322 | 1.0432 | 0.0000 | 0.0000 |
| CD80 | 0.1442 | 0.3434 | 1.2515 | 0.0189 | 0.0305 |
| DAPL1 | 14.3786 | 3.5434 | -2.0207 | 0.0175 | 0.0284 |
| RERGL | 2.8290 | 0.2739 | -3.3684 | 0.0000 | 0.0000 |
| CENPA | 0.9031 | 6.3095 | 2.8045 | 0.0000 | 0.0000 |
| DPF1 | 0.0771 | 0.3340 | 2.1153 | 0.0001 | 0.0002 |
| DUSP26 | 0.4652 | 0.1969 | -1.2404 | 0.0000 | 0.0000 |
| BAG2 | 11.4663 | 2.2906 | -2.3236 | 0.0000 | 0.0000 |
| HIST1H4I | 2.2669 | 10.2755 | 2.1804 | 0.0000 | 0.0000 |
| CIP2A | 0.9397 | 3.4312 | 1.8685 | 0.0000 | 0.0000 |
| SH3D21 | 0.8882 | 2.0177 | 1.1838 | 0.0000 | 0.0001 |
| NAT8 | 0.0533 | 0.2918 | 2.4533 | 0.0161 | 0.0264 |
| LY6K | 1.5432 | 4.3353 | 1.4902 | 0.0013 | 0.0029 |
| ADARB1 | 12.7943 | 2.6385 | -2.2777 | 0.0003 | 0.0009 |
| LIG1 | 3.8188 | 10.4559 | 1.4531 | 0.0000 | 0.0000 |
| KCNC3 | 0.4038 | 1.6520 | 2.0325 | 0.0000 | 0.0002 |
| HIST1H3D | 0.4047 | 5.8136 | 3.8445 | 0.0000 | 0.0000 |
| KDF1 | 7.8178 | 17.2316 | 1.1402 | 0.0000 | 0.0000 |
| BDKRB2 | 10.1893 | 2.6922 | -1.9202 | 0.0000 | 0.0000 |
| GNAO1 | 3.7280 | 0.4443 | -3.0687 | 0.0000 | 0.0000 |
| CD40LG | 0.8073 | 0.2871 | -1.4919 | 0.0001 | 0.0002 |
| SP6 | 4.4126 | 12.0562 | 1.4501 | 0.0035 | 0.0070 |
| ALPP | 0.0270 | 5.2868 | 7.6151 | 0.0000 | 0.0000 |
| FAM136A | 9.6360 | 19.6983 | 1.0316 | 0.0000 | 0.0000 |
| EMX1 | 0.0426 | 0.2668 | 2.6462 | 0.0001 | 0.0004 |
| ASB5 | 10.6306 | 0.4071 | -4.7068 | 0.0000 | 0.0000 |
| YIF1B | 6.9658 | 16.5863 | 1.2516 | 0.0000 | 0.0000 |
| GSTM5 | 3.6677 | 0.8889 | -2.0447 | 0.0000 | 0.0000 |
| MST1R | 3.7813 | 8.2248 | 1.1211 | 0.0274 | 0.0422 |
| TMSB10 | 1497.0211 | 3090.2323 | 1.0456 | 0.0000 | 0.0000 |
| ARMCX1 | 12.6915 | 4.3459 | -1.5461 | 0.0000 | 0.0000 |
| RERG | 5.8104 | 1.1315 | -2.3605 | 0.0000 | 0.0000 |
| GDNF | 0.4135 | 0.1951 | -1.0835 | 0.0020 | 0.0042 |
| FAM72B | 0.1238 | 0.8241 | 2.7348 | 0.0000 | 0.0000 |
| HOXB9 | 1.2597 | 4.3733 | 1.7956 | 0.0018 | 0.0038 |
| C1QTNF7 | 3.9812 | 0.3449 | -3.5288 | 0.0000 | 0.0000 |
| SRCIN1 | 0.7061 | 1.9363 | 1.4554 | 0.0032 | 0.0065 |
| ISL2 | 0.2508 | 0.9871 | 1.9765 | 0.0000 | 0.0000 |
| KRT9 | 0.0611 | 0.6967 | 3.5123 | 0.0001 | 0.0002 |
| CDK1 | 2.7757 | 15.8437 | 2.5130 | 0.0000 | 0.0000 |
| SLC26A6 | 1.0324 | 4.0691 | 1.9788 | 0.0000 | 0.0000 |
| TARBP1 | 2.3904 | 5.6092 | 1.2306 | 0.0000 | 0.0000 |
| RFWD3 | 3.5239 | 7.8313 | 1.1521 | 0.0000 | 0.0000 |
| LIN9 | 0.8859 | 2.1270 | 1.2637 | 0.0000 | 0.0000 |
| NAA40 | 2.6777 | 6.8478 | 1.3547 | 0.0000 | 0.0000 |
| EPHA7 | 4.7506 | 0.6346 | -2.9042 | 0.0000 | 0.0000 |
| FYCO1 | 18.6030 | 5.7031 | -1.7057 | 0.0000 | 0.0000 |
| TAP1 | 18.5810 | 48.2578 | 1.3769 | 0.0097 | 0.0170 |
| PLIN4 | 24.4847 | 5.0545 | -2.2762 | 0.0000 | 0.0000 |
| SIK1 | 4.2433 | 1.5408 | -1.4615 | 0.0007 | 0.0018 |
| IFI44 | 8.8337 | 26.1638 | 1.5665 | 0.0045 | 0.0087 |
| ZDHHC11B | 1.7031 | 0.4825 | -1.8195 | 0.0000 | 0.0000 |
| COLGALT1 | 17.3863 | 34.9166 | 1.0060 | 0.0000 | 0.0000 |
| CYP1B1 | 12.0248 | 4.9039 | -1.2940 | 0.0000 | 0.0000 |
| EFCC1 | 2.6719 | 0.9355 | -1.5141 | 0.0000 | 0.0000 |
| PDRG1 | 7.3872 | 17.2428 | 1.2229 | 0.0000 | 0.0000 |
| PDE4D | 5.3550 | 1.3399 | -1.9988 | 0.0000 | 0.0000 |
| TMEM233 | 0.5604 | 0.2546 | -1.1386 | 0.0000 | 0.0000 |
| GAL | 0.1703 | 3.3687 | 4.3063 | 0.0029 | 0.0060 |
| PXYLP1 | 1.6264 | 4.3108 | 1.4063 | 0.0000 | 0.0000 |
| C10orf143 | 0.9504 | 0.4297 | -1.1452 | 0.0000 | 0.0000 |
| BRICD5 | 1.0087 | 2.5502 | 1.3381 | 0.0000 | 0.0000 |
| KLHDC1 | 1.2947 | 0.5432 | -1.2530 | 0.0000 | 0.0000 |
| TMEM160 | 7.3442 | 15.7190 | 1.0978 | 0.0000 | 0.0000 |
| ZNF681 | 0.5476 | 1.5058 | 1.4593 | 0.0000 | 0.0001 |
| KLRG2 | 0.3188 | 0.8026 | 1.3320 | 0.0081 | 0.0145 |
| ABRACL | 21.7195 | 47.7963 | 1.1379 | 0.0000 | 0.0000 |
| PPP1R14A | 44.8853 | 6.2627 | -2.8414 | 0.0000 | 0.0000 |
| PRR19 | 0.3463 | 1.3232 | 1.9338 | 0.0000 | 0.0000 |
| DONSON | 2.3778 | 7.3798 | 1.6339 | 0.0000 | 0.0000 |
| RAB26 | 0.4984 | 1.3304 | 1.4165 | 0.0300 | 0.0457 |
| ZNF138 | 2.1140 | 4.2385 | 1.0036 | 0.0000 | 0.0000 |
| PCP4 | 154.4232 | 10.7502 | -3.8445 | 0.0000 | 0.0000 |
| CD200 | 10.2314 | 3.5576 | -1.5240 | 0.0000 | 0.0000 |
| EVA1C | 8.9479 | 3.0294 | -1.5625 | 0.0000 | 0.0000 |
| HSD17B3 | 0.1657 | 0.7177 | 2.1149 | 0.0129 | 0.0218 |
| LTO1 | 1.7672 | 4.8367 | 1.4525 | 0.0000 | 0.0001 |
| SPP1 | 6.1791 | 145.6730 | 4.5592 | 0.0000 | 0.0000 |
| ZCWPW2 | 0.4755 | 0.1876 | -1.3416 | 0.0000 | 0.0000 |
| ZNF738 | 1.0912 | 3.4060 | 1.6422 | 0.0000 | 0.0000 |
| C3orf70 | 6.0094 | 1.0902 | -2.4627 | 0.0000 | 0.0000 |
| FOXN3 | 12.0990 | 4.6866 | -1.3683 | 0.0000 | 0.0000 |
| TBL1X | 10.8981 | 5.1295 | -1.0872 | 0.0000 | 0.0000 |
| NFIX | 23.6446 | 6.3314 | -1.9009 | 0.0000 | 0.0000 |
| EPN3 | 7.7503 | 17.3530 | 1.1629 | 0.0002 | 0.0007 |
| NPW | 0.0937 | 0.4805 | 2.3586 | 0.0001 | 0.0003 |
| AL121845.3 | 0.1959 | 0.5117 | 1.3852 | 0.0000 | 0.0000 |
| GYPC | 16.5086 | 4.4520 | -1.8907 | 0.0000 | 0.0000 |
| RASSF8 | 4.6034 | 2.1029 | -1.1303 | 0.0002 | 0.0005 |
| ITGB3 | 1.9792 | 0.4984 | -1.9894 | 0.0001 | 0.0002 |
| CDC42BPA | 8.4570 | 3.3816 | -1.3225 | 0.0000 | 0.0001 |
| PHYHIP | 5.7684 | 1.0242 | -2.4936 | 0.0000 | 0.0000 |
| EPHX2 | 6.7183 | 2.4293 | -1.4675 | 0.0000 | 0.0000 |
| PLEKHF1 | 6.6063 | 15.5637 | 1.2363 | 0.0080 | 0.0144 |
| CDC6 | 1.8149 | 8.0871 | 2.1558 | 0.0000 | 0.0000 |
| RASL11A | 16.1381 | 4.3700 | -1.8848 | 0.0000 | 0.0000 |
| HIST4H4 | 0.2658 | 0.8545 | 1.6847 | 0.0002 | 0.0006 |
| GAS6 | 38.1098 | 18.0249 | -1.0802 | 0.0000 | 0.0000 |
| SLC2A12 | 1.4275 | 0.7058 | -1.0162 | 0.0024 | 0.0051 |
| SOWAHD | 0.3218 | 0.7261 | 1.1741 | 0.0000 | 0.0002 |
| CLDN14 | 0.0886 | 0.2426 | 1.4524 | 0.0035 | 0.0070 |
| CABP4 | 0.3336 | 1.4071 | 2.0763 | 0.0003 | 0.0007 |
| FAM162B | 3.1121 | 1.0229 | -1.6052 | 0.0000 | 0.0001 |
| MYOC | 10.2895 | 0.2669 | -5.2688 | 0.0000 | 0.0000 |
| LAIR2 | 0.1786 | 0.4676 | 1.3881 | 0.0167 | 0.0272 |
| DNA2 | 0.5973 | 2.1566 | 1.8523 | 0.0000 | 0.0000 |
| STMN1 | 14.8778 | 53.6038 | 1.8492 | 0.0000 | 0.0000 |
| KLHDC7B | 2.7759 | 40.3356 | 3.8610 | 0.0000 | 0.0000 |
| EFCAB10 | 0.1091 | 0.2529 | 1.2128 | 0.0000 | 0.0000 |
| TMC6 | 5.2332 | 13.8989 | 1.4092 | 0.0000 | 0.0000 |
| HOXB5 | 1.2598 | 4.7071 | 1.9016 | 0.0000 | 0.0001 |
| PDGFD | 7.6875 | 2.2772 | -1.7552 | 0.0000 | 0.0000 |
| DPYSL2 | 16.8033 | 6.7068 | -1.3251 | 0.0000 | 0.0000 |
| GPAT3 | 3.3936 | 1.1456 | -1.5668 | 0.0000 | 0.0000 |
| NFATC1 | 3.5367 | 1.3365 | -1.4040 | 0.0000 | 0.0000 |
| CCDC60 | 1.8450 | 0.5423 | -1.7664 | 0.0000 | 0.0000 |
| ZNF749 | 0.7266 | 1.4832 | 1.0294 | 0.0000 | 0.0000 |
| ZFP69B | 0.4221 | 1.1360 | 1.4283 | 0.0000 | 0.0000 |
| SSBP2 | 3.1793 | 1.3323 | -1.2548 | 0.0000 | 0.0000 |
| ZNF117 | 1.3054 | 5.2679 | 2.0127 | 0.0002 | 0.0005 |
| NEIL3 | 0.2847 | 1.4548 | 2.3534 | 0.0000 | 0.0000 |
| SPATC1L | 2.7808 | 6.0986 | 1.1330 | 0.0002 | 0.0006 |
| ZNF488 | 0.5385 | 3.5801 | 2.7331 | 0.0000 | 0.0001 |
| GPIHBP1 | 4.4876 | 0.6779 | -2.7269 | 0.0000 | 0.0000 |
| MEIG1 | 0.1108 | 0.2382 | 1.1046 | 0.0037 | 0.0073 |
| CA2 | 2.8966 | 15.4426 | 2.4145 | 0.0027 | 0.0056 |
| ZP3 | 1.0869 | 2.9218 | 1.4266 | 0.0000 | 0.0001 |
| RGS20 | 0.1333 | 1.1571 | 3.1178 | 0.0000 | 0.0000 |
| PLA2G2F | 2.9655 | 17.1990 | 2.5360 | 0.0020 | 0.0042 |
| TACR2 | 14.7255 | 0.8703 | -4.0806 | 0.0000 | 0.0000 |
| CENPK | 0.5670 | 2.5383 | 2.1624 | 0.0000 | 0.0000 |
| CYTL1 | 6.4332 | 1.9460 | -1.7250 | 0.0000 | 0.0000 |
| RGCC | 38.2915 | 15.9145 | -1.2667 | 0.0000 | 0.0001 |
| LRFN2 | 0.0826 | 0.4950 | 2.5827 | 0.0051 | 0.0098 |
| WNT9A | 4.9077 | 2.0646 | -1.2492 | 0.0000 | 0.0000 |
| SPRR2D | 6.6955 | 16.5240 | 1.3033 | 0.0029 | 0.0059 |
| OIP5 | 0.8639 | 3.8702 | 2.1635 | 0.0000 | 0.0000 |
| AHRR | 0.8568 | 0.4240 | -1.0151 | 0.0063 | 0.0116 |
| FHL1 | 117.2400 | 7.2412 | -4.0171 | 0.0000 | 0.0000 |
| TNFAIP8L3 | 8.2167 | 1.7888 | -2.1995 | 0.0000 | 0.0000 |
| ZNF90 | 0.8267 | 1.7267 | 1.0626 | 0.0266 | 0.0411 |
| HOXC13 | 0.2875 | 2.0354 | 2.8238 | 0.0000 | 0.0001 |
| ESPL1 | 1.7039 | 3.5334 | 1.0522 | 0.0000 | 0.0000 |
| ROCK2 | 12.0931 | 6.0368 | -1.0023 | 0.0005 | 0.0012 |
| FSCN1 | 29.9770 | 87.8281 | 1.5508 | 0.0001 | 0.0003 |
| HSPB6 | 243.5957 | 11.5029 | -4.4044 | 0.0000 | 0.0000 |
| RNASE2 | 0.4298 | 1.1783 | 1.4549 | 0.0170 | 0.0277 |
| RAB3B | 0.2232 | 0.6640 | 1.5730 | 0.0013 | 0.0029 |
| SEMA3E | 2.5009 | 0.5389 | -2.2144 | 0.0000 | 0.0000 |
| ZFHX4 | 1.5239 | 0.4439 | -1.7795 | 0.0000 | 0.0000 |
| CGB7 | 0.1700 | 0.4104 | 1.2718 | 0.0027 | 0.0055 |
| BRI3BP | 1.9094 | 5.0985 | 1.4169 | 0.0000 | 0.0000 |
| PRR9 | 0.1889 | 6.6497 | 5.1375 | 0.0012 | 0.0027 |
| TBX20 | 4.2935 | 0.2580 | -4.0568 | 0.0003 | 0.0008 |
| GPR155 | 2.1060 | 0.9972 | -1.0786 | 0.0000 | 0.0000 |
| C10orf55 | 0.2919 | 0.7977 | 1.4503 | 0.0025 | 0.0051 |
| KIF4A | 1.3483 | 7.5386 | 2.4832 | 0.0000 | 0.0000 |
| UCK2 | 3.4618 | 8.2901 | 1.2599 | 0.0000 | 0.0000 |
| RDH5 | 0.5636 | 0.2511 | -1.1665 | 0.0019 | 0.0041 |
| TROAP | 1.1502 | 8.0767 | 2.8119 | 0.0000 | 0.0000 |
| PAM | 19.3041 | 9.4311 | -1.0334 | 0.0000 | 0.0000 |
| MGAT4B | 12.6015 | 25.6512 | 1.0254 | 0.0000 | 0.0000 |
| CNNM1 | 0.7350 | 0.1996 | -1.8804 | 0.0000 | 0.0000 |
| PRAME | 0.0811 | 4.6013 | 5.8270 | 0.0275 | 0.0424 |
| RTBDN | 0.0725 | 0.2421 | 1.7390 | 0.0001 | 0.0002 |
| TMEM252 | 5.1713 | 0.1983 | -4.7050 | 0.0000 | 0.0000 |
| TICRR | 0.3882 | 1.6395 | 2.0784 | 0.0000 | 0.0000 |
| AHNAK2 | 8.5220 | 4.2488 | -1.0041 | 0.0029 | 0.0058 |
| JAM2 | 5.9447 | 1.0629 | -2.4835 | 0.0000 | 0.0000 |
| STEAP3 | 6.9182 | 16.1260 | 1.2209 | 0.0000 | 0.0001 |
| HES2 | 1.7829 | 6.6686 | 1.9032 | 0.0004 | 0.0011 |
| MKX | 2.1602 | 0.3127 | -2.7882 | 0.0000 | 0.0000 |
| SMC1B | 0.0999 | 0.7693 | 2.9445 | 0.0000 | 0.0000 |
| MMP1 | 9.0735 | 64.4516 | 2.8285 | 0.0006 | 0.0015 |
| VEPH1 | 0.0672 | 0.4557 | 2.7621 | 0.0005 | 0.0013 |
| MZT2A | 6.0241 | 12.9524 | 1.1044 | 0.0000 | 0.0000 |
| ADAM2 | 0.1004 | 0.2988 | 1.5732 | 0.0048 | 0.0092 |
| HSPA12B | 3.9343 | 1.8447 | -1.0928 | 0.0000 | 0.0000 |
| YDJC | 7.0453 | 21.8448 | 1.6326 | 0.0000 | 0.0000 |
| HIST1H4B | 0.0302 | 1.9671 | 6.0259 | 0.0000 | 0.0000 |
| HPD | 1.5244 | 0.5225 | -1.5447 | 0.0000 | 0.0001 |
| DRAXIN | 0.0348 | 0.2971 | 3.0950 | 0.0000 | 0.0000 |
| UTRN | 6.0934 | 2.7649 | -1.1400 | 0.0000 | 0.0000 |
| CLIC3 | 21.0978 | 50.4877 | 1.2588 | 0.0002 | 0.0007 |
| LRRC4 | 1.6447 | 0.6030 | -1.4476 | 0.0000 | 0.0001 |
| TRIP13 | 1.9853 | 9.6564 | 2.2821 | 0.0000 | 0.0000 |
| CPEB3 | 1.1911 | 0.5811 | -1.0355 | 0.0000 | 0.0000 |
| TMX4 | 14.7211 | 7.3097 | -1.0100 | 0.0000 | 0.0000 |
| FOXA3 | 0.1406 | 0.4973 | 1.8223 | 0.0034 | 0.0068 |
| TRIT1 | 7.8943 | 17.4966 | 1.1482 | 0.0000 | 0.0000 |
| AKAP6 | 1.9702 | 0.1863 | -3.4023 | 0.0000 | 0.0000 |
| TTC23L | 0.1132 | 0.3567 | 1.6561 | 0.0063 | 0.0117 |
| KIF3C | 2.1335 | 5.4269 | 1.3469 | 0.0000 | 0.0000 |
| MDK | 52.5790 | 159.9319 | 1.6049 | 0.0000 | 0.0000 |
| MSTO1 | 1.6017 | 3.2089 | 1.0025 | 0.0000 | 0.0000 |
| FILIP1L | 30.6159 | 6.1277 | -2.3209 | 0.0000 | 0.0000 |
| ADRA2A | 3.9337 | 1.2449 | -1.6599 | 0.0000 | 0.0000 |
| NLGN3 | 1.0056 | 0.4560 | -1.1409 | 0.0000 | 0.0000 |
| HOXC6 | 0.1234 | 1.1793 | 3.2568 | 0.0000 | 0.0000 |
| MEN1 | 6.0078 | 15.0390 | 1.3238 | 0.0000 | 0.0000 |
| HIST3H2BB | 0.1420 | 1.1251 | 2.9863 | 0.0000 | 0.0000 |
| SMAD9 | 3.0831 | 1.2167 | -1.3414 | 0.0000 | 0.0000 |
| HIST1H3H | 0.7802 | 6.7060 | 3.1036 | 0.0000 | 0.0000 |
| SMTN | 72.4963 | 9.2837 | -2.9651 | 0.0000 | 0.0000 |
| TNS2 | 16.9706 | 4.6566 | -1.8657 | 0.0000 | 0.0000 |
| HIC1 | 2.9029 | 1.2218 | -1.2484 | 0.0000 | 0.0000 |
| TAC1 | 1.5066 | 0.7170 | -1.0711 | 0.0000 | 0.0000 |
| RPRM | 3.1615 | 1.5418 | -1.0360 | 0.0001 | 0.0002 |
| SSC5D | 7.5285 | 1.9826 | -1.9249 | 0.0000 | 0.0000 |
| TCAP | 0.4779 | 1.7766 | 1.8945 | 0.0087 | 0.0155 |
| ADAMTS15 | 3.2162 | 1.3667 | -1.2347 | 0.0000 | 0.0000 |
| MT1F | 2.0509 | 4.5957 | 1.1640 | 0.0071 | 0.0130 |
| LRRC45 | 4.3980 | 12.9689 | 1.5601 | 0.0000 | 0.0000 |
| SLC31A2 | 0.4151 | 0.8480 | 1.0307 | 0.0084 | 0.0150 |
| TMEM190 | 0.1207 | 0.3213 | 1.4124 | 0.0001 | 0.0003 |
| CENPU | 2.5860 | 9.2928 | 1.8454 | 0.0000 | 0.0000 |
| CARHSP1 | 9.0429 | 18.2930 | 1.0164 | 0.0001 | 0.0003 |
| DHRS11 | 2.1428 | 4.7431 | 1.1463 | 0.0000 | 0.0001 |
| TRPM2 | 1.2427 | 3.5868 | 1.5293 | 0.0000 | 0.0001 |
| FRMD5 | 0.1182 | 0.5061 | 2.0985 | 0.0002 | 0.0006 |
| ATAD2 | 2.8867 | 9.0137 | 1.6427 | 0.0000 | 0.0000 |
| KRT31 | 0.2569 | 4.0152 | 3.9664 | 0.0086 | 0.0154 |
| RCCD1 | 2.0196 | 4.1307 | 1.0324 | 0.0000 | 0.0000 |
| ZNF69 | 1.3425 | 2.7161 | 1.0166 | 0.0052 | 0.0099 |
| SLC2A6 | 2.3798 | 7.1613 | 1.5894 | 0.0001 | 0.0002 |
| ZC3HAV1L | 0.8966 | 2.2725 | 1.3417 | 0.0000 | 0.0001 |
| LRFN4 | 4.5906 | 9.8294 | 1.0984 | 0.0036 | 0.0071 |
| HIST1H1D | 0.0696 | 3.1560 | 5.5021 | 0.0000 | 0.0000 |
| ERAL1 | 16.8362 | 33.9120 | 1.0102 | 0.0000 | 0.0000 |
| C4orf48 | 2.8244 | 15.3004 | 2.4375 | 0.0000 | 0.0000 |
| KCNAB3 | 0.3067 | 0.6937 | 1.1776 | 0.0004 | 0.0010 |
| FAM111B | 0.9063 | 4.8316 | 2.4145 | 0.0000 | 0.0000 |
| SCD | 21.8772 | 91.0852 | 2.0578 | 0.0000 | 0.0000 |
| CFP | 2.0223 | 0.6046 | -1.7419 | 0.0000 | 0.0000 |
| KLK2 | 0.0063 | 0.5849 | 6.5315 | 0.0055 | 0.0103 |
| RHPN1 | 2.3360 | 10.3060 | 2.1414 | 0.0000 | 0.0000 |
| HBA2 | 16.7692 | 8.1222 | -1.0459 | 0.0000 | 0.0000 |
| MAGEA9B | 0.1284 | 1.2226 | 3.2507 | 0.0010 | 0.0022 |
| MT1A | 16.4611 | 3.4959 | -2.2353 | 0.0000 | 0.0000 |
| RNF213 | 4.9494 | 10.9644 | 1.1475 | 0.0000 | 0.0001 |
| SAC3D1 | 3.4827 | 10.9361 | 1.6508 | 0.0000 | 0.0000 |
| AC004233.2 | 0.0115 | 0.3248 | 4.8186 | 0.0000 | 0.0000 |
| PDZK1IP1 | 7.6390 | 28.6322 | 1.9062 | 0.0055 | 0.0103 |
| KRTAP4-1 | 0.1198 | 0.7128 | 2.5722 | 0.0000 | 0.0000 |
| LDLRAD4 | 1.6088 | 0.7407 | -1.1190 | 0.0000 | 0.0000 |
| TMPRSS6 | 0.0786 | 0.2764 | 1.8145 | 0.0032 | 0.0064 |
| ZBED2 | 0.6881 | 3.0481 | 2.1471 | 0.0033 | 0.0066 |
| RSPO3 | 3.9929 | 0.9683 | -2.0439 | 0.0000 | 0.0000 |
| MNS1 | 0.9088 | 2.0647 | 1.1839 | 0.0008 | 0.0018 |
| PDSS1 | 2.0775 | 4.2131 | 1.0201 | 0.0000 | 0.0000 |
| MMP15 | 8.0235 | 16.4732 | 1.0378 | 0.0001 | 0.0004 |
| ZNF714 | 0.5917 | 1.2269 | 1.0521 | 0.0010 | 0.0024 |
| EIF2AK1 | 17.0077 | 37.3601 | 1.1353 | 0.0000 | 0.0000 |
| SLC3A2 | 21.4342 | 45.0162 | 1.0705 | 0.0000 | 0.0000 |
| MEST | 2.7268 | 16.8144 | 2.6244 | 0.0000 | 0.0000 |
| PIGW | 2.4667 | 5.1063 | 1.0497 | 0.0000 | 0.0000 |
| TAP2 | 4.2967 | 9.8747 | 1.2005 | 0.0004 | 0.0011 |
| SLC35D3 | 0.0161 | 0.2718 | 4.0772 | 0.0081 | 0.0145 |
| PSORS1C2 | 0.5885 | 20.8434 | 5.1465 | 0.0006 | 0.0015 |
| CACNB1 | 0.8562 | 1.9479 | 1.1860 | 0.0000 | 0.0001 |
| MYZAP | 15.5024 | 3.4324 | -2.1752 | 0.0000 | 0.0000 |
| MKI67 | 2.3224 | 10.8096 | 2.2186 | 0.0000 | 0.0000 |
| MYRFL | 0.0259 | 0.2592 | 3.3232 | 0.0000 | 0.0000 |
| KCNA5 | 1.3256 | 0.1582 | -3.0667 | 0.0000 | 0.0000 |
| B3GNT4 | 0.1117 | 0.5646 | 2.3372 | 0.0000 | 0.0000 |
| FGF10 | 2.1657 | 0.1839 | -3.5578 | 0.0000 | 0.0000 |
| PLIN1 | 2.1454 | 0.8668 | -1.3075 | 0.0000 | 0.0000 |
| RCSD1 | 4.8215 | 2.0486 | -1.2348 | 0.0000 | 0.0000 |
| CACNA1C | 3.4772 | 0.5608 | -2.6325 | 0.0000 | 0.0000 |
| B3GALT2 | 0.6842 | 0.1792 | -1.9327 | 0.0000 | 0.0000 |
| HABP2 | 0.0083 | 0.2907 | 5.1281 | 0.0054 | 0.0102 |
| HIST1H3B | 0.0583 | 5.6566 | 6.6000 | 0.0000 | 0.0000 |
| HIST1H2AM | 0.1369 | 1.5827 | 3.5313 | 0.0000 | 0.0000 |
| FBN1 | 11.8966 | 4.5259 | -1.3943 | 0.0007 | 0.0017 |
| HIST1H2BK | 16.3951 | 73.5660 | 2.1658 | 0.0000 | 0.0000 |
| SCN8A | 0.0767 | 0.2888 | 1.9126 | 0.0026 | 0.0053 |
| ADAMTSL3 | 3.0764 | 0.2034 | -3.9190 | 0.0000 | 0.0000 |
| ERVV-2 | 0.0013 | 0.4899 | 8.5715 | 0.0000 | 0.0000 |
| THOP1 | 3.6677 | 7.7074 | 1.0714 | 0.0000 | 0.0000 |
| PNRC1 | 56.1506 | 24.1901 | -1.2149 | 0.0000 | 0.0000 |
| CCNF | 2.0177 | 5.7062 | 1.4998 | 0.0000 | 0.0000 |
| SOX7 | 7.1060 | 3.1441 | -1.1764 | 0.0000 | 0.0001 |
| NCEH1 | 3.7851 | 7.6925 | 1.0231 | 0.0002 | 0.0006 |
| FKBPL | 3.8874 | 8.9173 | 1.1978 | 0.0000 | 0.0000 |
| HIF3A | 3.5564 | 0.5004 | -2.8294 | 0.0000 | 0.0000 |
| PRKAG2 | 7.6547 | 2.8975 | -1.4015 | 0.0000 | 0.0000 |
| MMP13 | 1.1296 | 15.7709 | 3.8034 | 0.0002 | 0.0006 |
| SGCE | 15.7474 | 6.4319 | -1.2918 | 0.0000 | 0.0000 |
| PROSER2 | 2.0507 | 6.8818 | 1.7467 | 0.0000 | 0.0000 |
| PRAC1 | 8.2303 | 1.9957 | -2.0441 | 0.0000 | 0.0000 |
| KRT6A | 160.7322 | 415.7351 | 1.3710 | 0.0142 | 0.0237 |
| SIM2 | 0.4562 | 2.1259 | 2.2202 | 0.0000 | 0.0000 |
| MARCKSL1 | 60.0078 | 165.3327 | 1.4621 | 0.0000 | 0.0000 |
| BEND3 | 0.5685 | 1.3207 | 1.2161 | 0.0000 | 0.0000 |
| HPGDS | 1.8494 | 0.5185 | -1.8348 | 0.0000 | 0.0000 |
| COPG1 | 28.4995 | 58.5206 | 1.0380 | 0.0000 | 0.0000 |
| FOXE1 | 0.3313 | 1.0118 | 1.6105 | 0.0241 | 0.0377 |
| SYPL2 | 1.4877 | 0.3064 | -2.2793 | 0.0000 | 0.0000 |
| RACGAP1 | 4.4487 | 11.9761 | 1.4287 | 0.0000 | 0.0000 |
| LGALS3BP | 122.1989 | 247.1849 | 1.0164 | 0.0000 | 0.0001 |
| STARD8 | 2.4055 | 1.0327 | -1.2199 | 0.0000 | 0.0000 |
| LRRC73 | 0.2468 | 0.5905 | 1.2586 | 0.0004 | 0.0010 |
| ZFP2 | 0.8080 | 0.3325 | -1.2810 | 0.0000 | 0.0000 |
| CYGB | 25.8412 | 7.2833 | -1.8270 | 0.0000 | 0.0000 |
| LMCD1 | 12.7915 | 3.7551 | -1.7683 | 0.0000 | 0.0000 |
| VGF | 0.1298 | 1.3375 | 3.3655 | 0.0000 | 0.0000 |
| KRT16 | 76.8444 | 205.1485 | 1.4167 | 0.0050 | 0.0095 |
| PAQR4 | 1.2858 | 6.2665 | 2.2850 | 0.0000 | 0.0000 |
| PILRB | 0.3989 | 1.5403 | 1.9493 | 0.0000 | 0.0000 |
| PLEKHJ1 | 7.5337 | 15.8675 | 1.0746 | 0.0000 | 0.0000 |
| CNTD2 | 0.0887 | 0.6275 | 2.8232 | 0.0000 | 0.0000 |
| EYA1 | 0.9674 | 0.3125 | -1.6303 | 0.0000 | 0.0000 |
| FBXW9 | 3.4434 | 7.6632 | 1.1541 | 0.0000 | 0.0000 |
| NDC80 | 1.7441 | 7.5650 | 2.1168 | 0.0000 | 0.0000 |
| ZNF516 | 4.3756 | 2.1227 | -1.0436 | 0.0000 | 0.0000 |
| BIRC5 | 4.5570 | 19.4520 | 2.0938 | 0.0000 | 0.0000 |
| ACBD7 | 0.2862 | 0.6956 | 1.2811 | 0.0257 | 0.0399 |
| DNMT1 | 4.5150 | 10.8820 | 1.2691 | 0.0000 | 0.0000 |
| PTGER3 | 1.6240 | 0.4510 | -1.8482 | 0.0000 | 0.0000 |
| ZEB1 | 12.7741 | 2.1833 | -2.5486 | 0.0000 | 0.0000 |
| POLG2 | 1.6415 | 3.4552 | 1.0737 | 0.0000 | 0.0000 |
| DKC1 | 12.1422 | 25.0441 | 1.0444 | 0.0000 | 0.0000 |
| TRMU | 2.1382 | 4.5045 | 1.0750 | 0.0000 | 0.0000 |
| UBASH3B | 1.2232 | 3.0371 | 1.3120 | 0.0008 | 0.0019 |
| REELD1 | 0.4272 | 0.1911 | -1.1608 | 0.0013 | 0.0029 |
| DPT | 59.0380 | 6.2962 | -3.2291 | 0.0000 | 0.0000 |
| REEP2 | 3.8742 | 1.3744 | -1.4951 | 0.0000 | 0.0000 |
| CDC42EP2 | 16.8576 | 8.0827 | -1.0605 | 0.0000 | 0.0000 |
| SLC38A1 | 10.6460 | 21.5817 | 1.0195 | 0.0000 | 0.0000 |
| RCC2 | 26.2564 | 68.7750 | 1.3892 | 0.0000 | 0.0000 |
| DCAF12L2 | 0.6071 | 0.2021 | -1.5871 | 0.0007 | 0.0018 |
| HSPB8 | 154.5437 | 21.6582 | -2.8350 | 0.0000 | 0.0000 |
| MPP7 | 8.8143 | 4.3333 | -1.0244 | 0.0000 | 0.0000 |
| ERBB3 | 11.8720 | 25.3443 | 1.0941 | 0.0016 | 0.0034 |
| LRP8 | 0.4587 | 1.8331 | 1.9988 | 0.0000 | 0.0000 |
| COL4A6 | 16.7557 | 4.5465 | -1.8818 | 0.0000 | 0.0000 |
| ACAN | 0.1734 | 0.5387 | 1.6355 | 0.0046 | 0.0088 |
| IL34 | 4.6660 | 2.1647 | -1.1080 | 0.0000 | 0.0000 |
| PRDX4 | 22.8373 | 46.4072 | 1.0230 | 0.0000 | 0.0000 |
| DUSP8 | 4.3500 | 1.3566 | -1.6811 | 0.0000 | 0.0000 |
| HIST1H4A | 0.0217 | 0.9517 | 5.4538 | 0.0003 | 0.0007 |
| EXO1 | 0.6631 | 3.4993 | 2.3998 | 0.0000 | 0.0000 |
| DDR2 | 11.3594 | 2.0941 | -2.4395 | 0.0000 | 0.0000 |
| C2CD4D | 0.1774 | 0.3956 | 1.1569 | 0.0213 | 0.0339 |
| NHLRC1 | 0.9794 | 2.0016 | 1.0311 | 0.0012 | 0.0027 |
| CDKL1 | 0.6429 | 0.1983 | -1.6969 | 0.0000 | 0.0000 |
| CALD1 | 155.8522 | 22.2860 | -2.8060 | 0.0000 | 0.0001 |
| ARRDC4 | 10.7948 | 4.7526 | -1.1836 | 0.0000 | 0.0001 |
| PLPPR4 | 4.6778 | 0.4066 | -3.5241 | 0.0000 | 0.0000 |
| CENPH | 2.2740 | 6.8298 | 1.5866 | 0.0000 | 0.0000 |
| EGR2 | 12.4521 | 2.7461 | -2.1809 | 0.0000 | 0.0000 |
| CPA6 | 1.4429 | 0.3301 | -2.1278 | 0.0004 | 0.0010 |
| PDE7B | 3.3734 | 1.1169 | -1.5948 | 0.0000 | 0.0000 |
| APOA2 | 0.1417 | 46.4837 | 8.3582 | 0.0080 | 0.0144 |
| REEP1 | 7.7684 | 0.6958 | -3.4809 | 0.0000 | 0.0000 |
| SYT11 | 7.5775 | 2.6673 | -1.5063 | 0.0000 | 0.0000 |
| INHBA | 0.8995 | 5.7427 | 2.6745 | 0.0003 | 0.0008 |
| ORC1 | 0.7733 | 3.7467 | 2.2766 | 0.0000 | 0.0000 |
| OGN | 23.3234 | 1.1981 | -4.2829 | 0.0000 | 0.0000 |
| DBF4B | 0.7342 | 1.9569 | 1.4144 | 0.0000 | 0.0000 |
| LRRC69 | 0.2025 | 0.5204 | 1.3616 | 0.0001 | 0.0003 |
| GOLGA8B | 1.3349 | 3.0829 | 1.2076 | 0.0006 | 0.0015 |
| TMEM88 | 9.9773 | 3.7814 | -1.3997 | 0.0000 | 0.0000 |
| AGTR1 | 3.8903 | 0.4573 | -3.0886 | 0.0000 | 0.0000 |
| TMEM92 | 0.8735 | 2.1023 | 1.2670 | 0.0192 | 0.0308 |
| LATS2 | 9.4811 | 3.6335 | -1.3837 | 0.0000 | 0.0000 |
| FOXS1 | 0.8493 | 1.9779 | 1.2196 | 0.0008 | 0.0018 |
| HIST1H4E | 0.2230 | 7.3456 | 5.0418 | 0.0000 | 0.0000 |
| ZBTB16 | 2.2809 | 0.2235 | -3.3514 | 0.0000 | 0.0000 |
| TBC1D7 | 1.0769 | 2.7192 | 1.3363 | 0.0000 | 0.0000 |
| MAP6D1 | 0.8129 | 1.9794 | 1.2839 | 0.0000 | 0.0000 |
| ATG9B | 0.4173 | 1.3510 | 1.6948 | 0.0009 | 0.0021 |
| ARG2 | 10.9872 | 3.8988 | -1.4947 | 0.0081 | 0.0145 |
| KLF15 | 3.9214 | 1.8339 | -1.0965 | 0.0001 | 0.0002 |
| C4orf47 | 0.2346 | 0.5541 | 1.2400 | 0.0053 | 0.0100 |
| TRIM59 | 0.5854 | 1.5590 | 1.4133 | 0.0000 | 0.0000 |
| TSTA3 | 15.2163 | 34.6151 | 1.1858 | 0.0000 | 0.0000 |
| MICB | 0.8945 | 3.6111 | 2.0133 | 0.0000 | 0.0000 |
| WDR34 | 14.9313 | 41.9881 | 1.4916 | 0.0000 | 0.0000 |
| PELI2 | 5.2836 | 1.2992 | -2.0239 | 0.0000 | 0.0000 |
| TFF2 | 0.9949 | 6.6515 | 2.7411 | 0.0277 | 0.0427 |
| MEIS1 | 5.1611 | 1.7770 | -1.5383 | 0.0000 | 0.0000 |
| MARVELD3 | 1.3711 | 3.6765 | 1.4230 | 0.0000 | 0.0000 |
| CEMIP | 0.8517 | 3.2289 | 1.9227 | 0.0004 | 0.0011 |
| STUM | 3.7744 | 0.3279 | -3.5251 | 0.0000 | 0.0000 |
| C11orf96 | 109.7391 | 13.7758 | -2.9939 | 0.0000 | 0.0000 |
| SPECC1 | 0.8797 | 2.2779 | 1.3726 | 0.0004 | 0.0009 |
| LINGO1 | 0.4313 | 1.1456 | 1.4095 | 0.0002 | 0.0007 |
| CD55 | 91.0676 | 26.1094 | -1.8024 | 0.0011 | 0.0026 |
| FBXL7 | 6.8347 | 1.3582 | -2.3312 | 0.0000 | 0.0000 |
| KPNA2 | 17.7960 | 57.6377 | 1.6955 | 0.0000 | 0.0000 |
| FNBP1 | 29.5032 | 6.9107 | -2.0940 | 0.0000 | 0.0000 |
| SREBF1 | 10.5688 | 26.2263 | 1.3112 | 0.0000 | 0.0000 |
| F10 | 7.1845 | 0.6063 | -3.5669 | 0.0000 | 0.0000 |
| DRICH1 | 0.1019 | 0.2403 | 1.2371 | 0.0019 | 0.0041 |
| POC1A | 3.2758 | 9.2375 | 1.4956 | 0.0000 | 0.0000 |
| MAGEA6 | 1.3267 | 9.0927 | 2.7768 | 0.0061 | 0.0113 |
| BLK | 1.5519 | 0.5198 | -1.5779 | 0.0139 | 0.0232 |
| CMTM1 | 0.2401 | 0.6471 | 1.4304 | 0.0000 | 0.0000 |
| ITGA3 | 22.3165 | 52.0082 | 1.2206 | 0.0043 | 0.0083 |
| SAMD15 | 0.1559 | 0.5170 | 1.7292 | 0.0000 | 0.0000 |
| NAMPT | 37.2041 | 16.8930 | -1.1390 | 0.0077 | 0.0138 |
| CALR | 192.3598 | 452.3841 | 1.2337 | 0.0000 | 0.0000 |
| TEX29 | 0.2191 | 0.6799 | 1.6338 | 0.0003 | 0.0009 |
| RASSF7 | 12.0470 | 28.8839 | 1.2616 | 0.0000 | 0.0000 |
| SHMT2 | 10.7724 | 30.5171 | 1.5023 | 0.0000 | 0.0000 |
| ARHGEF25 | 19.2999 | 4.3482 | -2.1501 | 0.0000 | 0.0000 |
| FCMR | 6.8629 | 3.1898 | -1.1053 | 0.0067 | 0.0124 |
| SFRP2 | 189.2179 | 67.5484 | -1.4861 | 0.0000 | 0.0000 |
| HSPB9 | 0.1146 | 0.2890 | 1.3342 | 0.0001 | 0.0003 |
| ZMYND19 | 6.5552 | 13.8537 | 1.0796 | 0.0000 | 0.0000 |
| PHEX | 0.1238 | 0.3764 | 1.6037 | 0.0276 | 0.0425 |
| LEPR | 2.5890 | 0.6785 | -1.9319 | 0.0000 | 0.0000 |
| BMF | 2.6851 | 5.4774 | 1.0285 | 0.0020 | 0.0042 |
| PHYHD1 | 5.3783 | 2.2931 | -1.2298 | 0.0000 | 0.0000 |
| C9orf84 | 0.0714 | 0.2859 | 2.0008 | 0.0019 | 0.0040 |
| SH3GL3 | 0.7903 | 0.3140 | -1.3315 | 0.0002 | 0.0005 |
| ANTXR2 | 21.6256 | 4.7250 | -2.1944 | 0.0000 | 0.0000 |
| SLC22A11 | 0.0063 | 0.2370 | 5.2260 | 0.0073 | 0.0133 |
| P2RX1 | 26.4405 | 1.5140 | -4.1263 | 0.0000 | 0.0000 |
| CEP131 | 3.7647 | 10.1336 | 1.4285 | 0.0000 | 0.0000 |
| KRT6B | 9.6373 | 76.8158 | 2.9947 | 0.0089 | 0.0158 |
| PRR7 | 1.1922 | 2.8562 | 1.2604 | 0.0000 | 0.0000 |
| C9orf116 | 1.0193 | 2.6993 | 1.4050 | 0.0000 | 0.0000 |
| TMEM97 | 23.8314 | 56.9361 | 1.2565 | 0.0098 | 0.0171 |
| MOV10 | 6.8193 | 13.9535 | 1.0329 | 0.0000 | 0.0000 |
| WISP1 | 0.6491 | 3.2128 | 2.3072 | 0.0231 | 0.0363 |
| MCM6 | 7.7295 | 17.2912 | 1.1616 | 0.0000 | 0.0000 |
| NAPSA | 0.1472 | 0.3423 | 1.2175 | 0.0001 | 0.0003 |
| SLC9A7 | 1.2489 | 2.8166 | 1.1733 | 0.0004 | 0.0011 |
| IRF7 | 7.3771 | 17.0609 | 1.2096 | 0.0001 | 0.0003 |
| TACC3 | 4.2746 | 17.0858 | 1.9990 | 0.0000 | 0.0000 |
| CKS2 | 19.8388 | 76.3822 | 1.9449 | 0.0000 | 0.0000 |
| NPTX1 | 0.7640 | 0.2838 | -1.4288 | 0.0000 | 0.0000 |
| KIF20B | 1.0850 | 2.9178 | 1.4272 | 0.0000 | 0.0000 |
| AHCY | 28.8212 | 58.1483 | 1.0126 | 0.0000 | 0.0000 |
| EBP | 13.6190 | 31.0525 | 1.1891 | 0.0000 | 0.0000 |
| GNAL | 4.7619 | 0.7821 | -2.6062 | 0.0000 | 0.0000 |
| SLC25A39 | 28.4913 | 64.0183 | 1.1680 | 0.0000 | 0.0000 |
| ANO1 | 4.6792 | 13.1392 | 1.4895 | 0.0054 | 0.0102 |
| PRSS8 | 7.1417 | 33.9070 | 2.2472 | 0.0000 | 0.0000 |
| KLF9 | 29.1047 | 5.4698 | -2.4117 | 0.0000 | 0.0000 |
| NFIA | 10.3302 | 3.1289 | -1.7231 | 0.0000 | 0.0000 |
| GPC2 | 0.1988 | 1.8368 | 3.2077 | 0.0000 | 0.0000 |
| ACOX2 | 3.0021 | 0.5556 | -2.4339 | 0.0000 | 0.0000 |
| TTLL7 | 3.0899 | 0.8148 | -1.9231 | 0.0000 | 0.0000 |
| COL21A1 | 2.3758 | 0.5891 | -2.0117 | 0.0000 | 0.0000 |
| OAS2 | 7.4221 | 19.2880 | 1.3778 | 0.0019 | 0.0041 |
| HIST1H2AB | 0.0337 | 1.0637 | 4.9810 | 0.0001 | 0.0002 |
| S100B | 5.8039 | 2.7568 | -1.0740 | 0.0000 | 0.0000 |
| TRPS1 | 2.5111 | 1.1141 | -1.1725 | 0.0000 | 0.0000 |
| PPP1R1A | 2.1842 | 0.2963 | -2.8819 | 0.0000 | 0.0000 |
| RUNX1T1 | 1.0079 | 0.2000 | -2.3335 | 0.0000 | 0.0000 |
| SLC25A4 | 14.8627 | 5.6572 | -1.3935 | 0.0000 | 0.0000 |
| GPR15 | 1.8660 | 0.3578 | -2.3827 | 0.0001 | 0.0003 |
| KLHL13 | 2.5566 | 0.6733 | -1.9249 | 0.0000 | 0.0000 |
| DHCR24 | 34.9200 | 78.6334 | 1.1711 | 0.0001 | 0.0003 |
| CHKB-CPT1B | 0.2070 | 0.4307 | 1.0575 | 0.0020 | 0.0043 |
| TDRD10 | 0.6080 | 0.2302 | -1.4009 | 0.0000 | 0.0000 |
| DIRAS1 | 2.4487 | 0.9139 | -1.4219 | 0.0000 | 0.0000 |
| MAPRE2 | 10.5893 | 4.4085 | -1.2642 | 0.0000 | 0.0000 |
| SGCB | 15.9736 | 6.1509 | -1.3768 | 0.0001 | 0.0002 |
| MS4A2 | 1.2765 | 0.2904 | -2.1362 | 0.0000 | 0.0000 |
| FAM183A | 1.8343 | 4.1208 | 1.1677 | 0.0121 | 0.0206 |
| ANGPTL1 | 7.1428 | 1.0963 | -2.7038 | 0.0000 | 0.0000 |
| KIAA1024 | 0.0759 | 0.2447 | 1.6893 | 0.0000 | 0.0000 |
| PCSK1N | 0.3051 | 3.2144 | 3.3974 | 0.0007 | 0.0018 |
| TPSD1 | 3.2470 | 0.7048 | -2.2039 | 0.0000 | 0.0000 |
| TGFBR2 | 41.4140 | 18.2128 | -1.1852 | 0.0000 | 0.0000 |
| CC2D2A | 3.8151 | 1.4245 | -1.4213 | 0.0000 | 0.0000 |
| GNG3 | 0.1240 | 0.4144 | 1.7404 | 0.0000 | 0.0000 |
| XAF1 | 0.9389 | 2.2765 | 1.2778 | 0.0284 | 0.0437 |
| E2F2 | 0.7101 | 2.5195 | 1.8270 | 0.0000 | 0.0000 |
| ANKRD65 | 9.8908 | 28.2940 | 1.5163 | 0.0029 | 0.0060 |
| FANCE | 2.9667 | 6.3136 | 1.0896 | 0.0000 | 0.0000 |
| SLC1A6 | 0.0637 | 5.4140 | 6.4094 | 0.0000 | 0.0001 |
| ATP5MF | 28.7551 | 59.7546 | 1.0552 | 0.0000 | 0.0000 |
| DES | 2904.3624 | 172.0796 | -4.0771 | 0.0000 | 0.0000 |
| CDCA5 | 2.6745 | 11.4272 | 2.0951 | 0.0000 | 0.0000 |
| ANXA6 | 70.7691 | 17.1612 | -2.0440 | 0.0000 | 0.0000 |
| TENT5C | 6.9694 | 2.6117 | -1.4160 | 0.0000 | 0.0001 |
| NFATC4 | 8.3862 | 3.2332 | -1.3750 | 0.0000 | 0.0000 |
| CLEC7A | 1.2430 | 3.1189 | 1.3272 | 0.0286 | 0.0439 |
| CLDN5 | 20.1227 | 3.8321 | -2.3926 | 0.0000 | 0.0000 |
| B4GALNT4 | 0.7317 | 5.3382 | 2.8671 | 0.0033 | 0.0066 |
| SETBP1 | 3.1071 | 0.9233 | -1.7507 | 0.0000 | 0.0000 |
| EFR3B | 0.4574 | 0.1984 | -1.2046 | 0.0012 | 0.0027 |
| TMPRSS3 | 0.2725 | 1.6031 | 2.5568 | 0.0139 | 0.0232 |
| CBX6 | 16.6002 | 7.9182 | -1.0680 | 0.0000 | 0.0001 |
| JAZF1 | 8.4468 | 2.4625 | -1.7783 | 0.0000 | 0.0000 |
| SLC19A1 | 0.9529 | 3.4412 | 1.8525 | 0.0000 | 0.0000 |
| NKX2-8 | 0.0964 | 0.7578 | 2.9753 | 0.0010 | 0.0023 |
| XPOT | 7.2486 | 14.9599 | 1.0453 | 0.0000 | 0.0000 |
| ACOT4 | 0.7783 | 2.6104 | 1.7458 | 0.0000 | 0.0000 |
| FAM43A | 12.4199 | 3.8548 | -1.6879 | 0.0000 | 0.0000 |
| THEM6 | 15.2059 | 33.5882 | 1.1433 | 0.0006 | 0.0015 |
| PDE1C | 1.8086 | 0.1692 | -3.4181 | 0.0000 | 0.0000 |
| BOLA1 | 6.0239 | 13.7807 | 1.1939 | 0.0000 | 0.0000 |
| MTURN | 14.4603 | 4.3096 | -1.7465 | 0.0000 | 0.0000 |
| CAVIN4 | 0.1255 | 0.3518 | 1.4871 | 0.0000 | 0.0001 |
| LRRK2 | 1.3893 | 0.3130 | -2.1501 | 0.0000 | 0.0000 |
| PNMA1 | 30.0578 | 12.5372 | -1.2615 | 0.0002 | 0.0005 |
| ITPKB | 19.6798 | 5.4762 | -1.8455 | 0.0000 | 0.0000 |
| PER1 | 26.8116 | 7.0710 | -1.9229 | 0.0000 | 0.0000 |
| WISP2 | 7.1703 | 1.5171 | -2.2407 | 0.0000 | 0.0000 |
| FBXO6 | 5.4693 | 13.3509 | 1.2875 | 0.0000 | 0.0000 |
| RHOJ | 7.2028 | 2.2307 | -1.6911 | 0.0000 | 0.0000 |
| ANO5 | 1.4769 | 0.3251 | -2.1835 | 0.0000 | 0.0000 |
| CCNA2 | 3.3579 | 13.9426 | 2.0539 | 0.0000 | 0.0000 |
| SSTR1 | 0.9510 | 0.2277 | -2.0625 | 0.0000 | 0.0000 |
| DLL3 | 0.0493 | 0.5850 | 3.5693 | 0.0001 | 0.0003 |
| IER5L | 2.4569 | 11.2036 | 2.1890 | 0.0000 | 0.0000 |
| RELL2 | 0.4383 | 1.5075 | 1.7820 | 0.0000 | 0.0000 |
| TMEM74B | 0.4491 | 3.3687 | 2.9072 | 0.0000 | 0.0000 |
| OXCT2 | 0.1335 | 0.2898 | 1.1177 | 0.0010 | 0.0023 |
| AOC3 | 68.9734 | 7.8652 | -3.1325 | 0.0000 | 0.0000 |
| CAPN12 | 0.3076 | 0.8380 | 1.4457 | 0.0002 | 0.0005 |
| CDKAL1 | 3.2891 | 6.7916 | 1.0461 | 0.0020 | 0.0043 |
| C10orf95 | 0.2976 | 0.7338 | 1.3022 | 0.0000 | 0.0000 |
| RBPMS | 23.8898 | 8.4673 | -1.4964 | 0.0000 | 0.0000 |
| DCST2 | 0.2507 | 0.6114 | 1.2859 | 0.0004 | 0.0010 |
| TMEM108 | 1.5573 | 0.3598 | -2.1138 | 0.0000 | 0.0002 |
| DLGAP5 | 1.6483 | 7.7597 | 2.2350 | 0.0000 | 0.0000 |
| AOX1 | 6.0475 | 0.6081 | -3.3139 | 0.0000 | 0.0000 |
| CD34 | 16.3648 | 4.4018 | -1.8944 | 0.0000 | 0.0000 |
| C20orf204 | 0.1429 | 0.4312 | 1.5929 | 0.0000 | 0.0000 |
| TACC2 | 9.1027 | 2.8918 | -1.6544 | 0.0000 | 0.0000 |
| E2F5 | 0.8594 | 1.8933 | 1.1396 | 0.0000 | 0.0001 |
| LRRIQ4 | 0.0834 | 0.3960 | 2.2475 | 0.0004 | 0.0010 |
| TMEM139 | 1.1814 | 5.4151 | 2.1964 | 0.0021 | 0.0045 |
| SLC6A11 | 0.5169 | 1.6737 | 1.6951 | 0.0212 | 0.0337 |
| METTL24 | 1.7845 | 0.1785 | -3.3215 | 0.0000 | 0.0000 |
| DLX6 | 0.1490 | 1.0847 | 2.8638 | 0.0000 | 0.0001 |
| SELENOI | 3.6569 | 7.4664 | 1.0298 | 0.0000 | 0.0000 |
| FAM171A2 | 0.3007 | 1.8880 | 2.6506 | 0.0000 | 0.0000 |
| HIST2H3D | 0.0616 | 0.7100 | 3.5278 | 0.0000 | 0.0000 |
| TACC1 | 23.3678 | 9.5033 | -1.2980 | 0.0000 | 0.0000 |
| SLC25A10 | 5.3249 | 12.3353 | 1.2120 | 0.0001 | 0.0002 |
| SLC22A3 | 3.5065 | 1.3699 | -1.3560 | 0.0000 | 0.0000 |
| AGRN | 16.0839 | 49.7369 | 1.6287 | 0.0000 | 0.0000 |
| SAP25 | 0.1226 | 0.2740 | 1.1603 | 0.0004 | 0.0010 |
| LHX1 | 0.0126 | 0.3105 | 4.6285 | 0.0001 | 0.0002 |
| ZNF850 | 0.2748 | 0.6249 | 1.1851 | 0.0008 | 0.0020 |
| NFASC | 2.8877 | 0.5039 | -2.5188 | 0.0000 | 0.0000 |
| HILPDA | 7.1590 | 27.3019 | 1.9312 | 0.0000 | 0.0000 |
| LYG1 | 0.4575 | 1.1557 | 1.3368 | 0.0000 | 0.0001 |
| FOS | 917.6179 | 120.5713 | -2.9280 | 0.0000 | 0.0000 |
| GPRIN1 | 0.3545 | 2.6621 | 2.9087 | 0.0000 | 0.0000 |
| HIST1H4D | 0.1391 | 3.9475 | 4.8270 | 0.0000 | 0.0000 |
| GPR89B | 0.3915 | 0.8094 | 1.0478 | 0.0000 | 0.0000 |
| RHEBL1 | 0.5006 | 1.9833 | 1.9863 | 0.0000 | 0.0000 |
| FBXO30 | 4.3671 | 1.9620 | -1.1543 | 0.0001 | 0.0003 |
| MAPT | 1.5059 | 0.3064 | -2.2969 | 0.0000 | 0.0001 |
| PGF | 1.5224 | 4.4863 | 1.5592 | 0.0006 | 0.0015 |
| TYMP | 15.8677 | 52.5092 | 1.7265 | 0.0002 | 0.0005 |
| EGR1 | 571.8888 | 64.9063 | -3.1393 | 0.0000 | 0.0000 |
| BOP1 | 11.1273 | 28.4933 | 1.3565 | 0.0000 | 0.0000 |
| ITGA5 | 91.9836 | 20.4919 | -2.1663 | 0.0000 | 0.0000 |
| MYADM | 110.1005 | 24.1477 | -2.1889 | 0.0000 | 0.0000 |
| IL36G | 0.2103 | 2.5349 | 3.5912 | 0.0008 | 0.0020 |
| LAMA2 | 7.8871 | 1.6350 | -2.2702 | 0.0000 | 0.0000 |
| UHRF1 | 0.9601 | 5.7411 | 2.5801 | 0.0000 | 0.0000 |
| LMO3 | 7.7127 | 0.7456 | -3.3708 | 0.0000 | 0.0000 |
| CDKN1A | 211.6809 | 83.1583 | -1.3480 | 0.0003 | 0.0007 |
| ATXN1 | 3.4074 | 1.6473 | -1.0485 | 0.0000 | 0.0000 |
| PNMA2 | 0.8428 | 0.3253 | -1.3735 | 0.0000 | 0.0000 |
| FEN1 | 8.2633 | 26.3462 | 1.6728 | 0.0000 | 0.0000 |
| SLC11A1 | 0.6723 | 1.4505 | 1.1094 | 0.0028 | 0.0056 |
| ABCA12 | 0.3580 | 1.1154 | 1.6393 | 0.0003 | 0.0007 |
| FAM72D | 0.0907 | 0.6578 | 2.8579 | 0.0000 | 0.0000 |
| FOXF1 | 26.8626 | 3.7763 | -2.8306 | 0.0000 | 0.0000 |
| CASQ2 | 45.9501 | 2.1242 | -4.4351 | 0.0000 | 0.0000 |
| NUF2 | 1.1298 | 7.5836 | 2.7468 | 0.0000 | 0.0000 |
| FAM234B | 1.7902 | 3.6313 | 1.0203 | 0.0033 | 0.0066 |
| HAUS8 | 1.2299 | 2.5326 | 1.0421 | 0.0000 | 0.0000 |
| FEZF1 | 0.0537 | 0.4056 | 2.9157 | 0.0088 | 0.0155 |
| CEP126 | 1.3278 | 0.5266 | -1.3343 | 0.0000 | 0.0000 |
| SLC5A7 | 1.0851 | 0.4414 | -1.2976 | 0.0000 | 0.0001 |
| RAB19 | 0.3774 | 0.7768 | 1.0416 | 0.0019 | 0.0041 |
| BAAT | 0.0082 | 1.0764 | 7.0401 | 0.0125 | 0.0212 |
| CENPX | 15.8919 | 40.7281 | 1.3577 | 0.0000 | 0.0000 |
| ETV7 | 2.9596 | 7.4806 | 1.3378 | 0.0002 | 0.0005 |
| TCEAL1 | 27.8789 | 11.7035 | -1.2522 | 0.0000 | 0.0000 |
| BRINP1 | 0.5784 | 0.2354 | -1.2972 | 0.0028 | 0.0058 |
| EFNA3 | 1.3095 | 3.4072 | 1.3796 | 0.0000 | 0.0000 |
| PPY | 0.0154 | 0.6371 | 5.3715 | 0.0231 | 0.0363 |
| KNOP1 | 1.3201 | 3.1906 | 1.2732 | 0.0000 | 0.0000 |
| ECE2 | 0.7465 | 2.5792 | 1.7888 | 0.0000 | 0.0000 |
| TMPRSS13 | 1.0391 | 2.0890 | 1.0075 | 0.0130 | 0.0220 |
| HIC2 | 0.8980 | 3.6343 | 2.0169 | 0.0005 | 0.0013 |
| FADS3 | 2.1769 | 4.7790 | 1.1344 | 0.0171 | 0.0278 |
| HIST1H2AJ | 0.1312 | 0.7612 | 2.5361 | 0.0004 | 0.0010 |
| HSBP1L1 | 3.7841 | 8.7286 | 1.2058 | 0.0000 | 0.0000 |
| CPOX | 3.6672 | 7.4204 | 1.0168 | 0.0000 | 0.0000 |
| TPD52L1 | 9.2680 | 4.6135 | -1.0064 | 0.0072 | 0.0131 |
| C19orf73 | 0.6801 | 1.9396 | 1.5120 | 0.0000 | 0.0000 |
| SEMA4F | 0.9234 | 2.4486 | 1.4070 | 0.0000 | 0.0000 |
| LIPG | 0.3656 | 2.4791 | 2.7613 | 0.0000 | 0.0000 |
| PPM1N | 0.8740 | 3.2250 | 1.8836 | 0.0096 | 0.0168 |
| LRRC32 | 14.5676 | 7.1735 | -1.0220 | 0.0000 | 0.0001 |
| HJURP | 1.0685 | 6.8618 | 2.6830 | 0.0000 | 0.0000 |
| CDCA7 | 1.5499 | 6.2631 | 2.0147 | 0.0000 | 0.0000 |
| MAFF | 39.8896 | 11.2410 | -1.8272 | 0.0000 | 0.0000 |
| CX3CL1 | 17.5610 | 7.3065 | -1.2651 | 0.0000 | 0.0000 |
| MIIP | 6.9232 | 15.6000 | 1.1720 | 0.0000 | 0.0000 |
| WNT2 | 0.6604 | 2.6062 | 1.9805 | 0.0053 | 0.0100 |
| CRYGS | 0.4448 | 1.1508 | 1.3714 | 0.0000 | 0.0000 |
| KBTBD8 | 1.2115 | 0.5741 | -1.0775 | 0.0000 | 0.0001 |
| AREG | 72.7297 | 26.2203 | -1.4719 | 0.0024 | 0.0050 |
| CCNE2 | 0.3232 | 1.5451 | 2.2574 | 0.0000 | 0.0000 |
| MCM8 | 1.2066 | 2.8739 | 1.2521 | 0.0000 | 0.0000 |
| P3H1 | 3.7177 | 8.0994 | 1.1234 | 0.0000 | 0.0000 |
| SYNGR4 | 0.0273 | 0.3599 | 3.7186 | 0.0000 | 0.0000 |
| PPIL1 | 11.2810 | 22.7451 | 1.0117 | 0.0000 | 0.0000 |
| RNASEH2A | 7.7158 | 23.2304 | 1.5901 | 0.0000 | 0.0000 |
| ROR2 | 6.4192 | 2.3049 | -1.4777 | 0.0000 | 0.0000 |
| CA9 | 1.5887 | 20.4409 | 3.6855 | 0.0000 | 0.0001 |
| BARX1 | 0.0580 | 3.0653 | 5.7234 | 0.0000 | 0.0000 |
| NFE2L3 | 3.1458 | 10.3873 | 1.7233 | 0.0000 | 0.0000 |
| USP43 | 1.1111 | 2.2232 | 1.0006 | 0.0003 | 0.0008 |
| LACTB2 | 4.4743 | 9.1091 | 1.0257 | 0.0000 | 0.0000 |
| UGCG | 36.9939 | 15.3511 | -1.2689 | 0.0000 | 0.0001 |
| HIST1H2BJ | 0.6022 | 6.3501 | 3.3985 | 0.0000 | 0.0000 |
| CKS1B | 5.8314 | 17.4180 | 1.5787 | 0.0000 | 0.0000 |
| ANKLE1 | 0.1568 | 0.4590 | 1.5500 | 0.0009 | 0.0022 |
| LTBP4 | 51.4524 | 10.0829 | -2.3513 | 0.0000 | 0.0000 |
| AC005726.1 | 0.3628 | 0.9307 | 1.3592 | 0.0000 | 0.0002 |
| CFAP47 | 0.0208 | 0.2576 | 3.6294 | 0.0003 | 0.0008 |
| UNC5CL | 0.3174 | 0.9178 | 1.5319 | 0.0050 | 0.0095 |
| RAP1GAP | 2.8366 | 6.2163 | 1.1319 | 0.0016 | 0.0036 |
| SPDL1 | 1.5145 | 3.2312 | 1.0932 | 0.0000 | 0.0000 |
| OLFM4 | 196.8746 | 37.1952 | -2.4041 | 0.0128 | 0.0217 |
| LPAR1 | 10.3459 | 3.9072 | -1.4048 | 0.0000 | 0.0000 |
| HCAR2 | 3.6596 | 9.1661 | 1.3246 | 0.0059 | 0.0110 |
| ZNF610 | 0.5933 | 1.2257 | 1.0468 | 0.0097 | 0.0170 |
| DKK2 | 0.1144 | 0.6415 | 2.4869 | 0.0002 | 0.0006 |
| FANCF | 2.1080 | 4.3943 | 1.0598 | 0.0000 | 0.0000 |
| USP2 | 2.1246 | 0.6281 | -1.7582 | 0.0000 | 0.0000 |
| TMEM132A | 2.4224 | 12.7443 | 2.3953 | 0.0000 | 0.0000 |
| GAS7 | 7.1781 | 1.9063 | -1.9128 | 0.0000 | 0.0000 |
| FBXO32 | 28.8514 | 9.0871 | -1.6667 | 0.0011 | 0.0026 |
| TNFRSF25 | 2.3908 | 5.6834 | 1.2493 | 0.0000 | 0.0000 |
| TDRKH | 1.3803 | 3.8432 | 1.4774 | 0.0000 | 0.0000 |
| ADGRD1 | 4.1305 | 0.2987 | -3.7896 | 0.0000 | 0.0000 |
| DNAJB13 | 0.0718 | 0.4724 | 2.7179 | 0.0010 | 0.0024 |
| PARP9 | 4.7468 | 9.6653 | 1.0258 | 0.0004 | 0.0010 |
| MT1M | 9.7721 | 3.6351 | -1.4267 | 0.0000 | 0.0000 |
| WEE1 | 19.5926 | 6.4588 | -1.6010 | 0.0001 | 0.0003 |
| C20orf96 | 3.3502 | 7.1829 | 1.1003 | 0.0000 | 0.0001 |
| MAPK15 | 0.4188 | 3.1862 | 2.9275 | 0.0000 | 0.0000 |
| SPAG4 | 3.1009 | 10.6008 | 1.7734 | 0.0000 | 0.0000 |
| TLR2 | 1.8807 | 4.4794 | 1.2521 | 0.0047 | 0.0090 |
| MFSD3 | 5.3916 | 15.4588 | 1.5196 | 0.0000 | 0.0000 |
| FANCA | 0.5321 | 2.5247 | 2.2463 | 0.0000 | 0.0000 |
| BID | 5.6103 | 14.2318 | 1.3430 | 0.0000 | 0.0000 |
| SSC4D | 0.1768 | 0.7437 | 2.0725 | 0.0000 | 0.0000 |
| UNC13D | 3.7523 | 12.9350 | 1.7854 | 0.0000 | 0.0001 |
| PCLAF | 2.1287 | 9.0248 | 2.0839 | 0.0000 | 0.0000 |
| PTGER4 | 9.7291 | 4.8178 | -1.0139 | 0.0001 | 0.0004 |
| GPC6 | 4.9528 | 1.8322 | -1.4347 | 0.0000 | 0.0000 |
| CMTM4 | 2.3437 | 4.7176 | 1.0092 | 0.0007 | 0.0017 |
| PYGM | 8.7322 | 0.4266 | -4.3553 | 0.0000 | 0.0000 |
| SLC6A8 | 12.6007 | 31.8780 | 1.3391 | 0.0093 | 0.0165 |
| SYNPO2 | 129.0950 | 4.8752 | -4.7268 | 0.0000 | 0.0000 |
| DGCR6 | 0.2564 | 0.6190 | 1.2714 | 0.0185 | 0.0299 |
| ETS2 | 131.4235 | 53.1260 | -1.3067 | 0.0000 | 0.0002 |
| DUSP9 | 0.6913 | 3.8496 | 2.4773 | 0.0003 | 0.0007 |
| FLG | 0.5598 | 0.2538 | -1.1412 | 0.0198 | 0.0318 |
| IL37 | 0.0080 | 0.6767 | 6.4100 | 0.0000 | 0.0000 |
| FGF8 | 0.0246 | 0.7846 | 4.9948 | 0.0122 | 0.0207 |
| LDB2 | 4.5132 | 1.6490 | -1.4526 | 0.0000 | 0.0000 |
| SUSD2 | 1.0627 | 3.6408 | 1.7765 | 0.0089 | 0.0158 |
| HIST1H2BF | 0.1097 | 2.9852 | 4.7662 | 0.0000 | 0.0000 |
| MELK | 1.7971 | 10.6425 | 2.5661 | 0.0000 | 0.0000 |
| COL2A1 | 0.0081 | 0.8212 | 6.6609 | 0.0000 | 0.0001 |
| ITGB1BP2 | 3.4347 | 0.6807 | -2.3352 | 0.0000 | 0.0000 |
| DSEL | 0.7815 | 0.3665 | -1.0925 | 0.0000 | 0.0000 |
| MST1 | 0.8478 | 2.0799 | 1.2947 | 0.0003 | 0.0007 |
| KRT18 | 113.8939 | 349.9366 | 1.6194 | 0.0000 | 0.0001 |
| SLC35B1 | 4.9037 | 10.0785 | 1.0393 | 0.0000 | 0.0000 |
| RTEL1-TNFRSF6B | 0.1888 | 0.3956 | 1.0671 | 0.0000 | 0.0001 |
| HMGB2 | 24.8188 | 60.3645 | 1.2823 | 0.0000 | 0.0000 |
| GABRD | 0.3453 | 1.1975 | 1.7942 | 0.0000 | 0.0000 |
| HRC | 1.0425 | 0.4966 | -1.0699 | 0.0000 | 0.0000 |
| C8G | 0.3613 | 0.9077 | 1.3292 | 0.0000 | 0.0000 |
| PLCL1 | 1.3682 | 0.3016 | -2.1813 | 0.0000 | 0.0000 |
| TRPC3 | 0.9308 | 0.1665 | -2.4827 | 0.0000 | 0.0000 |
| GPR137C | 0.1440 | 0.4010 | 1.4773 | 0.0045 | 0.0087 |
| PROC | 0.3070 | 1.0326 | 1.7498 | 0.0008 | 0.0020 |
| PRELP | 42.8381 | 3.9612 | -3.4349 | 0.0000 | 0.0000 |
| KNL1 | 0.5268 | 2.0753 | 1.9780 | 0.0000 | 0.0000 |
| FGL2 | 45.4346 | 4.8770 | -3.2197 | 0.0000 | 0.0000 |
| TRPC4 | 0.6911 | 0.2267 | -1.6083 | 0.0000 | 0.0000 |
| GAST | 0.0199 | 0.3090 | 3.9590 | 0.0035 | 0.0069 |
| GRP | 0.3550 | 1.0004 | 1.4948 | 0.0049 | 0.0093 |
| SLCO1A2 | 0.0348 | 0.2098 | 2.5935 | 0.0019 | 0.0041 |
| ARVCF | 1.9547 | 4.5349 | 1.2141 | 0.0002 | 0.0005 |
| KRTCAP3 | 13.6027 | 27.7699 | 1.0296 | 0.0044 | 0.0085 |
| DOLPP1 | 6.2459 | 12.6364 | 1.0166 | 0.0000 | 0.0000 |
| KRT7 | 185.3456 | 554.7295 | 1.5816 | 0.0001 | 0.0003 |
| PGGHG | 7.1613 | 14.9634 | 1.0631 | 0.0008 | 0.0020 |
| ZNF66 | 0.5557 | 1.5220 | 1.4537 | 0.0164 | 0.0268 |
| COL7A1 | 4.3435 | 23.7313 | 2.4498 | 0.0000 | 0.0000 |
| ATP6V1FNB | 0.3707 | 0.9102 | 1.2959 | 0.0024 | 0.0050 |
| ST14 | 36.6951 | 78.5224 | 1.0975 | 0.0000 | 0.0001 |
| CADM3 | 5.8800 | 1.0951 | -2.4247 | 0.0000 | 0.0000 |
| ADAP1 | 1.2635 | 2.8271 | 1.1619 | 0.0021 | 0.0045 |
| SACS | 2.1991 | 1.0822 | -1.0230 | 0.0037 | 0.0074 |
| SMC4 | 2.8746 | 7.0354 | 1.2912 | 0.0000 | 0.0000 |
| MMP16 | 0.5076 | 0.2522 | -1.0088 | 0.0000 | 0.0001 |
| SHISAL1 | 8.3489 | 0.5268 | -3.9862 | 0.0000 | 0.0000 |
| PNKP | 6.3097 | 13.3597 | 1.0822 | 0.0000 | 0.0000 |
| MS4A15 | 0.0215 | 1.4159 | 6.0422 | 0.0007 | 0.0017 |
| SMUG1 | 3.6703 | 8.5763 | 1.2245 | 0.0000 | 0.0000 |
| ETV4 | 1.8238 | 11.4793 | 2.6540 | 0.0000 | 0.0000 |
| TP73 | 0.4265 | 1.6731 | 1.9719 | 0.0002 | 0.0007 |
| UHMK1 | 7.9266 | 16.8215 | 1.0855 | 0.0000 | 0.0000 |
| CREB5 | 1.3917 | 0.3870 | -1.8466 | 0.0000 | 0.0000 |
| CDC7 | 1.3699 | 4.5513 | 1.7322 | 0.0000 | 0.0000 |
| PLEKHH2 | 1.1045 | 0.5496 | -1.0069 | 0.0000 | 0.0001 |
| CHI3L1 | 7.7202 | 29.4251 | 1.9303 | 0.0022 | 0.0047 |
| CAV1 | 101.1735 | 43.5107 | -1.2174 | 0.0000 | 0.0001 |
| SPTBN2 | 3.1442 | 6.4363 | 1.0335 | 0.0002 | 0.0005 |
| ADCY4 | 1.8856 | 0.9398 | -1.0046 | 0.0000 | 0.0000 |
| BRIP1 | 0.3517 | 1.6757 | 2.2523 | 0.0000 | 0.0000 |
| NRP2 | 8.3073 | 3.1699 | -1.3900 | 0.0006 | 0.0014 |
| ZBED6CL | 2.0593 | 5.4487 | 1.4038 | 0.0002 | 0.0005 |
| STX2 | 5.0547 | 2.4305 | -1.0564 | 0.0002 | 0.0006 |
| DUS1L | 11.2504 | 27.6731 | 1.2985 | 0.0000 | 0.0000 |
| KLF4 | 59.1993 | 12.2355 | -2.2745 | 0.0000 | 0.0000 |
| DCUN1D3 | 6.4922 | 2.4205 | -1.4234 | 0.0000 | 0.0000 |
| RANBP1 | 9.9688 | 23.7105 | 1.2500 | 0.0000 | 0.0000 |
| HSPG2 | 36.3316 | 10.9181 | -1.7345 | 0.0103 | 0.0179 |
| RFTN2 | 0.9105 | 0.4396 | -1.0503 | 0.0000 | 0.0000 |
| TMEM147 | 32.5661 | 65.4509 | 1.0070 | 0.0000 | 0.0000 |
| PARVA | 20.3336 | 7.6312 | -1.4139 | 0.0000 | 0.0001 |
| FLVCR2 | 0.6198 | 1.5689 | 1.3398 | 0.0000 | 0.0000 |
| SLC7A9 | 0.0914 | 0.2451 | 1.4237 | 0.0190 | 0.0306 |
| PCSK4 | 0.3738 | 0.9369 | 1.3256 | 0.0010 | 0.0024 |
| C16orf45 | 13.2335 | 3.5220 | -1.9097 | 0.0000 | 0.0000 |
| FAM71E1 | 0.7917 | 1.8051 | 1.1890 | 0.0004 | 0.0010 |
| PPP1R14C | 1.7872 | 7.3379 | 2.0377 | 0.0000 | 0.0000 |
| HSPB3 | 1.4197 | 0.5136 | -1.4667 | 0.0002 | 0.0005 |
| CLIP4 | 6.5515 | 2.9403 | -1.1559 | 0.0000 | 0.0000 |
| TDO2 | 0.2211 | 1.2052 | 2.4467 | 0.0000 | 0.0000 |
| LMNB1 | 6.0267 | 21.1118 | 1.8086 | 0.0000 | 0.0000 |
| MSRB3 | 40.9359 | 4.4014 | -3.2173 | 0.0000 | 0.0000 |
| PRSS2 | 0.1603 | 32.9716 | 7.6840 | 0.0079 | 0.0142 |
| ASPSCR1 | 2.1473 | 4.8833 | 1.1853 | 0.0000 | 0.0000 |
| DMRTA2 | 0.0784 | 1.0685 | 3.7693 | 0.0000 | 0.0001 |
| COL14A1 | 30.0335 | 4.6672 | -2.6860 | 0.0000 | 0.0000 |
| IL6 | 48.1763 | 5.1994 | -3.2119 | 0.0000 | 0.0000 |
| RIPPLY3 | 0.6647 | 3.0654 | 2.2054 | 0.0000 | 0.0000 |
| NCAPH | 1.5307 | 7.7453 | 2.3391 | 0.0000 | 0.0000 |
| TMEM177 | 1.9069 | 4.0393 | 1.0829 | 0.0000 | 0.0000 |
| ANGPTL2 | 31.9755 | 13.0242 | -1.2958 | 0.0000 | 0.0001 |
| KHDC4 | 3.9942 | 10.2687 | 1.3623 | 0.0000 | 0.0000 |
| OTX1 | 0.1939 | 1.1397 | 2.5554 | 0.0000 | 0.0000 |
| CDC20 | 7.3976 | 43.7225 | 2.5632 | 0.0000 | 0.0000 |
| SH3BP2 | 2.2586 | 4.9361 | 1.1280 | 0.0000 | 0.0000 |
| ZNF91 | 1.4856 | 3.0063 | 1.0170 | 0.0283 | 0.0435 |
| SULT1C4 | 0.7684 | 0.3011 | -1.3516 | 0.0000 | 0.0000 |
| TERT | 0.0569 | 0.6295 | 3.4677 | 0.0000 | 0.0000 |
| RIBC2 | 0.5251 | 2.0732 | 1.9811 | 0.0000 | 0.0000 |
| SOX4 | 12.5263 | 43.1268 | 1.7836 | 0.0000 | 0.0000 |
| EN1 | 0.1755 | 1.1493 | 2.7116 | 0.0000 | 0.0001 |
| ADIPOQ | 1.3557 | 0.5744 | -1.2388 | 0.0000 | 0.0000 |
| VHL | 6.9205 | 14.4497 | 1.0621 | 0.0000 | 0.0000 |
| RGS1 | 24.7732 | 12.3512 | -1.0041 | 0.0000 | 0.0002 |
| MEOX2 | 2.2093 | 0.6233 | -1.8256 | 0.0000 | 0.0000 |
| SPDYA | 0.0971 | 0.2784 | 1.5192 | 0.0000 | 0.0000 |
| CNKSR3 | 1.5261 | 0.6078 | -1.3282 | 0.0000 | 0.0000 |
| PTGFR | 4.5520 | 0.4905 | -3.2141 | 0.0000 | 0.0000 |
| FAM24B | 0.5125 | 1.5596 | 1.6056 | 0.0000 | 0.0000 |
| CLSPN | 0.4496 | 1.9200 | 2.0944 | 0.0000 | 0.0000 |
| PHETA1 | 3.4138 | 7.0930 | 1.0550 | 0.0000 | 0.0000 |
| IRF4 | 1.5072 | 0.6514 | -1.2104 | 0.0016 | 0.0036 |
| CTGF | 424.0402 | 63.9133 | -2.7300 | 0.0000 | 0.0000 |
| PLAC1 | 0.4186 | 1.2515 | 1.5800 | 0.0192 | 0.0308 |
| UCP1 | 0.0176 | 0.2982 | 4.0849 | 0.0074 | 0.0134 |
| MTX1 | 3.0200 | 6.3024 | 1.0614 | 0.0000 | 0.0000 |
| TNNI3 | 0.0187 | 1.0347 | 5.7914 | 0.0000 | 0.0000 |
| LURAP1 | 1.3723 | 0.5339 | -1.3621 | 0.0000 | 0.0000 |
| PLA2G15 | 3.6044 | 8.5955 | 1.2538 | 0.0000 | 0.0000 |
| HDAC4 | 3.2887 | 0.9681 | -1.7644 | 0.0000 | 0.0000 |
| SIX1 | 0.5516 | 2.2571 | 2.0328 | 0.0000 | 0.0000 |
| CEP85L | 1.7851 | 0.6274 | -1.5084 | 0.0000 | 0.0000 |
| CCND2 | 18.6058 | 6.5882 | -1.4978 | 0.0000 | 0.0000 |
| TMEM119 | 17.3362 | 5.8088 | -1.5775 | 0.0000 | 0.0001 |
| KLHL42 | 9.9560 | 4.2697 | -1.2214 | 0.0238 | 0.0372 |
| LIX1L | 12.9297 | 6.2647 | -1.0454 | 0.0000 | 0.0000 |
| KCNG1 | 1.0852 | 8.8185 | 3.0225 | 0.0011 | 0.0026 |
| CREB3L4 | 3.6805 | 8.2415 | 1.1630 | 0.0000 | 0.0001 |
| UST | 7.4717 | 3.5006 | -1.0939 | 0.0000 | 0.0001 |
| MYEOV | 1.3908 | 8.2396 | 2.5666 | 0.0009 | 0.0021 |
| COA6 | 10.0568 | 21.6061 | 1.1033 | 0.0000 | 0.0000 |
| GOLGA8A | 2.7374 | 7.2940 | 1.4139 | 0.0005 | 0.0013 |
| ODC1 | 84.6765 | 31.9058 | -1.4081 | 0.0000 | 0.0001 |
| NCAM1 | 8.4621 | 0.8938 | -3.2429 | 0.0000 | 0.0000 |
| RUNDC3A | 0.1769 | 0.6816 | 1.9463 | 0.0137 | 0.0230 |
| PPP1R16B | 2.5495 | 1.1244 | -1.1811 | 0.0001 | 0.0004 |
| KREMEN2 | 0.2747 | 1.5189 | 2.4670 | 0.0000 | 0.0000 |
| CCDC167 | 14.8316 | 33.1146 | 1.1588 | 0.0000 | 0.0000 |
| CASP6 | 6.2565 | 14.2092 | 1.1834 | 0.0000 | 0.0000 |
| FOXD4 | 0.1521 | 0.3164 | 1.0571 | 0.0069 | 0.0127 |
| NGF | 2.1731 | 0.9890 | -1.1356 | 0.0000 | 0.0000 |
| NCAPD2 | 8.4120 | 21.3668 | 1.3448 | 0.0000 | 0.0000 |
| HIST1H2AH | 0.0182 | 1.5356 | 6.3951 | 0.0000 | 0.0000 |
| ARHGAP20 | 1.6390 | 0.2083 | -2.9763 | 0.0000 | 0.0000 |
| LEMD1 | 0.0362 | 0.5964 | 4.0415 | 0.0003 | 0.0009 |
| MAGEA1 | 0.3220 | 3.2190 | 3.3213 | 0.0053 | 0.0100 |
| HAVCR1 | 0.0166 | 3.0100 | 7.5002 | 0.0008 | 0.0019 |
| KIF24 | 0.3471 | 1.1979 | 1.7871 | 0.0000 | 0.0000 |
| PRR36 | 0.4653 | 3.5828 | 2.9450 | 0.0001 | 0.0002 |
| WFIKKN1 | 0.1028 | 0.2717 | 1.4020 | 0.0316 | 0.0478 |
| KIAA1841 | 0.9161 | 2.2694 | 1.3087 | 0.0000 | 0.0000 |
| GOLM1 | 12.2066 | 25.2110 | 1.0464 | 0.0001 | 0.0002 |
| BMPER | 0.5885 | 0.2683 | -1.1330 | 0.0000 | 0.0000 |
| CEP55 | 2.7530 | 11.4378 | 2.0547 | 0.0000 | 0.0000 |
| RHOB | 376.9711 | 99.4781 | -1.9220 | 0.0000 | 0.0000 |
| TNNT2 | 4.6585 | 0.7287 | -2.6765 | 0.0001 | 0.0004 |
| NUP210 | 3.5380 | 12.5686 | 1.8288 | 0.0000 | 0.0001 |
| ZNF124 | 0.6119 | 1.4170 | 1.2115 | 0.0001 | 0.0002 |
| RDM1 | 0.1754 | 1.4548 | 3.0522 | 0.0000 | 0.0000 |
| POLD1 | 3.3485 | 9.7851 | 1.5471 | 0.0000 | 0.0000 |
| C2CD4B | 6.8437 | 1.3494 | -2.3425 | 0.0000 | 0.0000 |
| PPP1R15A | 111.2462 | 47.5140 | -1.2273 | 0.0000 | 0.0000 |
| CORO1C | 36.9404 | 18.4301 | -1.0031 | 0.0006 | 0.0016 |
| 44259.0000 | 0.0075 | 0.4684 | 5.9603 | 0.0000 | 0.0000 |
| GEN1 | 0.6553 | 1.8886 | 1.5271 | 0.0000 | 0.0000 |
| SLC39A2 | 2.6503 | 6.6864 | 1.3351 | 0.0246 | 0.0384 |
| ANO7 | 0.1690 | 0.4586 | 1.4404 | 0.0000 | 0.0000 |
| DSC1 | 0.0792 | 0.3739 | 2.2384 | 0.0042 | 0.0081 |
| TEPSIN | 1.9469 | 4.4753 | 1.2008 | 0.0000 | 0.0000 |
| ASPM | 0.5735 | 3.2288 | 2.4932 | 0.0000 | 0.0000 |
| APOD | 51.2746 | 25.4396 | -1.0112 | 0.0000 | 0.0000 |
| RSRP1 | 3.7639 | 9.2675 | 1.3000 | 0.0000 | 0.0000 |
| TLCD1 | 6.0541 | 17.7800 | 1.5543 | 0.0000 | 0.0000 |
| ADRB2 | 2.8585 | 1.3633 | -1.0681 | 0.0000 | 0.0001 |
| ZIC2 | 0.3001 | 1.7803 | 2.5687 | 0.0001 | 0.0002 |
| PER3 | 5.1969 | 2.1440 | -1.2774 | 0.0000 | 0.0000 |
| DYNC1I1 | 6.3977 | 1.5778 | -2.0196 | 0.0030 | 0.0061 |
| LIMK1 | 4.5168 | 9.1375 | 1.0165 | 0.0000 | 0.0000 |
| KLRB1 | 5.1782 | 1.2965 | -1.9979 | 0.0010 | 0.0024 |
| DNAJB5 | 11.9928 | 2.0558 | -2.5444 | 0.0000 | 0.0000 |
| INPP5A | 17.6818 | 8.1377 | -1.1196 | 0.0000 | 0.0000 |
| FAM57B | 0.0144 | 0.2271 | 3.9821 | 0.0000 | 0.0001 |
| POLE2 | 0.7396 | 2.6516 | 1.8421 | 0.0000 | 0.0000 |
| GGN | 0.1504 | 0.3142 | 1.0626 | 0.0001 | 0.0003 |
| LYPD6B | 5.3445 | 10.8465 | 1.0211 | 0.0024 | 0.0050 |
| CITED4 | 9.5465 | 23.8647 | 1.3218 | 0.0020 | 0.0043 |
| DIRAS3 | 1.6683 | 0.6675 | -1.3215 | 0.0000 | 0.0000 |
| BDKRB1 | 5.0153 | 0.9777 | -2.3588 | 0.0000 | 0.0000 |
| MIS18A | 4.9402 | 13.5230 | 1.4528 | 0.0000 | 0.0000 |
| CLN6 | 6.5453 | 14.6742 | 1.1647 | 0.0000 | 0.0000 |
| PUS7 | 2.4839 | 5.5391 | 1.1570 | 0.0000 | 0.0000 |
| EXO5 | 1.5130 | 3.0545 | 1.0136 | 0.0000 | 0.0000 |
| ESRP1 | 15.9399 | 32.7323 | 1.0381 | 0.0001 | 0.0002 |
| PDIA3 | 72.0602 | 145.9551 | 1.0182 | 0.0000 | 0.0000 |
| COL27A1 | 1.1726 | 2.9529 | 1.3325 | 0.0024 | 0.0050 |
| FAM110A | 7.2051 | 17.8454 | 1.3085 | 0.0000 | 0.0000 |
| P2RY6 | 1.0725 | 2.9246 | 1.4473 | 0.0043 | 0.0083 |
| LYNX1 | 9.4984 | 4.0815 | -1.2186 | 0.0000 | 0.0000 |
| JDP2 | 8.5966 | 3.8244 | -1.1685 | 0.0000 | 0.0000 |
| SCUBE3 | 2.7209 | 0.8455 | -1.6862 | 0.0010 | 0.0024 |
| CLSTN3 | 5.7554 | 14.0865 | 1.2913 | 0.0000 | 0.0000 |
| CYP27A1 | 12.7580 | 5.7946 | -1.1386 | 0.0000 | 0.0000 |
| NXPH4 | 0.2958 | 4.9773 | 4.0726 | 0.0000 | 0.0000 |
| KANK2 | 55.3858 | 9.6790 | -2.5166 | 0.0000 | 0.0000 |
| DYSF | 8.8938 | 3.2608 | -1.4476 | 0.0028 | 0.0056 |
| UGT3A2 | 0.1467 | 0.4529 | 1.6265 | 0.0318 | 0.0482 |
| C4A | 2.1828 | 0.8612 | -1.3418 | 0.0002 | 0.0004 |
| PRKCZ | 1.6521 | 3.6478 | 1.1427 | 0.0000 | 0.0000 |
| APOBEC3B | 2.9555 | 11.3465 | 1.9408 | 0.0000 | 0.0001 |
| BAX | 18.0404 | 36.1608 | 1.0032 | 0.0000 | 0.0000 |
| DARS2 | 4.0843 | 10.5220 | 1.3653 | 0.0000 | 0.0000 |
| SHTN1 | 1.3221 | 2.7844 | 1.0745 | 0.0010 | 0.0024 |
| FABP6 | 3.0249 | 12.0602 | 1.9953 | 0.0012 | 0.0027 |
| ASTL | 2.3021 | 0.2802 | -3.0383 | 0.0172 | 0.0280 |
| RAI2 | 12.8151 | 2.2246 | -2.5262 | 0.0000 | 0.0000 |
| NUDT10 | 0.9284 | 0.3177 | -1.5473 | 0.0000 | 0.0000 |
| SNRPA1 | 6.0640 | 12.4286 | 1.0353 | 0.0000 | 0.0000 |
| ASIC3 | 0.2134 | 0.6000 | 1.4913 | 0.0052 | 0.0099 |
| AHNAK | 99.4444 | 41.9283 | -1.2460 | 0.0000 | 0.0000 |
| MRPL38 | 1.3786 | 2.7586 | 1.0008 | 0.0000 | 0.0000 |
| CSF3 | 35.1936 | 3.4281 | -3.3598 | 0.0000 | 0.0000 |
| C8orf44 | 0.8066 | 1.7554 | 1.1219 | 0.0000 | 0.0000 |
| KANK1 | 14.0295 | 5.1378 | -1.4492 | 0.0000 | 0.0000 |
| CSPG5 | 0.1386 | 0.4306 | 1.6351 | 0.0025 | 0.0051 |
| SIX4 | 0.5774 | 1.8946 | 1.7141 | 0.0000 | 0.0000 |
| TSPYL2 | 15.2949 | 5.7036 | -1.4231 | 0.0000 | 0.0000 |
| PPP1R1C | 0.1426 | 0.3965 | 1.4753 | 0.0026 | 0.0053 |
| KLHL35 | 0.1351 | 0.6378 | 2.2389 | 0.0000 | 0.0000 |
| ST6GALNAC3 | 0.6149 | 0.2599 | -1.2425 | 0.0000 | 0.0000 |
| FAM81A | 0.2866 | 0.5827 | 1.0236 | 0.0237 | 0.0371 |
| FXYD6 | 23.5517 | 2.7713 | -3.0872 | 0.0000 | 0.0000 |
| FAM155A | 0.6195 | 0.2109 | -1.5548 | 0.0002 | 0.0005 |
| SH3BGRL | 89.9767 | 37.8121 | -1.2507 | 0.0000 | 0.0000 |
| SGCG | 2.1925 | 0.1646 | -3.7354 | 0.0000 | 0.0000 |
| HAS1 | 7.4804 | 1.0839 | -2.7869 | 0.0000 | 0.0000 |
| DNAH17 | 0.1498 | 0.3732 | 1.3174 | 0.0000 | 0.0001 |
| SLC39A5 | 0.0347 | 0.8723 | 4.6509 | 0.0001 | 0.0002 |
| GPT2 | 3.5801 | 8.4266 | 1.2350 | 0.0000 | 0.0001 |
| PQLC2 | 3.6090 | 9.2917 | 1.3643 | 0.0000 | 0.0000 |
| RFC2 | 8.9883 | 21.0632 | 1.2286 | 0.0000 | 0.0000 |
| PRIM1 | 1.8560 | 4.9299 | 1.4094 | 0.0000 | 0.0000 |
| PALM3 | 2.6139 | 12.1088 | 2.2118 | 0.0027 | 0.0056 |
| ZNF772 | 0.6254 | 1.2648 | 1.0161 | 0.0001 | 0.0004 |
| PTCH1 | 2.1325 | 0.7531 | -1.5017 | 0.0000 | 0.0001 |
| SLIT2 | 4.2710 | 0.7326 | -2.5434 | 0.0000 | 0.0000 |
| KCNMA1 | 6.6627 | 0.5072 | -3.7155 | 0.0000 | 0.0000 |
| PRDM5 | 0.5941 | 0.2831 | -1.0695 | 0.0000 | 0.0000 |
| WFDC1 | 7.4097 | 1.4083 | -2.3954 | 0.0000 | 0.0000 |
| MMP9 | 6.2122 | 51.0776 | 3.0395 | 0.0031 | 0.0062 |
| LPCAT1 | 8.0778 | 23.2219 | 1.5234 | 0.0000 | 0.0000 |
| LDLR | 24.5650 | 9.6292 | -1.3511 | 0.0021 | 0.0044 |
| FBN2 | 0.3059 | 2.8532 | 3.2213 | 0.0006 | 0.0014 |
| GPR158 | 0.0655 | 0.4015 | 2.6167 | 0.0033 | 0.0066 |
| MCAM | 43.7821 | 13.6123 | -1.6854 | 0.0001 | 0.0002 |
| PLAU | 15.6299 | 74.6369 | 2.2556 | 0.0000 | 0.0000 |
| RASL11B | 1.4124 | 3.4122 | 1.2725 | 0.0222 | 0.0351 |
| XYLB | 0.7144 | 1.8213 | 1.3502 | 0.0000 | 0.0000 |
| BCL2L12 | 4.9453 | 13.8689 | 1.4877 | 0.0000 | 0.0000 |
| ZP1 | 0.0905 | 0.3813 | 2.0746 | 0.0033 | 0.0066 |
| KIF26B | 0.2469 | 1.7766 | 2.8470 | 0.0000 | 0.0000 |
| TLX3 | 0.1196 | 0.4960 | 2.0523 | 0.0152 | 0.0251 |
| NKX6-1 | 0.0556 | 0.3320 | 2.5772 | 0.0197 | 0.0316 |
| ZNF114 | 0.0806 | 1.4362 | 4.1546 | 0.0000 | 0.0000 |
| MASTL | 3.0756 | 6.7021 | 1.1237 | 0.0000 | 0.0000 |
| PDF | 0.9491 | 2.0691 | 1.1245 | 0.0000 | 0.0000 |
| SPHK1 | 3.0106 | 8.6235 | 1.5183 | 0.0167 | 0.0273 |
| CBX8 | 1.0299 | 3.3670 | 1.7089 | 0.0000 | 0.0000 |
| SLC16A4 | 2.9960 | 1.2393 | -1.2735 | 0.0000 | 0.0000 |
| HLF | 5.6872 | 0.2739 | -4.3761 | 0.0000 | 0.0000 |
| IGFL1 | 4.8976 | 82.6396 | 4.0767 | 0.0036 | 0.0071 |
| ADAMTS9 | 3.5346 | 1.1868 | -1.5745 | 0.0000 | 0.0001 |
| CIAO2A | 7.5972 | 15.2587 | 1.0061 | 0.0000 | 0.0000 |
| PDE5A | 7.6367 | 1.6613 | -2.2006 | 0.0000 | 0.0000 |
| FRAS1 | 0.3146 | 1.0543 | 1.7445 | 0.0097 | 0.0170 |
| HASPIN | 0.3812 | 1.5972 | 2.0669 | 0.0000 | 0.0000 |
| MYOM1 | 9.0309 | 0.4963 | -4.1856 | 0.0000 | 0.0000 |
| ZNF530 | 0.4931 | 1.1496 | 1.2211 | 0.0000 | 0.0000 |
| TFRC | 12.7036 | 32.3300 | 1.3476 | 0.0001 | 0.0003 |
| C1orf53 | 1.3983 | 4.4041 | 1.6552 | 0.0000 | 0.0000 |
| CST2 | 0.0239 | 1.8938 | 6.3087 | 0.0000 | 0.0000 |
| PLK1 | 2.2537 | 11.7791 | 2.3858 | 0.0000 | 0.0000 |
| MMP11 | 0.7980 | 36.2926 | 5.5072 | 0.0000 | 0.0000 |
| GALNT14 | 1.5769 | 5.8463 | 1.8905 | 0.0001 | 0.0004 |
| TARBP2 | 4.1748 | 9.6682 | 1.2115 | 0.0000 | 0.0000 |
| PGP | 5.0199 | 10.4098 | 1.0522 | 0.0000 | 0.0000 |
| KIFC1 | 3.2437 | 15.5663 | 2.2627 | 0.0000 | 0.0000 |
| APOL1 | 68.1457 | 153.7363 | 1.1738 | 0.0275 | 0.0424 |
| RGS17 | 0.2906 | 0.6116 | 1.0733 | 0.0223 | 0.0352 |
| RAC3 | 1.4856 | 10.8096 | 2.8632 | 0.0000 | 0.0000 |
| KIF2C | 2.4620 | 13.9113 | 2.4983 | 0.0000 | 0.0000 |
| PAQR6 | 1.0137 | 2.8900 | 1.5115 | 0.0003 | 0.0008 |
| TMSB15A | 0.7455 | 4.5654 | 2.6145 | 0.0008 | 0.0018 |
| MSH5 | 0.1584 | 0.5114 | 1.6910 | 0.0000 | 0.0000 |
| PCDHGB7 | 0.9761 | 0.3335 | -1.5492 | 0.0000 | 0.0000 |
| KLHL17 | 1.5259 | 4.2886 | 1.4909 | 0.0000 | 0.0000 |
| CCNB2 | 3.4143 | 16.0546 | 2.2333 | 0.0000 | 0.0000 |
| SNRPF | 11.3458 | 25.2518 | 1.1542 | 0.0000 | 0.0000 |
| IGFBP1 | 0.1298 | 0.3298 | 1.3453 | 0.0311 | 0.0472 |
| MZT1 | 6.2841 | 12.7462 | 1.0203 | 0.0000 | 0.0000 |
| TCEAL2 | 6.4352 | 0.9005 | -2.8372 | 0.0000 | 0.0000 |
| ATOH8 | 4.6684 | 2.3238 | -1.0064 | 0.0000 | 0.0000 |
| GNAZ | 4.1059 | 0.9553 | -2.1037 | 0.0000 | 0.0000 |
| ERRFI1 | 62.6652 | 30.5548 | -1.0363 | 0.0030 | 0.0060 |
| ITM2A | 24.0212 | 6.4503 | -1.8969 | 0.0000 | 0.0000 |
| RUNDC3B | 0.7450 | 0.2111 | -1.8196 | 0.0000 | 0.0000 |
| KRT83 | 0.0774 | 0.7412 | 3.2598 | 0.0271 | 0.0418 |
| C16orf89 | 13.4645 | 0.6925 | -4.2813 | 0.0000 | 0.0000 |
| MYRF | 0.3754 | 0.9703 | 1.3700 | 0.0023 | 0.0047 |
| SCN4B | 2.4954 | 0.5583 | -2.1602 | 0.0000 | 0.0000 |
| CDX1 | 0.0592 | 0.6609 | 3.4814 | 0.0019 | 0.0041 |
| C1QTNF2 | 2.2831 | 0.5859 | -1.9622 | 0.0000 | 0.0000 |
| EME1 | 0.2913 | 1.9957 | 2.7761 | 0.0000 | 0.0000 |
| ZEB2 | 3.3220 | 1.0306 | -1.6886 | 0.0000 | 0.0000 |
| KCNN2 | 1.0675 | 0.2771 | -1.9456 | 0.0004 | 0.0010 |
| CLIC4 | 109.6921 | 29.9993 | -1.8705 | 0.0007 | 0.0016 |
| SPTB | 0.0965 | 0.2208 | 1.1939 | 0.0294 | 0.0449 |
| TAS1R3 | 0.2372 | 0.9874 | 2.0572 | 0.0026 | 0.0053 |
| PRRG2 | 4.5824 | 9.8511 | 1.1042 | 0.0000 | 0.0001 |
| DUSP23 | 27.0895 | 57.7113 | 1.0911 | 0.0000 | 0.0000 |
| ITIH5 | 14.7794 | 1.4927 | -3.3076 | 0.0000 | 0.0000 |
| DQX1 | 1.1486 | 2.9049 | 1.3387 | 0.0008 | 0.0020 |
| KIF20A | 2.2715 | 10.7846 | 2.2473 | 0.0000 | 0.0000 |
| SLC39A7 | 39.5108 | 81.9434 | 1.0524 | 0.0000 | 0.0000 |
| CACNA1H | 21.2927 | 2.2190 | -3.2624 | 0.0000 | 0.0000 |
| RECK | 3.7696 | 1.0547 | -1.8376 | 0.0000 | 0.0000 |
| DGKG | 1.3490 | 0.3059 | -2.1408 | 0.0000 | 0.0000 |
| IGFL3 | 0.0537 | 0.6188 | 3.5265 | 0.0006 | 0.0014 |
| C2orf68 | 4.9941 | 10.3693 | 1.0540 | 0.0000 | 0.0000 |
| RUSC2 | 11.5009 | 3.9758 | -1.5324 | 0.0000 | 0.0000 |
| TEN1-CDK3 | 0.2820 | 0.6560 | 1.2183 | 0.0001 | 0.0002 |
| SMTNL2 | 2.5703 | 0.9768 | -1.3959 | 0.0001 | 0.0002 |
| SLC22A1 | 0.1113 | 0.4298 | 1.9485 | 0.0054 | 0.0102 |
| TCIM | 180.7090 | 49.0724 | -1.8807 | 0.0000 | 0.0000 |
| SERTAD1 | 34.9981 | 14.7754 | -1.2441 | 0.0002 | 0.0005 |
| IL17B | 0.6657 | 0.2294 | -1.5371 | 0.0002 | 0.0007 |
| BBC3 | 2.5007 | 5.8318 | 1.2216 | 0.0000 | 0.0000 |
| TSC22D1 | 58.3317 | 21.2771 | -1.4550 | 0.0000 | 0.0000 |
| IL33 | 15.5067 | 6.1565 | -1.3327 | 0.0000 | 0.0000 |
| TCEAL7 | 6.8890 | 1.4009 | -2.2979 | 0.0000 | 0.0000 |
| NUP155 | 2.9608 | 6.2742 | 1.0834 | 0.0000 | 0.0000 |
| SYT12 | 0.3115 | 0.9652 | 1.6315 | 0.0159 | 0.0261 |
| MX1 | 8.5293 | 20.4874 | 1.2642 | 0.0322 | 0.0486 |
| CLCA4 | 68.7061 | 15.1314 | -2.1829 | 0.0159 | 0.0261 |
| CHTF18 | 1.7040 | 6.1852 | 1.8599 | 0.0000 | 0.0000 |
| AMOTL1 | 17.4275 | 5.6438 | -1.6266 | 0.0001 | 0.0004 |
| FXYD1 | 1.9309 | 0.2539 | -2.9273 | 0.0000 | 0.0000 |
| DHCR7 | 6.6451 | 20.6557 | 1.6362 | 0.0000 | 0.0000 |
| FBXL6 | 3.2467 | 11.3758 | 1.8089 | 0.0000 | 0.0000 |
| ITPRIP | 13.6094 | 6.5385 | -1.0576 | 0.0000 | 0.0001 |
| CXCL11 | 1.3557 | 13.6876 | 3.3357 | 0.0004 | 0.0010 |
| LRRC43 | 0.0971 | 0.3354 | 1.7883 | 0.0000 | 0.0001 |
| FASN | 15.8355 | 49.7691 | 1.6521 | 0.0000 | 0.0000 |
| TM4SF19 | 0.0530 | 0.9690 | 4.1931 | 0.0000 | 0.0000 |
| C11orf45 | 0.4198 | 0.8936 | 1.0898 | 0.0006 | 0.0015 |
| CCDC78 | 0.2629 | 0.6492 | 1.3043 | 0.0000 | 0.0000 |
| SYNE1 | 2.7161 | 0.5920 | -2.1980 | 0.0000 | 0.0000 |
| ZNF706 | 7.4409 | 14.9991 | 1.0113 | 0.0000 | 0.0000 |
| ASCL5 | 0.0383 | 0.3267 | 3.0926 | 0.0000 | 0.0001 |
| RPS6KA2 | 9.2674 | 3.2599 | -1.5073 | 0.0000 | 0.0000 |
| RTKN2 | 0.4482 | 1.5161 | 1.7582 | 0.0000 | 0.0000 |
| FBLN5 | 20.9898 | 4.4483 | -2.2384 | 0.0000 | 0.0000 |
| EPCAM | 19.0271 | 63.5209 | 1.7392 | 0.0000 | 0.0000 |
| AQP9 | 0.5836 | 1.5908 | 1.4465 | 0.0192 | 0.0308 |
| EBF3 | 1.3253 | 0.4675 | -1.5032 | 0.0000 | 0.0000 |
| SLFN13 | 0.7769 | 2.7644 | 1.8311 | 0.0070 | 0.0128 |
| LY9 | 0.4572 | 0.2278 | -1.0054 | 0.0149 | 0.0247 |
| SPINT1 | 46.3560 | 110.7595 | 1.2566 | 0.0000 | 0.0000 |
| SCG5 | 0.7979 | 2.1923 | 1.4581 | 0.0007 | 0.0017 |
| MTHFD2 | 6.7906 | 14.8738 | 1.1312 | 0.0000 | 0.0001 |
| RNF112 | 1.5196 | 0.3179 | -2.2570 | 0.0000 | 0.0000 |
| PRKAR2B | 8.3802 | 2.6336 | -1.6699 | 0.0000 | 0.0000 |
| AC007906.2 | 9.1802 | 3.9547 | -1.2150 | 0.0159 | 0.0261 |
| GREM2 | 3.1735 | 0.4121 | -2.9451 | 0.0000 | 0.0000 |
| DLX4 | 0.1106 | 0.7655 | 2.7904 | 0.0000 | 0.0002 |
| CLEC18B | 0.0850 | 0.2266 | 1.4152 | 0.0037 | 0.0074 |
| ENGASE | 5.1678 | 10.7751 | 1.0601 | 0.0002 | 0.0007 |
| CDSN | 0.0074 | 0.2176 | 4.8760 | 0.0038 | 0.0074 |
| FAM102B | 7.0632 | 2.9867 | -1.2418 | 0.0000 | 0.0000 |
| LYVE1 | 10.3098 | 1.9692 | -2.3883 | 0.0000 | 0.0000 |
| FAM107A | 14.3509 | 0.9874 | -3.8614 | 0.0000 | 0.0000 |
| RND1 | 5.3873 | 1.4664 | -1.8773 | 0.0002 | 0.0005 |
| CSGALNACT1 | 6.6868 | 1.7925 | -1.8993 | 0.0000 | 0.0000 |
| PTGS2 | 62.5859 | 14.6684 | -2.0931 | 0.0000 | 0.0000 |
| SMIM22 | 13.1765 | 32.0965 | 1.2844 | 0.0019 | 0.0042 |
| LRRC26 | 0.0559 | 0.2084 | 1.8987 | 0.0049 | 0.0094 |
| CYB561 | 9.9078 | 25.1816 | 1.3457 | 0.0000 | 0.0000 |
| TENT5A | 11.6553 | 4.6083 | -1.3387 | 0.0000 | 0.0000 |
| PRUNE2 | 15.8057 | 0.9962 | -3.9878 | 0.0000 | 0.0000 |
| SLC35A2 | 7.8391 | 19.3207 | 1.3014 | 0.0000 | 0.0000 |
| C2orf88 | 1.4297 | 0.5859 | -1.2869 | 0.0000 | 0.0000 |
| A2M | 321.6205 | 74.2804 | -2.1143 | 0.0000 | 0.0000 |
| DDX39A | 12.0240 | 27.8577 | 1.2122 | 0.0000 | 0.0000 |
| FNDC10 | 3.2642 | 10.3264 | 1.6615 | 0.0000 | 0.0000 |
| HAAO | 7.8920 | 1.7930 | -2.1380 | 0.0000 | 0.0000 |
| CABP1 | 0.6364 | 0.2445 | -1.3803 | 0.0000 | 0.0000 |
| HCAR1 | 1.1656 | 4.4021 | 1.9170 | 0.0002 | 0.0005 |
| ENDOU | 3.1882 | 0.3305 | -3.2702 | 0.0000 | 0.0000 |
| MCM4 | 7.8200 | 24.5604 | 1.6511 | 0.0000 | 0.0000 |
| TFAP2A | 2.2648 | 6.1705 | 1.4460 | 0.0002 | 0.0007 |
| GPRIN2 | 0.8814 | 2.3780 | 1.4318 | 0.0144 | 0.0240 |
| CD164L2 | 0.2320 | 0.6358 | 1.4543 | 0.0001 | 0.0002 |
| HAP1 | 0.1208 | 0.7534 | 2.6404 | 0.0008 | 0.0019 |
| TSC22D3 | 51.0951 | 21.3582 | -1.2584 | 0.0000 | 0.0000 |
| TSSK6 | 0.5042 | 1.1141 | 1.1438 | 0.0000 | 0.0000 |
| SERPINA6 | 0.0069 | 0.4540 | 6.0485 | 0.0138 | 0.0232 |
| NDNF | 4.4007 | 0.5756 | -2.9347 | 0.0000 | 0.0000 |
| PRDM6 | 4.0127 | 0.5048 | -2.9907 | 0.0000 | 0.0000 |
| ZFP36 | 881.8590 | 137.1978 | -2.6843 | 0.0000 | 0.0000 |
| TBX1 | 1.3859 | 8.6971 | 2.6497 | 0.0048 | 0.0091 |
| SHOX2 | 0.1144 | 0.6381 | 2.4794 | 0.0070 | 0.0127 |
| DMBX1 | 0.2710 | 2.0777 | 2.9387 | 0.0001 | 0.0002 |
| DTNA | 6.8806 | 0.5750 | -3.5808 | 0.0000 | 0.0000 |
| BHLHA15 | 0.2173 | 0.4421 | 1.0246 | 0.0153 | 0.0252 |
| PJA2 | 37.6697 | 12.7509 | -1.5628 | 0.0000 | 0.0000 |
| LOXL2 | 4.4869 | 9.3215 | 1.0549 | 0.0112 | 0.0193 |
| CCDC18 | 0.5750 | 1.4949 | 1.3784 | 0.0000 | 0.0000 |
| POP7 | 18.0507 | 40.4375 | 1.1636 | 0.0000 | 0.0000 |
| SLC25A35 | 1.3633 | 2.7973 | 1.0370 | 0.0057 | 0.0107 |
| KCND3 | 4.6591 | 0.7662 | -2.6043 | 0.0000 | 0.0000 |
| OLFM2 | 1.6496 | 5.5741 | 1.7566 | 0.0051 | 0.0097 |
| RIPK2 | 5.5292 | 11.4969 | 1.0561 | 0.0000 | 0.0000 |
| RFC4 | 3.5976 | 11.0515 | 1.6192 | 0.0000 | 0.0000 |
| LAMP3 | 3.1066 | 6.3454 | 1.0304 | 0.0194 | 0.0311 |
| FGF7 | 7.9917 | 1.6394 | -2.2854 | 0.0000 | 0.0000 |
| ABCA9 | 1.6180 | 0.4894 | -1.7252 | 0.0000 | 0.0000 |
| FBLN2 | 59.4174 | 13.6262 | -2.1245 | 0.0000 | 0.0000 |
| TCF4 | 3.3797 | 1.6738 | -1.0138 | 0.0000 | 0.0000 |
| SVEP1 | 5.2590 | 0.7044 | -2.9004 | 0.0000 | 0.0000 |
| C6orf223 | 0.0568 | 0.9043 | 3.9927 | 0.0000 | 0.0000 |
| ZBED9 | 0.1920 | 0.6213 | 1.6943 | 0.0000 | 0.0002 |
| MYCT1 | 4.6248 | 1.6971 | -1.4463 | 0.0000 | 0.0000 |
| SPATA17 | 0.1247 | 0.5502 | 2.1412 | 0.0000 | 0.0000 |
| C1QTNF6 | 1.4492 | 8.8204 | 2.6056 | 0.0000 | 0.0000 |
| ERCC6L | 0.4257 | 1.9220 | 2.1747 | 0.0000 | 0.0000 |
| EVC | 3.9401 | 1.7006 | -1.2122 | 0.0001 | 0.0003 |
| ENTPD1 | 6.9985 | 3.2730 | -1.0964 | 0.0001 | 0.0004 |
| CALML5 | 21.8687 | 81.1498 | 1.8917 | 0.0017 | 0.0038 |
| KLHL5 | 10.3666 | 4.5879 | -1.1760 | 0.0113 | 0.0194 |
| LIF | 16.1223 | 5.7131 | -1.4967 | 0.0015 | 0.0033 |
| RMI1 | 1.8606 | 4.5694 | 1.2963 | 0.0000 | 0.0000 |
| CACNB4 | 0.8812 | 0.1707 | -2.3680 | 0.0000 | 0.0000 |
| SLC50A1 | 14.4267 | 37.0972 | 1.3626 | 0.0000 | 0.0000 |
| CHCHD1 | 14.1609 | 28.3934 | 1.0036 | 0.0000 | 0.0000 |
| TBX19 | 0.5113 | 1.1081 | 1.1159 | 0.0000 | 0.0000 |
| MRAS | 7.1115 | 2.9868 | -1.2515 | 0.0035 | 0.0069 |
| TTK | 0.8738 | 4.8862 | 2.4834 | 0.0000 | 0.0000 |
| CCL19 | 44.3653 | 13.4599 | -1.7208 | 0.0014 | 0.0031 |
| GLA | 8.4472 | 17.0879 | 1.0164 | 0.0000 | 0.0000 |
| CPEB2 | 5.4408 | 1.9141 | -1.5072 | 0.0000 | 0.0000 |
| OSBPL10 | 6.6842 | 2.0582 | -1.6994 | 0.0000 | 0.0000 |
| LRCH2 | 3.0456 | 0.6393 | -2.2522 | 0.0000 | 0.0000 |
| MTMR4 | 3.8641 | 8.2989 | 1.1028 | 0.0000 | 0.0000 |
| CREM | 8.8763 | 3.6949 | -1.2644 | 0.0000 | 0.0000 |
| LSR | 49.9109 | 111.3739 | 1.1580 | 0.0000 | 0.0000 |
| TMEM184A | 4.9428 | 10.1902 | 1.0438 | 0.0024 | 0.0050 |
| CXCR2 | 3.5166 | 0.7448 | -2.2392 | 0.0005 | 0.0013 |
| MAP3K9 | 0.6037 | 1.2118 | 1.0053 | 0.0003 | 0.0008 |
| RASL12 | 22.8709 | 3.3649 | -2.7649 | 0.0000 | 0.0000 |
| CRTAP | 45.0074 | 22.0477 | -1.0295 | 0.0000 | 0.0000 |
| GALR2 | 0.1732 | 0.7917 | 2.1924 | 0.0109 | 0.0187 |
| BDNF | 0.8560 | 0.2484 | -1.7848 | 0.0000 | 0.0000 |
| PSMC3IP | 1.1775 | 2.7151 | 1.2053 | 0.0000 | 0.0000 |
| KIF11 | 2.4796 | 9.2166 | 1.8941 | 0.0000 | 0.0000 |
| GULP1 | 1.7007 | 0.5861 | -1.5370 | 0.0000 | 0.0000 |
| TEFM | 1.2654 | 2.5619 | 1.0177 | 0.0000 | 0.0000 |
| FLVCR1 | 1.2358 | 3.3656 | 1.4455 | 0.0000 | 0.0000 |
| DNAJC18 | 2.2314 | 0.9771 | -1.1913 | 0.0000 | 0.0000 |
| PLA2G2A | 124.4721 | 59.7398 | -1.0591 | 0.0000 | 0.0001 |
| METTL7B | 0.6186 | 3.6855 | 2.5747 | 0.0001 | 0.0002 |
| TUB | 2.7990 | 0.7895 | -1.8259 | 0.0000 | 0.0000 |
| DMPK | 31.1376 | 9.0286 | -1.7861 | 0.0001 | 0.0002 |
| ERO1A | 10.1625 | 21.7381 | 1.0970 | 0.0000 | 0.0000 |
| DPYSL3 | 72.0979 | 17.7174 | -2.0248 | 0.0000 | 0.0001 |
| OAS3 | 6.5833 | 18.8650 | 1.5188 | 0.0000 | 0.0001 |
| SPAG5 | 2.1245 | 10.3706 | 2.2873 | 0.0000 | 0.0000 |
| PGM5 | 59.1299 | 2.6743 | -4.4666 | 0.0000 | 0.0000 |
| FOXM1 | 2.6602 | 13.4652 | 2.3396 | 0.0000 | 0.0000 |
| ADRB3 | 1.9244 | 0.1737 | -3.4695 | 0.0000 | 0.0000 |
| PPFIA3 | 1.5722 | 3.4505 | 1.1341 | 0.0001 | 0.0004 |
| PTGDS | 77.6312 | 16.4323 | -2.2401 | 0.0000 | 0.0000 |
| SRMS | 1.6220 | 3.4035 | 1.0692 | 0.0062 | 0.0116 |
| CHAC1 | 0.6052 | 3.1132 | 2.3630 | 0.0000 | 0.0000 |
| MYCL | 13.6269 | 28.9523 | 1.0872 | 0.0088 | 0.0155 |
| CHRNB1 | 1.0872 | 3.1433 | 1.5316 | 0.0000 | 0.0000 |
| HMGB3 | 6.4057 | 27.4187 | 2.0977 | 0.0000 | 0.0000 |
| ANKEF1 | 0.9966 | 2.5846 | 1.3748 | 0.0000 | 0.0000 |
| OLR1 | 1.2903 | 9.2288 | 2.8385 | 0.0051 | 0.0097 |
| COL10A1 | 0.0536 | 6.9252 | 7.0147 | 0.0000 | 0.0000 |
| ASPA | 2.1737 | 0.2457 | -3.1449 | 0.0000 | 0.0000 |
| SGCA | 15.0057 | 1.4729 | -3.3488 | 0.0000 | 0.0000 |
| ADAMTS4 | 19.0108 | 3.1275 | -2.6037 | 0.0002 | 0.0006 |
| PFKFB4 | 0.6591 | 3.7775 | 2.5190 | 0.0000 | 0.0000 |
| PPP1R14B | 30.9419 | 85.4755 | 1.4659 | 0.0000 | 0.0000 |
| ZHX1-C8orf76 | 0.3688 | 1.0100 | 1.4534 | 0.0000 | 0.0000 |
| TMEM234 | 2.1396 | 4.3639 | 1.0283 | 0.0000 | 0.0000 |
| TCEAL5 | 1.0764 | 0.4092 | -1.3954 | 0.0000 | 0.0000 |
| TMC5 | 0.8287 | 1.8916 | 1.1906 | 0.0278 | 0.0427 |
| STK40 | 18.7246 | 8.8455 | -1.0819 | 0.0000 | 0.0000 |
| QRFP | 0.9102 | 0.3504 | -1.3771 | 0.0000 | 0.0000 |
| IL11RA | 4.2558 | 2.0651 | -1.0432 | 0.0000 | 0.0000 |
| HTR1B | 0.9872 | 0.1994 | -2.3078 | 0.0000 | 0.0000 |
| CAND2 | 2.0454 | 0.9697 | -1.0767 | 0.0001 | 0.0004 |
| HIST1H2BC | 0.7421 | 6.4258 | 3.1141 | 0.0000 | 0.0000 |
| KLK6 | 6.4971 | 13.9044 | 1.0977 | 0.0074 | 0.0134 |
| SQLE | 6.8873 | 21.4577 | 1.6395 | 0.0000 | 0.0000 |
| KCNH2 | 6.1205 | 1.2834 | -2.2537 | 0.0000 | 0.0000 |
| EPHA3 | 5.4305 | 1.0266 | -2.4032 | 0.0000 | 0.0000 |
| ACTN2 | 3.1360 | 0.1915 | -4.0332 | 0.0000 | 0.0001 |
| PARM1 | 22.7685 | 8.0902 | -1.4928 | 0.0000 | 0.0000 |
| RAPSN | 0.0943 | 0.5963 | 2.6614 | 0.0001 | 0.0004 |
| ONECUT2 | 0.1090 | 0.6286 | 2.5276 | 0.0019 | 0.0041 |
| FLRT2 | 0.5969 | 0.2725 | -1.1310 | 0.0000 | 0.0000 |
| ATAD5 | 0.4544 | 1.6815 | 1.8876 | 0.0000 | 0.0000 |
| DNAH14 | 0.1279 | 0.3933 | 1.6205 | 0.0000 | 0.0000 |
| POLR3G | 0.4762 | 1.0673 | 1.1643 | 0.0102 | 0.0177 |
| OLFML3 | 36.4901 | 11.6032 | -1.6530 | 0.0000 | 0.0000 |
| TPBGL | 0.1904 | 0.6875 | 1.8526 | 0.0151 | 0.0250 |
| ADCK5 | 3.3697 | 7.7565 | 1.2028 | 0.0000 | 0.0000 |
| CLCN2 | 0.9323 | 2.8245 | 1.5992 | 0.0000 | 0.0000 |
| ZNF707 | 1.3078 | 2.7665 | 1.0809 | 0.0000 | 0.0000 |
| HIST1H3E | 0.5545 | 1.9548 | 1.8179 | 0.0001 | 0.0004 |
| STK32B | 0.9484 | 0.3194 | -1.5702 | 0.0000 | 0.0000 |
| MFAP4 | 236.6482 | 29.5257 | -3.0027 | 0.0000 | 0.0000 |
| DCBLD1 | 1.4603 | 3.8352 | 1.3931 | 0.0000 | 0.0000 |
| BCL2L2 | 23.9360 | 10.6762 | -1.1648 | 0.0000 | 0.0000 |
| LHX2 | 0.0587 | 0.3887 | 2.7264 | 0.0001 | 0.0002 |
| HIST1H2BN | 0.0952 | 0.4345 | 2.1898 | 0.0000 | 0.0000 |
| FUOM | 4.1031 | 9.7981 | 1.2558 | 0.0001 | 0.0003 |
| NCR3 | 1.3361 | 0.3873 | -1.7864 | 0.0207 | 0.0331 |
| NAP1L2 | 2.9668 | 0.8416 | -1.8177 | 0.0000 | 0.0000 |
| CYBRD1 | 41.1373 | 10.0065 | -2.0395 | 0.0000 | 0.0000 |
| MICU3 | 1.7784 | 0.2331 | -2.9313 | 0.0000 | 0.0000 |
| PLSCR4 | 6.6823 | 2.0535 | -1.7023 | 0.0000 | 0.0000 |
| SH3BGR | 14.6159 | 2.4585 | -2.5717 | 0.0000 | 0.0000 |
| ORC6 | 0.8662 | 4.0007 | 2.2074 | 0.0000 | 0.0000 |
| ZNF695 | 0.0896 | 0.5763 | 2.6857 | 0.0000 | 0.0000 |
| REV3L | 3.6140 | 1.5777 | -1.1958 | 0.0000 | 0.0000 |
| ADCY9 | 4.1589 | 1.7957 | -1.2117 | 0.0000 | 0.0000 |
| SLC37A4 | 2.1808 | 5.7623 | 1.4018 | 0.0000 | 0.0000 |
| ICAM5 | 0.1463 | 0.5880 | 2.0068 | 0.0033 | 0.0065 |
| DNASE1L2 | 0.1704 | 0.7918 | 2.2163 | 0.0000 | 0.0000 |
| RLN1 | 0.0663 | 0.3114 | 2.2319 | 0.0016 | 0.0035 |
| ITGB4 | 43.8602 | 91.6613 | 1.0634 | 0.0008 | 0.0019 |
| CKLF | 4.5894 | 10.4205 | 1.1830 | 0.0000 | 0.0000 |
| TMEM52B | 0.0725 | 1.3881 | 4.2590 | 0.0000 | 0.0000 |
| NPIPB3 | 0.1246 | 0.2731 | 1.1315 | 0.0015 | 0.0034 |
| STARD13 | 5.0764 | 1.5371 | -1.7236 | 0.0000 | 0.0000 |
| SPC25 | 1.2958 | 5.1748 | 1.9977 | 0.0000 | 0.0000 |
| TIPARP | 31.2489 | 8.7226 | -1.8410 | 0.0000 | 0.0000 |
| HAPLN1 | 0.1408 | 0.6386 | 2.1817 | 0.0055 | 0.0103 |
| ITPKA | 0.3083 | 1.2260 | 1.9917 | 0.0194 | 0.0311 |
| CCDC7 | 0.1447 | 0.2967 | 1.0355 | 0.0000 | 0.0000 |
| CATSPER1 | 0.1161 | 1.3903 | 3.5821 | 0.0000 | 0.0000 |
| HAND2 | 7.8585 | 0.9219 | -3.0916 | 0.0000 | 0.0000 |
| BAG3 | 66.3299 | 30.5549 | -1.1183 | 0.0001 | 0.0003 |
| PSD | 19.2065 | 1.6731 | -3.5210 | 0.0000 | 0.0000 |
| RND3 | 54.5327 | 21.7109 | -1.3287 | 0.0000 | 0.0001 |
| PAFAH1B3 | 16.4295 | 53.5983 | 1.7059 | 0.0000 | 0.0000 |
| MCM7 | 20.4827 | 46.6211 | 1.1866 | 0.0000 | 0.0000 |
| DNAAF3 | 0.0800 | 0.3821 | 2.2555 | 0.0001 | 0.0004 |
| PRKN | 1.5139 | 0.2793 | -2.4382 | 0.0000 | 0.0000 |
| ZNF25 | 4.2746 | 1.9974 | -1.0977 | 0.0000 | 0.0001 |
| ADAMTS5 | 1.9498 | 0.6285 | -1.6333 | 0.0000 | 0.0000 |
| CLPSL1 | 0.0261 | 0.3797 | 3.8601 | 0.0000 | 0.0001 |
| TLX2 | 0.0272 | 0.2407 | 3.1459 | 0.0000 | 0.0000 |
| MUC1 | 20.6476 | 62.6368 | 1.6010 | 0.0286 | 0.0439 |
| PAX8 | 0.8639 | 1.7495 | 1.0180 | 0.0035 | 0.0070 |
| HDAC10 | 1.4971 | 3.0560 | 1.0295 | 0.0000 | 0.0001 |
| DLC1 | 5.4373 | 2.0552 | -1.4036 | 0.0000 | 0.0000 |
| ARTN | 0.8462 | 2.3809 | 1.4924 | 0.0010 | 0.0023 |
| CGB3 | 0.0050 | 0.7206 | 7.1670 | 0.0000 | 0.0001 |
| TMEM238L | 14.1666 | 4.5911 | -1.6256 | 0.0083 | 0.0148 |
| FOSB | 289.0955 | 22.5471 | -3.6805 | 0.0000 | 0.0000 |
| AC138811.2 | 0.1117 | 0.2785 | 1.3178 | 0.0006 | 0.0014 |
| HDGF | 59.3717 | 118.9261 | 1.0022 | 0.0000 | 0.0000 |
| WLS | 36.2799 | 16.8478 | -1.1066 | 0.0000 | 0.0000 |
| ZNF835 | 0.5650 | 0.2177 | -1.3760 | 0.0001 | 0.0003 |
| CHRDL1 | 12.1724 | 1.5907 | -2.9359 | 0.0000 | 0.0000 |
| AJUBA | 2.9281 | 6.8880 | 1.2341 | 0.0006 | 0.0014 |
| GALNT17 | 5.4716 | 0.5992 | -3.1909 | 0.0000 | 0.0000 |
| VGLL1 | 13.2884 | 46.9325 | 1.8204 | 0.0003 | 0.0008 |
| HOXB6 | 1.7977 | 6.0275 | 1.7454 | 0.0000 | 0.0000 |
| ITGB6 | 11.8148 | 25.5941 | 1.1152 | 0.0023 | 0.0048 |
| SLC16A7 | 1.7785 | 0.8729 | -1.0268 | 0.0000 | 0.0000 |
| DSCC1 | 1.3420 | 4.3175 | 1.6859 | 0.0000 | 0.0000 |
| RRM2 | 5.4846 | 20.2328 | 1.8833 | 0.0000 | 0.0000 |
| MMRN1 | 3.7373 | 0.5310 | -2.8151 | 0.0000 | 0.0000 |
| KSR2 | 0.0859 | 0.3113 | 1.8574 | 0.0151 | 0.0250 |
| FGF11 | 0.0649 | 0.3050 | 2.2317 | 0.0000 | 0.0001 |
| PDCD5 | 16.2960 | 33.8690 | 1.0554 | 0.0000 | 0.0000 |
| ZNF521 | 2.1093 | 0.9252 | -1.1889 | 0.0000 | 0.0000 |
| ULBP3 | 0.6052 | 1.3014 | 1.1046 | 0.0016 | 0.0035 |
| HIST1H4K | 0.1488 | 0.6517 | 2.1311 | 0.0003 | 0.0008 |
| ADAM33 | 7.9763 | 1.0312 | -2.9513 | 0.0000 | 0.0000 |
| STRIP2 | 0.2025 | 1.1580 | 2.5159 | 0.0000 | 0.0000 |
| PLOD1 | 17.5966 | 38.2379 | 1.1197 | 0.0000 | 0.0000 |
| GNG11 | 18.9728 | 6.2134 | -1.6105 | 0.0000 | 0.0000 |
| KIF18A | 0.4353 | 2.1193 | 2.2835 | 0.0000 | 0.0000 |
| ARHGEF39 | 0.4864 | 1.8195 | 1.9032 | 0.0000 | 0.0000 |
| TINCR | 1.9831 | 7.0111 | 1.8219 | 0.0004 | 0.0010 |
| DDX55 | 2.3653 | 4.8575 | 1.0382 | 0.0000 | 0.0000 |
| AKR1E2 | 0.7937 | 1.8455 | 1.2174 | 0.0187 | 0.0302 |
| PMP22 | 63.3313 | 20.1608 | -1.6514 | 0.0000 | 0.0000 |
| MPPED2 | 1.9627 | 0.7350 | -1.4171 | 0.0000 | 0.0001 |
| MMP14 | 58.4714 | 132.2110 | 1.1770 | 0.0000 | 0.0001 |
| HIST1H4C | 0.0756 | 2.8756 | 5.2495 | 0.0000 | 0.0000 |
| TUBA3D | 0.0926 | 0.2131 | 1.2032 | 0.0211 | 0.0336 |
| HEPHL1 | 0.0980 | 1.3974 | 3.8336 | 0.0280 | 0.0430 |
| GJC2 | 2.3526 | 0.7145 | -1.7193 | 0.0000 | 0.0000 |
| LRRC39 | 0.2623 | 0.5991 | 1.1913 | 0.0001 | 0.0003 |
| CSDC2 | 4.4825 | 1.3000 | -1.7857 | 0.0000 | 0.0000 |
| TMEM223 | 4.8328 | 10.7968 | 1.1597 | 0.0000 | 0.0000 |
| SEC61G | 20.4386 | 51.2626 | 1.3266 | 0.0000 | 0.0000 |
| ERBB2 | 23.0180 | 63.2752 | 1.4589 | 0.0040 | 0.0079 |
| CYYR1 | 6.7032 | 2.6901 | -1.3172 | 0.0000 | 0.0000 |
| CENPF | 2.0538 | 8.0212 | 1.9655 | 0.0000 | 0.0000 |
| LILRA4 | 0.5041 | 0.2435 | -1.0494 | 0.0167 | 0.0273 |
| ECM1 | 32.4574 | 13.4091 | -1.2753 | 0.0090 | 0.0160 |
| CAD | 4.6884 | 9.8585 | 1.0723 | 0.0000 | 0.0000 |
| GPR183 | 14.8467 | 5.5360 | -1.4232 | 0.0000 | 0.0000 |
| SLC9A5 | 0.1652 | 0.3838 | 1.2166 | 0.0001 | 0.0003 |
| PMEL | 0.5446 | 1.4773 | 1.4399 | 0.0003 | 0.0009 |
| TES | 48.9990 | 23.7909 | -1.0423 | 0.0001 | 0.0003 |
| GLOD5 | 0.1079 | 0.2588 | 1.2619 | 0.0030 | 0.0061 |
| DACH1 | 1.3193 | 0.4687 | -1.4930 | 0.0000 | 0.0000 |
| IL17RD | 2.3274 | 1.0205 | -1.1894 | 0.0038 | 0.0074 |
| PCGF1 | 3.3825 | 7.3976 | 1.1290 | 0.0000 | 0.0000 |
| EPB41L4B | 1.3862 | 2.8035 | 1.0161 | 0.0027 | 0.0055 |
| PCDH7 | 7.4754 | 2.4891 | -1.5865 | 0.0000 | 0.0000 |
| SLC22A15 | 0.5927 | 1.2203 | 1.0419 | 0.0006 | 0.0014 |
| ALKAL2 | 0.8257 | 0.1756 | -2.2332 | 0.0000 | 0.0001 |
| UPK2 | 82.8237 | 336.4763 | 2.0224 | 0.0258 | 0.0400 |
| NCS1 | 33.5493 | 9.4274 | -1.8314 | 0.0000 | 0.0000 |
| FERMT2 | 21.9245 | 3.9030 | -2.4899 | 0.0000 | 0.0000 |
| IQCC | 1.0606 | 2.3993 | 1.1777 | 0.0000 | 0.0000 |
| CXCL2 | 23.8495 | 5.7667 | -2.0482 | 0.0000 | 0.0000 |
| PARPBP | 0.6571 | 2.2649 | 1.7853 | 0.0000 | 0.0000 |
| ISG15 | 37.9323 | 217.9782 | 2.5227 | 0.0000 | 0.0000 |
| DUSP2 | 113.9868 | 20.5291 | -2.4731 | 0.0042 | 0.0082 |
| TMEM255A | 0.9070 | 0.3037 | -1.5784 | 0.0000 | 0.0000 |
| KCNMB1 | 18.0526 | 1.4975 | -3.5916 | 0.0000 | 0.0000 |
| FBXO41 | 0.8743 | 2.6063 | 1.5758 | 0.0000 | 0.0000 |
| FAT4 | 1.5299 | 0.3470 | -2.1403 | 0.0000 | 0.0000 |
| ALKBH6 | 0.4714 | 1.2863 | 1.4482 | 0.0000 | 0.0000 |
| RBPMS2 | 38.8435 | 3.8981 | -3.3168 | 0.0000 | 0.0000 |
| RORA | 1.1972 | 0.5667 | -1.0789 | 0.0000 | 0.0000 |
| CHIT1 | 0.0713 | 0.9186 | 3.6884 | 0.0026 | 0.0054 |
| ZNF540 | 0.6474 | 0.2880 | -1.1687 | 0.0000 | 0.0000 |
| MYL9 | 1350.8951 | 113.1635 | -3.5774 | 0.0000 | 0.0000 |
| GPRASP2 | 4.6279 | 2.0237 | -1.1934 | 0.0000 | 0.0000 |
| C19orf57 | 0.6883 | 1.4796 | 1.1040 | 0.0077 | 0.0140 |
| ATP1A2 | 18.2931 | 0.8882 | -4.3642 | 0.0000 | 0.0000 |
| TSPAN10 | 0.3041 | 0.7184 | 1.2404 | 0.0004 | 0.0010 |
| OXER1 | 3.4926 | 1.1215 | -1.6389 | 0.0000 | 0.0000 |
| SPARCL1 | 255.4386 | 35.4317 | -2.8499 | 0.0000 | 0.0000 |
| FGFR1 | 13.6027 | 3.8221 | -1.8315 | 0.0000 | 0.0000 |
| MAGI2 | 1.5410 | 0.4777 | -1.6897 | 0.0000 | 0.0000 |
| LAMC3 | 9.5071 | 1.8495 | -2.3619 | 0.0000 | 0.0000 |
| FOXH1 | 0.0745 | 0.5254 | 2.8184 | 0.0000 | 0.0000 |
| JCAD | 11.4265 | 3.3371 | -1.7757 | 0.0004 | 0.0011 |
| DCLRE1B | 1.6902 | 3.4800 | 1.0419 | 0.0000 | 0.0000 |
| FCRLB | 0.9426 | 8.2515 | 3.1299 | 0.0000 | 0.0000 |
| CHRM3 | 3.1666 | 0.4312 | -2.8766 | 0.0003 | 0.0009 |
| CD24 | 102.0798 | 261.2155 | 1.3555 | 0.0119 | 0.0203 |
| C2orf15 | 0.7929 | 2.1243 | 1.4217 | 0.0000 | 0.0001 |
| ADAMTSL1 | 0.7911 | 0.3188 | -1.3115 | 0.0000 | 0.0000 |
| HIST1H1C | 21.7542 | 135.1818 | 2.6355 | 0.0000 | 0.0000 |
| ABI3BP | 9.2158 | 1.0625 | -3.1167 | 0.0000 | 0.0000 |
| SNAI3 | 1.8948 | 0.8876 | -1.0942 | 0.0045 | 0.0087 |
| HAUS5 | 3.2353 | 6.6431 | 1.0380 | 0.0000 | 0.0000 |
| MXRA7 | 23.3170 | 5.8429 | -1.9966 | 0.0000 | 0.0000 |
| SMIM10 | 9.6080 | 3.2407 | -1.5679 | 0.0000 | 0.0000 |
| PRKCB | 3.7175 | 0.9411 | -1.9820 | 0.0000 | 0.0000 |
| CGNL1 | 6.9594 | 0.8928 | -2.9625 | 0.0000 | 0.0000 |
| TTF2 | 1.3959 | 3.1158 | 1.1584 | 0.0000 | 0.0000 |
| CEP152 | 0.4104 | 1.0317 | 1.3300 | 0.0000 | 0.0000 |
| SOD3 | 52.9563 | 16.6384 | -1.6703 | 0.0000 | 0.0000 |
| JUNB | 607.7429 | 205.8415 | -1.5619 | 0.0000 | 0.0000 |
| COLGALT2 | 0.9391 | 0.3264 | -1.5245 | 0.0000 | 0.0000 |
| C21orf58 | 0.4050 | 2.5623 | 2.6615 | 0.0000 | 0.0000 |
| ZNF726 | 0.2925 | 1.1355 | 1.9567 | 0.0000 | 0.0000 |
| MAP1A | 10.1440 | 1.4698 | -2.7869 | 0.0000 | 0.0000 |
| ECM2 | 2.5239 | 1.0534 | -1.2606 | 0.0000 | 0.0001 |
| COX7A1 | 31.7541 | 5.7293 | -2.4705 | 0.0000 | 0.0000 |
| PID1 | 8.1046 | 1.0973 | -2.8848 | 0.0000 | 0.0000 |
| GDPD3 | 8.3824 | 21.1818 | 1.3374 | 0.0132 | 0.0222 |
| CHRM2 | 4.4491 | 0.2710 | -4.0372 | 0.0000 | 0.0000 |
| S1PR1 | 14.4835 | 4.4876 | -1.6904 | 0.0000 | 0.0000 |
| C1QTNF12 | 0.3219 | 1.4405 | 2.1620 | 0.0000 | 0.0001 |
| JAML | 2.3308 | 1.1641 | -1.0016 | 0.0062 | 0.0114 |
| BLOC1S3 | 3.8112 | 9.1505 | 1.2636 | 0.0000 | 0.0000 |
| ADCY5 | 8.8415 | 1.1371 | -2.9589 | 0.0000 | 0.0000 |
| TNFAIP6 | 9.5296 | 3.5964 | -1.4059 | 0.0028 | 0.0056 |
| KCP | 0.3101 | 1.0118 | 1.7062 | 0.0002 | 0.0007 |
| ACTG2 | 974.2562 | 61.9605 | -3.9749 | 0.0000 | 0.0000 |
| SGO1 | 0.5046 | 2.8024 | 2.4734 | 0.0000 | 0.0000 |
| CATSPERB | 0.1722 | 0.5001 | 1.5380 | 0.0014 | 0.0031 |
| CXCL10 | 7.5084 | 56.2206 | 2.9045 | 0.0017 | 0.0036 |
| MYLK2 | 0.0605 | 0.2096 | 1.7925 | 0.0000 | 0.0000 |
| BTG2 | 418.8302 | 103.6954 | -2.0140 | 0.0000 | 0.0000 |
| RASL10A | 0.2665 | 1.0667 | 2.0008 | 0.0004 | 0.0010 |
| SORBS2 | 11.5114 | 1.1795 | -3.2868 | 0.0000 | 0.0000 |
| FCER2 | 1.9640 | 0.7640 | -1.3622 | 0.0000 | 0.0000 |
| AP1S1 | 23.0931 | 48.2989 | 1.0645 | 0.0000 | 0.0000 |
| PPP1R14D | 0.1460 | 5.1916 | 5.1523 | 0.0000 | 0.0001 |
| TRIM17 | 0.4913 | 2.7662 | 2.4931 | 0.0000 | 0.0002 |
| PALB2 | 2.1036 | 4.5939 | 1.1269 | 0.0000 | 0.0000 |
| CUZD1 | 0.1889 | 0.4234 | 1.1648 | 0.0002 | 0.0006 |
| ZBTB4 | 29.7213 | 14.3050 | -1.0550 | 0.0000 | 0.0000 |
| CFAP45 | 0.6662 | 1.8333 | 1.4605 | 0.0032 | 0.0065 |
| KIFC2 | 3.2965 | 12.2521 | 1.8940 | 0.0000 | 0.0000 |
| HSPA2 | 13.5403 | 4.1431 | -1.7085 | 0.0000 | 0.0000 |
| CKAP2 | 3.7785 | 10.0630 | 1.4132 | 0.0000 | 0.0000 |
| LRFN1 | 0.3880 | 1.1019 | 1.5061 | 0.0016 | 0.0035 |
| MAGOHB | 2.0236 | 4.1754 | 1.0450 | 0.0000 | 0.0000 |
| EMP1 | 117.8845 | 19.2389 | -2.6153 | 0.0000 | 0.0000 |
| LPP | 30.4022 | 5.5540 | -2.4526 | 0.0000 | 0.0000 |
| BLCAP | 14.1564 | 28.4646 | 1.0077 | 0.0000 | 0.0001 |
| VIT | 5.3391 | 0.2556 | -4.3846 | 0.0000 | 0.0000 |
| EPSTI1 | 2.7344 | 8.1015 | 1.5670 | 0.0019 | 0.0041 |
| UCN2 | 0.4088 | 2.5171 | 2.6223 | 0.0000 | 0.0000 |
| DBF4 | 1.3328 | 3.7017 | 1.4737 | 0.0000 | 0.0000 |
| SNX10 | 1.3465 | 4.9202 | 1.8695 | 0.0000 | 0.0000 |
| DIP2C | 6.7022 | 3.1906 | -1.0708 | 0.0027 | 0.0056 |
| GJB7 | 0.2310 | 1.1781 | 2.3505 | 0.0000 | 0.0000 |
| MAMDC4 | 0.7322 | 1.9005 | 1.3761 | 0.0000 | 0.0001 |
| PARD3B | 2.9555 | 0.7072 | -2.0632 | 0.0000 | 0.0000 |
| SRD5A1 | 1.3361 | 3.4786 | 1.3805 | 0.0000 | 0.0000 |
| VCL | 54.8305 | 18.6138 | -1.5586 | 0.0001 | 0.0002 |
| EFNA1 | 18.6198 | 46.0832 | 1.3074 | 0.0000 | 0.0002 |
| SCGB3A2 | 0.0222 | 0.2485 | 3.4853 | 0.0018 | 0.0038 |
| ENO1 | 204.2592 | 433.7473 | 1.0865 | 0.0000 | 0.0000 |
| CMTM8 | 3.3311 | 7.3353 | 1.1388 | 0.0004 | 0.0009 |
| KISS1R | 0.0479 | 0.3873 | 3.0141 | 0.0002 | 0.0006 |
| IGFL2 | 0.0273 | 2.7466 | 6.6514 | 0.0000 | 0.0000 |
| BAIAP2L2 | 0.9628 | 3.3740 | 1.8092 | 0.0003 | 0.0009 |
| KIF15 | 0.7123 | 3.3413 | 2.2298 | 0.0000 | 0.0000 |
| PPM1K | 2.5968 | 1.2031 | -1.1100 | 0.0000 | 0.0000 |
| ATP13A2 | 6.9538 | 15.1312 | 1.1217 | 0.0000 | 0.0000 |
| PLXNA1 | 4.8155 | 13.9273 | 1.5322 | 0.0000 | 0.0000 |
| MAPK4 | 1.6966 | 0.4016 | -2.0789 | 0.0000 | 0.0000 |
| ODF3L1 | 2.6063 | 0.3645 | -2.8381 | 0.0000 | 0.0000 |
| TMEM44 | 1.9549 | 4.9925 | 1.3527 | 0.0000 | 0.0000 |
| PAMR1 | 14.9268 | 2.3028 | -2.6965 | 0.0000 | 0.0000 |
| SLC35F2 | 2.6977 | 7.0638 | 1.3887 | 0.0000 | 0.0000 |
| PLEKHG4B | 0.0578 | 0.9496 | 4.0392 | 0.0039 | 0.0076 |
| SLAMF9 | 0.1155 | 0.5587 | 2.2741 | 0.0051 | 0.0098 |
| PFDN6 | 7.7440 | 18.4938 | 1.2559 | 0.0000 | 0.0000 |
| AVPR2 | 1.0127 | 0.3434 | -1.5603 | 0.0000 | 0.0000 |
| KMO | 0.1295 | 0.3526 | 1.4447 | 0.0044 | 0.0085 |
| ZNF92 | 1.2303 | 3.3304 | 1.4367 | 0.0000 | 0.0000 |
| FCER1A | 5.5361 | 1.3074 | -2.0822 | 0.0000 | 0.0000 |
| FHAD1 | 0.1520 | 0.4259 | 1.4868 | 0.0067 | 0.0123 |
| FRMD3 | 1.8431 | 0.7314 | -1.3334 | 0.0000 | 0.0000 |
| NPIPB15 | 0.6648 | 3.3153 | 2.3180 | 0.0020 | 0.0043 |
| NKX2-5 | 0.0066 | 0.3739 | 5.8161 | 0.0039 | 0.0077 |
| LY6E | 52.7694 | 160.0548 | 1.6008 | 0.0003 | 0.0008 |
| NUDT1 | 6.2604 | 14.5194 | 1.2136 | 0.0000 | 0.0000 |
| GRK5 | 4.4441 | 2.1520 | -1.0462 | 0.0000 | 0.0000 |
| PRR11 | 1.6028 | 7.3050 | 2.1883 | 0.0000 | 0.0000 |
| MTBP | 0.2945 | 1.0614 | 1.8499 | 0.0000 | 0.0000 |
| SYNGR3 | 0.2334 | 0.7980 | 1.7736 | 0.0052 | 0.0098 |
| ADAM8 | 5.1819 | 15.2730 | 1.5594 | 0.0000 | 0.0000 |
| TACR1 | 1.0844 | 0.2394 | -2.1792 | 0.0000 | 0.0000 |
| C5orf46 | 0.0912 | 1.4056 | 3.9468 | 0.0000 | 0.0000 |
| METTL26 | 16.0112 | 32.7968 | 1.0345 | 0.0000 | 0.0000 |
| MATN2 | 28.5280 | 6.7675 | -2.0757 | 0.0000 | 0.0000 |
| MTHFD1L | 2.5939 | 8.0083 | 1.6264 | 0.0000 | 0.0000 |
| PRKG1 | 4.9162 | 0.8307 | -2.5651 | 0.0000 | 0.0000 |
| PPP1R12B | 35.8955 | 3.0145 | -3.5738 | 0.0000 | 0.0000 |
| SLC12A8 | 0.8355 | 3.9394 | 2.2373 | 0.0000 | 0.0000 |
| RGMA | 1.1701 | 0.4981 | -1.2320 | 0.0000 | 0.0000 |
| STARD9 | 1.2147 | 0.3022 | -2.0069 | 0.0000 | 0.0000 |
| ZNF606 | 0.8039 | 1.6638 | 1.0495 | 0.0000 | 0.0000 |
| FANCI | 2.2050 | 7.4308 | 1.7528 | 0.0000 | 0.0000 |
| ZWINT | 4.2229 | 22.7611 | 2.4302 | 0.0000 | 0.0000 |
| ARHGAP39 | 1.0299 | 3.6956 | 1.8434 | 0.0000 | 0.0000 |
| MRGPRF | 33.1131 | 4.7016 | -2.8162 | 0.0000 | 0.0000 |
| CPA3 | 19.0614 | 4.3839 | -2.1204 | 0.0000 | 0.0000 |
| KLK4 | 0.0601 | 0.7484 | 3.6382 | 0.0011 | 0.0026 |
| BGN | 66.7098 | 174.2259 | 1.3850 | 0.0329 | 0.0497 |
| PPP1R35 | 9.2147 | 20.9246 | 1.1832 | 0.0000 | 0.0000 |
| SLMAP | 21.4879 | 4.4391 | -2.2752 | 0.0000 | 0.0000 |
| SCNN1G | 4.5565 | 15.8466 | 1.7982 | 0.0288 | 0.0442 |
| DDIAS | 0.6081 | 2.2664 | 1.8981 | 0.0000 | 0.0000 |
| DOCK11 | 3.9405 | 1.7464 | -1.1740 | 0.0001 | 0.0002 |
| CNGB3 | 0.0456 | 0.2165 | 2.2472 | 0.0000 | 0.0000 |
| MAPK8IP2 | 0.2999 | 1.5623 | 2.3813 | 0.0003 | 0.0008 |
| TEX30 | 2.4259 | 5.7741 | 1.2511 | 0.0000 | 0.0000 |
| POU4F1 | 0.0198 | 0.2685 | 3.7642 | 0.0020 | 0.0043 |
| SYCP2 | 0.2050 | 1.1295 | 2.4623 | 0.0000 | 0.0001 |
| CDCA4 | 4.6471 | 14.7261 | 1.6640 | 0.0000 | 0.0000 |
| DCN | 116.9103 | 21.2226 | -2.4617 | 0.0000 | 0.0000 |
| GPR146 | 0.5547 | 0.2127 | -1.3828 | 0.0000 | 0.0000 |
| PPOX | 2.8053 | 5.9301 | 1.0799 | 0.0000 | 0.0000 |
| ACP6 | 1.6482 | 3.6189 | 1.1346 | 0.0008 | 0.0019 |
| DIO1 | 0.0593 | 0.4489 | 2.9213 | 0.0022 | 0.0046 |
| BRMS1 | 15.5841 | 31.4726 | 1.0140 | 0.0000 | 0.0000 |
| SELENOM | 40.5575 | 16.6588 | -1.2837 | 0.0000 | 0.0000 |
| OLFML1 | 6.2848 | 1.4269 | -2.1390 | 0.0000 | 0.0000 |
| LDLRAD2 | 1.1919 | 0.2806 | -2.0866 | 0.0000 | 0.0000 |
| SLC38A7 | 1.8503 | 4.7265 | 1.3530 | 0.0000 | 0.0000 |
| SHH | 24.0079 | 4.7686 | -2.3319 | 0.0061 | 0.0114 |
| HIST1H1E | 0.2444 | 6.9855 | 4.8369 | 0.0000 | 0.0000 |
| FYN | 9.5360 | 4.1923 | -1.1856 | 0.0000 | 0.0001 |
| COL6A2 | 278.1627 | 126.0648 | -1.1418 | 0.0000 | 0.0000 |
| CDH19 | 1.3633 | 0.2182 | -2.6434 | 0.0000 | 0.0000 |
| TCTEX1D2 | 2.2427 | 4.5799 | 1.0301 | 0.0002 | 0.0006 |
| NDN | 19.4908 | 8.5348 | -1.1914 | 0.0000 | 0.0000 |
| MVK | 2.9085 | 6.0877 | 1.0656 | 0.0000 | 0.0000 |
| FOXP2 | 1.7755 | 0.2744 | -2.6941 | 0.0000 | 0.0000 |
| PSPH | 2.8620 | 6.8658 | 1.2624 | 0.0000 | 0.0000 |
| DBNDD1 | 1.6035 | 5.7106 | 1.8324 | 0.0000 | 0.0000 |
| CDO1 | 2.3090 | 0.6760 | -1.7721 | 0.0000 | 0.0000 |
| CREBRF | 2.9267 | 1.4578 | -1.0055 | 0.0000 | 0.0001 |
| APOBEC3H | 0.6800 | 1.4434 | 1.0858 | 0.0143 | 0.0239 |
| MAGEA4 | 0.4133 | 9.0525 | 4.4530 | 0.0105 | 0.0181 |
| C17orf53 | 0.9063 | 3.6060 | 1.9924 | 0.0000 | 0.0000 |
| CHAF1A | 4.8222 | 11.3118 | 1.2301 | 0.0000 | 0.0000 |
| ZNF85 | 0.6652 | 1.5608 | 1.2305 | 0.0000 | 0.0000 |
| HIST1H3G | 0.0543 | 1.2647 | 4.5419 | 0.0000 | 0.0000 |
| TEAD4 | 3.2021 | 7.9313 | 1.3085 | 0.0006 | 0.0014 |
| MIDN | 66.8468 | 30.3484 | -1.1392 | 0.0002 | 0.0004 |
| FAM149A | 3.3340 | 1.0206 | -1.7079 | 0.0000 | 0.0000 |
| F8 | 1.9639 | 0.6770 | -1.5364 | 0.0000 | 0.0000 |
| CDC25C | 0.5412 | 2.7562 | 2.3484 | 0.0000 | 0.0000 |
| MID2 | 4.3109 | 2.1174 | -1.0257 | 0.0000 | 0.0000 |
| FILIP1 | 8.5744 | 0.5481 | -3.9675 | 0.0000 | 0.0000 |
| TPPP | 8.2561 | 0.9040 | -3.1910 | 0.0000 | 0.0000 |
| C17orf107 | 1.3699 | 0.6572 | -1.0596 | 0.0053 | 0.0100 |
| CYP26A1 | 0.0522 | 0.4495 | 3.1073 | 0.0012 | 0.0027 |
| TGFBR3 | 19.0421 | 5.0477 | -1.9155 | 0.0000 | 0.0000 |
| SOX17 | 7.1823 | 1.1079 | -2.6966 | 0.0000 | 0.0000 |
| FGF2 | 4.0673 | 0.7564 | -2.4269 | 0.0000 | 0.0000 |
| COL4A4 | 1.6802 | 0.4681 | -1.8439 | 0.0000 | 0.0000 |
| NXPH3 | 4.1480 | 0.7533 | -2.4611 | 0.0000 | 0.0000 |
| CPED1 | 9.9813 | 0.8383 | -3.5737 | 0.0000 | 0.0000 |
| DDX11 | 1.8061 | 4.8492 | 1.4249 | 0.0000 | 0.0000 |
| HSD17B6 | 12.3573 | 2.3431 | -2.3989 | 0.0000 | 0.0000 |
| NME1 | 9.8683 | 26.2519 | 1.4116 | 0.0000 | 0.0000 |
| RAD51AP1 | 1.3709 | 5.7449 | 2.0671 | 0.0000 | 0.0000 |
| FLAD1 | 7.2034 | 16.6421 | 1.2081 | 0.0000 | 0.0000 |
| MOGS | 14.6108 | 34.5129 | 1.2401 | 0.0000 | 0.0000 |
| SOBP | 4.5570 | 0.6107 | -2.8996 | 0.0000 | 0.0000 |
| NTN1 | 4.2323 | 1.6043 | -1.3995 | 0.0000 | 0.0000 |
| MAD2L2 | 5.8789 | 13.5113 | 1.2005 | 0.0000 | 0.0000 |
| HOXB3 | 2.3062 | 4.7573 | 1.0446 | 0.0034 | 0.0068 |
| ARHGEF37 | 6.4366 | 1.9925 | -1.6917 | 0.0000 | 0.0001 |
| CNKSR1 | 5.1608 | 12.5805 | 1.2855 | 0.0000 | 0.0000 |
| PITX2 | 4.3674 | 1.2779 | -1.7730 | 0.0000 | 0.0001 |
| GFY | 0.0084 | 0.3221 | 5.2572 | 0.0001 | 0.0002 |
| EMCN | 5.3618 | 1.6413 | -1.7079 | 0.0000 | 0.0000 |
| ARID5B | 24.4674 | 7.8668 | -1.6370 | 0.0000 | 0.0000 |
| PSRC1 | 1.4449 | 5.0637 | 1.8092 | 0.0000 | 0.0000 |
| SCARB1 | 3.6579 | 7.8400 | 1.0999 | 0.0001 | 0.0002 |
| DHRS13 | 1.7751 | 4.3168 | 1.2820 | 0.0001 | 0.0003 |
| RASGRP2 | 4.3983 | 0.9698 | -2.1811 | 0.0000 | 0.0000 |
| ERC1 | 7.5656 | 3.7527 | -1.0115 | 0.0001 | 0.0004 |
| SELP | 5.4792 | 1.7833 | -1.6194 | 0.0000 | 0.0000 |
| RNASE7 | 0.3483 | 3.2549 | 3.2240 | 0.0002 | 0.0007 |
| MEF2C | 4.3109 | 1.3759 | -1.6476 | 0.0000 | 0.0000 |
| PMFBP1 | 0.1424 | 0.2955 | 1.0535 | 0.0083 | 0.0149 |
| SLC9A9 | 6.3020 | 1.9886 | -1.6641 | 0.0000 | 0.0000 |
| CEND1 | 0.9802 | 0.4013 | -1.2883 | 0.0000 | 0.0000 |
| MYLK | 75.2987 | 5.0543 | -3.8970 | 0.0000 | 0.0000 |
| CBX2 | 1.0055 | 5.0579 | 2.3306 | 0.0000 | 0.0000 |
| XRCC2 | 0.3249 | 1.9087 | 2.5544 | 0.0000 | 0.0000 |
| ADGRE2 | 0.3469 | 1.0743 | 1.6309 | 0.0002 | 0.0006 |
| PLK3 | 17.4456 | 8.1345 | -1.1007 | 0.0078 | 0.0140 |
| NCAPD3 | 2.0357 | 4.5257 | 1.1526 | 0.0000 | 0.0000 |
| AC008687.4 | 0.0542 | 0.4438 | 3.0338 | 0.0000 | 0.0000 |
| SFXN2 | 0.8303 | 1.7159 | 1.0473 | 0.0000 | 0.0001 |
| ZNF43 | 1.2324 | 2.6303 | 1.0938 | 0.0011 | 0.0024 |
| MISP3 | 1.9659 | 4.3103 | 1.1326 | 0.0076 | 0.0138 |
| DCHS1 | 6.6860 | 1.7844 | -1.9057 | 0.0000 | 0.0000 |
| CASP5 | 0.0456 | 0.2448 | 2.4251 | 0.0205 | 0.0328 |
| CELSR3 | 0.2962 | 2.0423 | 2.7856 | 0.0000 | 0.0000 |
| DHDH | 0.6520 | 1.8897 | 1.5352 | 0.0032 | 0.0064 |
| C4B | 2.3484 | 0.9927 | -1.2423 | 0.0001 | 0.0002 |
| SCARA5 | 26.6699 | 0.8451 | -4.9800 | 0.0000 | 0.0000 |
| EPOP | 2.9497 | 7.0739 | 1.2619 | 0.0004 | 0.0011 |
| DNAJB11 | 8.0534 | 17.4076 | 1.1120 | 0.0000 | 0.0000 |
| DNASE1L3 | 2.5072 | 0.9697 | -1.3704 | 0.0000 | 0.0000 |
| ATP6V0A4 | 0.1726 | 1.1514 | 2.7379 | 0.0019 | 0.0041 |
| TMEM201 | 2.3258 | 4.6805 | 1.0089 | 0.0000 | 0.0000 |
| BORA | 0.7996 | 2.7378 | 1.7756 | 0.0000 | 0.0000 |
| ISYNA1 | 14.6921 | 37.8108 | 1.3638 | 0.0008 | 0.0019 |
| PI16 | 96.4248 | 2.3062 | -5.3858 | 0.0000 | 0.0000 |
| CCDC136 | 2.2608 | 0.5416 | -2.0614 | 0.0000 | 0.0002 |
| PARP14 | 5.2151 | 12.5752 | 1.2698 | 0.0000 | 0.0000 |
| 44442.0000 | 0.3252 | 1.7914 | 2.4618 | 0.0000 | 0.0001 |
| PBXIP1 | 88.9699 | 43.3490 | -1.0373 | 0.0000 | 0.0000 |
| GLIPR2 | 14.8931 | 6.8634 | -1.1176 | 0.0000 | 0.0001 |
| MIF | 26.5983 | 62.3548 | 1.2292 | 0.0000 | 0.0000 |
| NECAB1 | 2.9780 | 0.4241 | -2.8119 | 0.0000 | 0.0000 |
| BCL2L1 | 22.8266 | 46.4609 | 1.0253 | 0.0000 | 0.0000 |
| CCM2L | 1.7944 | 0.8727 | -1.0400 | 0.0000 | 0.0001 |
| ENPP2 | 7.8667 | 3.8827 | -1.0187 | 0.0000 | 0.0000 |
| ZGRF1 | 0.2889 | 0.8887 | 1.6214 | 0.0000 | 0.0000 |
| SLC30A3 | 0.0310 | 0.2361 | 2.9305 | 0.0083 | 0.0148 |
| KIF14 | 0.4380 | 2.1471 | 2.2934 | 0.0000 | 0.0000 |
| OPN3 | 1.0297 | 2.2102 | 1.1019 | 0.0030 | 0.0061 |
| BMP8A | 0.1575 | 0.4372 | 1.4734 | 0.0000 | 0.0000 |
| WDCP | 1.5976 | 3.3457 | 1.0664 | 0.0000 | 0.0000 |
| TCF21 | 11.7172 | 0.7302 | -4.0043 | 0.0000 | 0.0000 |
| PDZRN3 | 12.4546 | 1.8735 | -2.7329 | 0.0000 | 0.0000 |
| MTG1 | 1.0030 | 2.6863 | 1.4212 | 0.0000 | 0.0000 |
| PIGU | 9.5746 | 20.4234 | 1.0929 | 0.0000 | 0.0000 |
| OTOF | 0.0476 | 0.4713 | 3.3067 | 0.0000 | 0.0001 |
| MBOAT7 | 20.1027 | 46.6765 | 1.2153 | 0.0000 | 0.0000 |
| GLI1 | 1.1659 | 0.4190 | -1.4764 | 0.0000 | 0.0000 |
| DDTL | 0.8725 | 1.8289 | 1.0677 | 0.0001 | 0.0002 |
| C12orf56 | 0.2075 | 0.5234 | 1.3345 | 0.0088 | 0.0156 |
| DUSP1 | 795.2293 | 103.3641 | -2.9436 | 0.0000 | 0.0000 |
| OMD | 3.7842 | 0.5741 | -2.7206 | 0.0000 | 0.0000 |
| CAVIN1 | 221.9331 | 67.4804 | -1.7176 | 0.0000 | 0.0000 |
| PRDM8 | 2.6068 | 0.8681 | -1.5864 | 0.0000 | 0.0000 |
| RBFOX3 | 6.0433 | 0.2128 | -4.8280 | 0.0000 | 0.0000 |
| TPM1 | 144.5294 | 16.7516 | -3.1090 | 0.0000 | 0.0000 |
| GINS2 | 2.3206 | 7.4263 | 1.6782 | 0.0000 | 0.0000 |
| CENPN | 2.2983 | 4.8545 | 1.0788 | 0.0000 | 0.0000 |
| GRAMD2A | 0.2112 | 1.1806 | 2.4828 | 0.0161 | 0.0264 |
| GINS3 | 1.0250 | 2.3163 | 1.1762 | 0.0000 | 0.0000 |
| ZNF883 | 1.4090 | 3.0368 | 1.1079 | 0.0261 | 0.0404 |
| OR7E47P | 2.0470 | 0.5965 | -1.7788 | 0.0000 | 0.0000 |
| ZDHHC24 | 2.2197 | 4.7982 | 1.1121 | 0.0000 | 0.0000 |
| ACY1 | 0.9871 | 2.3740 | 1.2661 | 0.0000 | 0.0000 |
| CRY2 | 11.5349 | 4.1537 | -1.4735 | 0.0000 | 0.0000 |
| BMP5 | 5.9841 | 0.6171 | -3.2775 | 0.0000 | 0.0000 |
| HIST1H2AD | 0.1526 | 4.2337 | 4.7943 | 0.0000 | 0.0000 |
| DSTN | 322.9235 | 100.3794 | -1.6857 | 0.0000 | 0.0000 |
| SOX9 | 3.6892 | 11.6925 | 1.6642 | 0.0046 | 0.0088 |
| ITGA8 | 10.5044 | 1.1345 | -3.2109 | 0.0000 | 0.0000 |
| SHE | 1.4200 | 0.4914 | -1.5310 | 0.0000 | 0.0000 |
| SAPCD2 | 1.4073 | 8.8351 | 2.6503 | 0.0000 | 0.0000 |
| CTU1 | 1.0741 | 2.7891 | 1.3767 | 0.0000 | 0.0000 |
| GKN1 | 2.4879 | 1.2068 | -1.0437 | 0.0302 | 0.0459 |
| TMEM151A | 0.0292 | 0.2325 | 2.9934 | 0.0005 | 0.0012 |
| MXD3 | 0.9921 | 2.7073 | 1.4483 | 0.0000 | 0.0000 |
| NEB | 0.0744 | 0.2793 | 1.9077 | 0.0081 | 0.0145 |
| SERPINF1 | 69.7480 | 33.8171 | -1.0444 | 0.0000 | 0.0000 |
| FAAP24 | 1.1687 | 3.2545 | 1.4775 | 0.0000 | 0.0000 |
| SELE | 14.7565 | 2.8633 | -2.3656 | 0.0000 | 0.0001 |
| CFAP53 | 0.4431 | 1.2384 | 1.4828 | 0.0001 | 0.0002 |
| CXorf57 | 1.7585 | 4.6577 | 1.4053 | 0.0089 | 0.0158 |
| ZWILCH | 2.0752 | 5.1766 | 1.3187 | 0.0000 | 0.0000 |
| CCNL2 | 12.0639 | 28.1334 | 1.2216 | 0.0000 | 0.0000 |
| CDC42EP3 | 12.5634 | 4.6899 | -1.4216 | 0.0002 | 0.0004 |
| HIST1H3F | 0.0502 | 2.4856 | 5.6306 | 0.0000 | 0.0000 |
| ARHGEF15 | 3.5160 | 1.7004 | -1.0481 | 0.0000 | 0.0000 |
| STK32C | 2.1458 | 5.4377 | 1.3415 | 0.0000 | 0.0000 |
| CNTNAP1 | 3.3795 | 1.6101 | -1.0696 | 0.0000 | 0.0001 |
| MND1 | 1.2492 | 3.5873 | 1.5219 | 0.0000 | 0.0000 |
| ITGA9 | 4.9866 | 1.2785 | -1.9636 | 0.0000 | 0.0000 |
| PARP12 | 4.2400 | 10.7155 | 1.3376 | 0.0000 | 0.0000 |
| PDX1 | 0.0388 | 0.5069 | 3.7059 | 0.0003 | 0.0009 |
| APBB1 | 8.6528 | 2.5027 | -1.7897 | 0.0000 | 0.0000 |
| MYO19 | 1.6472 | 4.6721 | 1.5041 | 0.0000 | 0.0000 |
| IL16 | 2.0556 | 0.8678 | -1.2442 | 0.0000 | 0.0000 |
| GLCCI1 | 1.6364 | 3.4981 | 1.0961 | 0.0012 | 0.0027 |
| SRF | 45.7378 | 16.5927 | -1.4628 | 0.0000 | 0.0000 |
| PTGES3L | 1.3541 | 0.2790 | -2.2791 | 0.0000 | 0.0001 |
| HSPA12A | 2.1109 | 0.6254 | -1.7551 | 0.0000 | 0.0000 |
| ALB | 0.0186 | 3.0062 | 7.3389 | 0.0271 | 0.0418 |
| RMI2 | 3.5246 | 11.0376 | 1.6469 | 0.0000 | 0.0000 |
| HELLS | 0.7499 | 2.6347 | 1.8128 | 0.0000 | 0.0000 |
| ARHGAP11A | 1.4339 | 4.4962 | 1.6488 | 0.0000 | 0.0000 |
| HIST3H2A | 3.9700 | 22.6331 | 2.5112 | 0.0000 | 0.0000 |
| C6orf52 | 0.4328 | 1.3510 | 1.6424 | 0.0000 | 0.0001 |
| KCNS3 | 3.1264 | 6.7208 | 1.1041 | 0.0087 | 0.0154 |
| BEAN1 | 0.1034 | 0.4392 | 2.0872 | 0.0107 | 0.0185 |
| RNF150 | 4.7315 | 0.5255 | -3.1706 | 0.0000 | 0.0000 |
| RAD9A | 3.3014 | 7.8401 | 1.2478 | 0.0000 | 0.0000 |
| RHBDF2 | 3.0384 | 7.8350 | 1.3666 | 0.0000 | 0.0000 |
| CHEK1 | 1.2286 | 3.9593 | 1.6882 | 0.0000 | 0.0000 |
| ATL1 | 2.9468 | 1.3719 | -1.1029 | 0.0000 | 0.0000 |
| HSD17B1 | 0.4369 | 2.4407 | 2.4819 | 0.0000 | 0.0000 |
| RBL1 | 1.3311 | 3.4389 | 1.3693 | 0.0000 | 0.0000 |
| TOR2A | 2.3957 | 4.9635 | 1.0509 | 0.0000 | 0.0000 |
| GALNT15 | 3.9485 | 0.7246 | -2.4460 | 0.0000 | 0.0000 |
| EPM2A | 3.9545 | 0.7911 | -2.3216 | 0.0000 | 0.0000 |
| TNS1 | 114.6837 | 7.4446 | -3.9453 | 0.0000 | 0.0000 |
| ZNF74 | 1.4952 | 3.3754 | 1.1748 | 0.0000 | 0.0000 |
| HIST1H2AG | 0.2251 | 1.9465 | 3.1125 | 0.0000 | 0.0000 |
| SNRPB | 97.4091 | 231.7206 | 1.2503 | 0.0000 | 0.0000 |
| TBX4 | 4.2272 | 0.6663 | -2.6654 | 0.0006 | 0.0014 |
| HSD17B7 | 1.4702 | 3.1281 | 1.0893 | 0.0000 | 0.0000 |
| CSKMT | 0.7465 | 1.6680 | 1.1599 | 0.0000 | 0.0000 |
| CCL23 | 1.6623 | 0.6514 | -1.3516 | 0.0000 | 0.0000 |
| FAM50B | 5.4559 | 2.7000 | -1.0149 | 0.0000 | 0.0000 |
| ALG3 | 8.9872 | 22.7532 | 1.3401 | 0.0000 | 0.0000 |
| PFKFB1 | 0.1270 | 0.2628 | 1.0488 | 0.0000 | 0.0001 |
| TPM2 | 433.3469 | 61.3989 | -2.8192 | 0.0000 | 0.0000 |
| RAD51 | 1.3505 | 5.4198 | 2.0048 | 0.0000 | 0.0000 |
| TLN1 | 101.7212 | 30.7278 | -1.7270 | 0.0001 | 0.0002 |
| ASB12 | 0.4157 | 0.1951 | -1.0915 | 0.0002 | 0.0006 |
| BCO2 | 0.7346 | 0.2563 | -1.5189 | 0.0000 | 0.0000 |
| GCKR | 0.0722 | 0.3978 | 2.4618 | 0.0291 | 0.0446 |
| KNTC1 | 0.9340 | 3.4615 | 1.8899 | 0.0000 | 0.0000 |
| UBE2C | 10.4487 | 70.0631 | 2.7453 | 0.0000 | 0.0000 |
| SNTA1 | 20.6541 | 10.2669 | -1.0084 | 0.0003 | 0.0008 |
| ATP6V1B1 | 0.2344 | 2.0164 | 3.1046 | 0.0012 | 0.0026 |
| TRIB3 | 3.1023 | 11.1202 | 1.8418 | 0.0000 | 0.0000 |
| LHB | 0.2126 | 0.8816 | 2.0522 | 0.0000 | 0.0000 |
| POLQ | 0.2772 | 1.6614 | 2.5832 | 0.0000 | 0.0000 |
| CIT | 0.7260 | 2.4719 | 1.7676 | 0.0000 | 0.0000 |
| CLEC3A | 4.8239 | 0.4471 | -3.4316 | 0.0000 | 0.0000 |
| EPHB2 | 0.9685 | 3.3161 | 1.7757 | 0.0000 | 0.0001 |
| H2AFX | 18.5647 | 58.7116 | 1.6611 | 0.0000 | 0.0000 |
| ARHGAP33 | 1.2693 | 2.7818 | 1.1320 | 0.0003 | 0.0008 |
| HM13 | 11.8095 | 24.0945 | 1.0288 | 0.0000 | 0.0000 |
| C3 | 111.3366 | 40.2152 | -1.4691 | 0.0000 | 0.0000 |
| KIF12 | 0.1699 | 1.0993 | 2.6937 | 0.0000 | 0.0001 |
| RECQL4 | 2.6213 | 12.2632 | 2.2260 | 0.0000 | 0.0000 |
| ERFE | 0.1444 | 0.4366 | 1.5966 | 0.0004 | 0.0011 |
| ZNF154 | 1.3252 | 0.4816 | -1.4603 | 0.0000 | 0.0000 |
| LPAR2 | 6.8015 | 14.0816 | 1.0499 | 0.0001 | 0.0002 |
| CENPS-CORT | 0.1790 | 0.3649 | 1.0279 | 0.0000 | 0.0000 |
| SOWAHC | 31.1274 | 12.2419 | -1.3464 | 0.0008 | 0.0019 |
| CCDC58 | 5.3243 | 12.0271 | 1.1756 | 0.0000 | 0.0000 |
| GRIN2D | 0.4126 | 5.2278 | 3.6633 | 0.0000 | 0.0000 |
| GSDMB | 4.7623 | 9.5577 | 1.0050 | 0.0078 | 0.0140 |
| SGPL1 | 13.9738 | 28.1336 | 1.0096 | 0.0002 | 0.0007 |
| ANO9 | 2.7935 | 5.6789 | 1.0235 | 0.0005 | 0.0012 |
| TMEM241 | 0.9864 | 2.0501 | 1.0555 | 0.0000 | 0.0001 |
| PAEP | 0.0067 | 0.8757 | 7.0394 | 0.0000 | 0.0001 |
| TMPO | 6.8155 | 15.6696 | 1.2011 | 0.0000 | 0.0000 |
| TRIB1 | 86.8644 | 27.0911 | -1.6809 | 0.0003 | 0.0008 |
| ADAMTS12 | 0.4768 | 1.7775 | 1.8985 | 0.0037 | 0.0074 |
| GPR78 | 0.1982 | 1.2957 | 2.7088 | 0.0003 | 0.0008 |
| TBX5 | 2.6686 | 0.4112 | -2.6980 | 0.0000 | 0.0000 |
| C4orf46 | 1.6533 | 3.4790 | 1.0733 | 0.0000 | 0.0000 |
| PTPRN2 | 3.6661 | 1.1326 | -1.6947 | 0.0000 | 0.0000 |
| PI3 | 190.7877 | 464.9974 | 1.2853 | 0.0043 | 0.0083 |
| FAM180A | 1.8817 | 0.3462 | -2.4425 | 0.0000 | 0.0000 |
| SPC24 | 1.8967 | 7.7182 | 2.0248 | 0.0000 | 0.0000 |
| LCN12 | 0.1119 | 0.4975 | 2.1520 | 0.0002 | 0.0006 |
| SAMD1 | 15.1032 | 31.3085 | 1.0517 | 0.0000 | 0.0000 |
| RFC5 | 3.5453 | 8.6663 | 1.2895 | 0.0000 | 0.0000 |
| WRAP73 | 2.0979 | 4.5473 | 1.1161 | 0.0000 | 0.0000 |
| CD1E | 0.8296 | 0.3962 | -1.0660 | 0.0068 | 0.0124 |
| EPYC | 0.0171 | 0.9704 | 5.8227 | 0.0053 | 0.0100 |
| NCAPG | 1.1116 | 5.3634 | 2.2705 | 0.0000 | 0.0000 |
| FAM184A | 0.6803 | 0.3359 | -1.0181 | 0.0001 | 0.0003 |
| KRTAP2-3 | 0.0090 | 0.4005 | 5.4817 | 0.0042 | 0.0082 |
| NTNG1 | 1.0315 | 0.1974 | -2.3855 | 0.0000 | 0.0000 |
| CA3 | 1.2248 | 0.3971 | -1.6251 | 0.0000 | 0.0000 |
| TMEM262 | 0.1659 | 0.3821 | 1.2037 | 0.0000 | 0.0000 |
| DOK6 | 2.4934 | 0.3049 | -3.0319 | 0.0000 | 0.0000 |
| NFIL3 | 49.8986 | 15.3970 | -1.6963 | 0.0000 | 0.0000 |
| PLAC9 | 23.5158 | 3.1922 | -2.8810 | 0.0000 | 0.0000 |
| EEF1E1 | 3.6289 | 7.7816 | 1.1005 | 0.0000 | 0.0000 |
| ATAD3C | 0.9174 | 2.0110 | 1.1323 | 0.0245 | 0.0382 |
| HCN2 | 0.1096 | 0.3126 | 1.5120 | 0.0013 | 0.0029 |
| IFIT3 | 6.0836 | 22.5590 | 1.8907 | 0.0037 | 0.0073 |
| ANAPC11 | 13.0798 | 26.4081 | 1.0136 | 0.0000 | 0.0000 |
| TMUB1 | 15.1581 | 33.8974 | 1.1611 | 0.0000 | 0.0000 |
| PDXK | 7.4851 | 18.1102 | 1.2747 | 0.0000 | 0.0000 |
| NT5DC2 | 10.1918 | 25.1465 | 1.3029 | 0.0000 | 0.0000 |
| NETO2 | 1.3190 | 3.4198 | 1.3744 | 0.0007 | 0.0016 |
