# Supplementary material for: Prognostic Value of Vascular-Expressed PSMA and CD248 in Urothelial Carcinoma of the Bladder
Source: Front Oncol. 2021 Nov 17;11:771036. doi: 10.3389/fonc.2021.771036 (PMC8635966; doi:10.3389/fonc.2021.771036)
Supplement: Supplementary file 2 [file DataSheet_1.zip › Supporting Data 2.DOCX]

**Supporting data 2. Cor-DEGs list of TCGA-BLCA dataset**

| **Gene** | **ConMean** | **TreatMean** | **logFC** | ***P*** | **FDR** |
| --- | --- | --- | --- | --- | --- |
| CD248 | 45.3202 | 17.6300 | -1.3621 | 0.0000 | 0.0000 |
| EMILIN1 | 75.2884 | 22.9546 | -1.7136 | 0.0000 | 0.0000 |
| LRRC32 | 14.5676 | 7.1735 | -1.0220 | 0.0000 | 0.0001 |
| COL6A2 | 278.1627 | 126.0648 | -1.1418 | 0.0000 | 0.0000 |
| OLFML1 | 6.2848 | 1.4269 | -2.1390 | 0.0000 | 0.0000 |
| HSPA12B | 3.9343 | 1.8447 | -1.0928 | 0.0000 | 0.0000 |
| BGN | 66.7098 | 174.2259 | 1.3850 | 0.0329 | 0.0497 |
| ANGPTL2 | 31.9755 | 13.0242 | -1.2958 | 0.0000 | 0.0001 |
| TIMP2 | 86.8500 | 42.5270 | -1.0301 | 0.0000 | 0.0001 |
| SSC5D | 7.5285 | 1.9826 | -1.9249 | 0.0000 | 0.0000 |
| ANXA6 | 70.7691 | 17.1612 | -2.0440 | 0.0000 | 0.0000 |
| ACTA2 | 864.0475 | 104.9944 | -3.0408 | 0.0000 | 0.0000 |
| TAGLN | 743.6427 | 94.6789 | -2.9735 | 0.0000 | 0.0000 |
| COX7A1 | 31.7541 | 5.7293 | -2.4705 | 0.0000 | 0.0000 |
| LHFPL6 | 26.8588 | 10.1413 | -1.4052 | 0.0000 | 0.0000 |
| CTGF | 424.0402 | 63.9133 | -2.7300 | 0.0000 | 0.0000 |
| MYL9 | 1350.8951 | 113.1635 | -3.5774 | 0.0000 | 0.0000 |
| TGFB3 | 10.5770 | 4.6773 | -1.1772 | 0.0000 | 0.0000 |
| HIC1 | 2.9029 | 1.2218 | -1.2484 | 0.0000 | 0.0000 |
| FBN1 | 11.8966 | 4.5259 | -1.3943 | 0.0007 | 0.0017 |
| ADGRA2 | 18.8081 | 4.2999 | -2.1290 | 0.0000 | 0.0000 |
| VSTM4 | 5.7763 | 1.1733 | -2.2995 | 0.0000 | 0.0000 |
| OLFML3 | 36.4901 | 11.6032 | -1.6530 | 0.0000 | 0.0000 |
| DCN | 116.9103 | 21.2226 | -2.4617 | 0.0000 | 0.0000 |
| FBXL7 | 6.8347 | 1.3582 | -2.3312 | 0.0000 | 0.0000 |
| CALD1 | 155.8522 | 22.2860 | -2.8060 | 0.0000 | 0.0001 |
| HEPH | 7.0685 | 2.0962 | -1.7536 | 0.0000 | 0.0000 |
| GYPC | 16.5086 | 4.4520 | -1.8907 | 0.0000 | 0.0000 |
| FILIP1L | 30.6159 | 6.1277 | -2.3209 | 0.0000 | 0.0000 |
| CCDC80 | 36.8499 | 6.6151 | -2.4778 | 0.0000 | 0.0000 |
| LAMA4 | 14.7250 | 5.8627 | -1.3286 | 0.0011 | 0.0026 |
| EDNRA | 9.7135 | 2.7926 | -1.7984 | 0.0000 | 0.0000 |
| HSPB2 | 2.3281 | 0.5286 | -2.1388 | 0.0000 | 0.0000 |
| MRGPRF | 33.1131 | 4.7016 | -2.8162 | 0.0000 | 0.0000 |
| KCNE4 | 14.3569 | 1.6295 | -3.1393 | 0.0000 | 0.0000 |
| TMEM119 | 17.3362 | 5.8088 | -1.5775 | 0.0000 | 0.0001 |
| FBLN5 | 20.9898 | 4.4483 | -2.2384 | 0.0000 | 0.0000 |
| TPM2 | 433.3469 | 61.3989 | -2.8192 | 0.0000 | 0.0000 |
| SERPINF1 | 69.7480 | 33.8171 | -1.0444 | 0.0000 | 0.0000 |
| MSRB3 | 40.9359 | 4.4014 | -3.2173 | 0.0000 | 0.0000 |
| TNFAIP6 | 9.5296 | 3.5964 | -1.4059 | 0.0028 | 0.0056 |
| CAVIN1 | 221.9331 | 67.4804 | -1.7176 | 0.0000 | 0.0000 |
| KCNJ8 | 7.2736 | 2.5578 | -1.5078 | 0.0000 | 0.0001 |
| ZNF521 | 2.1093 | 0.9252 | -1.1889 | 0.0000 | 0.0000 |
| SFRP2 | 189.2179 | 67.5484 | -1.4861 | 0.0000 | 0.0000 |
| TGFB1I1 | 25.6236 | 6.3967 | -2.0021 | 0.0000 | 0.0000 |
| PDLIM3 | 31.8033 | 4.4032 | -2.8525 | 0.0000 | 0.0000 |
| CRISPLD2 | 30.7942 | 8.0197 | -1.9410 | 0.0000 | 0.0000 |
| MEDAG | 16.2647 | 3.7661 | -2.1106 | 0.0000 | 0.0000 |
| DDR2 | 11.3594 | 2.0941 | -2.4395 | 0.0000 | 0.0000 |
| PODN | 27.2646 | 5.9080 | -2.2063 | 0.0000 | 0.0000 |
| RHOJ | 7.2028 | 2.2307 | -1.6911 | 0.0000 | 0.0000 |
| GNG11 | 18.9728 | 6.2134 | -1.6105 | 0.0000 | 0.0000 |
| IFFO1 | 3.1586 | 1.5327 | -1.0432 | 0.0000 | 0.0001 |
| TWIST2 | 5.1498 | 1.5414 | -1.7403 | 0.0000 | 0.0000 |
| GPC6 | 4.9528 | 1.8322 | -1.4347 | 0.0000 | 0.0000 |
| COL14A1 | 30.0335 | 4.6672 | -2.6860 | 0.0000 | 0.0000 |
| PLPP7 | 2.2186 | 0.7657 | -1.5349 | 0.0000 | 0.0000 |
| LMCD1 | 12.7915 | 3.7551 | -1.7683 | 0.0000 | 0.0000 |
| ADAMTS4 | 19.0108 | 3.1275 | -2.6037 | 0.0002 | 0.0006 |
| CYR61 | 459.5135 | 68.6112 | -2.7436 | 0.0000 | 0.0000 |
| CNRIP1 | 5.8094 | 1.8663 | -1.6382 | 0.0000 | 0.0000 |
| LIMS2 | 23.4183 | 2.6994 | -3.1169 | 0.0000 | 0.0000 |
| RASL12 | 22.8709 | 3.3649 | -2.7649 | 0.0000 | 0.0000 |
| C11orf96 | 109.7391 | 13.7758 | -2.9939 | 0.0000 | 0.0000 |
| MRVI1 | 20.5796 | 3.0158 | -2.7706 | 0.0000 | 0.0000 |
| CPXM2 | 21.2445 | 3.4458 | -2.6242 | 0.0000 | 0.0000 |
| TCEAL7 | 6.8890 | 1.4009 | -2.2979 | 0.0000 | 0.0000 |
| FERMT2 | 21.9245 | 3.9030 | -2.4899 | 0.0000 | 0.0000 |
| ITGA5 | 91.9836 | 20.4919 | -2.1663 | 0.0000 | 0.0000 |
| CTHRC1 | 9.7606 | 31.6084 | 1.6953 | 0.0207 | 0.0330 |
| JAM3 | 19.5551 | 3.5410 | -2.4653 | 0.0000 | 0.0000 |
| MYADM | 110.1005 | 24.1477 | -2.1889 | 0.0000 | 0.0000 |
| SH3RF3 | 2.9453 | 1.3477 | -1.1279 | 0.0000 | 0.0000 |
| COLEC12 | 7.1333 | 1.9919 | -1.8404 | 0.0000 | 0.0000 |
| GEM | 47.7413 | 6.3662 | -2.9067 | 0.0000 | 0.0000 |
| ECM2 | 2.5239 | 1.0534 | -1.2606 | 0.0000 | 0.0001 |
| FGFR1 | 13.6027 | 3.8221 | -1.8315 | 0.0000 | 0.0000 |
| FBLN2 | 59.4174 | 13.6262 | -2.1245 | 0.0000 | 0.0000 |
| NRP2 | 8.3073 | 3.1699 | -1.3900 | 0.0006 | 0.0014 |
| MFAP4 | 236.6482 | 29.5257 | -3.0027 | 0.0000 | 0.0000 |
| DPT | 59.0380 | 6.2962 | -3.2291 | 0.0000 | 0.0000 |
| GAS7 | 7.1781 | 1.9063 | -1.9128 | 0.0000 | 0.0000 |
| CXCL12 | 22.2139 | 6.4569 | -1.7825 | 0.0000 | 0.0000 |
| SCN1B | 2.3436 | 0.9963 | -1.2341 | 0.0000 | 0.0000 |
| MCAM | 43.7821 | 13.6123 | -1.6854 | 0.0001 | 0.0002 |
| ADAM12 | 1.2223 | 3.4588 | 1.5007 | 0.0147 | 0.0244 |
| SPON1 | 28.6916 | 6.2084 | -2.2083 | 0.0000 | 0.0000 |
| CD34 | 16.3648 | 4.4018 | -1.8944 | 0.0000 | 0.0000 |
| GLIPR2 | 14.8931 | 6.8634 | -1.1176 | 0.0000 | 0.0001 |
| FGF7 | 7.9917 | 1.6394 | -2.2854 | 0.0000 | 0.0000 |
| MFAP5 | 17.1103 | 3.5165 | -2.2827 | 0.0000 | 0.0000 |
| ECSCR | 6.8016 | 2.5324 | -1.4254 | 0.0000 | 0.0000 |
| CNN1 | 670.6525 | 34.5311 | -4.2796 | 0.0000 | 0.0000 |
| ZCCHC24 | 23.1641 | 4.3679 | -2.4069 | 0.0000 | 0.0000 |
| SGCD | 3.8585 | 0.5100 | -2.9195 | 0.0000 | 0.0000 |
| PECAM1 | 27.0390 | 13.0303 | -1.0532 | 0.0000 | 0.0001 |
| SOD3 | 52.9563 | 16.6384 | -1.6703 | 0.0000 | 0.0000 |
| PDLIM7 | 52.0123 | 19.0958 | -1.4456 | 0.0004 | 0.0010 |
| KIAA1755 | 1.1640 | 0.5670 | -1.0376 | 0.0002 | 0.0007 |
| LOXL2 | 4.4869 | 9.3215 | 1.0549 | 0.0112 | 0.0193 |
| DACT3 | 9.8950 | 1.3235 | -2.9023 | 0.0000 | 0.0000 |
| DYSF | 8.8938 | 3.2608 | -1.4476 | 0.0028 | 0.0056 |
| CSDC2 | 4.4825 | 1.3000 | -1.7857 | 0.0000 | 0.0000 |
| A2M | 321.6205 | 74.2804 | -2.1143 | 0.0000 | 0.0000 |
| ACTG2 | 974.2562 | 61.9605 | -3.9749 | 0.0000 | 0.0000 |
| SELENOM | 40.5575 | 16.6588 | -1.2837 | 0.0000 | 0.0000 |
| DCHS1 | 6.6860 | 1.7844 | -1.9057 | 0.0000 | 0.0000 |
| PLEKHO1 | 20.6429 | 9.3717 | -1.1393 | 0.0000 | 0.0001 |
| RECK | 3.7696 | 1.0547 | -1.8376 | 0.0000 | 0.0000 |
| SNED1 | 1.7678 | 0.4985 | -1.8262 | 0.0000 | 0.0000 |
| LAMA2 | 7.8871 | 1.6350 | -2.2702 | 0.0000 | 0.0000 |
| ADAMTS12 | 0.4768 | 1.7775 | 1.8985 | 0.0037 | 0.0074 |
| C1R | 105.3019 | 52.3921 | -1.0071 | 0.0000 | 0.0001 |
| PTGDS | 77.6312 | 16.4323 | -2.2401 | 0.0000 | 0.0000 |
| LRRN4CL | 6.1143 | 1.0163 | -2.5889 | 0.0000 | 0.0000 |
| PDGFRA | 10.1090 | 2.7486 | -1.8789 | 0.0000 | 0.0000 |
| IGDCC4 | 1.4358 | 0.5442 | -1.3996 | 0.0000 | 0.0000 |
| BNC2 | 2.0948 | 0.4658 | -2.1691 | 0.0000 | 0.0000 |
| ENTPD1 | 6.9985 | 3.2730 | -1.0964 | 0.0001 | 0.0004 |
| TPM1 | 144.5294 | 16.7516 | -3.1090 | 0.0000 | 0.0000 |
| CRYAB | 72.9754 | 7.1650 | -3.3484 | 0.0000 | 0.0000 |
| MAP1A | 10.1440 | 1.4698 | -2.7869 | 0.0000 | 0.0000 |
| ITGA1 | 8.4630 | 2.4311 | -1.7995 | 0.0011 | 0.0026 |
| NEXN | 22.9352 | 2.8687 | -2.9991 | 0.0000 | 0.0000 |
| EBF1 | 4.2858 | 0.8909 | -2.2663 | 0.0000 | 0.0000 |
| PRELP | 42.8381 | 3.9612 | -3.4349 | 0.0000 | 0.0000 |
| PKIG | 56.8237 | 16.0506 | -1.8239 | 0.0000 | 0.0000 |
| PDZRN3 | 12.4546 | 1.8735 | -2.7329 | 0.0000 | 0.0000 |
| SMOC2 | 47.3837 | 7.2474 | -2.7089 | 0.0000 | 0.0000 |
| ELN | 26.4519 | 6.8002 | -1.9597 | 0.0000 | 0.0000 |
| CLIP3 | 28.2417 | 4.7576 | -2.5695 | 0.0000 | 0.0000 |
| JAM2 | 5.9447 | 1.0629 | -2.4835 | 0.0000 | 0.0000 |
| TPSAB1 | 27.4220 | 5.6378 | -2.2821 | 0.0000 | 0.0000 |
| PMP22 | 63.3313 | 20.1608 | -1.6514 | 0.0000 | 0.0000 |
| MGP | 206.3658 | 69.2456 | -1.5754 | 0.0000 | 0.0000 |
| PTH1R | 2.1484 | 0.4529 | -2.2459 | 0.0000 | 0.0000 |
| LMOD1 | 196.0279 | 11.5485 | -4.0853 | 0.0000 | 0.0000 |
| FXYD6 | 23.5517 | 2.7713 | -3.0872 | 0.0000 | 0.0000 |
| ZEB2 | 3.3220 | 1.0306 | -1.6886 | 0.0000 | 0.0000 |
| RGS2 | 132.5773 | 21.8729 | -2.5996 | 0.0000 | 0.0000 |
| DPYSL2 | 16.8033 | 6.7068 | -1.3251 | 0.0000 | 0.0000 |
| RAMP1 | 50.9102 | 12.3098 | -2.0481 | 0.0000 | 0.0000 |
| MYLK | 75.2987 | 5.0543 | -3.8970 | 0.0000 | 0.0000 |
| ROR2 | 6.4192 | 2.3049 | -1.4777 | 0.0000 | 0.0000 |
| TPSB2 | 23.6386 | 5.8173 | -2.0227 | 0.0000 | 0.0000 |
| THBS1 | 197.0031 | 38.1688 | -2.3678 | 0.0000 | 0.0001 |
| PALLD | 100.8551 | 14.6228 | -2.7860 | 0.0000 | 0.0000 |
| GRASP | 10.4856 | 2.9988 | -1.8060 | 0.0000 | 0.0000 |
| RSPO3 | 3.9929 | 0.9683 | -2.0439 | 0.0000 | 0.0000 |
| CXorf36 | 3.7710 | 1.7137 | -1.1379 | 0.0000 | 0.0000 |
| CYGB | 25.8412 | 7.2833 | -1.8270 | 0.0000 | 0.0000 |
| ADRA2A | 3.9337 | 1.2449 | -1.6599 | 0.0000 | 0.0000 |
| SPARCL1 | 255.4386 | 35.4317 | -2.8499 | 0.0000 | 0.0000 |
| GAS6 | 38.1098 | 18.0249 | -1.0802 | 0.0000 | 0.0000 |
| ZEB1 | 12.7741 | 2.1833 | -2.5486 | 0.0000 | 0.0000 |
| S1PR1 | 14.4835 | 4.4876 | -1.6904 | 0.0000 | 0.0000 |
| WNT2 | 0.6604 | 2.6062 | 1.9805 | 0.0053 | 0.0100 |
| CYS1 | 2.7215 | 0.8553 | -1.6699 | 0.0000 | 0.0000 |
| HSPB6 | 243.5957 | 11.5029 | -4.4044 | 0.0000 | 0.0000 |
| SYT11 | 7.5775 | 2.6673 | -1.5063 | 0.0000 | 0.0000 |
| CYBRD1 | 41.1373 | 10.0065 | -2.0395 | 0.0000 | 0.0000 |
| PLA2G5 | 5.0173 | 0.7067 | -2.8278 | 0.0000 | 0.0001 |
| ARID5A | 26.9496 | 7.8167 | -1.7856 | 0.0000 | 0.0000 |
| MXRA7 | 23.3170 | 5.8429 | -1.9966 | 0.0000 | 0.0000 |
| PRKG1 | 4.9162 | 0.8307 | -2.5651 | 0.0000 | 0.0000 |
| ST6GALNAC5 | 2.1538 | 1.0003 | -1.1064 | 0.0009 | 0.0022 |
| FLNC | 150.6621 | 6.0034 | -4.6494 | 0.0000 | 0.0000 |
| TNS2 | 16.9706 | 4.6566 | -1.8657 | 0.0000 | 0.0000 |
| LDB2 | 4.5132 | 1.6490 | -1.4526 | 0.0000 | 0.0000 |
| TNFSF12 | 20.9430 | 9.1494 | -1.1947 | 0.0000 | 0.0000 |
| CILP | 24.2077 | 3.2693 | -2.8884 | 0.0000 | 0.0000 |
| JPH2 | 18.4228 | 2.0221 | -3.1875 | 0.0000 | 0.0000 |
| ATP8B2 | 6.5150 | 2.7853 | -1.2259 | 0.0000 | 0.0000 |
| PLN | 55.9697 | 4.3513 | -3.6851 | 0.0000 | 0.0000 |
| SDC2 | 29.7522 | 10.1954 | -1.5451 | 0.0000 | 0.0000 |
| CCL2 | 92.5650 | 12.4648 | -2.8926 | 0.0000 | 0.0000 |
| JCAD | 11.4265 | 3.3371 | -1.7757 | 0.0004 | 0.0011 |
| SLC2A3 | 21.9974 | 8.2693 | -1.4115 | 0.0000 | 0.0000 |
| HSPB7 | 29.8995 | 2.7514 | -3.4419 | 0.0000 | 0.0000 |
| DPYSL3 | 72.0979 | 17.7174 | -2.0248 | 0.0000 | 0.0001 |
| INMT | 5.0642 | 1.2605 | -2.0063 | 0.0000 | 0.0000 |
| FHL1 | 117.2400 | 7.2412 | -4.0171 | 0.0000 | 0.0000 |
| SLIT2 | 4.2710 | 0.7326 | -2.5434 | 0.0000 | 0.0000 |
| GPR183 | 14.8467 | 5.5360 | -1.4232 | 0.0000 | 0.0000 |
| GRK5 | 4.4441 | 2.1520 | -1.0462 | 0.0000 | 0.0000 |
| MEF2C | 4.3109 | 1.3759 | -1.6476 | 0.0000 | 0.0000 |
| TSPAN18 | 11.7507 | 2.3453 | -2.3249 | 0.0000 | 0.0000 |
| CPA3 | 19.0614 | 4.3839 | -2.1204 | 0.0000 | 0.0000 |
| FLNA | 792.9072 | 102.7258 | -2.9484 | 0.0000 | 0.0000 |
| TNS1 | 114.6837 | 7.4446 | -3.9453 | 0.0000 | 0.0000 |
| SRPX | 39.3936 | 8.0897 | -2.2838 | 0.0000 | 0.0000 |
| STARD8 | 2.4055 | 1.0327 | -1.2199 | 0.0000 | 0.0000 |
| KLF9 | 29.1047 | 5.4698 | -2.4117 | 0.0000 | 0.0000 |
| KCNMB1 | 18.0526 | 1.4975 | -3.5916 | 0.0000 | 0.0000 |
| ARHGEF17 | 7.8386 | 3.7906 | -1.0482 | 0.0002 | 0.0007 |
| ADAMTS16 | 1.5787 | 0.6221 | -1.3434 | 0.0287 | 0.0440 |
| CTSG | 12.4415 | 1.8783 | -2.7276 | 0.0000 | 0.0000 |
| PODNL1 | 0.6791 | 2.1242 | 1.6452 | 0.0071 | 0.0130 |
| FOXF1 | 26.8626 | 3.7763 | -2.8306 | 0.0000 | 0.0000 |
| RUSC2 | 11.5009 | 3.9758 | -1.5324 | 0.0000 | 0.0000 |
| KLF2 | 50.1027 | 7.8978 | -2.6654 | 0.0000 | 0.0000 |
| DNAJB5 | 11.9928 | 2.0558 | -2.5444 | 0.0000 | 0.0000 |
| ADAM33 | 7.9763 | 1.0312 | -2.9513 | 0.0000 | 0.0000 |
| BOC | 5.0671 | 1.1335 | -2.1604 | 0.0000 | 0.0000 |
| PTGIS | 54.2121 | 6.3505 | -3.0937 | 0.0000 | 0.0000 |
| PDE4B | 4.2675 | 1.1938 | -1.8378 | 0.0000 | 0.0000 |
| PCDH18 | 4.4399 | 1.3055 | -1.7660 | 0.0000 | 0.0000 |
| HAND2 | 7.8585 | 0.9219 | -3.0916 | 0.0000 | 0.0000 |
| RCAN2 | 24.4643 | 3.1431 | -2.9604 | 0.0000 | 0.0000 |
| PDE1A | 3.2791 | 0.5254 | -2.6419 | 0.0000 | 0.0000 |
| SYNC | 2.5249 | 0.5594 | -2.1742 | 0.0000 | 0.0001 |
| AOC3 | 68.9734 | 7.8652 | -3.1325 | 0.0000 | 0.0000 |
| LIX1L | 12.9297 | 6.2647 | -1.0454 | 0.0000 | 0.0000 |
| GALNT15 | 3.9485 | 0.7246 | -2.4460 | 0.0000 | 0.0000 |
| SYNPO | 19.8413 | 6.7404 | -1.5576 | 0.0000 | 0.0000 |
| C1QTNF2 | 2.2831 | 0.5859 | -1.9622 | 0.0000 | 0.0000 |
| SVEP1 | 5.2590 | 0.7044 | -2.9004 | 0.0000 | 0.0000 |
| GSN | 168.7679 | 36.7816 | -2.1980 | 0.0000 | 0.0000 |
| CLDN5 | 20.1227 | 3.8321 | -2.3926 | 0.0000 | 0.0000 |
| AQP1 | 110.1434 | 23.8330 | -2.2084 | 0.0000 | 0.0000 |
| FYN | 9.5360 | 4.1923 | -1.1856 | 0.0000 | 0.0001 |
| TUBA1A | 91.4231 | 40.1857 | -1.1859 | 0.0000 | 0.0000 |
| MRAS | 7.1115 | 2.9868 | -1.2515 | 0.0035 | 0.0069 |
| STARD13 | 5.0764 | 1.5371 | -1.7236 | 0.0000 | 0.0000 |
| IGFBP5 | 130.1210 | 45.6972 | -1.5097 | 0.0000 | 0.0000 |
| TEK | 2.7376 | 1.2106 | -1.1772 | 0.0000 | 0.0000 |
| TSHZ3 | 6.2660 | 1.7924 | -1.8056 | 0.0000 | 0.0000 |
| PPP1R14A | 44.8853 | 6.2627 | -2.8414 | 0.0000 | 0.0000 |
| TMEM273 | 2.8015 | 1.2553 | -1.1581 | 0.0000 | 0.0000 |
| ARHGAP24 | 2.5567 | 0.8047 | -1.6678 | 0.0000 | 0.0000 |
| TCF4 | 3.3797 | 1.6738 | -1.0138 | 0.0000 | 0.0000 |
| GATA6 | 9.2907 | 1.5442 | -2.5889 | 0.0000 | 0.0000 |
| RBPMS2 | 38.8435 | 3.8981 | -3.3168 | 0.0000 | 0.0000 |
| CLEC3B | 53.1329 | 2.6517 | -4.3246 | 0.0000 | 0.0000 |
| CH25H | 15.9923 | 2.0900 | -2.9358 | 0.0000 | 0.0000 |
| KANK2 | 55.3858 | 9.6790 | -2.5166 | 0.0000 | 0.0000 |
| CCL21 | 31.2784 | 14.6117 | -1.0980 | 0.0002 | 0.0007 |
| DES | 2904.3624 | 172.0796 | -4.0771 | 0.0000 | 0.0000 |
| LATS2 | 9.4811 | 3.6335 | -1.3837 | 0.0000 | 0.0000 |
| CLIC4 | 109.6921 | 29.9993 | -1.8705 | 0.0007 | 0.0016 |
| CXCR4 | 47.4861 | 19.6747 | -1.2712 | 0.0021 | 0.0045 |
| WISP1 | 0.6491 | 3.2128 | 2.3072 | 0.0231 | 0.0363 |
| HAS1 | 7.4804 | 1.0839 | -2.7869 | 0.0000 | 0.0000 |
| KANK3 | 2.4002 | 1.1518 | -1.0593 | 0.0000 | 0.0000 |
| GPBAR1 | 2.1667 | 0.5198 | -2.0595 | 0.0000 | 0.0000 |
| JAZF1 | 8.4468 | 2.4625 | -1.7783 | 0.0000 | 0.0000 |
| SGCA | 15.0057 | 1.4729 | -3.3488 | 0.0000 | 0.0000 |
| NPR1 | 5.3286 | 1.6402 | -1.6999 | 0.0000 | 0.0000 |
| F10 | 7.1845 | 0.6063 | -3.5669 | 0.0000 | 0.0000 |
| PLPP4 | 0.1289 | 2.5141 | 4.2852 | 0.0000 | 0.0001 |
| ADCY4 | 1.8856 | 0.9398 | -1.0046 | 0.0000 | 0.0000 |
| MYCT1 | 4.6248 | 1.6971 | -1.4463 | 0.0000 | 0.0000 |
| ECM1 | 32.4574 | 13.4091 | -1.2753 | 0.0090 | 0.0160 |
| NEGR1 | 5.5978 | 0.3951 | -3.8245 | 0.0000 | 0.0000 |
| BIN1 | 16.7546 | 4.4480 | -1.9133 | 0.0000 | 0.0000 |
| CD200 | 10.2314 | 3.5576 | -1.5240 | 0.0000 | 0.0000 |
| HABP4 | 6.2656 | 2.6685 | -1.2314 | 0.0000 | 0.0000 |
| ACTN1 | 64.9789 | 28.9581 | -1.1660 | 0.0165 | 0.0269 |
| LYVE1 | 10.3098 | 1.9692 | -2.3883 | 0.0000 | 0.0000 |
| ABI3BP | 9.2158 | 1.0625 | -3.1167 | 0.0000 | 0.0000 |
| EFEMP1 | 37.2686 | 15.7699 | -1.2408 | 0.0000 | 0.0000 |
| ARHGEF25 | 19.2999 | 4.3482 | -2.1501 | 0.0000 | 0.0000 |
| PTGFR | 4.5520 | 0.4905 | -3.2141 | 0.0000 | 0.0000 |
| PKDCC | 13.4060 | 2.5911 | -2.3713 | 0.0000 | 0.0000 |
| IL6 | 48.1763 | 5.1994 | -3.2119 | 0.0000 | 0.0000 |
| ITGA7 | 20.2385 | 3.4503 | -2.5523 | 0.0000 | 0.0000 |
| EGR2 | 12.4521 | 2.7461 | -2.1809 | 0.0000 | 0.0000 |
| GADD45B | 119.8627 | 23.8533 | -2.3291 | 0.0000 | 0.0000 |
| PRICKLE2 | 5.9806 | 1.4433 | -2.0509 | 0.0000 | 0.0000 |
| RASGRP2 | 4.3983 | 0.9698 | -2.1811 | 0.0000 | 0.0000 |
| CFL2 | 20.5453 | 2.7302 | -2.9117 | 0.0000 | 0.0000 |
| CSRP1 | 217.6064 | 26.2443 | -3.0516 | 0.0000 | 0.0000 |
| OMD | 3.7842 | 0.5741 | -2.7206 | 0.0000 | 0.0000 |
| ARHGEF15 | 3.5160 | 1.7004 | -1.0481 | 0.0000 | 0.0000 |
| ALDH1B1 | 81.9402 | 16.8738 | -2.2798 | 0.0001 | 0.0003 |
| OGN | 23.3234 | 1.1981 | -4.2829 | 0.0000 | 0.0000 |
| HSD17B6 | 12.3573 | 2.3431 | -2.3989 | 0.0000 | 0.0000 |
| FCN1 | 1.9577 | 0.6971 | -1.4897 | 0.0000 | 0.0002 |
| SFRP1 | 20.7511 | 3.2464 | -2.6763 | 0.0000 | 0.0000 |
| INHBA | 0.8995 | 5.7427 | 2.6745 | 0.0003 | 0.0008 |
| CHI3L1 | 7.7202 | 29.4251 | 1.9303 | 0.0022 | 0.0047 |
| FGL2 | 45.4346 | 4.8770 | -3.2197 | 0.0000 | 0.0000 |
| SHISAL1 | 8.3489 | 0.5268 | -3.9862 | 0.0000 | 0.0000 |
| FOXS1 | 0.8493 | 1.9779 | 1.2196 | 0.0008 | 0.0018 |
| IL16 | 2.0556 | 0.8678 | -1.2442 | 0.0000 | 0.0000 |
| TNFAIP8L3 | 8.2167 | 1.7888 | -2.1995 | 0.0000 | 0.0000 |
| STX2 | 5.0547 | 2.4305 | -1.0564 | 0.0002 | 0.0006 |
| ABCC9 | 2.0300 | 0.4423 | -2.1984 | 0.0000 | 0.0000 |
| BAG2 | 11.4663 | 2.2906 | -2.3236 | 0.0000 | 0.0000 |
| COL10A1 | 0.0536 | 6.9252 | 7.0147 | 0.0000 | 0.0000 |
| PLAC9 | 23.5158 | 3.1922 | -2.8810 | 0.0000 | 0.0000 |
| XPNPEP2 | 4.4633 | 0.3911 | -3.5126 | 0.0000 | 0.0000 |
| PTGS1 | 108.3385 | 6.7910 | -3.9958 | 0.0000 | 0.0000 |
| RGCC | 38.2915 | 15.9145 | -1.2667 | 0.0000 | 0.0001 |
| TSPAN11 | 2.9874 | 0.9773 | -1.6121 | 0.0000 | 0.0000 |
| BHMT2 | 5.1575 | 0.3845 | -3.7457 | 0.0000 | 0.0000 |
| SMTN | 72.4963 | 9.2837 | -2.9651 | 0.0000 | 0.0000 |
| NXPH3 | 4.1480 | 0.7533 | -2.4611 | 0.0000 | 0.0000 |
| HSPA12A | 2.1109 | 0.6254 | -1.7551 | 0.0000 | 0.0000 |
| PDE2A | 5.7639 | 1.0736 | -2.4246 | 0.0000 | 0.0000 |
| OR7E47P | 2.0470 | 0.5965 | -1.7788 | 0.0000 | 0.0000 |
| GPIHBP1 | 4.4876 | 0.6779 | -2.7269 | 0.0000 | 0.0000 |
| RCSD1 | 4.8215 | 2.0486 | -1.2348 | 0.0000 | 0.0000 |
| DOCK11 | 3.9405 | 1.7464 | -1.1740 | 0.0001 | 0.0002 |
| ZFPM2 | 2.6797 | 0.5007 | -2.4201 | 0.0000 | 0.0000 |
| P2RX1 | 26.4405 | 1.5140 | -4.1263 | 0.0000 | 0.0000 |
| ADAMTS9 | 3.5346 | 1.1868 | -1.5745 | 0.0000 | 0.0001 |
| NAP1L3 | 2.2109 | 0.6656 | -1.7319 | 0.0000 | 0.0000 |
| MAP1B | 23.3192 | 3.1265 | -2.8989 | 0.0000 | 0.0000 |
| DIXDC1 | 9.5731 | 1.3281 | -2.8497 | 0.0000 | 0.0000 |
| C4B | 2.3484 | 0.9927 | -1.2423 | 0.0001 | 0.0002 |
| MYH11 | 976.3795 | 30.7093 | -4.9907 | 0.0000 | 0.0000 |
| CELF2 | 6.7850 | 1.3353 | -2.3451 | 0.0000 | 0.0000 |
| PID1 | 8.1046 | 1.0973 | -2.8848 | 0.0000 | 0.0000 |
| SOX17 | 7.1823 | 1.1079 | -2.6966 | 0.0000 | 0.0000 |
| CNTNAP1 | 3.3795 | 1.6101 | -1.0696 | 0.0000 | 0.0001 |
| ANTXR2 | 21.6256 | 4.7250 | -2.1944 | 0.0000 | 0.0000 |
| RGS1 | 24.7732 | 12.3512 | -1.0041 | 0.0000 | 0.0002 |
| ITM2A | 24.0212 | 6.4503 | -1.8969 | 0.0000 | 0.0000 |
| NES | 18.6010 | 8.9057 | -1.0626 | 0.0000 | 0.0000 |
| CMA1 | 6.7037 | 0.8556 | -2.9699 | 0.0000 | 0.0000 |
| NCKAP1L | 5.3899 | 2.1815 | -1.3050 | 0.0000 | 0.0001 |
| P3H1 | 3.7177 | 8.0994 | 1.1234 | 0.0000 | 0.0000 |
| CCL23 | 1.6623 | 0.6514 | -1.3516 | 0.0000 | 0.0000 |
| PDE3A | 1.2177 | 0.5859 | -1.0555 | 0.0000 | 0.0000 |
| KCTD12 | 22.8151 | 10.0221 | -1.1868 | 0.0000 | 0.0000 |
| SPHK1 | 3.0106 | 8.6235 | 1.5183 | 0.0167 | 0.0273 |
| COL11A1 | 0.0845 | 4.8236 | 5.8343 | 0.0000 | 0.0000 |
| DAAM2 | 3.9533 | 1.0166 | -1.9593 | 0.0000 | 0.0000 |
| EPB41L2 | 7.0224 | 3.1624 | -1.1509 | 0.0000 | 0.0000 |
| POPDC2 | 26.6200 | 2.0715 | -3.6838 | 0.0000 | 0.0000 |
| PARVA | 20.3336 | 7.6312 | -1.4139 | 0.0000 | 0.0001 |
| AOX1 | 6.0475 | 0.6081 | -3.3139 | 0.0000 | 0.0000 |
| PSD | 19.2065 | 1.6731 | -3.5210 | 0.0000 | 0.0000 |
| EMCN | 5.3618 | 1.6413 | -1.7079 | 0.0000 | 0.0000 |
| WISP2 | 7.1703 | 1.5171 | -2.2407 | 0.0000 | 0.0000 |
| SLC24A3 | 10.0749 | 2.3590 | -2.0945 | 0.0000 | 0.0000 |
| SOBP | 4.5570 | 0.6107 | -2.8996 | 0.0000 | 0.0000 |
| CYTL1 | 6.4332 | 1.9460 | -1.7250 | 0.0000 | 0.0000 |
| ACTC1 | 220.2905 | 7.1666 | -4.9420 | 0.0000 | 0.0000 |
| GALNT17 | 5.4716 | 0.5992 | -3.1909 | 0.0000 | 0.0000 |
| TCF21 | 11.7172 | 0.7302 | -4.0043 | 0.0000 | 0.0000 |
