# Supplementary material for: Prognostic Value of Vascular-Expressed PSMA and CD248 in Urothelial Carcinoma of the Bladder
Source: Front Oncol. 2021 Nov 17;11:771036. doi: 10.3389/fonc.2021.771036 (PMC8635966; doi:10.3389/fonc.2021.771036)
Supplement: Supplementary file 2 [file DataSheet_1.zip › Supporting Data 3.DOCX]

**Supporting data 3. DETFs list of TCGA-BLCA dataset**

| **Gene** | **ConMean** | **TreatMean** | **logFC** | ***P*** | **FDR** |
| --- | --- | --- | --- | --- | --- |
| AR | 3.0652 | 1.4591 | -1.0709 | 0.0000 | 0.0000 |
| ARID3A | 0.9785 | 4.2947 | 2.1339 | 0.0005 | 0.0012 |
| ATF3 | 160.0250 | 21.1543 | -2.9193 | 0.0000 | 0.0000 |
| BRCA1 | 0.9776 | 3.0501 | 1.6415 | 0.0000 | 0.0000 |
| CBX2 | 1.0055 | 5.0579 | 2.3306 | 0.0000 | 0.0000 |
| CBX3 | 23.2061 | 49.7704 | 1.1008 | 0.0000 | 0.0000 |
| CBX7 | 15.1581 | 3.2511 | -2.2211 | 0.0000 | 0.0000 |
| CBX8 | 1.0299 | 3.3670 | 1.7089 | 0.0000 | 0.0000 |
| CDK2 | 7.4066 | 17.0603 | 1.2038 | 0.0000 | 0.0000 |
| CDX2 | 0.0704 | 0.7411 | 3.3965 | 0.0178 | 0.0288 |
| CEBPA | 8.2394 | 19.1930 | 1.2200 | 0.0055 | 0.0103 |
| CENPA | 0.9031 | 6.3095 | 2.8045 | 0.0000 | 0.0000 |
| DNMT1 | 4.5150 | 10.8820 | 1.2691 | 0.0000 | 0.0000 |
| E2F1 | 3.8291 | 15.3941 | 2.0073 | 0.0000 | 0.0000 |
| E2F3 | 3.7303 | 10.3299 | 1.4695 | 0.0000 | 0.0000 |
| E2F7 | 0.3556 | 1.8553 | 2.3832 | 0.0000 | 0.0000 |
| EBF1 | 4.2858 | 0.8909 | -2.2663 | 0.0000 | 0.0000 |
| EGR1 | 571.8888 | 64.9063 | -3.1393 | 0.0000 | 0.0000 |
| EGR2 | 12.4521 | 2.7461 | -2.1809 | 0.0000 | 0.0000 |
| EMX1 | 0.0426 | 0.2668 | 2.6462 | 0.0001 | 0.0004 |
| ESR1 | 2.4269 | 0.4085 | -2.5708 | 0.0000 | 0.0000 |
| EZH2 | 1.9273 | 7.5238 | 1.9649 | 0.0000 | 0.0000 |
| FOS | 917.6179 | 120.5713 | -2.9280 | 0.0000 | 0.0000 |
| FOSL1 | 80.0768 | 22.2569 | -1.8471 | 0.0024 | 0.0051 |
| FOXM1 | 2.6602 | 13.4652 | 2.3396 | 0.0000 | 0.0000 |
| FOXP2 | 1.7755 | 0.2744 | -2.6941 | 0.0000 | 0.0000 |
| GATA3 | 42.1348 | 86.2928 | 1.0342 | 0.0051 | 0.0097 |
| GATA6 | 9.2907 | 1.5442 | -2.5889 | 0.0000 | 0.0000 |
| GRHL2 | 7.2730 | 15.0435 | 1.0485 | 0.0001 | 0.0003 |
| H2AFX | 18.5647 | 58.7116 | 1.6611 | 0.0000 | 0.0000 |
| HOXB7 | 8.3365 | 20.0301 | 1.2647 | 0.0000 | 0.0000 |
| HOXC9 | 0.1697 | 2.1563 | 3.6679 | 0.0001 | 0.0002 |
| IRF4 | 1.5072 | 0.6514 | -1.2104 | 0.0016 | 0.0036 |
| IRF5 | 3.2458 | 9.9357 | 1.6140 | 0.0000 | 0.0000 |
| JUN | 257.5879 | 68.2420 | -1.9163 | 0.0000 | 0.0000 |
| JUNB | 607.7429 | 205.8415 | -1.5619 | 0.0000 | 0.0000 |
| JUND | 232.2511 | 94.7068 | -1.2941 | 0.0000 | 0.0000 |
| KDM6B | 19.8578 | 9.0722 | -1.1302 | 0.0002 | 0.0007 |
| KLF4 | 59.1993 | 12.2355 | -2.2745 | 0.0000 | 0.0000 |
| LHX2 | 0.0587 | 0.3887 | 2.7264 | 0.0001 | 0.0002 |
| LIN9 | 0.8859 | 2.1270 | 1.2637 | 0.0000 | 0.0000 |
| LMNB1 | 6.0267 | 21.1118 | 1.8086 | 0.0000 | 0.0000 |
| MAFF | 39.8896 | 11.2410 | -1.8272 | 0.0000 | 0.0000 |
| MEF2C | 4.3109 | 1.3759 | -1.6476 | 0.0000 | 0.0000 |
| MEIS1 | 5.1611 | 1.7770 | -1.5383 | 0.0000 | 0.0000 |
| MITF | 3.3856 | 1.0033 | -1.7546 | 0.0000 | 0.0000 |
| MYB | 0.3828 | 1.6231 | 2.0839 | 0.0001 | 0.0004 |
| MYBL2 | 6.8975 | 40.2458 | 2.5447 | 0.0000 | 0.0000 |
| MYC | 95.1203 | 35.7543 | -1.4116 | 0.0000 | 0.0001 |
| MYH11 | 976.3795 | 30.7093 | -4.9907 | 0.0000 | 0.0000 |
| NCAPG | 1.1116 | 5.3634 | 2.2705 | 0.0000 | 0.0000 |
| NFATC1 | 3.5367 | 1.3365 | -1.4040 | 0.0000 | 0.0000 |
| NFIC | 19.7054 | 8.9237 | -1.1429 | 0.0000 | 0.0000 |
| NR2F1 | 11.6356 | 3.7170 | -1.6463 | 0.0000 | 0.0000 |
| NR4A1 | 151.4262 | 12.1114 | -3.6442 | 0.0000 | 0.0000 |
| PBX1 | 10.7898 | 4.7747 | -1.1762 | 0.0000 | 0.0000 |
| PDX1 | 0.0388 | 0.5069 | 3.7059 | 0.0003 | 0.0009 |
| POLR3G | 0.4762 | 1.0673 | 1.1643 | 0.0102 | 0.0177 |
| POU5F1 | 1.0754 | 4.6919 | 2.1253 | 0.0011 | 0.0025 |
| PRDM1 | 10.5738 | 3.9981 | -1.4031 | 0.0227 | 0.0358 |
| RFX2 | 3.6811 | 1.5713 | -1.2282 | 0.0000 | 0.0000 |
| RUNX1T1 | 1.0079 | 0.2000 | -2.3335 | 0.0000 | 0.0000 |
| SALL4 | 0.1522 | 1.4427 | 3.2451 | 0.0000 | 0.0000 |
| SOX17 | 7.1823 | 1.1079 | -2.6966 | 0.0000 | 0.0000 |
| SOX4 | 12.5263 | 43.1268 | 1.7836 | 0.0000 | 0.0000 |
| SOX9 | 3.6892 | 11.6925 | 1.6642 | 0.0046 | 0.0088 |
| SREBF1 | 10.5688 | 26.2263 | 1.3112 | 0.0000 | 0.0000 |
| SRF | 45.7378 | 16.5927 | -1.4628 | 0.0000 | 0.0000 |
| STAT1 | 26.6203 | 53.4255 | 1.0050 | 0.0112 | 0.0192 |
| TCF21 | 11.7172 | 0.7302 | -4.0043 | 0.0000 | 0.0000 |
| TEAD1 | 18.0913 | 5.7635 | -1.6503 | 0.0000 | 0.0001 |
| TEAD4 | 3.2021 | 7.9313 | 1.3085 | 0.0006 | 0.0014 |
| TFAP2A | 2.2648 | 6.1705 | 1.4460 | 0.0002 | 0.0007 |
| TP63 | 10.5906 | 21.2218 | 1.0028 | 0.0111 | 0.0190 |
| TP73 | 0.4265 | 1.6731 | 1.9719 | 0.0002 | 0.0007 |
| TTF2 | 1.3959 | 3.1158 | 1.1584 | 0.0000 | 0.0000 |
| WWTR1 | 22.0261 | 6.3980 | -1.7835 | 0.0000 | 0.0000 |
