# Supplementary material for: Prognostic Value of Vascular-Expressed PSMA and CD248 in Urothelial Carcinoma of the Bladder
Source: Front Oncol. 2021 Nov 17;11:771036. doi: 10.3389/fonc.2021.771036 (PMC8635966; doi:10.3389/fonc.2021.771036)
Supplement: Supplementary file 2 [file DataSheet_1.zip › Supporting Data 4.DOCX]

**Supporting data 4. PCor-DEGs list of TCGA-BLCA dataset**

| **Gene** | **HR** | **HR.95L** | **HR.95H** | ***P*** |
| --- | --- | --- | --- | --- |
| ABCC9 | 1.5410 | 1.2335 | 1.9251 | 0.0001 |
| ADAM12 | 1.0329 | 1.0047 | 1.0620 | 0.0221 |
| ADAMTS12 | 1.0494 | 1.0128 | 1.0874 | 0.0077 |
| ADAMTS16 | 1.1648 | 1.0873 | 1.2477 | 0.0000 |
| ADAMTS9 | 1.1460 | 1.0552 | 1.2446 | 0.0012 |
| ANXA6 | 1.0084 | 1.0010 | 1.0159 | 0.0262 |
| ARHGEF17 | 1.0693 | 1.0070 | 1.1354 | 0.0286 |
| ATP8B2 | 1.0329 | 1.0134 | 1.0529 | 0.0009 |
| BGN | 1.0008 | 1.0003 | 1.0014 | 0.0049 |
| CAVIN1 | 1.0025 | 1.0002 | 1.0048 | 0.0314 |
| CCDC80 | 1.0138 | 1.0048 | 1.0230 | 0.0026 |
| CD248 | 1.0036 | 1.0007 | 1.0066 | 0.0162 |
| CHI3L1 | 1.0006 | 1.0001 | 1.0012 | 0.0220 |
| COL11A1 | 1.0111 | 1.0018 | 1.0205 | 0.0194 |
| COL14A1 | 1.0086 | 1.0009 | 1.0165 | 0.0289 |
| COL6A2 | 1.0011 | 1.0003 | 1.0018 | 0.0051 |
| CRISPLD2 | 1.0197 | 1.0038 | 1.0358 | 0.0151 |
| CTHRC1 | 1.0035 | 1.0000 | 1.0070 | 0.0468 |
| CXCL12 | 1.0140 | 1.0057 | 1.0224 | 0.0009 |
| CYTL1 | 1.0147 | 1.0039 | 1.0257 | 0.0077 |
| DCN | 1.0052 | 1.0009 | 1.0095 | 0.0174 |
| DPT | 1.0077 | 1.0015 | 1.0139 | 0.0149 |
| DPYSL2 | 1.0314 | 1.0136 | 1.0494 | 0.0005 |
| DPYSL3 | 1.0058 | 1.0005 | 1.0112 | 0.0323 |
| DYSF | 1.0669 | 1.0143 | 1.1222 | 0.0120 |
| ECM1 | 1.0081 | 1.0033 | 1.0130 | 0.0009 |
| EDNRA | 1.0859 | 1.0348 | 1.1395 | 0.0008 |
| EFEMP1 | 1.0040 | 1.0010 | 1.0071 | 0.0096 |
| ELN | 1.0173 | 1.0056 | 1.0292 | 0.0036 |
| F10 | 1.1663 | 1.0603 | 1.2831 | 0.0016 |
| FBN1 | 1.0439 | 1.0193 | 1.0692 | 0.0004 |
| GALNT15 | 1.0910 | 1.0076 | 1.1814 | 0.0319 |
| GAS7 | 1.1159 | 1.0525 | 1.1830 | 0.0002 |
| GPIHBP1 | 1.1457 | 1.0339 | 1.2696 | 0.0094 |
| GRK5 | 1.1178 | 1.0444 | 1.1963 | 0.0013 |
| GSN | 1.0086 | 1.0034 | 1.0139 | 0.0013 |
| HSPB2 | 1.1807 | 1.0383 | 1.3426 | 0.0113 |
| IGDCC4 | 1.2168 | 1.0086 | 1.4679 | 0.0404 |
| ITGA1 | 1.0597 | 1.0022 | 1.1204 | 0.0414 |
| JAM3 | 1.0169 | 1.0016 | 1.0323 | 0.0302 |
| KCNE4 | 1.0962 | 1.0402 | 1.1551 | 0.0006 |
| LAMA2 | 1.1135 | 1.0405 | 1.1917 | 0.0019 |
| LAMA4 | 1.0360 | 1.0088 | 1.0640 | 0.0092 |
| LATS2 | 1.1016 | 1.0259 | 1.1830 | 0.0077 |
| LDB2 | 1.0965 | 1.0004 | 1.2019 | 0.0491 |
| LHFPL6 | 1.0267 | 1.0117 | 1.0419 | 0.0004 |
| LRRC32 | 1.0263 | 1.0085 | 1.0445 | 0.0037 |
| LRRN4CL | 1.0983 | 1.0004 | 1.2058 | 0.0490 |
| LYVE1 | 1.0245 | 1.0120 | 1.0372 | 0.0001 |
| MAP1A | 1.0675 | 1.0319 | 1.1043 | 0.0002 |
| MAP1B | 1.0485 | 1.0254 | 1.0722 | 0.0000 |
| MCAM | 1.0168 | 1.0043 | 1.0294 | 0.0083 |
| MEDAG | 1.0177 | 1.0088 | 1.0267 | 0.0001 |
| MFAP5 | 1.0142 | 1.0043 | 1.0242 | 0.0048 |
| MXRA7 | 1.0595 | 1.0300 | 1.0899 | 0.0001 |
| MYADM | 1.0086 | 1.0034 | 1.0139 | 0.0013 |
| NES | 1.0328 | 1.0201 | 1.0456 | 0.0000 |
| NRP2 | 1.0449 | 1.0117 | 1.0792 | 0.0076 |
| NXPH3 | 1.1152 | 1.0377 | 1.1985 | 0.0030 |
| OGN | 1.0404 | 1.0049 | 1.0771 | 0.0254 |
| OLFML1 | 1.1088 | 1.0143 | 1.2122 | 0.0231 |
| OLFML3 | 1.0067 | 1.0011 | 1.0123 | 0.0183 |
| P3H1 | 1.0194 | 1.0027 | 1.0363 | 0.0229 |
| PCDH18 | 1.0834 | 1.0270 | 1.1430 | 0.0033 |
| PDGFRA | 1.0464 | 1.0190 | 1.0744 | 0.0008 |
| PDZRN3 | 1.0475 | 1.0015 | 1.0956 | 0.0427 |
| PID1 | 1.1126 | 1.0342 | 1.1970 | 0.0042 |
| PODN | 1.0171 | 1.0008 | 1.0336 | 0.0392 |
| PRKG1 | 1.1640 | 1.0334 | 1.3110 | 0.0124 |
| RGCC | 1.0131 | 1.0059 | 1.0204 | 0.0004 |
| RGS1 | 0.9867 | 0.9740 | 0.9995 | 0.0418 |
| SCN1B | 1.1306 | 1.0062 | 1.2704 | 0.0390 |
| SERPINF1 | 1.0059 | 1.0029 | 1.0089 | 0.0001 |
| SH3RF3 | 1.1112 | 1.0144 | 1.2172 | 0.0234 |
| SLC24A3 | 1.0531 | 1.0064 | 1.1019 | 0.0253 |
| SLC2A3 | 1.0215 | 1.0116 | 1.0315 | 0.0000 |
| SLIT2 | 1.1530 | 1.0365 | 1.2825 | 0.0088 |
| SMOC2 | 1.0105 | 1.0002 | 1.0208 | 0.0452 |
| SNED1 | 1.4447 | 1.1702 | 1.7835 | 0.0006 |
| SPHK1 | 1.0136 | 1.0002 | 1.0272 | 0.0463 |
| SPON1 | 1.0130 | 1.0017 | 1.0245 | 0.0239 |
| SRPX | 1.0158 | 1.0086 | 1.0231 | 0.0000 |
| STARD8 | 1.1903 | 1.0600 | 1.3365 | 0.0032 |
| SVEP1 | 1.3261 | 1.1207 | 1.5692 | 0.0010 |
| SYNC | 1.2195 | 1.0066 | 1.4774 | 0.0426 |
| TCF4 | 1.1471 | 1.0692 | 1.2307 | 0.0001 |
| TGFB1I1 | 1.0218 | 1.0021 | 1.0418 | 0.0296 |
| TGFB3 | 1.0301 | 1.0045 | 1.0563 | 0.0208 |
| THBS1 | 1.0043 | 1.0011 | 1.0075 | 0.0076 |
| TIMP2 | 1.0031 | 1.0004 | 1.0058 | 0.0260 |
| TNFAIP6 | 1.0388 | 1.0073 | 1.0713 | 0.0153 |
| TNFAIP8L3 | 1.0804 | 1.0302 | 1.1331 | 0.0014 |
| TUBA1A | 1.0027 | 1.0012 | 1.0042 | 0.0006 |
| TWIST2 | 1.0352 | 1.0089 | 1.0621 | 0.0084 |
| WISP1 | 1.0235 | 1.0074 | 1.0399 | 0.0042 |
| WISP2 | 1.0368 | 1.0007 | 1.0742 | 0.0455 |
