# Supplementary material for: Prognostic Value of Vascular-Expressed PSMA and CD248 in Urothelial Carcinoma of the Bladder
Source: Front Oncol. 2021 Nov 17;11:771036. doi: 10.3389/fonc.2021.771036 (PMC8635966; doi:10.3389/fonc.2021.771036)
Supplement: Supplementary file 2 [file DataSheet_1.zip › Supporting Data 5.DOCX]

**Supporting data 5. TFs-based regulatory network for PCor-DEGs**

| **DETFs** | **PCor-DEGs** | **Correlation coefficient** | ***P*** | **Regulation** |
| --- | --- | --- | --- | --- |
| AR | CAVIN1 | -0.3061 | 0.0000 | negative |
| CBX2 | TUBA1A | 0.3373 | 0.0000 | postive |
| CBX7 | ANXA6 | 0.3321 | 0.0000 | postive |
| CBX7 | ARHGEF17 | 0.3030 | 0.0000 | postive |
| CBX7 | DPYSL3 | 0.3163 | 0.0000 | postive |
| CBX7 | ELN | 0.3618 | 0.0000 | postive |
| CBX7 | GPIHBP1 | 0.3108 | 0.0000 | postive |
| CBX7 | LRRN4CL | 0.4164 | 0.0000 | postive |
| CBX7 | OGN | 0.3266 | 0.0000 | postive |
| CBX7 | PDZRN3 | 0.3878 | 0.0000 | postive |
| CBX7 | PODN | 0.3543 | 0.0000 | postive |
| CBX7 | SMOC2 | 0.5502 | 0.0000 | postive |
| CBX7 | SPON1 | 0.3746 | 0.0000 | postive |
| CBX7 | TGFB1I1 | 0.3595 | 0.0000 | postive |
| CEBPA | CAVIN1 | -0.3912 | 0.0000 | negative |
| CEBPA | NRP2 | -0.3386 | 0.0000 | negative |
| CEBPA | SPHK1 | -0.3857 | 0.0000 | negative |
| DNMT1 | DPYSL2 | 0.3030 | 0.0000 | postive |
| E2F1 | TUBA1A | 0.3228 | 0.0000 | postive |
| E2F3 | TUBA1A | 0.3103 | 0.0000 | postive |
| E2F7 | TUBA1A | 0.3050 | 0.0000 | postive |
| EBF1 | ABCC9 | 0.4384 | 0.0000 | postive |
| EBF1 | ADAMTS9 | 0.4413 | 0.0000 | postive |
| EBF1 | ANXA6 | 0.5584 | 0.0000 | postive |
| EBF1 | ARHGEF17 | 0.3326 | 0.0000 | postive |
| EBF1 | ATP8B2 | 0.7755 | 0.0000 | postive |
| EBF1 | CCDC80 | 0.3411 | 0.0000 | postive |
| EBF1 | COL14A1 | 0.3719 | 0.0000 | postive |
| EBF1 | CRISPLD2 | 0.4194 | 0.0000 | postive |
| EBF1 | DCN | 0.3932 | 0.0000 | postive |
| EBF1 | DPYSL2 | 0.5428 | 0.0000 | postive |
| EBF1 | DPYSL3 | 0.4801 | 0.0000 | postive |
| EBF1 | DYSF | 0.4812 | 0.0000 | postive |
| EBF1 | EDNRA | 0.3480 | 0.0000 | postive |
| EBF1 | ELN | 0.4137 | 0.0000 | postive |
| EBF1 | F10 | 0.3110 | 0.0000 | postive |
| EBF1 | FBN1 | 0.3414 | 0.0000 | postive |
| EBF1 | GAS7 | 0.3813 | 0.0000 | postive |
| EBF1 | GPIHBP1 | 0.4750 | 0.0000 | postive |
| EBF1 | GRK5 | 0.4212 | 0.0000 | postive |
| EBF1 | GSN | 0.3100 | 0.0000 | postive |
| EBF1 | HSPB2 | 0.3644 | 0.0000 | postive |
| EBF1 | IGDCC4 | 0.3484 | 0.0000 | postive |
| EBF1 | ITGA1 | 0.3520 | 0.0000 | postive |
| EBF1 | KCNE4 | 0.4739 | 0.0000 | postive |
| EBF1 | LAMA2 | 0.3992 | 0.0000 | postive |
| EBF1 | LAMA4 | 0.3959 | 0.0000 | postive |
| EBF1 | LDB2 | 0.4691 | 0.0000 | postive |
| EBF1 | LHFPL6 | 0.4645 | 0.0000 | postive |
| EBF1 | LRRC32 | 0.4951 | 0.0000 | postive |
| EBF1 | LRRN4CL | 0.3774 | 0.0000 | postive |
| EBF1 | MAP1A | 0.3339 | 0.0000 | postive |
| EBF1 | MAP1B | 0.4785 | 0.0000 | postive |
| EBF1 | MCAM | 0.4610 | 0.0000 | postive |
| EBF1 | MEDAG | 0.3061 | 0.0000 | postive |
| EBF1 | MXRA7 | 0.3569 | 0.0000 | postive |
| EBF1 | MYADM | 0.3208 | 0.0000 | postive |
| EBF1 | OGN | 0.4201 | 0.0000 | postive |
| EBF1 | OLFML1 | 0.4026 | 0.0000 | postive |
| EBF1 | PCDH18 | 0.3518 | 0.0000 | postive |
| EBF1 | PDGFRA | 0.3288 | 0.0000 | postive |
| EBF1 | PDZRN3 | 0.7155 | 0.0000 | postive |
| EBF1 | PODN | 0.3734 | 0.0000 | postive |
| EBF1 | PRKG1 | 0.3792 | 0.0000 | postive |
| EBF1 | SCN1B | 0.3713 | 0.0000 | postive |
| EBF1 | SERPINF1 | 0.3468 | 0.0000 | postive |
| EBF1 | SH3RF3 | 0.3069 | 0.0000 | postive |
| EBF1 | SLIT2 | 0.5030 | 0.0000 | postive |
| EBF1 | SMOC2 | 0.4725 | 0.0000 | postive |
| EBF1 | SNED1 | 0.4359 | 0.0000 | postive |
| EBF1 | STARD8 | 0.5444 | 0.0000 | postive |
| EBF1 | SVEP1 | 0.3330 | 0.0000 | postive |
| EBF1 | SYNC | 0.3145 | 0.0000 | postive |
| EBF1 | TCF4 | 0.4468 | 0.0000 | postive |
| EBF1 | TGFB1I1 | 0.3925 | 0.0000 | postive |
| EBF1 | TGFB3 | 0.4133 | 0.0000 | postive |
| EBF1 | THBS1 | 0.3195 | 0.0000 | postive |
| EBF1 | TNFAIP8L3 | 0.3531 | 0.0000 | postive |
| EBF1 | TUBA1A | 0.6561 | 0.0000 | postive |
| EBF1 | WISP2 | 0.3183 | 0.0000 | postive |
| EGR1 | ANXA6 | 0.3130 | 0.0000 | postive |
| EGR1 | LATS2 | 0.3265 | 0.0000 | postive |
| EGR1 | MYADM | 0.3721 | 0.0000 | postive |
| EGR1 | NRP2 | 0.3222 | 0.0000 | postive |
| EGR2 | ANXA6 | 0.4087 | 0.0000 | postive |
| EGR2 | DPYSL2 | 0.3811 | 0.0000 | postive |
| EGR2 | FBN1 | 0.3044 | 0.0000 | postive |
| EGR2 | GAS7 | 0.3241 | 0.0000 | postive |
| EGR2 | IGDCC4 | 0.3090 | 0.0000 | postive |
| EGR2 | KCNE4 | 0.3073 | 0.0000 | postive |
| EGR2 | LAMA2 | 0.3108 | 0.0000 | postive |
| EGR2 | LRRC32 | 0.3996 | 0.0000 | postive |
| EGR2 | MYADM | 0.3250 | 0.0000 | postive |
| EGR2 | NRP2 | 0.3563 | 0.0000 | postive |
| EGR2 | OLFML1 | 0.3474 | 0.0000 | postive |
| EGR2 | PCDH18 | 0.3421 | 0.0000 | postive |
| EGR2 | RGS1 | 0.3026 | 0.0000 | postive |
| EGR2 | SLIT2 | 0.3529 | 0.0000 | postive |
| EGR2 | SNED1 | 0.3559 | 0.0000 | postive |
| EGR2 | TCF4 | 0.3167 | 0.0000 | postive |
| EGR2 | TGFB3 | 0.3402 | 0.0000 | postive |
| EZH2 | TUBA1A | 0.3444 | 0.0000 | postive |
| FOSL1 | CAVIN1 | 0.3237 | 0.0000 | postive |
| FOSL1 | SPHK1 | 0.3229 | 0.0000 | postive |
| FOXM1 | EFEMP1 | 0.3003 | 0.0000 | postive |
| GATA3 | ANXA6 | -0.3517 | 0.0000 | negative |
| GATA3 | CAVIN1 | -0.4406 | 0.0000 | negative |
| GATA3 | COL6A2 | -0.3225 | 0.0000 | negative |
| GATA3 | CTHRC1 | -0.3186 | 0.0000 | negative |
| GATA3 | DYSF | -0.3469 | 0.0000 | negative |
| GATA3 | EDNRA | -0.3209 | 0.0000 | negative |
| GATA3 | FBN1 | -0.3039 | 0.0000 | negative |
| GATA3 | GRK5 | -0.3220 | 0.0000 | negative |
| GATA3 | GSN | -0.3473 | 0.0000 | negative |
| GATA3 | LAMA4 | -0.3547 | 0.0000 | negative |
| GATA3 | LATS2 | -0.3183 | 0.0000 | negative |
| GATA3 | NRP2 | -0.3989 | 0.0000 | negative |
| GATA3 | RGS1 | -0.3355 | 0.0000 | negative |
| GATA3 | SPHK1 | -0.4535 | 0.0000 | negative |
| GATA3 | TGFB3 | -0.3379 | 0.0000 | negative |
| GATA3 | TNFAIP6 | -0.3437 | 0.0000 | negative |
| GATA3 | TNFAIP8L3 | -0.3119 | 0.0000 | negative |
| GATA6 | ABCC9 | 0.5537 | 0.0000 | postive |
| GATA6 | ADAM12 | 0.3244 | 0.0000 | postive |
| GATA6 | ADAMTS16 | 0.4953 | 0.0000 | postive |
| GATA6 | ADAMTS9 | 0.4896 | 0.0000 | postive |
| GATA6 | ANXA6 | 0.5968 | 0.0000 | postive |
| GATA6 | ARHGEF17 | 0.4668 | 0.0000 | postive |
| GATA6 | BGN | 0.3570 | 0.0000 | postive |
| GATA6 | CAVIN1 | 0.5025 | 0.0000 | postive |
| GATA6 | CCDC80 | 0.5172 | 0.0000 | postive |
| GATA6 | CD248 | 0.3039 | 0.0000 | postive |
| GATA6 | COL14A1 | 0.3126 | 0.0000 | postive |
| GATA6 | COL6A2 | 0.3889 | 0.0000 | postive |
| GATA6 | CRISPLD2 | 0.6105 | 0.0000 | postive |
| GATA6 | CTHRC1 | 0.4040 | 0.0000 | postive |
| GATA6 | CXCL12 | 0.3347 | 0.0000 | postive |
| GATA6 | DCN | 0.6339 | 0.0000 | postive |
| GATA6 | DPT | 0.3995 | 0.0000 | postive |
| GATA6 | DPYSL2 | 0.4031 | 0.0000 | postive |
| GATA6 | DPYSL3 | 0.6230 | 0.0000 | postive |
| GATA6 | DYSF | 0.3716 | 0.0000 | postive |
| GATA6 | EDNRA | 0.4441 | 0.0000 | postive |
| GATA6 | ELN | 0.5483 | 0.0000 | postive |
| GATA6 | F10 | 0.5604 | 0.0000 | postive |
| GATA6 | FBN1 | 0.4801 | 0.0000 | postive |
| GATA6 | GALNT15 | 0.3866 | 0.0000 | postive |
| GATA6 | GAS7 | 0.5220 | 0.0000 | postive |
| GATA6 | GPIHBP1 | 0.5300 | 0.0000 | postive |
| GATA6 | GRK5 | 0.3751 | 0.0000 | postive |
| GATA6 | GSN | 0.4749 | 0.0000 | postive |
| GATA6 | HSPB2 | 0.5378 | 0.0000 | postive |
| GATA6 | ITGA1 | 0.4438 | 0.0000 | postive |
| GATA6 | JAM3 | 0.3726 | 0.0000 | postive |
| GATA6 | KCNE4 | 0.6108 | 0.0000 | postive |
| GATA6 | LAMA2 | 0.5074 | 0.0000 | postive |
| GATA6 | LAMA4 | 0.4573 | 0.0000 | postive |
| GATA6 | LATS2 | 0.4504 | 0.0000 | postive |
| GATA6 | LDB2 | 0.3824 | 0.0000 | postive |
| GATA6 | LHFPL6 | 0.4974 | 0.0000 | postive |
| GATA6 | LRRC32 | 0.6075 | 0.0000 | postive |
| GATA6 | LRRN4CL | 0.5486 | 0.0000 | postive |
| GATA6 | MAP1A | 0.3075 | 0.0000 | postive |
| GATA6 | MAP1B | 0.3481 | 0.0000 | postive |
| GATA6 | MCAM | 0.4977 | 0.0000 | postive |
| GATA6 | MEDAG | 0.4905 | 0.0000 | postive |
| GATA6 | MXRA7 | 0.5572 | 0.0000 | postive |
| GATA6 | MYADM | 0.4544 | 0.0000 | postive |
| GATA6 | NRP2 | 0.4679 | 0.0000 | postive |
| GATA6 | NXPH3 | 0.4302 | 0.0000 | postive |
| GATA6 | OGN | 0.4821 | 0.0000 | postive |
| GATA6 | OLFML1 | 0.5455 | 0.0000 | postive |
| GATA6 | OLFML3 | 0.3374 | 0.0000 | postive |
| GATA6 | PCDH18 | 0.3152 | 0.0000 | postive |
| GATA6 | PDGFRA | 0.3427 | 0.0000 | postive |
| GATA6 | PDZRN3 | 0.5568 | 0.0000 | postive |
| GATA6 | PID1 | 0.4481 | 0.0000 | postive |
| GATA6 | PODN | 0.5836 | 0.0000 | postive |
| GATA6 | PRKG1 | 0.4888 | 0.0000 | postive |
| GATA6 | RGCC | 0.3595 | 0.0000 | postive |
| GATA6 | RGS1 | 0.3074 | 0.0000 | postive |
| GATA6 | SCN1B | 0.4107 | 0.0000 | postive |
| GATA6 | SERPINF1 | 0.4885 | 0.0000 | postive |
| GATA6 | SH3RF3 | 0.4029 | 0.0000 | postive |
| GATA6 | SLIT2 | 0.4478 | 0.0000 | postive |
| GATA6 | SMOC2 | 0.6887 | 0.0000 | postive |
| GATA6 | SNED1 | 0.5609 | 0.0000 | postive |
| GATA6 | SPON1 | 0.5559 | 0.0000 | postive |
| GATA6 | SRPX | 0.3321 | 0.0000 | postive |
| GATA6 | STARD8 | 0.4072 | 0.0000 | postive |
| GATA6 | SVEP1 | 0.5289 | 0.0000 | postive |
| GATA6 | SYNC | 0.5032 | 0.0000 | postive |
| GATA6 | TCF4 | 0.3105 | 0.0000 | postive |
| GATA6 | TGFB1I1 | 0.6046 | 0.0000 | postive |
| GATA6 | TGFB3 | 0.5289 | 0.0000 | postive |
| GATA6 | THBS1 | 0.4785 | 0.0000 | postive |
| GATA6 | TIMP2 | 0.4668 | 0.0000 | postive |
| GATA6 | TNFAIP6 | 0.4096 | 0.0000 | postive |
| GATA6 | TNFAIP8L3 | 0.5912 | 0.0000 | postive |
| GATA6 | TWIST2 | 0.3529 | 0.0000 | postive |
| GATA6 | WISP2 | 0.5633 | 0.0000 | postive |
| GRHL2 | ANXA6 | -0.4652 | 0.0000 | negative |
| GRHL2 | BGN | -0.3410 | 0.0000 | negative |
| GRHL2 | CAVIN1 | -0.3839 | 0.0000 | negative |
| GRHL2 | COL6A2 | -0.3948 | 0.0000 | negative |
| GRHL2 | CTHRC1 | -0.3211 | 0.0000 | negative |
| GRHL2 | DPYSL2 | -0.3305 | 0.0000 | negative |
| GRHL2 | DYSF | -0.3911 | 0.0000 | negative |
| GRHL2 | EDNRA | -0.3097 | 0.0000 | negative |
| GRHL2 | FBN1 | -0.3177 | 0.0000 | negative |
| GRHL2 | GRK5 | -0.3383 | 0.0000 | negative |
| GRHL2 | GSN | -0.3034 | 0.0000 | negative |
| GRHL2 | KCNE4 | -0.3232 | 0.0000 | negative |
| GRHL2 | LAMA4 | -0.3860 | 0.0000 | negative |
| GRHL2 | LRRC32 | -0.3291 | 0.0000 | negative |
| GRHL2 | MCAM | -0.3490 | 0.0000 | negative |
| GRHL2 | MXRA7 | -0.3152 | 0.0000 | negative |
| GRHL2 | NRP2 | -0.3277 | 0.0000 | negative |
| GRHL2 | P3H1 | -0.3281 | 0.0000 | negative |
| GRHL2 | RGS1 | -0.3113 | 0.0000 | negative |
| GRHL2 | SCN1B | -0.4170 | 0.0000 | negative |
| GRHL2 | SERPINF1 | -0.3248 | 0.0000 | negative |
| GRHL2 | SH3RF3 | -0.3566 | 0.0000 | negative |
| GRHL2 | SLIT2 | -0.3160 | 0.0000 | negative |
| GRHL2 | SPHK1 | -0.3572 | 0.0000 | negative |
| GRHL2 | STARD8 | -0.3657 | 0.0000 | negative |
| GRHL2 | TGFB1I1 | -0.3848 | 0.0000 | negative |
| GRHL2 | TGFB3 | -0.3443 | 0.0000 | negative |
| GRHL2 | TNFAIP6 | -0.3257 | 0.0000 | negative |
| GRHL2 | TNFAIP8L3 | -0.3018 | 0.0000 | negative |
| IRF4 | ANXA6 | 0.4358 | 0.0000 | postive |
| IRF4 | OLFML1 | 0.3004 | 0.0000 | postive |
| IRF4 | PID1 | 0.3086 | 0.0000 | postive |
| IRF4 | RGS1 | 0.4886 | 0.0000 | postive |
| JUND | GPIHBP1 | 0.3161 | 0.0000 | postive |
| JUND | RGCC | 0.3703 | 0.0000 | postive |
| LHX2 | ATP8B2 | 0.4200 | 0.0000 | postive |
| LHX2 | DPYSL2 | 0.3322 | 0.0000 | postive |
| LHX2 | TUBA1A | 0.4886 | 0.0000 | postive |
| LIN9 | LRRN4CL | -0.3062 | 0.0000 | negative |
| MEF2C | ABCC9 | 0.4721 | 0.0000 | postive |
| MEF2C | ADAMTS9 | 0.4577 | 0.0000 | postive |
| MEF2C | ANXA6 | 0.6608 | 0.0000 | postive |
| MEF2C | ARHGEF17 | 0.4342 | 0.0000 | postive |
| MEF2C | CCDC80 | 0.3100 | 0.0000 | postive |
| MEF2C | COL14A1 | 0.3557 | 0.0000 | postive |
| MEF2C | CRISPLD2 | 0.3779 | 0.0000 | postive |
| MEF2C | CXCL12 | 0.4433 | 0.0000 | postive |
| MEF2C | DCN | 0.3608 | 0.0000 | postive |
| MEF2C | DPYSL2 | 0.4716 | 0.0000 | postive |
| MEF2C | DPYSL3 | 0.3671 | 0.0000 | postive |
| MEF2C | DYSF | 0.4419 | 0.0000 | postive |
| MEF2C | EDNRA | 0.4056 | 0.0000 | postive |
| MEF2C | ELN | 0.3836 | 0.0000 | postive |
| MEF2C | FBN1 | 0.4198 | 0.0000 | postive |
| MEF2C | GAS7 | 0.4597 | 0.0000 | postive |
| MEF2C | GPIHBP1 | 0.4146 | 0.0000 | postive |
| MEF2C | GRK5 | 0.4433 | 0.0000 | postive |
| MEF2C | HSPB2 | 0.3014 | 0.0000 | postive |
| MEF2C | IGDCC4 | 0.4580 | 0.0000 | postive |
| MEF2C | ITGA1 | 0.4591 | 0.0000 | postive |
| MEF2C | KCNE4 | 0.4328 | 0.0000 | postive |
| MEF2C | LAMA2 | 0.4306 | 0.0000 | postive |
| MEF2C | LAMA4 | 0.4808 | 0.0000 | postive |
| MEF2C | LATS2 | 0.3690 | 0.0000 | postive |
| MEF2C | LDB2 | 0.5488 | 0.0000 | postive |
| MEF2C | LHFPL6 | 0.4785 | 0.0000 | postive |
| MEF2C | LRRC32 | 0.5095 | 0.0000 | postive |
| MEF2C | LRRN4CL | 0.3622 | 0.0000 | postive |
| MEF2C | MAP1A | 0.5083 | 0.0000 | postive |
| MEF2C | MAP1B | 0.4108 | 0.0000 | postive |
| MEF2C | MCAM | 0.4594 | 0.0000 | postive |
| MEF2C | MYADM | 0.3210 | 0.0000 | postive |
| MEF2C | NRP2 | 0.3346 | 0.0000 | postive |
| MEF2C | OGN | 0.4067 | 0.0000 | postive |
| MEF2C | OLFML1 | 0.4178 | 0.0000 | postive |
| MEF2C | PCDH18 | 0.3695 | 0.0000 | postive |
| MEF2C | PDGFRA | 0.4118 | 0.0000 | postive |
| MEF2C | PDZRN3 | 0.3364 | 0.0000 | postive |
| MEF2C | PID1 | 0.3575 | 0.0000 | postive |
| MEF2C | PODN | 0.3864 | 0.0000 | postive |
| MEF2C | PRKG1 | 0.5030 | 0.0000 | postive |
| MEF2C | RGS1 | 0.3285 | 0.0000 | postive |
| MEF2C | SCN1B | 0.3411 | 0.0000 | postive |
| MEF2C | SERPINF1 | 0.3292 | 0.0000 | postive |
| MEF2C | SH3RF3 | 0.3880 | 0.0000 | postive |
| MEF2C | SLIT2 | 0.4378 | 0.0000 | postive |
| MEF2C | SMOC2 | 0.4287 | 0.0000 | postive |
| MEF2C | SNED1 | 0.4831 | 0.0000 | postive |
| MEF2C | SPON1 | 0.3081 | 0.0000 | postive |
| MEF2C | STARD8 | 0.5737 | 0.0000 | postive |
| MEF2C | SVEP1 | 0.3416 | 0.0000 | postive |
| MEF2C | SYNC | 0.3934 | 0.0000 | postive |
| MEF2C | TCF4 | 0.4296 | 0.0000 | postive |
| MEF2C | TGFB1I1 | 0.4058 | 0.0000 | postive |
| MEF2C | TGFB3 | 0.4116 | 0.0000 | postive |
| MEF2C | THBS1 | 0.3894 | 0.0000 | postive |
| MEF2C | TNFAIP8L3 | 0.3169 | 0.0000 | postive |
| MEIS1 | COL14A1 | 0.3122 | 0.0000 | postive |
| MEIS1 | DPYSL3 | 0.3295 | 0.0000 | postive |
| MEIS1 | ELN | 0.3030 | 0.0000 | postive |
| MEIS1 | LAMA2 | 0.3126 | 0.0000 | postive |
| MEIS1 | LRRN4CL | 0.3203 | 0.0000 | postive |
| MEIS1 | OGN | 0.3349 | 0.0000 | postive |
| MEIS1 | PDZRN3 | 0.3123 | 0.0000 | postive |
| MEIS1 | PODN | 0.3426 | 0.0000 | postive |
| MEIS1 | SMOC2 | 0.3656 | 0.0000 | postive |
| MEIS1 | SPON1 | 0.4610 | 0.0000 | postive |
| MYC | SPHK1 | 0.3247 | 0.0000 | postive |
| MYH11 | ABCC9 | 0.4995 | 0.0000 | postive |
| MYH11 | ANXA6 | 0.5740 | 0.0000 | postive |
| MYH11 | ARHGEF17 | 0.4666 | 0.0000 | postive |
| MYH11 | CAVIN1 | 0.3857 | 0.0000 | postive |
| MYH11 | CRISPLD2 | 0.4129 | 0.0000 | postive |
| MYH11 | DCN | 0.4282 | 0.0000 | postive |
| MYH11 | DPYSL2 | 0.3320 | 0.0000 | postive |
| MYH11 | DPYSL3 | 0.6438 | 0.0000 | postive |
| MYH11 | DYSF | 0.3057 | 0.0000 | postive |
| MYH11 | EDNRA | 0.4091 | 0.0000 | postive |
| MYH11 | ELN | 0.4928 | 0.0000 | postive |
| MYH11 | GAS7 | 0.4344 | 0.0000 | postive |
| MYH11 | GPIHBP1 | 0.3317 | 0.0000 | postive |
| MYH11 | GSN | 0.4564 | 0.0000 | postive |
| MYH11 | HSPB2 | 0.3367 | 0.0000 | postive |
| MYH11 | ITGA1 | 0.4833 | 0.0000 | postive |
| MYH11 | JAM3 | 0.3782 | 0.0000 | postive |
| MYH11 | KCNE4 | 0.4898 | 0.0000 | postive |
| MYH11 | LAMA2 | 0.4409 | 0.0000 | postive |
| MYH11 | LAMA4 | 0.3809 | 0.0000 | postive |
| MYH11 | LATS2 | 0.3469 | 0.0000 | postive |
| MYH11 | LHFPL6 | 0.4652 | 0.0000 | postive |
| MYH11 | LRRC32 | 0.3676 | 0.0000 | postive |
| MYH11 | LRRN4CL | 0.5043 | 0.0000 | postive |
| MYH11 | MAP1A | 0.3461 | 0.0000 | postive |
| MYH11 | MAP1B | 0.3933 | 0.0000 | postive |
| MYH11 | MCAM | 0.4842 | 0.0000 | postive |
| MYH11 | MXRA7 | 0.5684 | 0.0000 | postive |
| MYH11 | MYADM | 0.3012 | 0.0000 | postive |
| MYH11 | NRP2 | 0.3484 | 0.0000 | postive |
| MYH11 | NXPH3 | 0.4520 | 0.0000 | postive |
| MYH11 | OGN | 0.4531 | 0.0000 | postive |
| MYH11 | OLFML1 | 0.3279 | 0.0000 | postive |
| MYH11 | PDZRN3 | 0.6315 | 0.0000 | postive |
| MYH11 | PODN | 0.4823 | 0.0000 | postive |
| MYH11 | PRKG1 | 0.5901 | 0.0000 | postive |
| MYH11 | SLC24A3 | 0.3426 | 0.0000 | postive |
| MYH11 | SLIT2 | 0.3838 | 0.0000 | postive |
| MYH11 | SMOC2 | 0.8011 | 0.0000 | postive |
| MYH11 | SNED1 | 0.3049 | 0.0000 | postive |
| MYH11 | SPON1 | 0.3801 | 0.0000 | postive |
| MYH11 | STARD8 | 0.3515 | 0.0000 | postive |
| MYH11 | SYNC | 0.5641 | 0.0000 | postive |
| MYH11 | TGFB1I1 | 0.6998 | 0.0000 | postive |
| MYH11 | TGFB3 | 0.4337 | 0.0000 | postive |
| MYH11 | TNFAIP8L3 | 0.4439 | 0.0000 | postive |
| MYH11 | WISP2 | 0.4379 | 0.0000 | postive |
| NCAPG | LRRN4CL | -0.3330 | 0.0000 | negative |
| NCAPG | TUBA1A | 0.3021 | 0.0000 | postive |
| NFATC1 | ADAMTS16 | 0.3589 | 0.0000 | postive |
| NFATC1 | ADAMTS9 | 0.5399 | 0.0000 | postive |
| NFATC1 | ANXA6 | 0.5603 | 0.0000 | postive |
| NFATC1 | BGN | 0.3463 | 0.0000 | postive |
| NFATC1 | CCDC80 | 0.4918 | 0.0000 | postive |
| NFATC1 | COL6A2 | 0.3812 | 0.0000 | postive |
| NFATC1 | CRISPLD2 | 0.4360 | 0.0000 | postive |
| NFATC1 | CTHRC1 | 0.3484 | 0.0000 | postive |
| NFATC1 | CXCL12 | 0.3327 | 0.0000 | postive |
| NFATC1 | DCN | 0.4346 | 0.0000 | postive |
| NFATC1 | DPT | 0.3883 | 0.0000 | postive |
| NFATC1 | DPYSL2 | 0.3160 | 0.0000 | postive |
| NFATC1 | DYSF | 0.3272 | 0.0000 | postive |
| NFATC1 | ELN | 0.3586 | 0.0000 | postive |
| NFATC1 | F10 | 0.4884 | 0.0000 | postive |
| NFATC1 | FBN1 | 0.3268 | 0.0000 | postive |
| NFATC1 | GAS7 | 0.3938 | 0.0000 | postive |
| NFATC1 | GPIHBP1 | 0.5111 | 0.0000 | postive |
| NFATC1 | GRK5 | 0.3485 | 0.0000 | postive |
| NFATC1 | GSN | 0.3303 | 0.0000 | postive |
| NFATC1 | HSPB2 | 0.3815 | 0.0000 | postive |
| NFATC1 | IGDCC4 | 0.3062 | 0.0000 | postive |
| NFATC1 | KCNE4 | 0.3715 | 0.0000 | postive |
| NFATC1 | LAMA2 | 0.3430 | 0.0000 | postive |
| NFATC1 | LAMA4 | 0.3658 | 0.0000 | postive |
| NFATC1 | LATS2 | 0.3390 | 0.0000 | postive |
| NFATC1 | LDB2 | 0.5215 | 0.0000 | postive |
| NFATC1 | LHFPL6 | 0.3518 | 0.0000 | postive |
| NFATC1 | LRRC32 | 0.5464 | 0.0000 | postive |
| NFATC1 | LRRN4CL | 0.3549 | 0.0000 | postive |
| NFATC1 | MAP1A | 0.3020 | 0.0000 | postive |
| NFATC1 | MCAM | 0.3014 | 0.0000 | postive |
| NFATC1 | MEDAG | 0.5150 | 0.0000 | postive |
| NFATC1 | MYADM | 0.4829 | 0.0000 | postive |
| NFATC1 | NRP2 | 0.3053 | 0.0000 | postive |
| NFATC1 | OGN | 0.3160 | 0.0000 | postive |
| NFATC1 | OLFML1 | 0.4010 | 0.0000 | postive |
| NFATC1 | OLFML3 | 0.4845 | 0.0000 | postive |
| NFATC1 | PDGFRA | 0.3148 | 0.0000 | postive |
| NFATC1 | PODN | 0.3077 | 0.0000 | postive |
| NFATC1 | RGCC | 0.5365 | 0.0000 | postive |
| NFATC1 | SCN1B | 0.3276 | 0.0000 | postive |
| NFATC1 | SERPINF1 | 0.4356 | 0.0000 | postive |
| NFATC1 | SH3RF3 | 0.4548 | 0.0000 | postive |
| NFATC1 | SLIT2 | 0.4490 | 0.0000 | postive |
| NFATC1 | SMOC2 | 0.3135 | 0.0000 | postive |
| NFATC1 | SNED1 | 0.5298 | 0.0000 | postive |
| NFATC1 | STARD8 | 0.3463 | 0.0000 | postive |
| NFATC1 | SVEP1 | 0.5022 | 0.0000 | postive |
| NFATC1 | TGFB1I1 | 0.3407 | 0.0000 | postive |
| NFATC1 | TGFB3 | 0.3187 | 0.0000 | postive |
| NFATC1 | THBS1 | 0.3762 | 0.0000 | postive |
| NFATC1 | TNFAIP6 | 0.4014 | 0.0000 | postive |
| NFATC1 | TNFAIP8L3 | 0.3963 | 0.0000 | postive |
| NFATC1 | TWIST2 | 0.3011 | 0.0000 | postive |
| NFATC1 | WISP2 | 0.3686 | 0.0000 | postive |
| NFIC | ANXA6 | 0.5306 | 0.0000 | postive |
| NFIC | ARHGEF17 | 0.3353 | 0.0000 | postive |
| NFIC | CAVIN1 | 0.3892 | 0.0000 | postive |
| NFIC | CHI3L1 | 0.7103 | 0.0000 | postive |
| NFIC | COL6A2 | 0.5408 | 0.0000 | postive |
| NFIC | DPYSL2 | 0.3371 | 0.0000 | postive |
| NFIC | DPYSL3 | 0.3258 | 0.0000 | postive |
| NFIC | DYSF | 0.3019 | 0.0000 | postive |
| NFIC | FBN1 | 0.3022 | 0.0000 | postive |
| NFIC | GAS7 | 0.3004 | 0.0000 | postive |
| NFIC | KCNE4 | 0.4646 | 0.0000 | postive |
| NFIC | LAMA4 | 0.4024 | 0.0000 | postive |
| NFIC | LYVE1 | 0.7181 | 0.0000 | postive |
| NFIC | MAP1A | 0.3399 | 0.0000 | postive |
| NFIC | MAP1B | 0.3184 | 0.0000 | postive |
| NFIC | MCAM | 0.3253 | 0.0000 | postive |
| NFIC | NES | 0.3086 | 0.0000 | postive |
| NFIC | PDGFRA | 0.3096 | 0.0000 | postive |
| NFIC | PRKG1 | 0.3010 | 0.0000 | postive |
| NFIC | SCN1B | 0.5288 | 0.0000 | postive |
| NFIC | SLIT2 | 0.3018 | 0.0000 | postive |
| NFIC | STARD8 | 0.3798 | 0.0000 | postive |
| NFIC | SYNC | 0.4033 | 0.0000 | postive |
| NFIC | TGFB1I1 | 0.4661 | 0.0000 | postive |
| NFIC | TGFB3 | 0.3423 | 0.0000 | postive |
| NFIC | TNFAIP8L3 | 0.3621 | 0.0000 | postive |
| NR2F1 | ADAMTS12 | 0.3145 | 0.0000 | postive |
| NR2F1 | ANXA6 | 0.4022 | 0.0000 | postive |
| NR2F1 | COL14A1 | 0.4280 | 0.0000 | postive |
| NR2F1 | CYTL1 | 0.3515 | 0.0000 | postive |
| NR2F1 | DPYSL2 | 0.3528 | 0.0000 | postive |
| NR2F1 | EDNRA | 0.3054 | 0.0000 | postive |
| NR2F1 | ELN | 0.3700 | 0.0000 | postive |
| NR2F1 | GPIHBP1 | 0.3385 | 0.0000 | postive |
| NR2F1 | IGDCC4 | 0.3466 | 0.0000 | postive |
| NR2F1 | LAMA2 | 0.4969 | 0.0000 | postive |
| NR2F1 | LAMA4 | 0.3978 | 0.0000 | postive |
| NR2F1 | LDB2 | 0.4557 | 0.0000 | postive |
| NR2F1 | LHFPL6 | 0.3543 | 0.0000 | postive |
| NR2F1 | LRRN4CL | 0.3351 | 0.0000 | postive |
| NR2F1 | MCAM | 0.3055 | 0.0000 | postive |
| NR2F1 | OGN | 0.3581 | 0.0000 | postive |
| NR2F1 | OLFML1 | 0.3098 | 0.0000 | postive |
| NR2F1 | PCDH18 | 0.4259 | 0.0000 | postive |
| NR2F1 | PDGFRA | 0.4488 | 0.0000 | postive |
| NR2F1 | PODN | 0.4361 | 0.0000 | postive |
| NR2F1 | SH3RF3 | 0.3544 | 0.0000 | postive |
| NR2F1 | SLIT2 | 0.3908 | 0.0000 | postive |
| NR2F1 | SNED1 | 0.4430 | 0.0000 | postive |
| NR2F1 | TGFB1I1 | 0.3524 | 0.0000 | postive |
| PBX1 | SPHK1 | -0.3157 | 0.0000 | negative |
| POLR3G | SPHK1 | 0.3128 | 0.0000 | postive |
| PRDM1 | DYSF | 0.3359 | 0.0000 | postive |
| PRDM1 | EDNRA | 0.3366 | 0.0000 | postive |
| PRDM1 | GRK5 | 0.3094 | 0.0000 | postive |
| PRDM1 | LATS2 | 0.3961 | 0.0000 | postive |
| PRDM1 | RGS1 | 0.4307 | 0.0000 | postive |
| PRDM1 | SPHK1 | 0.3430 | 0.0000 | postive |
| PRDM1 | TNFAIP6 | 0.4396 | 0.0000 | postive |
| SOX17 | ABCC9 | 0.4307 | 0.0000 | postive |
| SOX17 | ADAMTS16 | 0.4059 | 0.0000 | postive |
| SOX17 | ADAMTS9 | 0.8221 | 0.0000 | postive |
| SOX17 | ANXA6 | 0.4988 | 0.0000 | postive |
| SOX17 | ARHGEF17 | 0.3482 | 0.0000 | postive |
| SOX17 | BGN | 0.3033 | 0.0000 | postive |
| SOX17 | CAVIN1 | 0.3234 | 0.0000 | postive |
| SOX17 | CCDC80 | 0.5062 | 0.0000 | postive |
| SOX17 | COL14A1 | 0.4503 | 0.0000 | postive |
| SOX17 | CRISPLD2 | 0.6201 | 0.0000 | postive |
| SOX17 | CXCL12 | 0.3597 | 0.0000 | postive |
| SOX17 | DCN | 0.5633 | 0.0000 | postive |
| SOX17 | DPT | 0.3980 | 0.0000 | postive |
| SOX17 | DPYSL2 | 0.3641 | 0.0000 | postive |
| SOX17 | DPYSL3 | 0.3453 | 0.0000 | postive |
| SOX17 | DYSF | 0.4120 | 0.0000 | postive |
| SOX17 | EDNRA | 0.3161 | 0.0000 | postive |
| SOX17 | ELN | 0.5844 | 0.0000 | postive |
| SOX17 | F10 | 0.6604 | 0.0000 | postive |
| SOX17 | FBN1 | 0.3631 | 0.0000 | postive |
| SOX17 | GALNT15 | 0.3556 | 0.0000 | postive |
| SOX17 | GAS7 | 0.4603 | 0.0000 | postive |
| SOX17 | GPIHBP1 | 0.7925 | 0.0000 | postive |
| SOX17 | GRK5 | 0.4306 | 0.0000 | postive |
| SOX17 | GSN | 0.3891 | 0.0000 | postive |
| SOX17 | HSPB2 | 0.5526 | 0.0000 | postive |
| SOX17 | IGDCC4 | 0.3203 | 0.0000 | postive |
| SOX17 | ITGA1 | 0.3853 | 0.0000 | postive |
| SOX17 | KCNE4 | 0.6189 | 0.0000 | postive |
| SOX17 | LAMA2 | 0.5179 | 0.0000 | postive |
| SOX17 | LAMA4 | 0.4460 | 0.0000 | postive |
| SOX17 | LATS2 | 0.3897 | 0.0000 | postive |
| SOX17 | LDB2 | 0.6772 | 0.0000 | postive |
| SOX17 | LHFPL6 | 0.5242 | 0.0000 | postive |
| SOX17 | LRRC32 | 0.6986 | 0.0000 | postive |
| SOX17 | LRRN4CL | 0.5821 | 0.0000 | postive |
| SOX17 | MCAM | 0.5646 | 0.0000 | postive |
| SOX17 | MEDAG | 0.6815 | 0.0000 | postive |
| SOX17 | MXRA7 | 0.3445 | 0.0000 | postive |
| SOX17 | MYADM | 0.5380 | 0.0000 | postive |
| SOX17 | OGN | 0.5587 | 0.0000 | postive |
| SOX17 | OLFML1 | 0.5310 | 0.0000 | postive |
| SOX17 | PDGFRA | 0.4707 | 0.0000 | postive |
| SOX17 | PDZRN3 | 0.3628 | 0.0000 | postive |
| SOX17 | PODN | 0.4816 | 0.0000 | postive |
| SOX17 | PRKG1 | 0.3787 | 0.0000 | postive |
| SOX17 | RGCC | 0.6719 | 0.0000 | postive |
| SOX17 | SCN1B | 0.3427 | 0.0000 | postive |
| SOX17 | SERPINF1 | 0.5009 | 0.0000 | postive |
| SOX17 | SH3RF3 | 0.3866 | 0.0000 | postive |
| SOX17 | SLC2A3 | 0.3249 | 0.0000 | postive |
| SOX17 | SLIT2 | 0.3217 | 0.0000 | postive |
| SOX17 | SMOC2 | 0.5736 | 0.0000 | postive |
| SOX17 | SNED1 | 0.6276 | 0.0000 | postive |
| SOX17 | SPON1 | 0.4096 | 0.0000 | postive |
| SOX17 | STARD8 | 0.4910 | 0.0000 | postive |
| SOX17 | SVEP1 | 0.6119 | 0.0000 | postive |
| SOX17 | TCF4 | 0.3312 | 0.0000 | postive |
| SOX17 | TGFB1I1 | 0.4327 | 0.0000 | postive |
| SOX17 | TGFB3 | 0.3608 | 0.0000 | postive |
| SOX17 | THBS1 | 0.4734 | 0.0000 | postive |
| SOX17 | TNFAIP6 | 0.4469 | 0.0000 | postive |
| SOX17 | TNFAIP8L3 | 0.5660 | 0.0000 | postive |
| SOX17 | TWIST2 | 0.3157 | 0.0000 | postive |
| SOX17 | WISP2 | 0.5087 | 0.0000 | postive |
| SOX4 | TUBA1A | 0.4299 | 0.0000 | postive |
| SRF | ADAMTS9 | 0.3474 | 0.0000 | postive |
| SRF | ANXA6 | 0.5445 | 0.0000 | postive |
| SRF | ARHGEF17 | 0.4031 | 0.0000 | postive |
| SRF | CAVIN1 | 0.4241 | 0.0000 | postive |
| SRF | COL14A1 | 0.4497 | 0.0000 | postive |
| SRF | COL6A2 | 0.3088 | 0.0000 | postive |
| SRF | CRISPLD2 | 0.3424 | 0.0000 | postive |
| SRF | CYTL1 | 0.3922 | 0.0000 | postive |
| SRF | DCN | 0.3145 | 0.0000 | postive |
| SRF | DPYSL2 | 0.3411 | 0.0000 | postive |
| SRF | DPYSL3 | 0.3558 | 0.0000 | postive |
| SRF | DYSF | 0.3586 | 0.0000 | postive |
| SRF | EDNRA | 0.3530 | 0.0000 | postive |
| SRF | ELN | 0.3278 | 0.0000 | postive |
| SRF | GAS7 | 0.3259 | 0.0000 | postive |
| SRF | GPIHBP1 | 0.3408 | 0.0000 | postive |
| SRF | GRK5 | 0.3251 | 0.0000 | postive |
| SRF | GSN | 0.3517 | 0.0000 | postive |
| SRF | IGDCC4 | 0.3182 | 0.0000 | postive |
| SRF | ITGA1 | 0.3567 | 0.0000 | postive |
| SRF | KCNE4 | 0.4626 | 0.0000 | postive |
| SRF | LAMA2 | 0.3370 | 0.0000 | postive |
| SRF | LAMA4 | 0.4820 | 0.0000 | postive |
| SRF | LATS2 | 0.3569 | 0.0000 | postive |
| SRF | LDB2 | 0.3415 | 0.0000 | postive |
| SRF | LHFPL6 | 0.3740 | 0.0000 | postive |
| SRF | LRRC32 | 0.3375 | 0.0000 | postive |
| SRF | MCAM | 0.4729 | 0.0000 | postive |
| SRF | MXRA7 | 0.3566 | 0.0000 | postive |
| SRF | MYADM | 0.3982 | 0.0000 | postive |
| SRF | NRP2 | 0.3378 | 0.0000 | postive |
| SRF | OGN | 0.4023 | 0.0000 | postive |
| SRF | PCDH18 | 0.3835 | 0.0000 | postive |
| SRF | PDGFRA | 0.3904 | 0.0000 | postive |
| SRF | PDZRN3 | 0.3285 | 0.0000 | postive |
| SRF | PRKG1 | 0.3665 | 0.0000 | postive |
| SRF | SCN1B | 0.3069 | 0.0000 | postive |
| SRF | SERPINF1 | 0.3281 | 0.0000 | postive |
| SRF | SH3RF3 | 0.3566 | 0.0000 | postive |
| SRF | SLIT2 | 0.3348 | 0.0000 | postive |
| SRF | SMOC2 | 0.4052 | 0.0000 | postive |
| SRF | SNED1 | 0.3755 | 0.0000 | postive |
| SRF | SRPX | 0.3474 | 0.0000 | postive |
| SRF | STARD8 | 0.3620 | 0.0000 | postive |
| SRF | SYNC | 0.4025 | 0.0000 | postive |
| SRF | TGFB1I1 | 0.5032 | 0.0000 | postive |
| SRF | TGFB3 | 0.3932 | 0.0000 | postive |
| SRF | THBS1 | 0.3250 | 0.0000 | postive |
| SRF | TNFAIP6 | 0.3161 | 0.0000 | postive |
| SRF | TNFAIP8L3 | 0.3288 | 0.0000 | postive |
| STAT1 | CAVIN1 | 0.3111 | 0.0000 | postive |
| STAT1 | RGS1 | 0.4964 | 0.0000 | postive |
| STAT1 | SPHK1 | 0.3030 | 0.0000 | postive |
| STAT1 | TNFAIP6 | 0.3471 | 0.0000 | postive |
| TEAD1 | ABCC9 | 0.3300 | 0.0000 | postive |
| TEAD1 | ARHGEF17 | 0.4092 | 0.0000 | postive |
| TEAD1 | CAVIN1 | 0.3217 | 0.0000 | postive |
| TEAD1 | DPYSL2 | 0.3846 | 0.0000 | postive |
| TEAD1 | DPYSL3 | 0.3209 | 0.0000 | postive |
| TEAD1 | EDNRA | 0.3445 | 0.0000 | postive |
| TEAD1 | FBN1 | 0.3343 | 0.0000 | postive |
| TEAD1 | ITGA1 | 0.3397 | 0.0000 | postive |
| TEAD1 | JAM3 | 0.3062 | 0.0000 | postive |
| TEAD1 | LAMA2 | 0.3078 | 0.0000 | postive |
| TEAD1 | LAMA4 | 0.3066 | 0.0000 | postive |
| TEAD1 | LATS2 | 0.5207 | 0.0000 | postive |
| TEAD1 | LHFPL6 | 0.3870 | 0.0000 | postive |
| TEAD1 | MFAP5 | 0.3258 | 0.0000 | postive |
| TEAD1 | MXRA7 | 0.3235 | 0.0000 | postive |
| TEAD1 | MYADM | 0.3375 | 0.0000 | postive |
| TEAD1 | SYNC | 0.3403 | 0.0000 | postive |
| TEAD1 | TIMP2 | 0.3301 | 0.0000 | postive |
| TEAD4 | CAVIN1 | 0.5605 | 0.0000 | postive |
| TEAD4 | COL6A2 | 0.3058 | 0.0000 | postive |
| TEAD4 | EFEMP1 | 0.4456 | 0.0000 | postive |
| TEAD4 | MFAP5 | 0.3414 | 0.0000 | postive |
| TEAD4 | SCN1B | 0.3197 | 0.0000 | postive |
| TEAD4 | SLC2A3 | 0.3147 | 0.0000 | postive |
| TEAD4 | SPHK1 | 0.5838 | 0.0000 | postive |
| TEAD4 | TNFAIP6 | 0.3361 | 0.0000 | postive |
| TEAD4 | TNFAIP8L3 | 0.3035 | 0.0000 | postive |
| TP63 | ANXA6 | -0.3027 | 0.0000 | negative |
| TP63 | DPYSL2 | -0.3044 | 0.0000 | negative |
| TP73 | ATP8B2 | 0.3122 | 0.0000 | postive |
| TP73 | TUBA1A | 0.3685 | 0.0000 | postive |
| WWTR1 | ABCC9 | 0.5381 | 0.0000 | postive |
| WWTR1 | ADAM12 | 0.4152 | 0.0000 | postive |
| WWTR1 | ADAMTS12 | 0.4443 | 0.0000 | postive |
| WWTR1 | ADAMTS9 | 0.4837 | 0.0000 | postive |
| WWTR1 | ANXA6 | 0.6439 | 0.0000 | postive |
| WWTR1 | ARHGEF17 | 0.5687 | 0.0000 | postive |
| WWTR1 | BGN | 0.3483 | 0.0000 | postive |
| WWTR1 | CAVIN1 | 0.5539 | 0.0000 | postive |
| WWTR1 | CCDC80 | 0.3973 | 0.0000 | postive |
| WWTR1 | COL14A1 | 0.4009 | 0.0000 | postive |
| WWTR1 | COL6A2 | 0.4514 | 0.0000 | postive |
| WWTR1 | CRISPLD2 | 0.4943 | 0.0000 | postive |
| WWTR1 | CTHRC1 | 0.3833 | 0.0000 | postive |
| WWTR1 | CXCL12 | 0.4327 | 0.0000 | postive |
| WWTR1 | DCN | 0.4815 | 0.0000 | postive |
| WWTR1 | DPYSL2 | 0.4531 | 0.0000 | postive |
| WWTR1 | DPYSL3 | 0.6360 | 0.0000 | postive |
| WWTR1 | DYSF | 0.4945 | 0.0000 | postive |
| WWTR1 | EDNRA | 0.5909 | 0.0000 | postive |
| WWTR1 | ELN | 0.4422 | 0.0000 | postive |
| WWTR1 | FBN1 | 0.5244 | 0.0000 | postive |
| WWTR1 | GALNT15 | 0.3352 | 0.0000 | postive |
| WWTR1 | GAS7 | 0.5363 | 0.0000 | postive |
| WWTR1 | GPIHBP1 | 0.3972 | 0.0000 | postive |
| WWTR1 | GRK5 | 0.4336 | 0.0000 | postive |
| WWTR1 | GSN | 0.4599 | 0.0000 | postive |
| WWTR1 | HSPB2 | 0.3614 | 0.0000 | postive |
| WWTR1 | IGDCC4 | 0.4097 | 0.0000 | postive |
| WWTR1 | ITGA1 | 0.5044 | 0.0000 | postive |
| WWTR1 | JAM3 | 0.3256 | 0.0000 | postive |
| WWTR1 | KCNE4 | 0.5836 | 0.0000 | postive |
| WWTR1 | LAMA2 | 0.5204 | 0.0000 | postive |
| WWTR1 | LAMA4 | 0.6699 | 0.0000 | postive |
| WWTR1 | LATS2 | 0.5785 | 0.0000 | postive |
| WWTR1 | LDB2 | 0.4555 | 0.0000 | postive |
| WWTR1 | LHFPL6 | 0.5314 | 0.0000 | postive |
| WWTR1 | LRRC32 | 0.5069 | 0.0000 | postive |
| WWTR1 | LRRN4CL | 0.4128 | 0.0000 | postive |
| WWTR1 | MAP1A | 0.4596 | 0.0000 | postive |
| WWTR1 | MAP1B | 0.4821 | 0.0000 | postive |
| WWTR1 | MCAM | 0.6163 | 0.0000 | postive |
| WWTR1 | MXRA7 | 0.5553 | 0.0000 | postive |
| WWTR1 | MYADM | 0.5111 | 0.0000 | postive |
| WWTR1 | NES | 0.3754 | 0.0000 | postive |
| WWTR1 | NRP2 | 0.6084 | 0.0000 | postive |
| WWTR1 | NXPH3 | 0.3544 | 0.0000 | postive |
| WWTR1 | OGN | 0.4374 | 0.0000 | postive |
| WWTR1 | OLFML1 | 0.4178 | 0.0000 | postive |
| WWTR1 | P3H1 | 0.3161 | 0.0000 | postive |
| WWTR1 | PCDH18 | 0.4575 | 0.0000 | postive |
| WWTR1 | PDGFRA | 0.4713 | 0.0000 | postive |
| WWTR1 | PDZRN3 | 0.4994 | 0.0000 | postive |
| WWTR1 | PODN | 0.4731 | 0.0000 | postive |
| WWTR1 | PRKG1 | 0.6301 | 0.0000 | postive |
| WWTR1 | SCN1B | 0.3576 | 0.0000 | postive |
| WWTR1 | SERPINF1 | 0.3671 | 0.0000 | postive |
| WWTR1 | SH3RF3 | 0.5756 | 0.0000 | postive |
| WWTR1 | SLC24A3 | 0.3984 | 0.0000 | postive |
| WWTR1 | SLIT2 | 0.4921 | 0.0000 | postive |
| WWTR1 | SMOC2 | 0.5735 | 0.0000 | postive |
| WWTR1 | SNED1 | 0.4897 | 0.0000 | postive |
| WWTR1 | SPON1 | 0.4054 | 0.0000 | postive |
| WWTR1 | SRPX | 0.4461 | 0.0000 | postive |
| WWTR1 | STARD8 | 0.5345 | 0.0000 | postive |
| WWTR1 | SVEP1 | 0.3682 | 0.0000 | postive |
| WWTR1 | SYNC | 0.6174 | 0.0000 | postive |
| WWTR1 | TCF4 | 0.4369 | 0.0000 | postive |
| WWTR1 | TGFB1I1 | 0.6544 | 0.0000 | postive |
| WWTR1 | TGFB3 | 0.6200 | 0.0000 | postive |
| WWTR1 | THBS1 | 0.5294 | 0.0000 | postive |
| WWTR1 | TIMP2 | 0.4645 | 0.0000 | postive |
| WWTR1 | TNFAIP6 | 0.3656 | 0.0000 | postive |
| WWTR1 | TNFAIP8L3 | 0.5032 | 0.0000 | postive |
| WWTR1 | WISP1 | 0.3068 | 0.0000 | postive |
| WWTR1 | WISP2 | 0.3707 | 0.0000 | postive |
